# Pan-tumor activity of olomorasib, a next-generation KRAS G12C inhibitor in *KRAS* G12C-mutant advanced solid tumors: a first-in-human study

**DOI:** 10.1038/s41467-026-69943-7

**Published:** 2026-03-12

**Authors:** Yonina R. Murciano-Goroff, Antoine Hollebecque, Rebecca S. Heist, Philippe A. Cassier, Ji-Youn Han, So Yeon Kim, Joshua K. Sabari, Diego Tosi, Adrian Sacher, Timothy F. Burns, Toshio Shimizu, Natraj Reddy Ammakkanavar, Alexander Spira, Carlos Gomez-Roca, Amita Patnaik, Rasha Cosman, J. Nicholas Bodor, Misako Nagasaka, Arthur Xintian You, Samuel C. McNeely, Raimund Peter, Aaron Fink, Aaron Chen, Geoffrey R. Oxnard, Melinda D. Willard, Yasutoshi Kuboki, Takafumi Koyama

**Affiliations:** 1 Department of Medicine, Memorial Sloan Kettering Cancer Center, New York, NY USA; 2 Département d’Innovation Thérapeutique et Essais Précoces (DITEP), Gustave Roussy, Villejuif, France; 3 Department of Medicine, Massachusetts General Hospital, Boston, MA USA; 4 Centre Léon Bérard, Medical Oncology, Lyon, France; 5 National Cancer Center, Goyang, South Korea; 6 Yale University School of Medicine, New Haven, CT USA; 7 Perlmutter Cancer Center, NYU Langone Health, Long Island, New York, NY USA; 8 Institut régional du Cancer de Montpellier, Montpellier, France; 9 Department of Medical Oncology, Princess Margaret Cancer Center, University Health Network, University of Toronto, Toronto, ON Canada; 10 Department of Medicine, Division of Hematology Oncology, University of Pittsburgh Medical Center Hillman Cancer Center, Pittsburgh, PA USA; 11 Department of New Experimental Therapeutics, International Cancer New Drug Development Center, Kansai Medical University Hospital, NEXT Oncology KMU, Osaka, Japan; 12 Community Health Network, Indianapolis, IN USA; 13 Virginia Cancer Specialists, Fairfax, VA USA; 14 Institut Universitaire Du Cancer De Toulouse−Oncopole, Toulouse, France; 15 Department of Phase I Research, The START Center for Cancer Research, San Antonio, TX USA; 16 The Kinghorn Cancer Centre, St. Vincent’s Hospital, Darlinghurst, NSW Australia; 17 Faculty of Medicine, The University of New South Wales, Sydney, NSW Australia; 18 Fox Chase Cancer Center, Philadelphia, PA USA; 19 University of California, Irvine, Orange, CA USA; 20 Eli Lilly and Company, Indianapolis, IN USA; 21 Department of Experimental Therapeutics, National Cancer Center Hospital East, Kashiwa, Japan; 22 Experimental Therapeutics, National Cancer Center Hospital, Tokyo, Japan

**Keywords:** Drug delivery, Cancer therapy, Cancer therapy

## Abstract

This multicenter, first-in-human Phase 1 study (NCT04956640) evaluated olomorasib (LY3537982), a next-generation KRAS G12C inhibitor designed to enhance target occupancy at low absolute exposures. In total, data from 195 patients are reported: Phase 1a dose escalation (*n* = 112) assessed olomorasib monotherapy at 50, 100, 150 or 200 mg BID across *KRAS* G12C-mutant advanced solid tumors; the primary objective was to determine the recommended Phase 2 dose (RP2D) based on dose-limiting toxicities (DLTs). No DLTs occurred, and 150 mg BID was selected as the RP2D. The primary objective for the Phase 1b dose expansion (*n* = 83) was to evaluate the safety and tolerability of olomorasib in specific *KRAS* G12C-mutant tumor types. Olomorasib was well tolerated, with predominantly grade 1–2 treatment-related adverse events (TRAEs) and infrequent grade 3 TRAEs; no grade 4/5 TRAEs occurred. Secondary objectives evaluated the antitumor activity of olomorasib. Among 168 efficacy-evaluable patients, the ORR and median PFS were both higher in non-CRC solid tumors compared to CRC, including in patients with NSCLC who previously received a KRAS G12C inhibitor. Intracranial responses were observed in patients with untreated, active brain metastases. This may support the potential of next-generation KRAS G12C inhibitors to overcome limitations of earlier agents and justify further investigation of combination therapy.

## Abstract (web summary)

KRAS G12C inhibitors have improved outcomes but mainly in non-small cell lung cancer and colorectal cancer, despite it being a well-established oncogene in a range of cancer types. Here, the authors present a first-in-human phase I/II clinical trial investigating the next-generation KRAS G12C inhibitor, olomorasib, in KRAS G12C-mutant advanced solid tumors.

## Introduction

K*RAS*, the most commonly mutated oncogene across tumor types, has long been considered a challenging target. The discovery that the aberrant cysteine residue of KRAS G12C can enable binding of covalent inhibitors that traps the GTPase in its inactive, GDP-bound state^1^, has facilitated encouraging progress^2–4^. The FDA has now granted accelerated approval to two first-generation covalent inhibitors— sotorasib^5–7^ and adagrasib^8,9^ for non-small cell lung cancer (NSCLC) in the second-line and beyond, as well as for metastatic colorectal cancer (CRC) in combination with panitumumab (FDA Full Approval) and cetuximab (FDA Accelerated Approval), respectively, in previously treated patients. While this progress is encouraging, realizing the full benefit of this new therapeutic approach will likely require advancing these therapies earlier into the treatment course of NSCLC and other impacted cancer types^7,10,11^. Within NSCLC specifically, toxicity challenges in combining KRAS inhibition with PD-1 inhibition/PD-L1 inhibition have, in part, prevented the pursuit of earlier line use, and next-generation agents may be necessary to fully unlock this unmet need.

Importantly, activating *KRAS* mutations have also been observed in a range of solid tumors^12^, including *KRAS* G12C mutations in approximately 3% of CRCs, and 1–2% of other solid tumors^13–16^. However, there are currently no approved KRAS G12C targeted monotherapies for the heterogeneous group of histologies with *KRAS* G12C mutations beyond NSCLC^17^. Across cancer types, baseline co-alterations and the development of resistance mechanisms may impact the effectiveness and durability of KRAS-targeted therapies. CRC, which has the second-highest prevalence of *KRAS* G12C mutations^12^, is particularly challenging to treat due to EGFR-dependent lineage-specific feedback mechanisms that result in relative resistance to RAF/RAS inhibition^18,19^. As has been seen with BRAF inhibitors, this feedback diminishes the effectiveness of single-agent targeted therapy, motivating combination therapy with EGFR antibodies to achieve substantial responses in CRCs. This highlights the need for continued research on novel approaches to optimize and broaden the application of KRAS-targeted therapies beyond NSCLC, including the use of combination therapies where tolerable.

Olomorasib (LY3537982) was developed as an orally bioavailable, potent, and highly selective covalent inhibitor of KRAS G12C. In preclinical models, olomorasib demonstrated robust in vivo tumor growth inhibition and significant antitumor activity against a wide range of *KRAS* G12C-mutant tumor models^20^. Notably, olomorasib’s unique pharmacologic properties enable it to achieve >90% target occupancy at trough at low absolute exposure and doses^20^. Additionally, in vitro studies have shown olomorasib to be more potent than first-generation KRAS G12C inhibitors^20^. We thus hypothesized that the selectivity and potency of olomorasib would translate into improved safety as compared to first-generation agents as monotherapy, but most importantly enable safe combination with other first-line standard-of-care agents, including anti-PD-1 antibodies.

Here we report results from phase 1/2 LOXO-RAS-20001, a first-in-human study, providing the first evidence in manuscript form of olomorasib’s monotherapy clinical activity in patients with *KRAS* G12C-mutant advanced solid tumors. We aimed to demonstrate the feasibility of effective KRAS G12C inhibition without burdensome toxicity and to explore the activity of KRAS G12C across solid tumors.

## Results

### Study Design

**Fig. 1:**
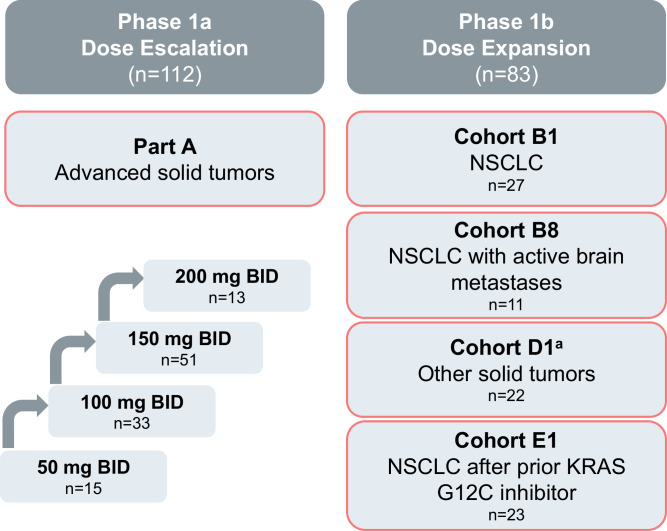
LOXO-RAS-20001 Trial Schema. Phase 1a included dose escalation and backfill cohorts, while Phase 1b included exploratory monotherapy expansion cohorts in select populations. **a** Includes solid tumors that are not NSCLC, CRC, or Pancreatic cancer. Abbreviations: BID twice a day, CRC colorectal cancer, NSCLC non-small cell lung cancer.

A total of 195 patients were treated in phase 1a dose escalation (*n* = 112) or phase 1b expansion cohorts (*n* = 83), including 26 unique solid tumor types harboring *KRAS* G12C mutations (Fig. 1, Supplementary Fig. 1) detected either in tumor tissue or circulating tumor DNA on local testing in a laboratory with CLIA, ISO/IEC, CAP, or other similar certification per local guidelines. Patients were enrolled from 46 sites across 6 countries, and the dataset includes those who started treatment between 29 Jul 2021–05 Jul 2024. Patients were generally heavily pretreated with a median of three prior lines of therapy (range, 0–11). Among the patients who received prior systemic therapy (97%, *n* = 189), 23% (44/189) had previously been treated with a KRAS G12C inhibitor (Supplementary Table 1). One specific cohort allowed patients with active, untreated measurable brain metastases (lesions ≥5 mm) and without associated neurological symptoms requiring urgent intervention with locoregional therapy (Fig. 1). Patients received 50 mg BID (*n* = 15), 100 mg BID (*n* = 33), 150 mg BID (*n* = 134), or 200 mg BID (*n* = 13) olomorasib in 21-day cycles. A data cutoff date of 05 July 2024 was used. Findings from the phase 1b cohorts of olomorasib in combination with pembrolizumab have been previously reported^21^.

### Phase 1a dose escalation/Phase 1b dose expansion

In phase 1a dose escalation, 112 patients received olomorasib monotherapy. Dose escalation was guided by rates of dose-limiting toxicities (DLTs) during a 21-day observation period using the modified toxicity probability interval (mTPI-2) method. For each therapeutically relevant dose level cleared in terms of safety, backfill cohorts were allowed while evaluating higher doses for DLTs. After completion of the 21-day DLT period, intrapatient dose escalation to the highest dose level cleared and deemed safe by the safety review committee (SRC) was allowed on a case-by-case basis, following Sponsor approval. Among the 112 patients, dosing was distributed as follows: 15 at 50 mg BID, 33 at 100 mg BID, 51 at 150 mg BID, and 13 at 200 mg BID of olomorasib. No DLTs were reported.

Pharmacokinetics (PK) of olomorasib was evaluated across all doses of 50-200 mg BID and showed a linear, dose-proportional increase in exposure, and predictable PK with exposures from 50-150 mg that plateaued between 150 and 200 mg, with a half-life of approximately 3 hours. Modeling indicated that 100 mg BID and above maintained target concentrations at or above TEC80 (target effective concentration to achieve 80% KRAS G12C inhibition) throughout the dosing interval (Supplementary Fig. 2). Consequently, for the monotherapy expansion, the 150 mg BID dose was chosen for investigation due to maintained concentrations above TEC80 for the entire dosing interval across all patients evaluated, no DLTs, and, as reported below in this manuscript, a similar safety profile to lower doses (predominantly grade 1-2 treatment-emergent adverse events [TEAEs] and infrequent grade 3 treatment-related adverse events [TRAEs]), aiming to optimize both therapeutic efficacy and safety. The 100 mg BID dose was additionally chosen for combination investigation due to its comparable PK profile. An additional 83 patients were treated in the phase 1b dose expansion at a starting dose of 150 mg BID and were assessed for safety as well as efficacy.

### Safety

Safety was assessed across all patients who received at least one dose of olomorasib monotherapy (*n* = 195). The median time on treatment with olomorasib was 5.6 months (range, 0.1–35.3). At the time of analysis, 56 patients (29%) remained on therapy and 139 (71%) patients discontinued treatment most commonly due to PD (*n* = 107) or withdrawal of consent (*n* = 15). Other reasons for discontinuation included adverse events (*n* = 6), death (*n* = 5), physician decision (*n* = 3), withdrawal by subject (*n* = 2), and to pursue subsequent anticancer radiation (*n* = 1).

Overall, 184 patients (94%) experienced at least one TEAE, and at least one TRAE occurred in 128 patients (66%). TRAEs were predominantly grade 1 (35%, Table 1). Any grade TRAEs occurring in ≥10% of patients were: diarrhea (22%), nausea (11%), and fatigue (11%); mostly grade 1 or 2. Grade 3 TRAEs occurred in 14 patients (7%) and there were no grade 4 or 5 TRAEs. TRAEs led to olomorasib dose holds in 25 patients (13%), dose reductions in 11 patients (6%), and olomorasib discontinuation in 2 patients (1%) due to hypersensitivity and diarrhea. Overall, safety profiles were comparable across doses, suggesting that safety is not dose-dependent (Supplementary Table 2). Focusing only on patients who had previously received immunotherapy, only 2 of 98 patients (2%) had grade 3 hepatotoxicity (both <10X ULN that did not require treatment discontinuation), including 1 of 30 patients who received immunotherapy within 12 weeks of their first dose of olomorasib (Supplementary Table 3).

**Table 1:** Treatment-emergent and treatment-related adverse events by grade

All Doses and Patients (50 – 200 mg BID) *N* = 195										
Adverse Event	TEAEs (≥10%)					TRAEs				
	Any Grade	Grade 1	Grade 2	Grade 3	Grade 4/5^a^	Any Grade	Grade 1	Grade 2	Grade 3^b^	Grade 4/5
Any AE, n (%)	184 (94)	28 (14)	73 (37)	76 (39)	7 (4)	128 (66)	69 (35)	45 (23)	14 (7)	0 (0)
Diarrhea	65 (33)	49 (25)	13 (7)	3 (2)	0 (0)	43 (22)	33 (17)	9 (5)	1 (1)	0 (0)
Fatigue	45 (23)	24 (12)	18 (9)	3(2)	0 (0)	21 (11)	12 (6)	8 (4)	1 (1)	0 (0)
Nausea	45 (23)	33 (17)	12 (6)	0 (0)	0 (0)	21 (11)	16 (8)	5 (3)	0 (0)	0 (0)
Constipation	36 (19)	25 (13)	11 (6)	0 (0)	0 (0)	6 (3)	6 (3)	0 (0)	0 (0)	0 (0)
Decreased appetite	28 (14)	17 (9)	9 (5)	2 (1)	0 (0)	13 (7)	9 (5)	3 (2)	1 (1)	0 (0)
Abdominal pain	27 (14)	17 (9)	8 (4)	2 (1)	0 (0)	5 (3)	4 (2)	0 (0)	1 (1)	0 (0)
AST increased	25 (13)	18 (9)	2 (1)	5 (3)	0 (0)	18 (9)	15 (8)	1 (1)	2 (1)	0 (0)
ALT increased	26 (13)	20 (10)	2 (1)	4 (2)	0 (0)	19 (10)	16 (8)	1 (1)	2 (1)	0 (0)
Vomiting	24 (12)	15 (8)	8 (4)	1 (1)	0 (0)	8 (4)	7 (4)	1 (1)	0 (0)	0 (0)
Arthralgia	21 (11)	16 (8)	5 (3)	0 (0)	0 (0)	3 (2)	2 (1)	1 (1)	0 (0)	0 (0)
Peripheral edema	21 (11)	15 (8)	6 (3)	0 (0)	0 (0)	6 (3)	5 (3)	1 (1)	0 (0)	0 (0)
Anemia	20 (10)	4 (2)	5 (3)	10 (5)	1 (1)	5 (3)	0 (0)	3 (2)	2 (1)	0 (0)

^a^Grade 5 AEs included multiple organ dysfunction syndrome (*n* = 2), respiratory failure (*n* = 1), cardiac arrest (*n* = 1), and glioma (*n* = 1), all of which were unrelated to the study drug.

^b^Other Grade 3 TRAEs included: rash/rash maculopapular (*n* = 3), neutropenia/neutrophil count decreased (*n* = 2), blood creatinine phosphokinase increased (*n* = 1), dyspnea (*n* = 1), and sunburn (*n* = 1).

Data cutoff date of 05 July 2024. Total % may be different from the individual components due to rounding.

Abbreviations: *AE* adverse event, *ALT* alanine aminotransferase, *AST* aspartate aminotransferase, *TEAE* treatment-emergent adverse event, *TRAE* treatment-related adverse event.

### Anti-tumor Activity

**Fig. 2:**
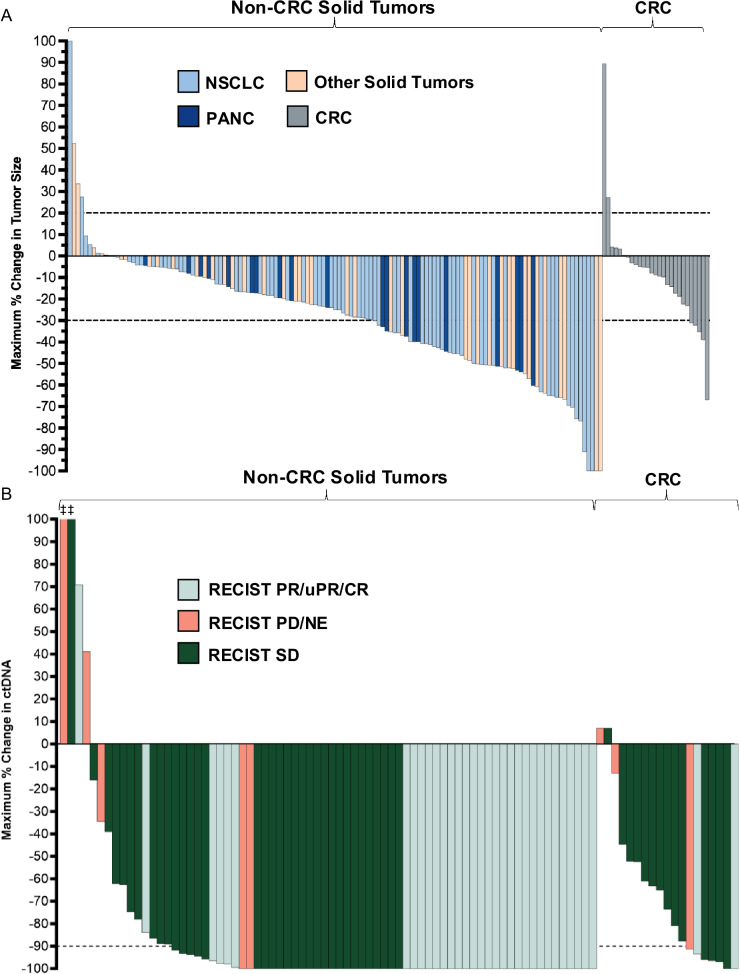
Anti-tumor activity of olomorasib. Studying both RECIST response (**A**) and ctDNA response (**B**), activity was greater in non-CRC solid tumors (left) versus CRC (right). Consistent RECIST responses were seen in NSCLC, pancreatic cancer, and other solid tumors. ctDNA clearance was common in non-CRC solid tumors, and was apparent in patients with and without a RECIST response. ‡ Indicates patient with ctDNA increase >100%. Source data are provided as a Source Data file. Abbreviations: CR complete response, CRC colorectal cancer, NE not evaluable, NSCLC non-small cell lung cancer, PANC pancreatic, PD progressive disease, PR partial response, SD stable disease, uPR unconfirmed partial response.

Efficacy analysis focused on doses at or above 100 mg BID where consistent activity was initially observed and PK modeling suggested optimal target occupancy (Supplementary Fig. 3). Overall, 168 efficacy evaluable patients were studied (Supplementary Fig. 1) using investigator-assessed RECIST v1.1, with a median follow-up of 13.1 months (95%CI, 10.2–13.8). As anticipated, monotherapy activity was greater in the 139 patients with non-CRC solid tumors (37.4% ORR, 95% CI, 29.4–46.0) as compared to the 29 patients with CRC (10.3% ORR, 95% CI, 2.2–27.4) (Fig. 2), with a disease control rate of 89.5% (95% CI, 82.0–93.3) in non-CRC compared to 82.8% (95% CI, 64.2–94.2) in CRC. Median PFS showed a similar trend with a more durable effect in non-CRC solid tumors (6.9 months; 95% CI, 5.5–7.9) than in CRC (4.2 months; 95% CI, 3.3–7.4) (Supplementary Fig. 4). Responses occurred sooner in patients with non-CRC solid tumors (median time to response (TTR) of 1.4 months; range, 1.1–9.5) than in patients with CRC (median TTR of 5.5 months; range, 1.4–7.7). However, in 55 patients with an objective response, the median duration of response was favorable both in non-CRC solid tumors (8.2 months; 95% CI, 5.8–12.5) and in CRC (10.4 months; 95% CI, 2.8–NE). Focusing on disease subsets, activity was consistent across NSCLC, pancreatic cancer and other non-CRC solid tumors (Supplementary Table 4), with responses observed in 14 unique histologies.

As an exploratory outcome, we investigated early circulating tumor DNA (ctDNA) kinetics on therapy using plasma next-generation sequencing (NGS) (FoundationOne Liquid CDx) to gain insight into target engagement. Of 134 patients with baseline sequencing data available, 112 had a *KRAS* G12C mutation detected at baseline with 91 of these patients also having data while on treatment, allowing assessment of ctDNA clearance (Fig. 2B, Supplementary Fig. 5). Circulating tumor DNA clearance was observed both in patients with and without an objective response on imaging (Fig. 2B, Supplementary Fig. 5). In non-CRC solid tumors, ctDNA clearance was seen in 48 (67%) patients and was seen across cancer types. In patients with CRC, while reductions in ctDNA VAF were common, ctDNA clearance was rare (*n* = 2, 11%), consistent with the reduced activity seen in this disease. We also explored baseline genomic profiles to better understand associations between co-occurring alterations and clinical response (Supplementary Fig. 5). Co-alterations in *STK11*, *KEAP1* and *SMARCA4* were frequently observed in patients with NSCLC, while co-alterations in *PIK3CA*, *PTEN* and *APC* were frequently found in CRC patients. While responses were observed in patients bearing some of these mutations, no clear trends in co-occurring alterations and response were observed; this may be due, at least in part, to the heterogeneity of the patient population and limited sample sizes for some of the mutated subgroups.

### Activity in populations of special interest: Intracranial metastases and patients previously treated with a KRAS G12C inhibitor

**Fig. 3:**
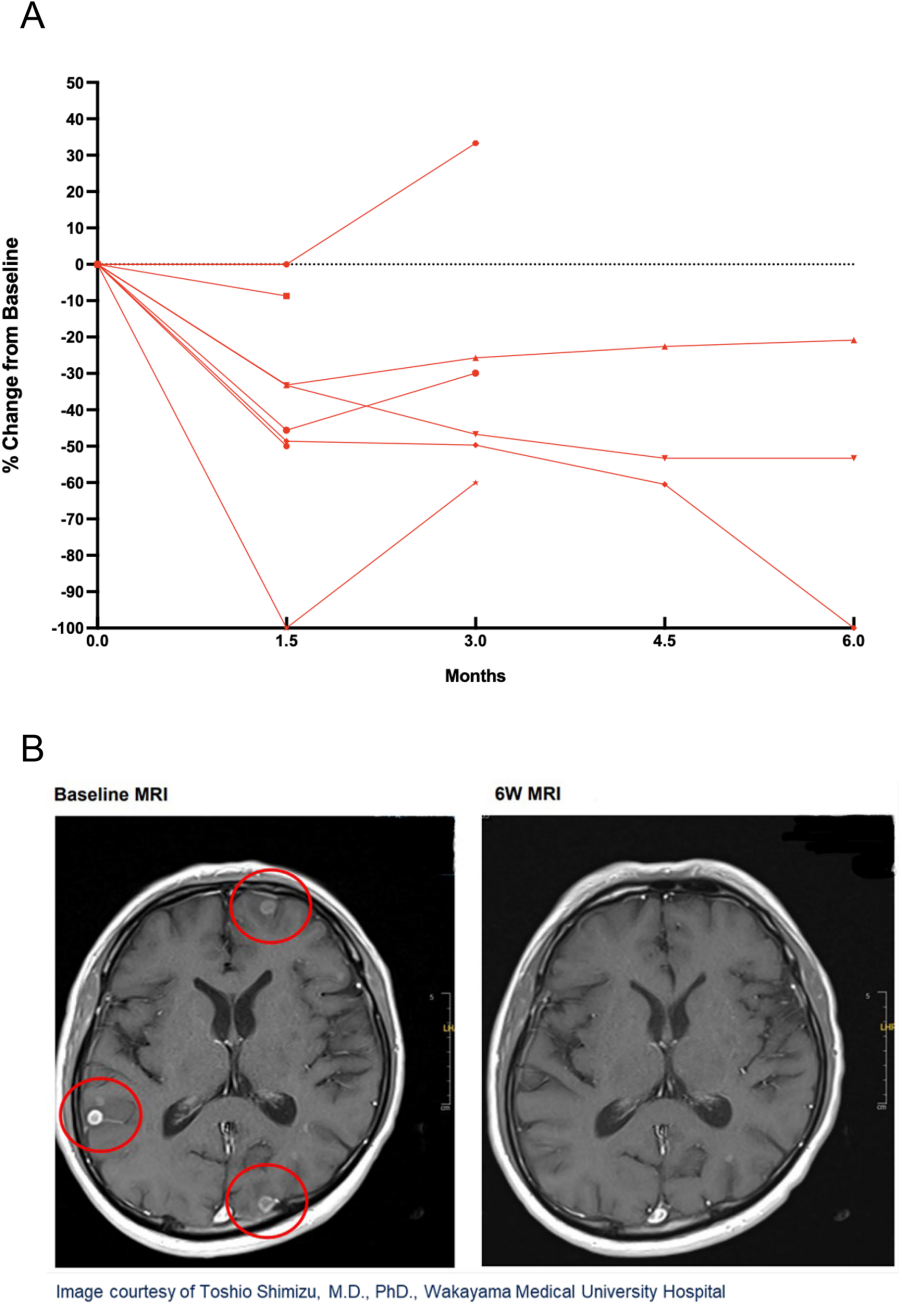
Intracranial activity of olomorasib. Of 11 patients enrolled with NSCLC and active, untreated brain metastases, on-treatment brain MRI was available for 9 (**A**) and demonstrated durable activity in a subset of patients. In one representative patient with NSCLC metastatic to brain and progression following systemic therapy and radiation (**B**), on-treatment brain MRI after 6 weeks showed resolution of measurable brain metastases. Source data are provided as a Source Data file. Abbreviations: MRI magnetic resonance imaging, NSCLC non-small cell lung cancer.

An additional aim of this study was to assess the preliminary antitumor activity of olomorasib in patients with untreated, active intracranial metastases using modified RECIST v1.1^22^. Eleven patients with NSCLC and measurable brain metastases at baseline were treated in cohort B8. While enrollment and follow-up are ongoing, in this preliminary assessment, we identified >30% tumor reduction in intracranial lesions in 6 of 9 patients with follow-up scans available (Fig. 3).

**Fig. 4:**
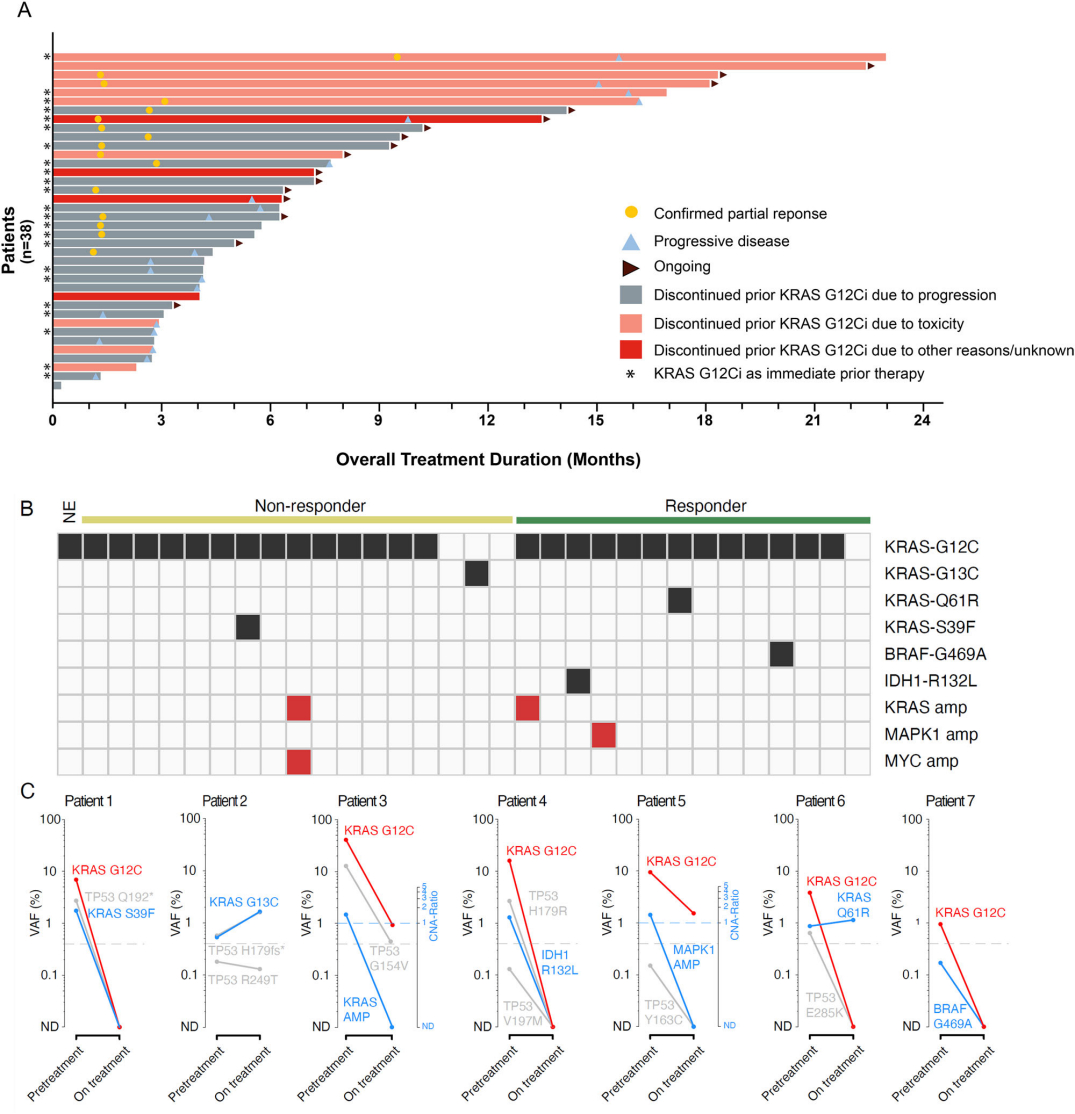
Activity of olomorasib after prior KRAS G12C inhibitor (KRAS G12Ci) treatment. Swimmer plot (**A**) demonstrates the potential for durable benefit from olomorasib in 38 patients previously treated with a KRAS G12Ci, with an ORR of 42% and median PFS of 8.2 months. Prolonged benefit was apparent in some patients, especially those who discontinued a prior KRAS G12Ci due to toxicity. **B** While ctDNA analysis identified putative resistance mutations present after prior KRAS G12Ci treatment there was no enrichment in non-responders. Serial ctDNA analysis (**C**) demonstrated a KRAS G12C molecular response (red) in most patients with putative resistance mutations (blue), questioning their biologic relevance. Source data are provided as a Source Data file. Abbreviations: ctDNA circulating tumor DNA, G12Ci G12C inhibitor, ORR overall response rate, PFS progression-free survival.

We further explored activity in the 38 efficacy evaluable patients who had received prior KRAS G12C inhibitors, all of whom had NSCLC (median time from prior KRAS G12C inhibitor exposure: 90 days, range: 3–721 days). Reasons these patients discontinued their most recent prior KRAS G12C inhibitor included progression (63%), toxicity (26%), and other/unknown (11%) (Fig. 4A). Activity was seen both in patients who discontinued prior KRAS G12C inhibitors due to progression and due to toxicity, though as expected treatment effect was more durable in patients without prior PD on a KRAS G12C inhibitor (Fig. 4A). Among the 38 patients who received prior KRAS G12C inhibitors, the ORR was 42% (95% CI, 26.3–59.2) with a median PFS of 8.2 months (95% CI, 4.3–15.6); ORR was similar (48%) in the 23 patients for whom a KRAS G12C inhibitor was their immediate prior therapy. Among the 10 patients that discontinued their prior KRAS G12C inhibitor due to toxicity, there were no grade ≥3 TRAEs or discontinuation of olomorasib due to tolerability.

Among patients who received olomorasib after prior KRAS G12C inhibition, 32 had plasma NGS from samples taken prior to olomorasib initiation (Fig. 4B). Of these patients, 21 also had paired on-treatment data (Cycle 2/Cycle 3) to characterize molecular response. In several cases we detected molecular and radiographic responses to olomorasib despite the presence of baseline genomic alterations in plasma which might be interpreted as associated with resistance to KRAS G12C inhibition, including^23,24^: *KRAS* amp, *BRAF* G469A, *KRAS* S39F, *MAPK1* amp, *KRAS* Q61H, *IDH1* R132L (Fig. 4C). However, a limitation of this study is the inability to rule out the possibility that some of these alterations were associated with clonal hematopoiesis. Only one case (patient 2 [Fig. 4C]) exhibited clear resistance as evidenced by a lack of both clinical and molecular responses: a patient with *KRAS* G13C detected in ctDNA despite a *KRAS* G12C mutation being detected in prior local tissue testing.

## Discussion

In this first-in-human trial of olomorasib, we demonstrate promising monotherapy activity in non-CRC solid tumors. In this heavily pretreated cohort of advanced solid tumors harboring *KRAS* G12C mutations, we observe monotherapy activity comparable to the efficacy described with prior KRAS G12C inhibitors across several tumor types^25^, including responses in untreated, active intracranial metastases^26^. The reduced activity seen in CRC is consistent with the historical challenges with monotherapy RAF/RAS inhibition in this cancer type and provides a strong rationale for combination treatment with cetuximab.

Our data suggest that olomorasib, a next-generation KRAS G12C inhibitor may be differentiated from existing KRAS G12C inhibitors in its tolerability, without compromising efficacy. In the design of olomorasib, we focused on the efficiency of covalent bond formation as reflected by the high Kinact/Ki, facilitating rapid target inactivation and overall high target occupancy at lower systemic drug concentrations, with the goal of less free drug and reduced rates of off-target toxicity^20^. As described, clinical data demonstrate that these pharmacologic properties do appear to help minimize or even avoid some of the burdensome toxicities, such as gastrointestinal events that have challenged other drugs in this class^5,8,20,27–30^. In contrast to other KRAS G12C inhibitors, which have reported diarrhea, nausea, and abdominal pain in up to 70%, 69%, and 21% of patients^5,8,27,29^, respectively, olomorasib demonstrates lower rates (33%, 23%, and 14%, respectively) of these common any grade treatment-emergent side effects. Other KRAS G12C inhibitors have also been shown to be associated with high-grade hepatotoxicity, including increases in AST and ALT. Hepatotoxicity was rare with olomorasib even following recent administration of checkpoint inhibitors, while other KRAS G12C inhibitors have seen an increased risk of high-grade hepatotoxicity in patients who received a KRAS G12C inhibitor within 3 months of checkpoint inhibitor treatment^31^. Furthermore, we did not observe a recurrence of high-grade hepatotoxicity in 6/7 patients who previously discontinued their prior KRAS G12C inhibitor due to hepatotoxicity. Approved KRAS G12C inhibitors have reported a meaningful rate of dose reduction (15–48%), and discontinuation (8–10%) due to TRAEs. In our trial, we observed a low rate of olomorasib dose reductions, seen in only 6% of patients, and a discontinuation rate of 1% due to TRAEs. The most common TRAE on olomorasib was diarrhea, which was predominantly grade 1; 1% of patients had grade 3, and no patients had grade 4/5 treatment-related diarrhea, whereas grade ≥3 treatment-related diarrhea has been described in 5–12% of patients receiving established KRAS G12C inhibitors^29,30^.

In the development of this molecule, both preclinically and clinically, we focused on delivering a drug that reduces the rates not only of high-grade AEs but importantly grade 2 AEs, as these can meaningfully contribute to the tolerability burden for continuously administered oral therapies. We expect this favorable safety profile will not only improve patient experience, but also maintain treatment compliance and give confidence in the potential to combine intentionally with possibly synergistic agents, such as immunotherapy, chemotherapy, and EGFR antibodies. Consistent with these expectations, a broad phase 3 development program in first-line advanced NSCLC of olomorasib in combination with standard-of-care immunotherapy or chemo-immunotherapy (SUNRAY-01 trial, NCT06119581) is ongoing. In August 2025, olomorasib was granted Breakthrough Therapy designation by the FDA for the following indication: olomorasib, in combination with pembrolizumab, for the first-line treatment of patients with unresectable advanced or metastatic NSCLC with a *KRAS* G12C mutation and PD‑L1 expression greater than or equal to 50%, as determined by FDA‑approved tests.

Unexpectedly, we observed that olomorasib monotherapy maintained activity even after prior treatment with a KRAS G12C inhibitor, including in patients with documented resistance to first-generation KRAS G12C inhibitors. While acquired resistance mutations within the switch II pocket^23,32^ that negatively impact drug potency have been well documented in this drug class^32^, it is noteworthy that these were not observed in any patients treated with olomorasib. These data provide additional supportive evidence that olomorasib is likely delivering more sustained and potent KRAS G12C inhibition, consistent with a next-generation pharmacophore. In patients who could not tolerate a prior KRAS G12C inhibitor due to toxicity, olomorasib demonstrated the ability to deliver durable efficacy and, importantly, tolerability, again consistent with a next-generation inhibitor. Preliminary evidence with olomorasib is encouraging; however, due to the early-stage nature of the study, conclusions drawn from cross-trial comparisons should be interpreted with caution. Additionally, limitations of this study, including the small number of patients in subgroups and the fact that a portion of patients did not report their race at baseline, should be noted.

With the emergence of a growing number of diverse KRAS inhibitors under development for cancer treatment, the optimal clinical efficacy and safety characteristics of KRAS G12C inhibition in advanced solid tumors are coming into greater focus. While G12C is the predominant *KRAS* mutation in NSCLC, *KRAS* G12D and G12V mutations are more prevalent overall and are more evenly distributed across gastrointestinal cancers, NSCLC, and other cancer types. Our data highlight the potential efficacy of KRAS G12C inhibition across diverse cancer types, though they suggest CRC may need separate consideration from other solid tumors in forthcoming KRAS inhibitor trials. As combination approaches are likely to be key in the development of emergent KRAS inhibitors, we find it reassuring that tolerable safety profiles are achievable with next-generation agents – potentially broadening the range of feasible clinical combination strategies.

In conclusion, olomorasib monotherapy shows promising efficacy and a favorable monotherapy safety profile across multiple types of *KRAS* G12C-mutated solid tumors, including cancers previously treated with a KRAS G12C inhibitor. The low AE incidence compared to other KRAS G12C inhibitors supports the opportunity of olomorasib to be combined intentionally with potentially synergistic therapeutic approaches. The continued progress in developing potent and selective KRAS inhibitors offers promise for the treatment of *KRAS*-mutant cancers in the years ahead.

## Methods

### Study Design

The study was conducted in accordance with the principles of the Declaration of Helsinki, Council for International Organizations of Medical Sciences International Ethical Guidelines, and applicable regulatory requirements. The study protocol was approved by the institutional review board (IRB) or independent ethics committee (IEC) at each participating site (Supplementary Table 5). All patients provided written informed consent before undergoing any study-specific screening tests or procedures.

LOXO-RAS-20001 is a global, multicenter, open-label, first-in-human, phase 1/2 study (NCT04956640) of olomorasib (LY3537982) in patients with KRAS G12C-mutant advanced solid tumors. This study was designed to evaluate the safety, tolerability, PK, pharmacodynamics (PD), and preliminary efficacy of olomorasib. Phase 1a (dose escalation) and the monotherapy cohorts of phase 1b (dose expansion) were included. In Phase Ia, the primary outcome was the determination of the recommended phase 2 dose (RP2D) based on DLTs assessed during cycle 1 and graded per NCI-CTCAE v5.0, while secondary outcomes included characterization of PK and PD, and exploratory outcomes comprised preliminary antitumor activity. The dose escalation phase evaluated patients who received oral olomorasib at 50 mg, 100 mg, 150 mg, or 200 mg twice daily in 21-day cycles. DLTs were assessed during cycle 1, graded per NCI Common Terminology Criteria for Adverse Events (NCI-CTCAE, version 5.0) based on investigator assessment, and defined to capture treatment-related grade ≥3 hematologic or nonhematologic toxicities. Dose escalation followed the mTPI-2 method, allowing for backfill to previously cleared dose levels that demonstrated therapeutically relevant exposures or direct evidence of clinical activity. In dose expansion, patients were enrolled into different cohorts based on tumor type. In Phase Ib, patients were enrolled into tumor-specific monotherapy cohorts, with preliminary efficacy as the primary outcome, safety and tolerability as secondary outcomes, and PK, PD, and additional biomarker analyses as exploratory outcomes. Findings from the phase 1b cohorts of olomorasib in combination with pembrolizumab have been previously published^21^.

Safety oversight was maintained by a study review committee (SRC), which ensured appropriate patient safety oversight throughout the study. The redacted protocol is available in the Supplementary Information.

### Patients

Patients ≥18 years old with locally advanced, unresectable and/or metastatic cancer per disease-specific criteria were enrolled. The protocol provides cohort-specific criteria. Patients included in the study needed to meet the following inclusion criteria: *KRAS* G12C mutations detected in tumor tissue or ctDNA locally, have measurable disease per Response Evaluation Criteria in Solid Tumors (RECIST) version 1.1; Eastern Cooperative Oncology Group performance status (ECOG PS) of 0-1; progressed or be intolerant to standard therapies; life expectancy ≥12 weeks; discontinued all previous treatments for cancer with improvement of any prior therapy-related AEs, with exceptions listed in the protocol. Prior KRAS G12C inhibitor therapy was permitted only for patients with NSCLC. In cohorts that allowed enrollment of individuals with NSCLC who progressed on a prior KRAS G12C inhibitor, individuals must have had a *KRAS* G12C mutation confirmed in a blood or tumor tissue sample collected within approximately 3 months of discontinuation of the prior KRAS G12C inhibitor due to disease progression, or at any time thereafter. Patients in cohort B8, who had NSCLC and untreated, active brain metastases (lesions ≥5 mm), must have had no local treatment to the area before the start of study treatment, and no neurological symptoms requiring urgent intervention with locoregional therapy. Individuals in all cohorts must have had adequate hematologic and organ function defined as platelet count ≥100,000/μL, absolute neutrophil count ≥1500/μL, hemoglobin ≥9 g/dL, total bilirubin ≤1.5 times the upper limit of normal (ULN), aspartate transaminase (AST) and alanine transaminase (ALT) ≤ 2.5 times ULN (if the liver has tumor involvement may have had AST and ALT ≤ 5.0 times ULN), creatinine clearance ≥50 mL/min using the Cockcroft–Gault, CKD-EPI, or MDRD equations; or direct measurement of serum creatinine or creatinine clearance in urine. Individuals must have agreed and adhere to contraceptive use by men or women that is consistent with local regulations regarding the methods of contraception for those participating in clinical studies.

Patients were excluded based on the following criteria: disease suitable for local therapy administered with curative intent; active fungal, bacterial, and/or active untreated viral infection, including HIV or viral (A, B, or C) hepatitis, with exceptions noted in the protocol. Individuals with a serious pre-existing medical condition(s) including ILD or severe dyspnea at rest and uncontrolled disease-related pericardial effusion or pleural effusion; Individual has clinically significant, active cardiovascular disease, unstable angina, or history of myocardial infarction within 6 months prior to planned start of LY3537982, or QTc of ≥470 msec on screening ECG as calculated using Fridericia’s formula (QTcF), with the exception of individuals with implanted pacemakers; Individual with a second active primary malignancy or has been diagnosed and/or treated for an additional malignancy within 3 years prior to enrollment with the exception of curatively treated basal cell carcinoma of the skin, nonmetastatic prostate cancer treated with observation only, squamous cell carcinoma of the skin, and/or curatively resected in situ cervical and/or breast cancers, with exceptions permitted following discussion with Sponsor. For all individuals except those in cohort B8: Have untreated active intracranial metastases and/or leptomeningeal disease; however, previously treated intracranial metastases were permitted to participate, provided they met the criteria listed in the protocol. Further exclusions for all cohorts included: individual has received prior treatment with any KRAS G12C small molecule inhibitor, except in phase 1a dose escalation backfill cohort (NSCLC only) and cohort E1 (which was specifically designed to enroll NSCLC with prior KRAS G12C inhibitor exposure). Individual is pregnant, breastfeeding, or expecting to conceive or father children within the projected duration of the trial, starting with the screening visit through 180 days after the last dose of study medication; known allergic reaction against any of the components of the study treatments; prior enrollment in another cohort in this study. Additional exclusion criterion specific to certain cohorts are listed in the protocol. In this study, patients were enrolled irrespective of sex. Any data on sex and gender was self-reported and collected at each clinical trial site. The terms sex and gender were used appropriately throughout the manuscript. Information on participant compensation is provided in the Reporting Summary.

### Study Objectives and Outcomes

The primary objective of the phase 1a dose escalation was to determine the recommended phase 2 dose (RP2D) of olomorasib. The phase 1b dose expansion further evaluated the safety and tolerability of olomorasib in specific solid tumor types. Secondary objectives were to characterize the PK, determine the preliminary anti-tumor activity of olomorasib, including disease control rate (DCR), overall response rate (ORR), duration of response (DOR), and progression-free survival (PFS) as assessed per RECIST v1.1, evaluate the intracranial ORR and DOR based on modified RECIST v1.1 and overall survival. Overall survival is not reported as the data was immature. Exploratory objectives included correlation of biomarker characteristics in tumor tissue or blood with clinical benefit and measurement of changes in biomarkers in response to study treatment and after progression.

### Study Assessments

Safety data included all patients who received at least one dose of the study drug and was assessed through the evaluation of all toxicities, using NCI CTCAE v5.0, based on the Investigator assessment, changes in laboratory test results, vital signs and electrocardiograms. The attribution of AEs to the study drug was determined by the investigator. For treatment-related AEs requiring a dose modification, specific guidance is provided in the protocol by AE. ORR was defined as the proportion of participants who achieved a CR or PR/unconfirmed partial response (uPR) out of all participants treated. All PR were confirmed except one which was pending confirmation and ongoing at time of analysis. BOR was categorized as CR, PR/uPR, SD, PD or NE (not evaluable), occurring between the first dose and the date of documented disease progression or the date of subsequent anticancer therapy, cancer-related surgery or anti-cancer radiation. All CR and PR responses were evaluated for confirmation by a second scan at least 4 weeks after the initial response. DCR was defined as the proportion of participants who have a BOR of CR, PR/uPR or SD. DOR was calculated for participants with CR or PR/uPR as their best overall response. DOR was defined as the number of months from the start date of the first documented response to the earlier of the documentation of PD or death from any cause. PFS was defined as the number of months from the date of the first dose to the earlier of the documentation of PD or death from any cause. Participants who were alive and without documented PD as of the data analysis cutoff were censored. For patients with radiographic progression, exceptions allowing for continuing study treatment were made on a case-by-case basis for participants who were believed to be clinically benefiting from study treatment, where the Investigator and the Sponsor agreed that continuing study treatment was in the participant’s best interest.

### Pharmacokinetics (PK)

Plasma samples were obtained to determine olomorasib concentration using a validated bioanalytical assay. PK analyses of olomorasib concentration results were conducted for participants who received at least 1 dose of the study drug and who had sufficient samples collected to allow for the estimation of olomorasib PK parameters. Olomorasib PK parameters were calculated using standard noncompartmental methods for analysis. Here, we report graphically the olomorasib AUC versus dose and the olomorasib concentration versus time profile.

### Biomarker Assessments

Peripheral blood was collected in Streck cfDNA Blood Collection Tubes, and cleared plasma was stored at -80 °C for retrospective analysis with FoundationOne Liquid CDx, a 324-gene next-generation sequencing panel^33^. Predose cycle 1-day 1 samples were used to evaluate baseline genomic profiles. Circulating tumor DNA kinetics were evaluated in patients in the efficacy analysis with a *KRAS* G12C mutation detected at cycle 1 day 1 (*n* = 112) and paired data from an on-treatment sample (*n* = 91). For the on-treatment assessment, a cycle 3, day 1 sample was used for most patients (*n* = 73), but a sample from day 1 of cycle 2 (*n* = 16), cycle 5 (*n* = 1), or cycle 7 (*n* = 1) was substituted in cases where a cycle 3 sample was unavailable or inadequate for testing. Circulating tumor DNA response was defined as at least 90% reduction in *KRAS* G12C variant allele frequency while clearance was defined as a lack of detection of *KRAS* G12C in the on-treatment sample.

### Statistical analysis

Patients were enrolled between 29 July 2021 and 05 July 2024. The data cutoff for all analyses was 05 July 2024. No formal power calculations were performed; sample sizes were determined to allow appropriate assessment of safety and anti-tumor activity. Randomization was not applicable as this was a non-randomized, open-label study. The safety analysis set included all enrolled patients who received at least 1 dose of olomorasib. For phase 1a and phase 1b, all efficacy analyses were conducted on the efficacy evaluable analysis set defined as all patients who had a baseline and ≥1 post-baseline response assessment or discontinued all treatment prior to the first post-baseline response assessment. Descriptive statistics were used to summarize the findings. For ORR, a 2-sided 95% confidence interval was estimated based on the exact binomial distribution; PFS was analyzed with the Kaplan-Meier method with 95% confidence intervals. In phase 1a, cohorts were allowed to backfill up to 40 patients, including up to 10 patients who received a prior KRAS G12C inhibitor. Results were analyzed using SAS 9.4.

### Software Details

Details of all software and tools used for statistical, PK, and biomarker analyses, including version numbers, are provided in Supplementary Table 6.

### Reporting summary

Further information on research design is available in the Nature Portfolio Reporting Summary linked to this article.

## Supplementary information

Supplementary Information

Reporting Summary

Transparent Peer Review file

## Source data

Source Data

## Data Availability

Eli Lilly and Company provides access to all individual data collected during the trial, after anonymization, with the exception of PK, genomic, or genetic data. Data are available to request 6 months after the indication studied has been approved in the US and EU and after primary publication acceptance, whichever is later. No expiration date of data requests is currently set once data are made available. Access is provided after a proposal has been approved by an independent review committee identified for this purpose and after receipt of a signed data sharing agreement. Data and documents, including the study protocol, statistical analysis plan, clinical study report, and blank or annotated case report forms, will be provided in a secure data sharing environment. For details on submitting a request, see the instructions provided at www.vivli.org. Source data are provided with this paper.
