# Supplementary material for: Pan-tumor activity of olomorasib, a next-generation KRAS G12C inhibitor in KRAS G12C-mutant advanced solid tumors: a first-in-human study
Source: Nat Commun. 2026 Mar 12;17:3834. doi: 10.1038/s41467-026-69943-7 (PMC13121619; doi:10.1038/s41467-026-69943-7)
Supplement: Supplementary file 1 — Supplementary Information [file 41467_2026_69943_MOESM1_ESM.pdf]

Supplementary Information

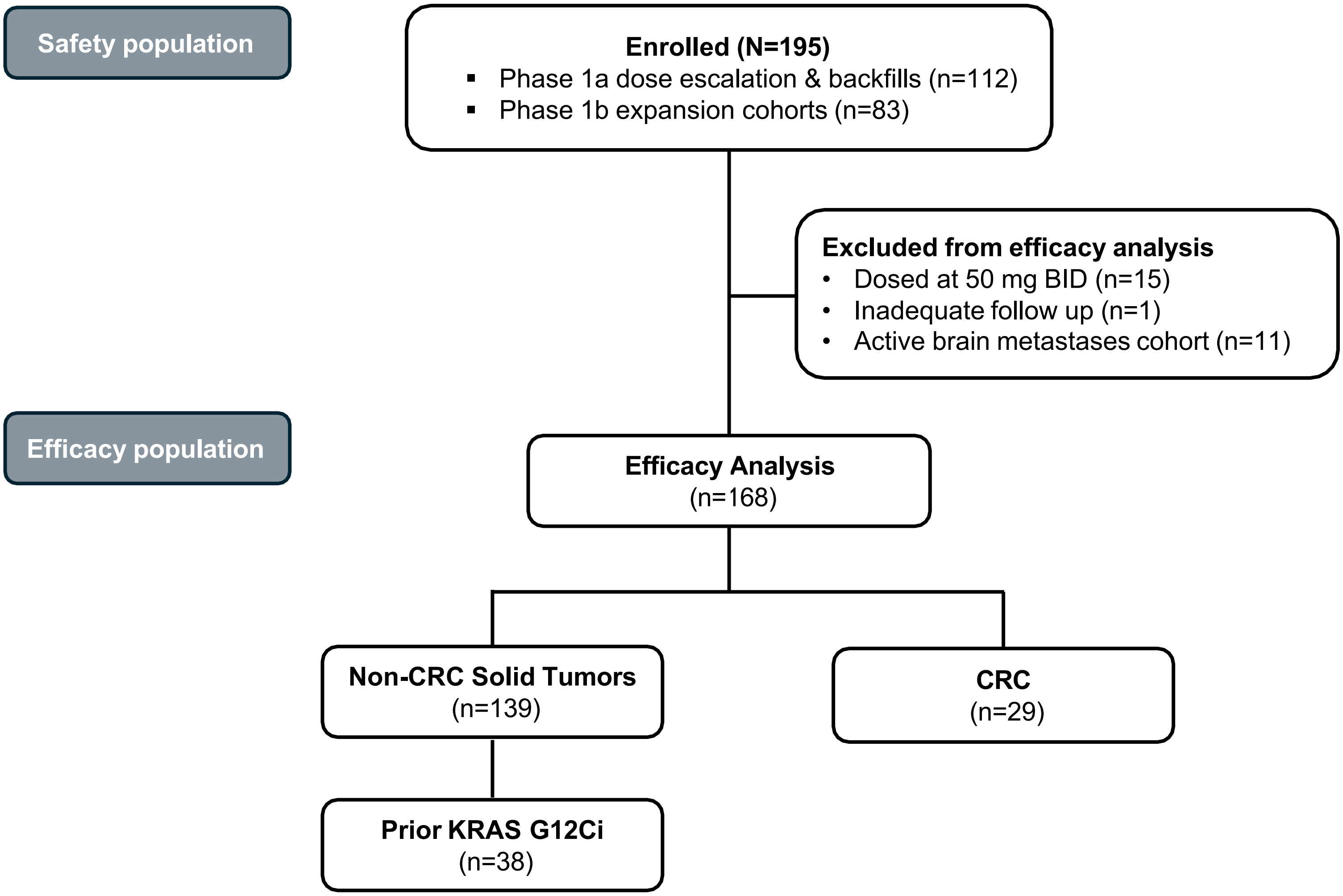

Supplementary Figure 1: Consort diagram.

Safety population included all 195 patients who received at least one dose of study drug. Efficacy analysis was limited to patients treated at 100 mg BID or greater with adequate follow-up. Intracranial response was studied separately in the cohort with active brain metastases.

Abbreviations: BID, twice a day; CRC, colorectal cancer; KRAS G12Ci, KRAS G12C inhibitor; NSCLC, non-small cell lung cancer.

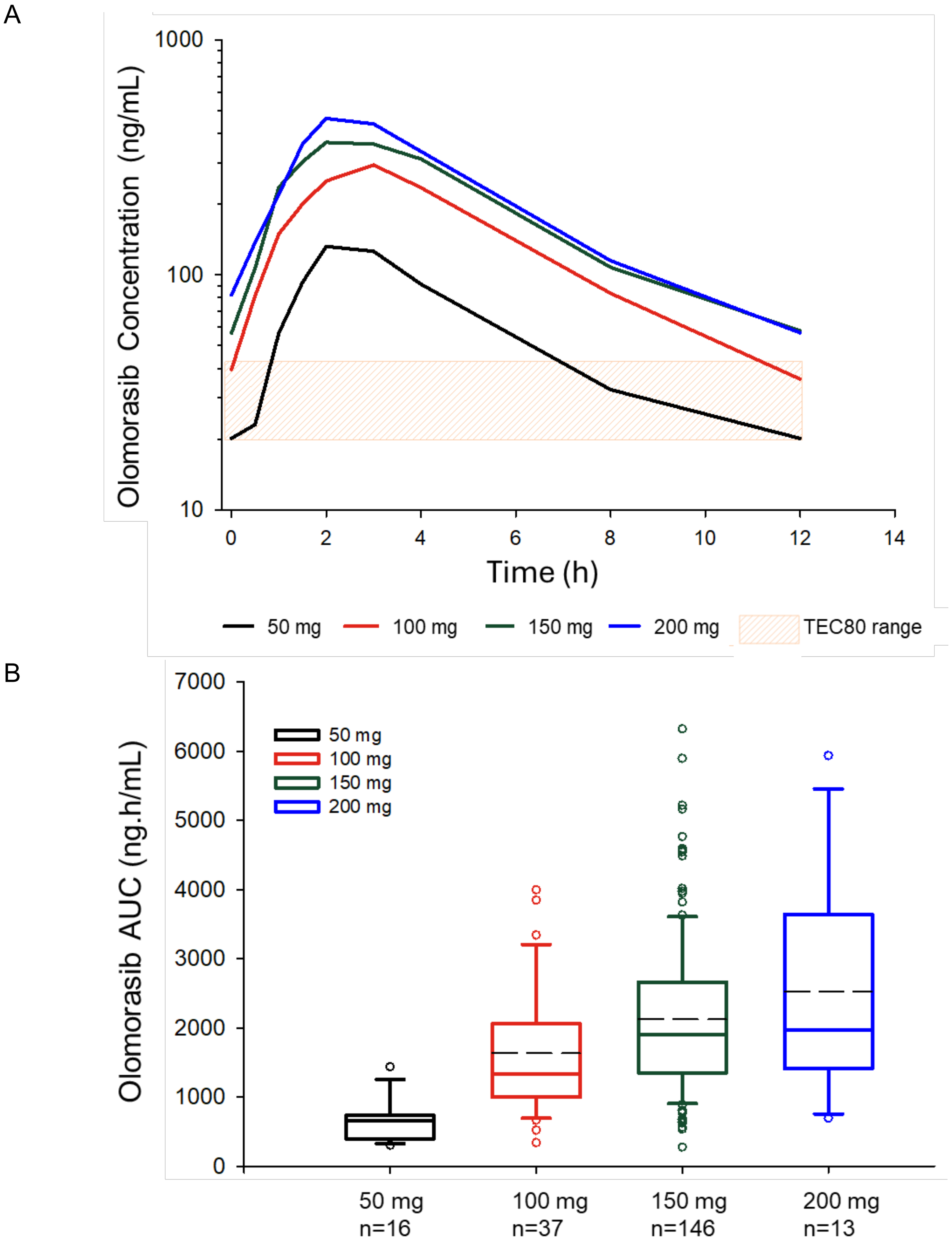

Supplementary Figure 2: Olomorasib PK across all doses studied. Olomorasib mean concentrations at steady-state versus time (A): maximum drug concentrations were observed at 3 hours post dose (median, range 0.5- 8 hours) with a terminal half-life of 2.96 hours (geomean) (2.79-3.14, 90%CI, N=140). This half-life resulted in negligible exposure accumulation from single to multiple BID dose of 1.15 (CV%=53%; N=136; 90% CI: 1.07, 1.24). When considering the predicted range of preclinical TEC80 (target effective concentration to achieve 80% KRAS G12C inhibition, shown in green) optimal target coverage is achieved at doses of 100 mg BID and above. Olomorasib steady state 12-hour exposure versus dose (B) AUC increases in a dose proportional fashion (linear pharmacokinetic) from 50 to 150 mg, resulting in a 2-fold increase in olomorasib average exposure from the 50 to 100 mg dose. Exposure distribution overlaps between the higher dose levels, particularly between 150 mg BID and 200 mg BID.
Source data are provided as a Source Data file.
Abbreviations: AUC, area under the curve; BID, twice daily; CI, confidence interval; h, hours; mg, milligram; ng/mL, nanogram per milliliter

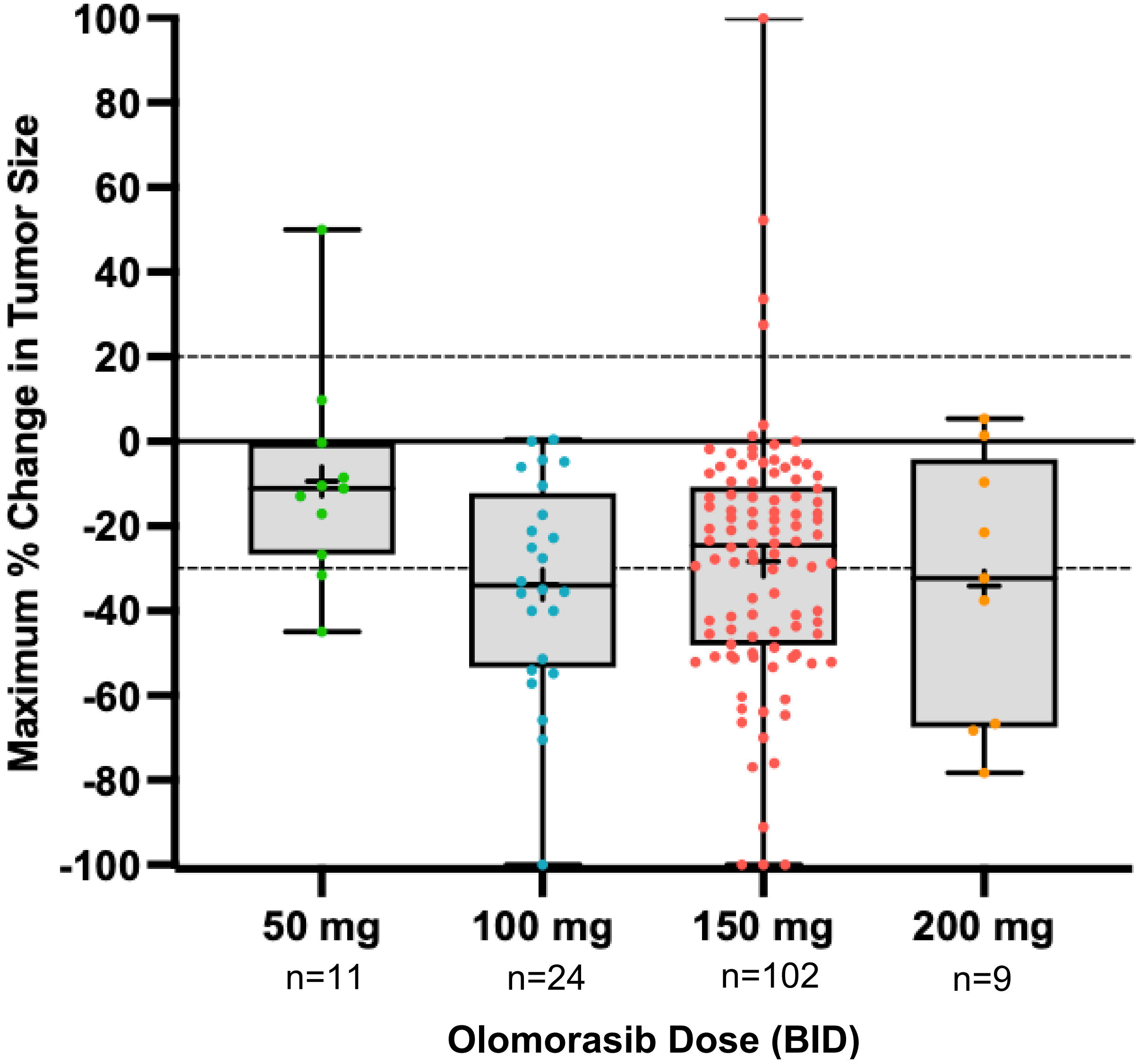

Supplementary Figure 3: Exploration of olomorasib activity by dose level. Studying 147 patients with non-CRC solid tumors and follow-up imaging on therapy, the depth of response was consistently deeper at doses of 100 mg BID and above; therefore, these doses were the focus of the efficacy analysis.
Source data are provided as a Source Data file.

Abbreviations: CRC, colorectal cancer.

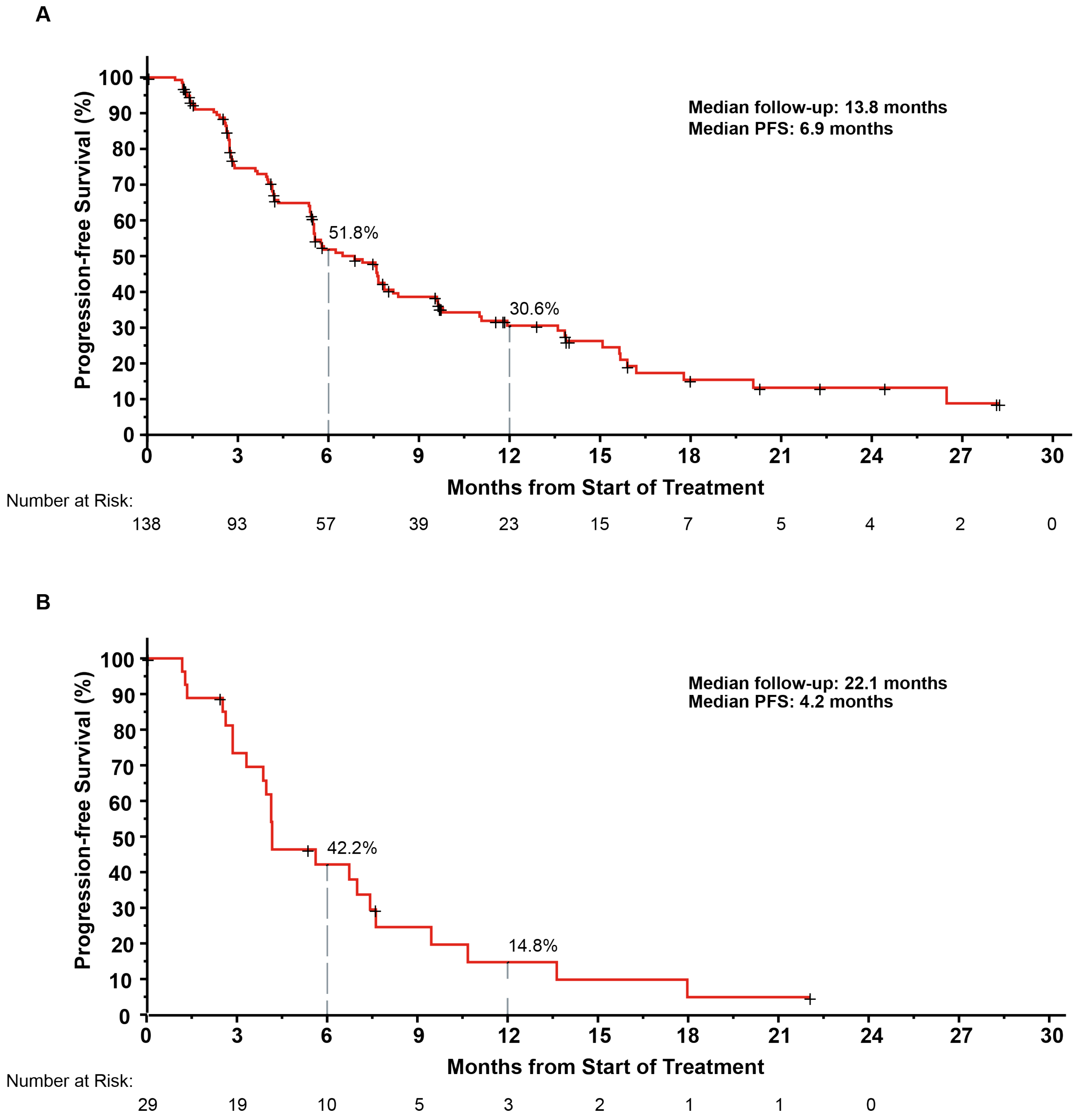

Supplementary Figure 4: Kaplan Meier curves for progression-free survival with olomorasib in patients with (A) non-CRC solid tumors, and (B) CRC. PFS trended longer in non-CRC solid tumors (6.9 months; 95% CI, 5.5-7.9) as compared to CRC (4.2 months; 95% CI, 3.3-7.4).
Source data are provided as a Source Data file.

Abbreviations: CI, confidence interval; CRC, colorectal cancer; PFS, progression-free survival.

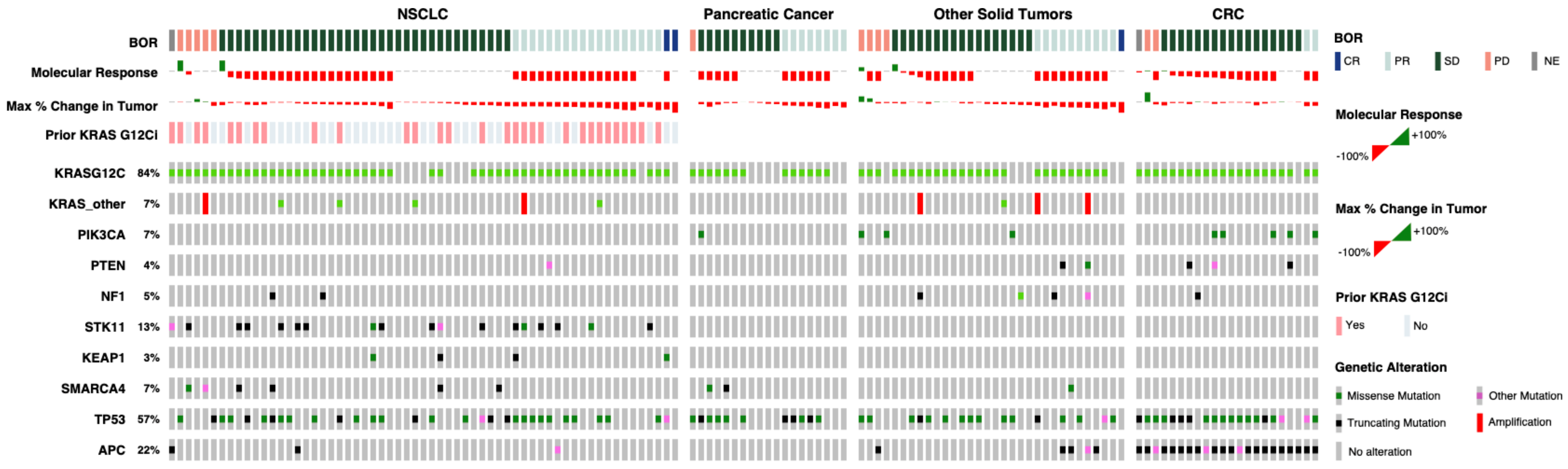

Supplementary Figure 5: Baseline Genomic Profiles. Oncoprint[1] visualization of the genetic landscape detected at baseline. After filtering for potential germline variants and alterations associated with clonal hematopoietic alterations, oncogenic alterations were annotated by OncoKB. Both oncogenes and tumor suppressor genes with more than 5% prevalence in the population and genes with relevant biology (PTEN in PI3K-mTOR pathway, and KEAP1 in NRF2 pathway) were visualized. Co-occurring mutations in STK11, KEAP1 and SMARCA4 were more frequently observed in NSCLC. Co-occurring mutations in PIK3CA, PTEN and APC were more frequently observed in CRC.
Source data are provided as a Source Data file.

Abbreviations: BOR, best overall response; CRC, colorectal cancer; CR, complete response; G12Ci, G12C inhibitor; NE, not evaluable (BOR); NSCLC, non-small cell lung cancer; PD, progressive disease; PFS, progression-free survival; PR, partial response; SD, stable disease.

Gao J, et al. *Sci Signal*. 2013.

Supplementary Table 1. Baseline characteristics of overall population

Characteristics (All Doses and Patients)[a]	Overall N=195
**Median age, years (range)**	65 (21-85)
**Sex, n (%)**	
Male	102 (52)
Female	93 (48)
**Race, n (%)**	
White	80 (41)
Asian	68 (35)
Black or African American	2 (1)
Not reported/Unknown	44 (23)
**ECOG PS, n (%)**	
0	87 (45)
1	106 (54)
2[b]	2 (1)
**Tumor Type**	
Non-CRC solid tumors	163 (84)
NSCLC	94 (48)
Pancreatic	24 (12)
Other solid tumors[c]	45 (23)
Colorectal cancer	32 (16)
**Brain metastases**	
No	147 (75)
Previously treated, stable	43 (22)
Active, untreated, measurable	11 (6)
**Median number of prior lines systemic therapies, (range)**	3 (0-11)
**KRAS G12C inhibitor pretreated NSCLC, n (%)**	44 (23)
Reason discontinued most recent KRAS G12C inhibitor, n (%)[d]	
Progressive disease	28 (64)
Toxicity	11 (25)
Other/Unknown	5 (11)

Data cutoff date of 05 July 2024. Total % may be different from the individual components due to rounding

[a]4 doses were evaluated: 50, 100, 150, 200 mg BID olomorasib.

[b]2 patients had a change in ECOG PS from 1 at screening to 2 by Cycle 1 Day 1.

[c]Other solid tumor types include cholangiocarcinoma (n=7), ovarian (n=7), duodenal (n=3), ampullary (n=2), bile duct (n=4), bladder (n=1), endometrial (n=3), gastric (n=1), gastric tubular (n=1), small intestine (n=2), uterine carcinosarcoma (n=1), cervical (n=1), chondrosarcoma (n=1), esophageal (n=1), goblet cell (n=1), jejunal adenocarcinoma (n=1), large cell neuroendocrine of lung (n=1), nasal malignant melanoma (n=1), pseudomyxoma peritonei (n=1), salivary adenoid cystic carcinoma (n=1), tracheal basaloid squamous cell carcinoma (n=1), urachal (n=2), and upper tract urothelial carcinoma (n=1).

[d] Calculated as percentage of patients who received most recent KRAS G12C inhibitor.

Abbreviations: CRC, colorectal cancer; NSCLC, non-small cell lung cancer.

Supplementary Table 2: Summary of treatment-emergent adverse events categorized by dose, as listed in Table 1

All Doses and Patients (50 – 200 mg BID) N=195								
Adverse Event	TEAEs, %							
	50 mg BID n=15		100 mg BID n=33		150 mg BID n=134		200 mg BID n=13	
	Any Grade	Grade ≥3	Any Grade	Grade ≥3	Any Grade	Grade ≥3	Any Grade	Grade ≥3
Any AE	100	53	97	52	93	36	100	39
Diarrhea	40	7	39	0	33	1	15	8
Nausea	27	0	30	0	21	0	23	0
Fatigue	20	0	18	0	22	2	54	0
Constipation	33	0	24	0	15	0	23	0
Decreased appetite	20	0	15	6	13	0	15	0
Abdominal pain	7	0	15	0	13	2	23	0
ALT increased	13	0	18	0	13	3	8	0
AST increased	13	0	21	0	11	4	8	0
Vomiting	13	0	24	0	10	1	0	0
Arthralgia	27	0	12	0	10	0	0	0
Peripheral edema	7	0	12	0	11	0	8	0
Anemia	13	13	15	6	9	5	8	8

Data cutoff date of 05 July 2024. Total % may be different from the individual components due to rounding.

Abbreviations: AE, adverse event; ALT, alanine aminotransferase; AST, aspartate aminotransferase; TEAE, treatment-emergent adverse event.

Supplementary Table 3: Hepatotoxicity by prior immunotherapy

	No Immunotherapy n=99	Prior Immunotherapy per Interval Between Last Immunotherapy and Olomorasib n=96		
		≤6 weeks n=13	6 to 12 weeks n=17	≥12 weeks n=66
**Treatment-emergent hepatotoxicity**				
Any grade, n (%)	19 (19%)	3 (23%)	4 (24%)	8 (12%)
Grade ≥3, n (%)	3 (3%)	0	1 (6%)	1 (2%)
**Worst grade hepatotoxicity on study**				
Grade 1, n (%)	14 (14%)	3 (23%)	2 (12%)	7 (11%)
Grade 2, n (%)	2 (2%)	0	1 (6%)	0
Grade 3, n (%)	3 (3%)	0	1 (6%)	1 (2%)
Grade 4, n (%)	0	0	0	0
**Treatment modification due to hepatotoxicity**				
Interruption or reduction, n (%)	4 (4%)	0	2 (12%)	1 (2%)
Treatment discontinuation, n	0	0	0	0

Supplementary Table 4: Efficacy Summary Across Subgroups

	Non-CRC solid tumors				CRC n=29
	**All** n=139	**NSCLC** n=76	**Pancreatic Cancer** n=22	**Other solid tumors** n=41	
**BOR**, n (%)					
CR	3 (2)	2 (3)	0 (0)	1 (2)	0 (0)
PR[a]	49 (35)	23 (30)	9 (41)	17 (42)	3 (10)
SD	71 (51)	41 (54)	11 (50)	19 (46)	21 (72)
PD	12 (9)	6 (8)	2 (9)	4 (10)	3 (10)
NE	4 (3)	4 (5.3)	0 (0)	0 (0)	2 (7)
**ORR,** % (95%CI)	37 (29.4-46.0)	33 (22.5-44.6)	41 (20.7-63.6)	44 (28.5-60.3)	10 (2.2-27.4)
**DCR**, % (95%CI)	89 (82.0-93.3)	87 (77.1-93.5)	91 (70.8-98.9)	90 (76.9-97.3)	83 (64.2-94.2)
**Median PFS,** months (95%CI)	6.9 (5.5-7.9)	6.5 (5.4-9.8)	5.7 (2.8-9.6)	7.1 (5.4-9.7)	4.2 (3.3-7.4)
**Median DOR**, months (95%CI)	8.2 (5.8-12.5)	13.1 (6.2-NE)	6.9 (4.3-11.1)	6.1 (4.2-10.9)	10.4 (2.8-NE)
**Time to Response**					
**Median,** months (IQR)	1.4 (1.1-9.5)	1.4 (1.1-9.5)	2.8 (1.2-5.6)	1.4 (1.1-6.8)	5.5 (1.4-7.7)
**Molecular Response**	**n=72**	**n=39**	**n=11**	**n=22**	**n=19**
**ctDNA response**[b], n (%)	57 (79)	30 (77)	10 (91)	17 (77)	7 (37)
**ctDNA clearance**[c], n (%)	48 (67)	27 (69)	7 (64)	14 (64)	2 (11)

[a]All PR were confirmed except one which was pending confirmation and ongoing at time of analysis.
[b]ctDNA response defined as a 90% reduction in KRAS G12C variant allele frequency
[c]ctDNA clearance defined as lack of detection of the KRAS G12C mutation on therapy

Abbreviations: BOR, best overall response; CRC, colorectal cancer; CR, complete response; ctDNA, circulating tumor DNA; DCR, Disease control rate; DOR, duration of response; NE, not evaluable (BOR); NE, not estimable (DOR 95% CI); NSCLC, non-small cell lung cancer; ORR, objective response rate; PD, progressive disease; PFS, progression-free survival; PR, partial response; SD, stable disease; TTR, time to response; uPR, unconfirmed partial response.

Supplementary Table 5: List of institutional review board (IRB) or independent ethics committee (IEC) at each participating site.

Organization
Asan Medical Center Institutional Review Board
Bellberry Human Research Ethics Committee<br>(Ref no: REGIS #: 2021/ETH01236)
Castle IRB
Chonnam National University Hwasun Hospital, Institutional Review Board
Comité de Protection des Personnes (CPP) Sud Ouest Et Outre Mer IV – Limoges<br>(Ref no: 21.02987.000027)
Dana-Farber Cancer Institute IRB
Dartmouth-Hitchcock Health Institutional Review Board
Health Research Ethics Board of Alberta (HREBA)
Hokkaido University Hospital Institutional Review Board
Institutional Review Board-Fox Chase Cancer Center
Kanazawa University Hospital Institutional Review Board
Mass General Brigham Incorporated IRBs
Memorial Sloan Kettering Cancer Center Institutional Review Board
National Cancer Center Institutional Review Board
National Cancer Center IRB
NYU Grossman School of Medicine IRB
Salus IRB
Seoul National University Hospital Institutional Review Board
The Catholic University of Korea St. Vincent's Hospital IRB
The Institutional Review Board of Aichi Cancer Center Hospital
University Health Network Research Ethics Board
University of Southern California Institutional Review Board
Vanderbilt University IRB
Wakayama Medical University Institutional Review Board
WIRB/Copernicus Group (WCG) IRB

Supplementary Table 6: Software Details and Purpose

Software	Version & Copyright	Purpose & Notes
SAS	9.4	Statistical analysis – No custom code used; all analyses performed in SAS
R	v4.4.0	Biomarker analysis – Used unless otherwise specified
Phoenix 32	v8.3<br>© Certara L.P	PK figure – Summary stat analysis of concentration data per dose and protocol nominal time; output exported as CSV
RStudio Pro	2023.12.1 build 402.prol © Posit Software PBC<br>(R v4.3.2)	PK figure – Created graph for summary stats
Phoenix 32	v8.3<br>© Certara L.P	Supplemental Figure 2B – NCA analysis and generation of AUC; output exported as XLS
SigmaPlot for Windows	v11.0<br>© Systat Software Inc	Supplemental Figure 2B – Graphical representation

Supplementary References:

1. Gao J, Aksoy BA, Dogrusoz U, et al. Integrative analysis of complex cancer genomics and clinical profiles using the cBioPortal. *Sci Signal*. 2013 Apr 2;6(269):pl1.doi: 10.1126/scisignal.2004088.

CLINICAL PROTOCOL [redacted]

A Phase 1/2 Study of LY3537982 in Patients with *KRAS* G12C-Mutant Advanced Solid Tumors

**Investigational Product:**	LY3537982
**Protocol Number:**	LOXO-RAS-20001
**Development Phase:**	1/2
**IND #:**	153658
**EudraCT#:**	2021-000595-12
**EU Trial Number:**	2022-502756-31-00
**Sponsor:**	Eli Lilly and Company<br>Indianapolis, Indiana, USA 46285

Confidentiality Statement

The information contained in this document is confidential and is intended for the use of clinical investigators. It is the property of Eli Lilly and Company or its subsidiaries and should not be copied by or distributed to persons not involved in the clinical investigation of LY3537982, unless such persons are bound by a confidentiality agreement with Eli Lilly and Company or its subsidiaries.

Note to Regulatory Authorities: This document may contain protected personal data and/or commercially confidential information exempt from public disclosure. Eli Lilly and Company requests consultation regarding release/redaction prior to any public release. In the United States, this document is subject to Freedom of Information Act (FOIA) Exemption 4 and may not be reproduced or otherwise disseminated without the written approval of Eli Lilly and Company or its subsidiaries.

PROTOCOL APPROVAL PAGE

**Protocol Title:**	A Phase 1/2 Study of LY3537982 in Patients with *KRAS* G12C-Mutant Advanced Solid Tumors
**Protocol Number:**	LOXO-RAS-20001
**Current Version:**	11.0

The current version of the protocol has been reviewed and approved.

The e-signature can be found at the end of this document.

____ _________________

PPD
PPD
Date

I have carefully read Protocol LOXO-RAS-20001 entitled "A Phase 1/2 Study of LY3537982 in Patients with *KRAS* G12C-Mutant Advanced Solid Tumors." I confirm that I have read and agree to conduct the clinical study as outlined in the protocol and in compliance with Good Clinical Practice (GCP), the Declaration of Helsinki as amended, and all other applicable regulatory requirements. Furthermore, I understand that the Sponsor, Eli Lilly and Company, and the Institutional Review Board/Research Ethics Board/Independent Ethics Committee (IRB/REB/IEC) must approve any changes to the protocol in writing before implementation.

I agree on behalf of myself, and all other personnel involved in the clinical study who are employed by me, to maintain confidentiality of all information received or developed in connection with this protocol. All data pertaining to this study will be provided to Eli Lilly and Company (Lilly) and any presentation or publication of study data will be reviewed by Lilly before release.

____ _________________

Principal Investigator's Signature   Date

____

Print Principal Investigator's Name

Protocol Amendment Summary of Changes Table

DOCUMENT HISTORY	
**Document**	**Date**
Version 10.0	15 January 2025
Version 9.0	09 April 2024
Version 8.0	16 February 2024
Version 7.0	27 September 2023
Version 6.0	16 December 2022
Version 5.0	13 May 2022
Version 4.0	14 December 2021
Version 3.0	09 November 2021
Version 2.0	04 May 2021
Version 1.0	17 March 2021

Amendment 11.0

This amendment is considered to be nonsubstantial.

Overall rationale for the amendment

The key change in this amendment is to add a 25 mg capsule of LY3537982 to the list of available interventions.

Section # and Name	Description of Change	Brief Rationale
Title Page	Moved protocol history (list of previous amendments and approval dates) to a new section called "Protocol Amendment Summary of Changes Table"	Align with current protocol template
Protocol Amendment Summary of Changes Table	Added a new section listing the changes in this amendment along with a brief rationale, and included a statement of the nonsubstantiality of this protocol amendment	Align with current protocol template
5.1, Study Intervention(s) Administered	Added 25 mg capsule to the list of available dose strengths for LY3537982	Update to match current clinical supply
7.1.1, Trial Schedule of Assessments Table 18	Consolidated footnotes "c" and "d" into footnote "c"	Correction, as these were accidentally separated in the prior amendment
7.1.1, Trial Schedule of Assessments, Table 20	In the 12-lead ECG row, formatted the list of central triplicate ECG assessments in the "Comments" column as a bulleted list	Clarification

SYNOPSIS

Title
A Phase 1/2 Study of LY3537982 in Patients with *KRAS* G12C-Mutant Advanced Solid Tumors

Protocol Number
LOXO-RAS-20001
EudraCT#
2021-000595-12
EU Trial Number
2022-502756-31-00

Study Sites: 49

Phase
Phase 1/2

Objectives

**Primary Objective Phase 1a Dose Escalation (Part A)**	• To determine the recommended Phase 2 dose (RP2D) of LY3537982 monotherapy in participants with *KRAS* G12C-mutant advanced solid tumors, as assessed by:<br>• Dose-limiting toxicities (DLTs)<br>• Adverse events (AEs) and serious adverse events (SAEs), changes in hematology and blood chemistry values, assessments of physical examinations, vital signs, and electrocardiograms (ECGs)
**Primary Objective Phase 1b Dose Expansion (Parts B to E)**	• To assess the safety and tolerability of LY3537982 when administered alone or in combination with other investigational agents in participants with advanced solid tumors with *KRAS* G12C mutation, in the following subgroups:<br>• Part B: Non-small cell lung cancer (NSCLC)<br>• Part C: Colorectal cancer (CRC)<br>• Part D: Other solid tumors (except NSCLC, CRC, and pancreatic cancer)<br>• Part E: NSCLC who have previously been treated with a *KRAS* G12C inhibitor
**Primary Objective Phase 1b Dose Optimization (Part G)**	• To determine the optimal dose of LY3537982 to be administered to treatment-naïve participants with advanced NSCLC in combination with pembrolizumab, as assessed by endpoints including, but not limited to treatment-emergent AEs (TEAEs), SAEs, deaths, and clinical laboratory abnormalities

**Primary Objective Phase 1b Dose Optimization (Part H)**	• To determine the optimal dose of LY3537982 in combination with cetuximab to be administered to participants who have received at least one prior oxaliplatin- or irinotecan-containing regimen for advanced or metastatic CRC, as assessed by endpoints including, but not limited to TEAEs, SAEs, deaths, and clinical laboratory abnormalities
**Primary Objective Phase 2 (Part F)**	• To assess the antitumor activity of LY3537982 monotherapy in participants with advanced pancreatic cancer with KRAS G12C mutation, as assessed by ORR determined by IRC using RECIST v1.1
**Secondary Objectives**	• To assess the preliminary antitumor activity of LY3537982 when administered alone or in combination with other investigational agents in participants with advanced solid tumors with *KRAS* G12C mutation using Response Evaluation Criteria in Solid Tumors version 1.1 (RECIST v1.1 [as assessed by Investigator for Phase 1a/1b and as assessed by Investigator and IRC for Phase 2 (Part F)]) based on evaluation of:<br>• Objective response rate (ORR)<br>• Best overall response (BOR)<br>• Duration of response (DOR)<br>• Time to response (TTR)<br>• Disease control rate (DCR)<br>• Progression-free survival (PFS)<br>• Overall survival (OS)<br>• Intracranial ORR based on modified RECIST v1.1 (Cohort B8 only)<br>• Intracranial DOR based on modified RECIST v1.1 (Cohort B8 only)<br>• To characterize the pharmacokinetic (PK) properties of LY3537982 when administered alone or in combination with other investigational agents in participants with advanced solid tumors with *KRAS* G12C mutation.<br>• Plasma concentrations of LY3537982, pemetrexed, carboplatin, and cisplatin; serum concentrations of pembrolizumab (Cohort B4 only) and cetuximab
**Exploratory Objectives**	• To correlate PK/pharmacodynamics changes<br>• To correlate biomarker characteristics in tumor tissue or blood with clinical benefit (e.g., including, but not limited to, somatic genetic variants in the *STK11*, *KEAP1*, and *TP53* genes)<br>• To measure changes in biomarkers in response to study treatment and after progression<br>[redacted]

	CCI [redacted]

Study Design

Study LOXO-RAS-20001 is a first-in-human, multicenter, open-label Phase 1/2 study to evaluate safety, tolerability, and preliminary efficacy of oral LY3537982 as monotherapy and as part of combination therapy in participants with *KRAS* G12C-mutant advanced solid tumor types including, but not limited to, NSCLC, CRC, and pancreatic cancer.

This study includes 4 parts:

- Phase 1a Dose Escalation,
- Phase 1b Dose Expansion,
- Phase 1b Dose Optimization, and
- Phase 2.

<u>Phase 1a—Dose Escalation</u>

In Phase 1a Part A (Dose Escalation), approximately 120 participants will receive LY3537982 monotherapy and combination treatment parts. The primary objective of Part A is to determine the RP2D of LY3537982 monotherapy part ($RP2D_M$) in participants with any *KRAS* G12C-mutant advanced solid tumors.

<u>RP2D Determination</u>

The LY3537982 $RP2D_M$ of [redacted] as monotherapy was determined based on aggregate analysis of the number of observed DLTs, PK, safety, tolerability, and clinical activity. No maximum tolerated dose (MTD) was determined.

Study Schema, Part A (Dose Escalation)

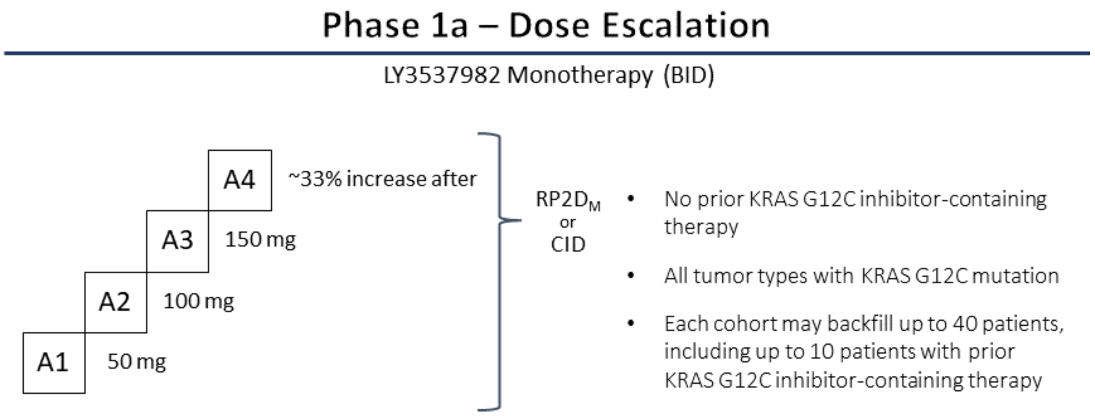

Note: The Combination Initial Dose (CID) may be defined by the Phase 1a SRC prior to $RP2D_M$ determination (see below) for safety lead-in cohorts in Part B and Part C.

<u>Phase 1b—Dose Expansion</u>

In Phase 1b, Part B through Part E (Dose Expansion), approximately 290 participants will be enrolled. The primary objective of dose expansion is to assess the safety and tolerability of LY3537982 when administered alone or in combination with other agents in participants with advanced solid tumors with a *KRAS* G12C mutation. Dose expansion cohort sizes are estimated, may decrease or increase in size, and will not exceed approximately 40 participants.

- LY3537982 monotherapy will be investigated in Cohort B1, Cohort B8, [redacted] and Part D through Part F; and combination therapy will be investigated in all other Phase 1b cohorts.
- Combination therapy agents in dose expansion will include pembrolizumab (Cohort B4 and Cohort B9), and cetuximab (Cohort C2).

- Participants in Cohort B9 will also receive pemetrexed with either carboplatin or cisplatin and will be treatment naïve for advanced or metastatic NSCLC.
- Prior *KRAS* G12C therapy is only allowed in Phase 1b in Cohort B4 and Cohorts E1 [redacted].

Study Schema, Parts B to E (Dose Expansion)

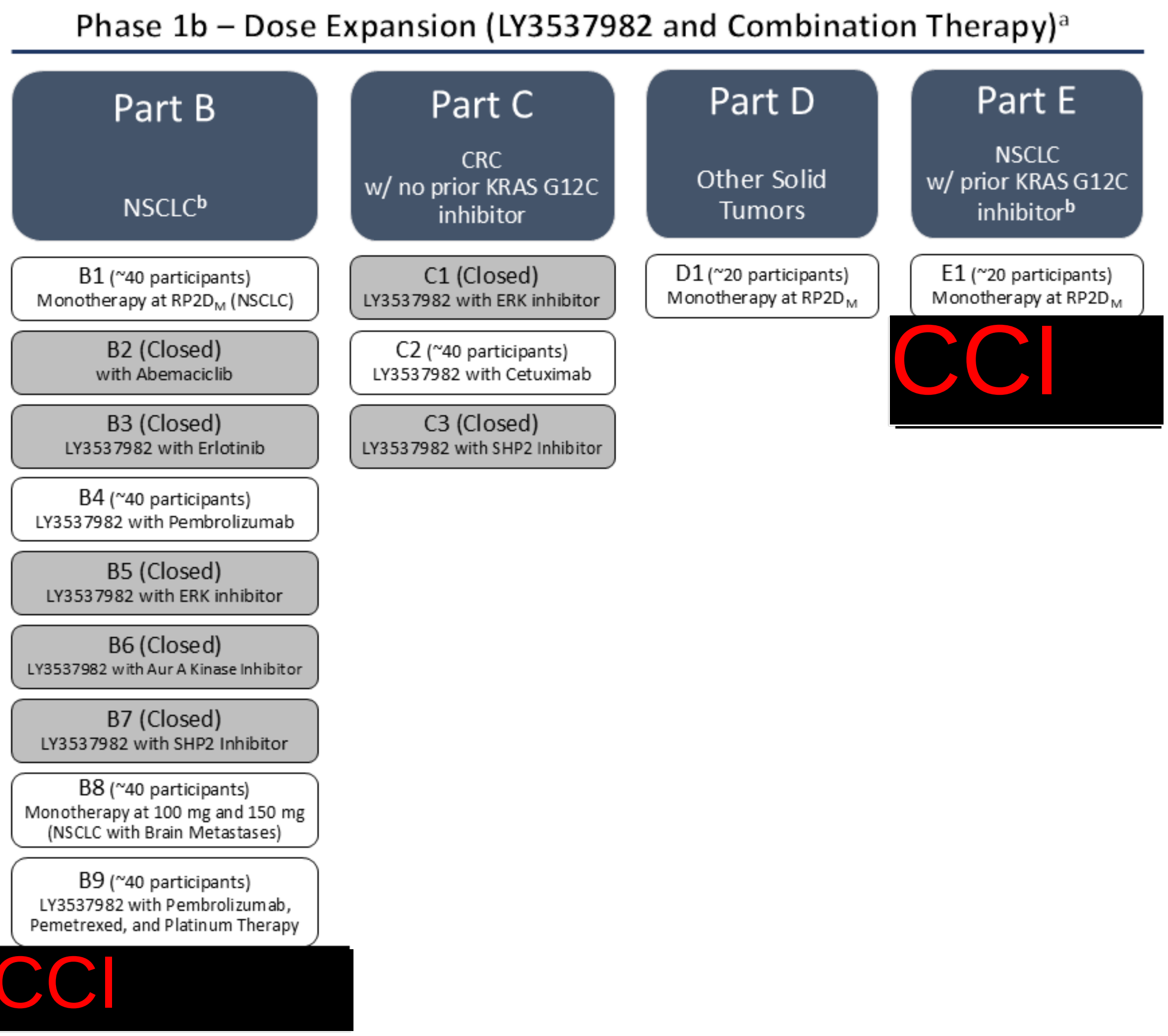

[a] Cohort sizes above (e.g., "~20 participants") indicate estimated enrollment at an established Combination Dose level after the safety lead-in period.

[b] Prior *KRAS* G12C inhibitor-containing therapy is allowed in Cohort B4 and Cohorts E1 [redacted].

Each combination cohort in Part B and Part C will be preceded by a safety lead-in cohort of approximately 6 participants, which will be assigned an initial LY3537982 dose and combination agent dose(s). The initial LY3537982 dose in a combination cohort may be the $RP2D_{M-1}$ or the Combination Initial Dose (CID). The CID is a LY3537982 dose level that may be defined for a particular cohort at any time, if agreed upon by the Sponsor and SRC, if it is at least one DL below the highest level previously deemed safe by the Phase 1b SRC and associated with exposures predicted to be efficacious. The CID may be taken forward as a safety

lead-in dose in Part B and Part C before or after a formal declaration of $RP2D_M$, in order to permit preliminary evaluation of the safety and feasibility of the particular combination therapy.

After the safety lead-in period has established 1 or more Combination Doses, combination cohorts in Part B and Part C may enroll additional participants at any Combination Dose of LY3537982 following a discussion between the Sponsor and Investigators. Up to approximately 40 participants may be enrolled at a Combination Dose level (including any safety lead-in participants treated at that dose), except for Cohort CCI, which may enroll up to approximately 30 participants, to further investigate the tolerability, PK, and biological activity of the LY3537982 combination. For instance, the combination cohorts that utilized the CID as the initial safety lead-in dose of LY3537982 may enroll additional participants at that dose level, if it is cleared as a Combination Dose, or other cleared dose levels (e.g., CID_{+1} or CID_{-1}) or the $RP2D_M$ once determined, if tolerated according to the DLT rules.

Phase 1b—Dose Optimization

In dose optimization (Part G), approximately 40 treatment-naïve participants with *KRAS* G12C-mutant NSCLC will be randomized to receive pembrolizumab 200 mg Q3W with 2 different doses of LY3537982 (50 mg BID and 100 mg BID).

Study Schema, Part G (Dose Optimization)

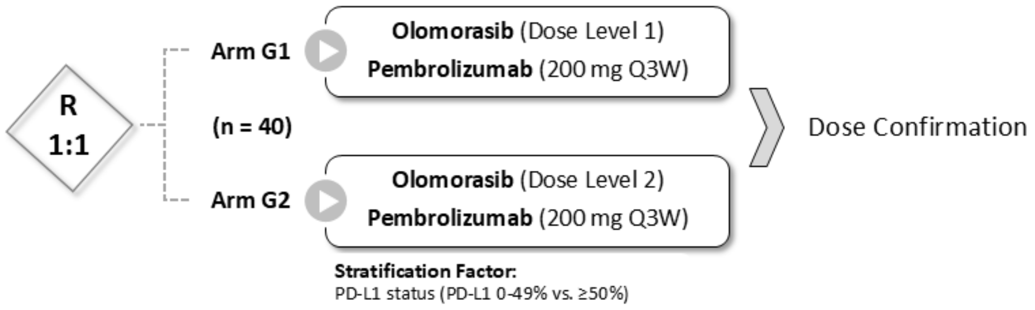

The primary objective of Part G is to optimize the dose of LY3537982 to be administered in combination with pembrolizumab to treatment-naïve participants. Participants will be stratified based on programmed cell death ligand 1 (PD-L1) status (0% to 49% versus ≥ 50%). The LY3537982 dose levels in Arm G1 and Arm G2 (50 mg BID and 100 mg BID) were selected based on observed PK, safety, and preliminary efficacy data from this study. The Phase 1b Safety Review Committee (SRC) along with the study statistician will select the optimal dose and oversee safety aspects in Part G (with a focus on prespecified AEs). At any time, based on the totality of emerging data from participants receiving LY3537982 and pembrolizumab in either Cohort B4 or Part G, the SRC may declare the optimal dose. If the optimal dose is declared prior to full enrollment of Arm G1 and Arm G2, the remaining participants may be enrolled at the selected dose.

In all phases, treatment cycles will be 21 days. The planned duration of LY3537982 treatment is not fixed; participants will remain on study until disease progression or unacceptable toxicity occurs or the participant withdraws consent. Pembrolizumab treatment will conclude after 35 cycles.

KRAS G12C mutations will be identified through standard of care testing as routinely performed at each participating site utilizing material collected prior to participant consent to this protocol.

Part H

In dose optimization Part H, approximately 40 participants with *KRAS* G12C-mutant CRC will be randomized to receive either 100 mg or 150 mg doses of LY3537982 in combination with cetuximab. The Phase 1b SRC along with the study statistician will select the optimal dose and oversee safety aspects in Part H. At any time,

based on the totality of emerging data from participants receiving LY3537982 and cetuximab in either Cohort C2 or Part H, the SRC may declare the optimal dose. If the optimal dose is declared prior to full enrollment of Arm H1 and Arm H2, the remaining participants may be enrolled at the selected dose.

Study Schema, Part H (Dose Optimization)

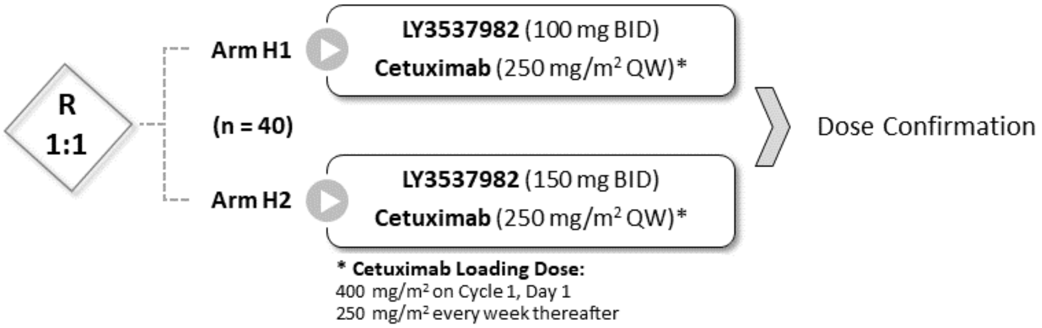

Part F (Phase 2)

In Phase 2, approximately [redacted] pancreatic cancer participants will be enrolled in Part F and will receive LY3537982 monotherapy at the $RP2D_M$. The primary objective of Part F is to assess the antitumor activity of LY3537982 by determining ORR (as assessed by IRC) using RECIST v1.1.

Dose Escalation for Phase 1a

For the Phase 1a dose escalation, participants with *KRAS* G12C mutation positive advanced solid tumors must be appropriate candidates for study treatment. Study treatment is considered appropriate where approved measures are no longer effective or are not considered appropriate or safe in the opinion of the Investigator. The starting dose of LY3537982 monotherapy in capsule form is 50 mg twice daily (BID), and the DLT observation period for both Phase 1a and Phase 1b is 1 cycle (21 days). Dose escalation will occur in Phase 1b during enrollment of safety lead-in cohorts for combination therapy in Part B and Part C.

Dose escalation decisions in Phase 1a and for safety lead-in cohorts in Phase 1b will be based on any DLT events occurring during a 21-day DLT observation period and will be driven by the mTPI-2 method, in which a precalculated decision table will guide the dose recommendations until the MTD is determined. Operating characteristics are given in the protocol. The target toxicity rate of the MTD is set to [redacted]

After 3 to 6 participants have completed the DLT period, the Sponsor and the Investigators will hold a meeting to review safety and available PK results from evaluable participants to ensure it is safe to proceed with the next cohort and to determine the next DL and schedule. The SRC may elect to pursue intermediate, lower, previously tested, or higher DLs based on overall review of safety, PK/pharmacodynamics, efficacy, pill burden, and other factors. Alternative dosing schedules (e.g., once daily [QD] instead of BID dosing) may also be considered based on the emerging PK/pharmacodynamics profile. After completion of the 21-day DLT period, intraparticipant dose escalation to the highest DL deemed safe by the SRC may be permitted following approval by the Sponsor. AEs from nonevaluable participants will also be reviewed throughout the dose escalation process in Phase 1a.

Once a given dose level (DL) is cleared for initial DLT evaluation, and if the PK supports a wider therapeutic index, more participants may be enrolled (i.e., backfilled) to that DL while a higher DL is being evaluated for DLT. If supported by the SRC, backfill slots may be moved between dose levels in a total participant number-neutral manner to allow further exploration of select dose levels.

A total of approximately 10 NSCLC participants with previous *KRAS* G12C inhibitor-containing therapy treatment may be enrolled into each backfill. This limitation on the enrollment of participants with previous

KRAS G12C inhibitor-containing therapy may be lifted, if supported by SRC and Sponsor based on emerging clinical data. If slots are available in both backfill cohorts as well as the current dose escalation cohort simultaneously, enrollment to the dose escalation cohort will be prioritized.

Any AEs reported by participants backfilled into DLs previously deemed safe by the SRC that would otherwise have met DLT criteria will be considered DLT-equivalent toxicities. To ensure that the aggressive DLT and DLT-equivalent toxicity rate does not exceed safe levels, a Bayesian toxicity monitoring will be implemented for DLT and DLT-equivalent toxicities added from backfill participants. DLT evaluation at a higher DL could be halted, and additional participants could be enrolled to a DL that has been cleared for initial DLT evaluation (i.e., stay) or to a lower DL (i.e., de-escalation), depending on the total number of DLTs observed among all DLT-evaluable participants at that DL (initial and backfilled participants).

Dose Escalation for Phase 1b – Safety Lead-In Cohorts

Participants eligible for each part of the expansion will be enrolled to one of the respective cohort options, per the treating physician's choice. Each combination therapy cohort in Part B and Part C will be preceded by a safety lead-in cohort with approximately 6 participants with an LY3537982 dose no higher than the CID or one dose level below $RP2D_M$ ($RP2D_{M-1}$) along with the final planned dose(s) of combination agent(s).

If the number of DLTs after the DLT evaluation period is consistent with an mTPI-2 decision to stay at the current dose or escalate CCI [redacted], continued enrollment may be allowed into the cohort at that dose level. Further, if the LY3537982 $RP2D_{M-1}$ (or CID) and dose level of combination agent(s) is considered tolerable following a review by the Sponsor and the number of DLTs observed is consistent with an mTPI-2 decision to escalate, the Sponsor may open a safety lead-in cohort of additional participants at a higher dose (e.g., $RP2D_M$ or CID_{+1}) of LY3537982 with the same dose level of combination agent. If the initial dose is considered intolerable (mTPI-2 algorithm recommends de-escalation), the LY3537982 dose will be reduced by 1 level ($RP2D_{M-2}$) or more, the combination agent may be reduced by 1 level or more, and 3 more participants will be enrolled and treated at the reduced DLs. Pembrolizumab will not be dose reduced. Further enrollment into the cohort will be paused for all participants to clear the DLT evaluation period. Following a discussion between the Sponsor and the study Investigators, an additional cohort may be enrolled to the reduced DL to further evaluate safety and efficacy.

If the participants in a combination cohort receiving treatment at a de-escalated (reduced) dose level complete the 21-day DLT evaluation period, and if the number of participants with DLTs is consistent with an mTPI-2 decision to re-escalate, the Sponsor may open 1 or more safety lead-in cohorts in a parallel or staggered fashion of additional participants at the next higher or intermediate dose of LY3537982 with the same or reduced dose level(s) of combination agent(s) specified in the protocol. The specific dosing regimen(s) of LY3537982 and combination agent(s) may include delays in administration of LY3537982 or other changes related to toxicity management and must be approved by the SRC after a review of the totality of relevant safety and PK data (if available) in that cohort.

In the event that any cohort is on hold for safety or other reasons, new participants will be enrolled into remaining available cohorts. Safety will be continually monitored with Bayesian toxicity modeling.

Dose-Limiting Toxicity Definitions

A DLT is defined as any of the TEAEs listed below; a TEAE is defined as an AE that starts or worsens on or after the first dose of study drug, as defined by the National Cancer Institute's Common Terminology Criteria for Adverse Events, version 5.0 (NCI CTCAE v5.0), that is deemed clinically significant and occurs during the first 21 days of study drug administration, unless the TEAE can be clearly related to the participant's underlying disease, other medical condition, or concomitant medications.

The DLTs defined below apply to all Phase 1a cohorts and Phase 1b combination therapy cohorts, except for Cohort B4, Cohort B9, and Part G, which will use DLT definitions provided further down in this section. DLT definitions apply to all AEs, with the exception of TEAEs that can clearly be attributed by the Investigator to the combination agent alone. These cases will be carefully reviewed by the SRC/Sponsor to determine whether LY3537982 may have contributed to the observed toxicity, in which case the TEAE would also be considered a DLT.

DLT Definitions for Phase 1a and Phase 1b Combination Therapy Cohorts Except Cohort B4, Cohort B9, and Part G

Hematologic Toxicity
• Any Grade 3 thrombocytopenia associated with clinically significant bleeding and/or requiring platelet transfusion or Grade 4 thrombocytopenia of any duration<br>• Grade ≥ 3 febrile neutropenia and/or neutropenia requiring G-CSF<br>• Grade ≥ 3 anemia requiring blood transfusion, except where the hemoglobin decline represents a clinically insignificant decline from baseline (e.g., ≤ 1.5 g/dL)<br>• Grade 4 neutropenia or leukopenia of > 7 days duration<br>• Other Grade ≥ 4 hematologic toxicity not otherwise specified above
**Nonhematologic Toxicity Nonlaboratory**
• Any Grade ≥ 3 toxicities, except the following:<br>o Grade 3 fatigue, nausea, vomiting, diarrhea, or constipation that lasts for < 72 hours and can be controlled with treatment and does not require hospitalization
**Nonhematologic Toxicities – Laboratory/investigations**<br>• Grade ≥ 3 laboratory abnormalities lasting > 7 days or requiring medical intervention except the following:<br>o Grade ≥3 electrolyte abnormalities that last up to 72 hours, are not clinically significant, and resolve spontaneously or respond to conventional medical interventions<br>• Participants with normal ALT or AST at baseline:<br>o ALT or AST ≥ 5× ULN<br>o ALT or AST ≥ 3× ULN and TBL ≥ 2× ULN in the absence of significant cholestasis (i.e., ALP > 2× ULN) and in the absence of a clear cause of hepatic injury other than the study drug<br>• Participants with elevated ALT or AST at baseline:<br>o ALT or AST ≥ 5× baseline[a] or 8× ULN, whatever is lower<br>o ALT or AST ≥ 2× baseline[a] and TBL ≥ 2× ULN in the absence of significant cholestasis (i.e., ALP > 2× ULN) and in the absence of a clear cause of hepatic injury other than the study drug

Abbreviations: ALP = alkaline phosphatase; ALT = alanine aminotransferase; AST = aspartate aminotransferase; CTCAE = Common Terminology Criteria for Adverse Events; DLT = dose-limiting toxicity; G-CSF = granulocyte colony-stimulating factor; NCI = National Cancer Institute; SRC = safety review committee; TBL = total bilirubin; ULN = upper limits of normal.

[a] Multiples of baseline pertain to the aminotransferase (ALT or AST) which is elevated at baseline.

Any toxicity, regardless of the NCI CTCAE v5.0 grade, resulting in discontinuation, dose reduction, or treatment with < 75% of planned doses of LY3537982 and any combination agent(s), or delay of ≥ 14 days to start the next cycle, will be reviewed by the SRC and will be considered a DLT if the SRC determines the toxicity is not clearly related to the participant's underlying disease, other medical condition, or concomitant medications.

DLT Definitions for Cohort B4, Cohort B9, and Part G

For participants receiving LY3537982 with pembrolizumab in Cohort B4, Cohort B9, and Part G, the occurrence of any of the following toxicities during Cycle 1 will be considered a DLT, if assessed by the Investigator to be possibly, probably, or definitely related to study treatment administration.

1. Grade 4 nonhematologic toxicity (not laboratory).
2. Grade 4 hematologic toxicity lasting ≥ 7 days, except thrombocytopenia:
   - Grade 4 thrombocytopenia of any duration
   - Grade 3 thrombocytopenia associated with clinically significant bleeding
3. Any nonhematologic AE ≥ Grade 3 in severity should be considered a DLT, with the following

exceptions: Grade 3 fatigue lasting ≤ 3 days; Grade 3 diarrhea, nausea, or vomiting without use of antiemetics or antidiarrheals per standard of care, and Grade 3 rash without use of corticosteroids or anti-inflammatory agents per standard of care.

4. Any Grade 3 or Grade 4 nonhematologic laboratory value if:
   - Clinically significant medical intervention is required to treat the participant, or
   - The abnormality leads to hospitalization, or
   - The abnormality persists for > 1 week, or
   - The abnormality results in a drug-induced liver injury
   - Exceptions: Clinically nonsignificant, treatable, or reversible laboratory abnormalities including liver function tests (LFTs), uric acid, etc.
5. Febrile neutropenia Grade 3 or Grade 4:
   - Grade 3 is defined as ANC < 1000/mm^3 with a single temperature of > 38.3°C (101°F) or a sustained temperature of ≥ 38°C (100.4°F) for more than 1 hour
   - Grade 4 is defined as absolute neutrophil count (ANC) < 1000/mm^3 with a single temperature of > 38.3°C (101°F) or a sustained temperature of ≥ 38°C (100.4°F) for more than 1 hour, with life-threatening consequences and urgent intervention indicated.
6. Prolonged delay (> 2 weeks) in initiating Cycle 2 due to treatment-related toxicity.
7. Any treatment-related toxicity that causes the participant to discontinue treatment during Cycle 1.
8. Missing > 25% of LY3537982 doses as a result of drug-related AE(s) during the first cycle.
9. Grade 5 toxicity.

Intervention Groups and Duration

The doses of LY3537982 monotherapy in Phase 1a will proceed through planned DLs as outlined in the Phase 1a Study Schema above (50 to 100 to 150 mg, then 33% increases thereafter).

In Phase 1b, all monotherapy cohorts except [redacted] will begin at the $RP2D_M$ as shown in the table below. All combination therapy cohorts will begin as safety lead-in cohorts with LY3537982 doses one level below $RP2D_M$ ($RP2D_{M-1}$), or at the CID, with combination therapy dose(s) at the levels shown below.

Phase 1b Dose Expansion and Dose Optimization Cohorts

Study Part	Dose Expansion Cohort	Study Drug(s) (Route of Administration)	Proposed Initial Doses[a] (Frequency)
**Part B:** *KRAS* G12C-mutant advanced NSCLC	B1[b]	LY3537982 (PO)	$RP2D_M$ (BID)
	B2 (closed)	LY3537982 (PO)	$RP2D_{M-1}$ or CID (BID)
		LY2835219 (abemaciclib) (PO)	150 mg (BID)
	B3 (closed)	LY3537982 (PO)	Not applicable
		Erlotinib (PO)	Not applicable
	B4	LY3537982 (PO)	$RP2D_{M-1}$ or CID (BID)
		Pembrolizumab (IV)	200 mg (Q3W)
	B5 (closed)	LY3537982 (PO)	Not applicable
		LY3214996 (ERK inhibitor) (PO)	Not applicable
	B6	LY3537982 (PO)	Not applicable

	(closed)	LY3295668 (Aurora A kinase inhibitor) (PO)	Not applicable
	B7 (closed)	LY3537982 (PO)	Not applicable
		TNO155 (SHP2 inhibitor) (PO)	Not applicable
	B8[b]	LY3537982 (PO)	100 mg (BID), $RP2D_{M}$
	B9	LY3537982 (PO)	$RP2D_{M-1}$ or CID (BID)
		Pembrolizumab (IV)<br><br>Pemetrexed (IV)<br><br>and<br><br>Cisplatin (IV)<br>OR<br>Carboplatin (IV)	200 mg (Q3W)<br><br>500 mg/m^2 (Q3W)<br><br>and<br><br>75 mg/m^2 (Q3W)<br>OR<br>AUC 5 mg/ml/min (Q3W)<br>(max dose = 750 mg)
	CCI	[redacted]	[redacted]
**Part C:**<br>*KRAS* G12C-mutant advanced CRC	C1 (closed)	LY3537982 (PO)	Not applicable
		LY3214996 (ERK inhibitor) (PO)	Not applicable
	C2	LY3537982 (PO)	$RP2D_{M-1}$ or CID (BID)
		Cetuximab (IV)	400 mg/m^2 loading dose C1D1;<br>250 mg/m^2 every week thereafter (QW)
	C3 (closed)	LY3537982 (PO)	Not applicable
		TNO155 (SHP2 inhibitor) (PO)	Not applicable
**Part D:**<br>*KRAS* G12C-mutant advanced solid tumors (except NSCLC, CRC, and pancreatic cancer)	D1	LY3537982 (PO)	$RP2D_{M}$ (BID)
**Part E:**<br>*KRAS* G12C-mutant advanced NSCLC who have previously been treated with a *KRAS* G12C inhibitor	E1[d]	LY3537982 (PO)	$RP2D_{M}$ (BID)
	CCI	[redacted]	[redacted]
**Part G:**<br>Previously untreated *KRAS* G12C-mutant advanced NSCLC	Arm G1	LY3537982 (PO)	50 mg BID
		Pembrolizumab (IV)	200 mg (Q3W)
	Arm G2	LY3537982 (PO)	100 mg BID
		Pembrolizumab (IV)	200 mg (Q3W)
**Part H:**<br>*KRAS* G12C-mutant advanced CRC	Arm H1	LY3537982 (PO)	100 mg BID
		Cetuximab (IV)	400 mg/m^2 loading dose C1D1;<br>250 mg/m^2 every week thereafter (QW)
	Arm H2	LY3537982 (PO)	150 mg BID
		Cetuximab (IV)	400 mg/m^2 loading dose C1D1;<br>250 mg/m^2 every week thereafter (QW)

Abbreviations: BID = twice daily; CRC = colorectal cancer; ERK = extracellular signal regulated kinase; IV = intravenous; NSCLC = non-small cell lung cancer; PO = orally; QD = once daily; QW = every week; Q3W = every 3 weeks; RP2D$_{M}$ = monotherapy recommended Phase 2 dose; RP2D$_{M-1}$ = 1 dose level below RP2D$_{M}$.

[a] For combination cohorts in Part B and Part C, the LY3537982 doses shown are those allowed in the first safety lead-in cohort. The dose taken forward in each cohort after the safety lead-in period may differ. Alternative dosing schedules (i.e., QD) may be considered by the Phase 1b SRC for all agents except pembrolizumab, pemetrexed, carboplatin, and cisplatin, based upon the totality of safety, PK/pharmacodynamics, and efficacy data.

[b] Cohort B1 will enroll participants with *KRAS* G12C-mutant advanced NSCLC, including those with treated, stable brain metastases; Cohort B8 will enroll participants with *KRAS* G12C-mutant advanced NSCLC with untreated, active brain metastases.

[c] Participants in Cohort B8, [redacted] may dose escalate with Sponsor approval under certain conditions; see Protocol Section 7.11.

[d] Will only be opened if activity observed in Phase 1a.

Phase 2 Cohort

Study Part	Dose Expansion Cohort	Study Drug(s) (Route of Administration)	Proposed Initial Doses[a] (Frequency)
**Part F:** **KRAS G12C-mutant pancreatic cancer**	F1	LY3537982 (PO)	RP2D$_{M}$ (BID)

Benefit/Risk:

LY3537982 is a potent, mutant-selective small molecule covalent binder of KRAS G12C and has demonstrated significant activity in preclinical models with KRAS G12C mutation. LY3537982 administration to participants with KRAS G12C mutation is expected to provide clinical benefit.

Since LY3537982 is in an early stage of drug development, it is possible that unforeseen, unknown, or unanticipated drug reactions and toxicities may occur. However, the study protocol is designed to mitigate risks to participants through a detailed plan for cautious dose escalation, careful safety monitoring, systematic review of AEs and SAEs, PK evaluation, and active pharmacovigilance review to assess safety signals or trends.

Given the potential therapeutic benefit anticipated for LY3537982 administered as monotherapy and in combination with pembrolizumab, cetuximab, or pembrolizumab plus pemetrexed and carboplatin or cisplatin, as well as the known risk profile of these combination therapies and the benefit/risk assessment of LY3537982 monotherapy, the benefit/risk profiles of the monotherapy and the combination therapies proposed in the current study are considered acceptable.

More detailed information about the known and expected benefits and risks of cetuximab, pemetrexed, cisplatin, and carboplatin may be found in the Patient Information Leaflet, Package Insert, and Summary of Product Characteristics.

More detailed information about the known and expected benefits and risks of pembrolizumab may be found in Protocol Section 9.9, Appendix 9.

Study Population

The study population will include adult participants with KRAS G12C-mutant advanced or metastatic solid tumors including, but not limited to, pancreatic cancer, CRC, and NSCLC.

Inclusion Criteria

Individuals are eligible to be included in the study only if all of the following criteria apply:

1. Individuals must be able to provide consent consistent with local regulations and be ≥ 18 years of age at the time of signing the informed consent.

2. Individuals must have measurable disease per RECIST v1.1.

3. Individuals must have disease with evidence of *KRAS* G12C mutation in tumor tissue or circulating tumor DNA as determined by molecular testing performed at a Clinical Laboratory Improvement Amendments (CLIA), International Organization for Standardization/International Electrotechnical Commission (ISO/IEC), College of American Pathologists (CAP), or other similar certified laboratory as per local guidelines including, but not limited to, In Vitro Diagnostic Medical Devices Regulation (European Union) 2017/746 (IVDR) compliance as applicable. In cohorts where individuals with NSCLC who have progressed on a prior *KRAS* G12C inhibitor are allowed to enroll, the individuals must have *KRAS* G12C mutation confirmed in a blood or tumor tissue sample collected within approximately 3 months preceding discontinuation of prior *KRAS* G12C inhibitor due to disease progression or at any time thereafter.

4. Individuals must have a histological or a cytologically proven diagnosis of locally advanced, unresectable, and/or metastatic cancer and meet cohort-specific criteria:

   a) Phase 1a Dose Escalation:

   - Individuals must be appropriate candidates for study treatment. Study treatment is considered appropriate where approved measures are no longer effective or are not considered appropriate or safe in the opinion of the Investigator. If the available standard therapy is not considered appropriate or safe in the opinion of the Investigator, the rationale for ineligibility shall be provided and documented in the CRF. Furthermore, in the opinion of the Investigator, if the individual is intolerant to standard therapy, drug information, toxicity, and grade must be documented in the case report form (CRF).

   b) Phase 1b Dose Expansion Part B (NSCLC):

   - Individuals must not have a known targetable oncogenic driver mutation or alteration in genes such as *EGFR*, *ALK*, *BRAF* (V600E), *MET* (exon 14), *ROS1*, *RET,* or *NTRK1/2/3*. Does not apply to individuals in Cohort B4.

     Cohort B9 Only Specific Criteria

     - Histologically or cytologically confirmed Stage IIIB-IIIC or Stage IV NSCLC that is previously untreated in the advanced/metastatic setting and not suitable for curative intent radical surgery or radiation therapy. Staging will be according to the American Joint Committee on Cancer (AJCC) Staging System (8th ed). The histology of the tumor must be predominantly nonsquamous. Squamous cell and/or mixed small cell/non-small cell histology is not permitted.
     - Individuals may have received up to one 21-day cycle of any or all components of the KEYNOTE-189 treatment regimen (i.e., pembrolizumab, pemetrexed, carboplatin/cisplatin) at or below the dose levels defined for Cohort B9 in Table 7, Section 5.1 of the protocol; such therapy must have been initiated within 21 days (+14 days) prior to enrollment. Start of study treatment may be delayed for a maximum of 42 days from day 1 of the prior cycle of therapy to allow sufficient time for recovery from treatment-related toxicity.
       - o If the SRC has approved delay of LY3537982 administration to the maintenance setting in Cohort B9 as per Section 3.1.6.1.1, these individuals may have received up to 6 cycles of any combination of the KEYNOTE-189 treatment regimen (i.e., pembrolizumab, pemetrexed, carboplatin/cisplatin) prior to enrollment.

   - Individuals should only be enrolled in Cohort B9 if, in the Investigator's judgment, the individual is an appropriate candidate to initiate their planned treatment regimen at the dose levels specified in each combination agent's Package Insert or Summary of Product Characteristics. Individuals entering Cohort B9 in the maintenance setting need not be appropriate to initiate treatment with carboplatin or cisplatin.

     Remaining Part B Cohorts

     - Individuals must be appropriate candidates for study treatment, after progressing on, being intolerant to, or ineligible for immunotherapy and platinum-based therapy. Study treatment is considered appropriate where approved measures are no longer effective or are not considered appropriate or safe in the opinion of the Investigator. If the available standard therapy is not considered appropriate or safe in the opinion of the Investigator, the rationale for ineligibility

shall be provided and documented in the CRF. Furthermore, in the opinion of the Investigator, if the individual is intolerant to standard therapy, drug information, toxicity, and grade must be documented in the CRF. Progressing on, being intolerant to, or ineligible for immunotherapy is NOT applicable to individuals in Cohort B4.

Additional Cohort B8 Only Specific Criteria

- Individuals must have at least 1 untreated, active brain metastasis, defined as a new and/or growing lesion at least 5 mm in diameter, without any subsequent local treatment before the start of study treatment, and without associated neurological symptoms requiring urgent intervention with locoregional therapy. Individuals with leptomeningeal disease are not eligible. Prophylactic anticonvulsants are permitted, provided the individual is on a stable dose for ≥ 14 days prior to Cycle 1 Day 1 (C1D1), except those taking enzyme-inducing anti-epileptic drugs must have discontinued such therapy with a washout period equivalent to 5 half-lives of the drug.

c) Phase 1b Dose Expansion Part C (CRC):

- Individuals must be appropriate candidates for study treatment and must have received at least 1 prior oxaliplatin- or irinotecan-containing regimen for advanced or metastatic CRC. Study treatment is considered appropriate where approved measures are no longer effective or are not considered appropriate or safe in the opinion of the Investigator. If the available standard therapy is not considered appropriate or safe in the opinion of the Investigator, the rationale for ineligibility shall be provided and documented in the CRF. Furthermore, in the opinion of the Investigator, if the individual is intolerant to standard therapy, drug information, toxicity, and grade must be documented in the CRF.
- For Cohort C2, individuals should only be enrolled if, in the Investigator's judgment, the individual is appropriate to initiate cetuximab at the standard dose in the Package Insert.

d) Phase 1b Dose Expansion Part D (Solid Tumors Except NSCLC, CRC, and Pancreatic Cancer):

- Individuals must have histological or cytologically proven recurrent/metastatic, unresectable solid tumors except NSCLC, CRC, and pancreatic cancer, and with *KRAS* G12C mutation.
- Individuals must be appropriate candidates for study treatment. Study treatment is considered appropriate where approved measures are no longer effective or are not considered appropriate or safe in the opinion of the Investigator. If the available standard therapy is not considered appropriate or safe in the opinion of the Investigator, the rationale for ineligibility shall be provided and documented in the CRF. Furthermore, in the opinion of the Investigator, if the individual is intolerant to standard therapy, drug information, toxicity, and grade must be documented in the CRF.

e) Phase 1b Dose Expansion Part E (NSCLC):

- Individuals must have been previously treated with a KRAS G12C inhibitor.
- Individuals must be appropriate candidates for study treatment. Study treatment is considered appropriate where approved measures are no longer effective or are not considered appropriate or safe in the opinion of the Investigator. If the available standard therapy is not considered appropriate or safe in the opinion of the Investigator, the rationale for ineligibility shall be provided and documented in the CRF. Furthermore, in the opinion of the Investigator, if the individual is intolerant to standard therapy, drug information, toxicity, and grade must be documented in the CRF.

f) Phase 2 (Part F) (Pancreatic Cancer)

- Individuals must have histological or cytologically proven recurrent/metastatic, unresectable pancreatic cancer with *KRAS* G12C mutation.
- Individuals must be appropriate candidates for study treatment. Study treatment is considered appropriate where approved measures are no longer effective or are not considered appropriate or

safe in the opinion of the Investigator. If the available standard therapy is not considered appropriate or safe in the opinion of the Investigator, the rationale for ineligibility shall be provided and documented in the CRF. Furthermore, in the opinion of the Investigator, if the individual is intolerant to standard therapy, drug information, toxicity, and grade must be documented in the CRF.

g) Phase 1b Dose Optimization Part G (NSCLC)

- Individuals must not have additional validated oncogenic drivers in NSCLC, if known, e.g., activating alterations in genes such as *EGFR*, *ALK*, *BRAF (V600E)*, *MET* (exon 14), *ROS1*, *RET*, or *NTRK1/2/3*
- Individuals must have histologically or cytologically confirmed Stage IIIB-IIIC or Stage IV NSCLC, previously untreated in the advanced/metastatic setting and not suitable for curative intent radical surgery or radiation therapy. Staging will be according to the AJCC Staging System (8th ed).
- Individuals may have received up to one 21-day cycle of pembrolizumab 200 mg Q3W; such therapy must have been initiated within 21 days (+14 days) prior to enrollment.

h) Phase 1b Dose Optimization Part H (CRC):

- Individuals must be appropriate candidates for study treatment and must have received at least 1 prior oxaliplatin- or irinotecan-containing regimen for advanced or metastatic CRC. Study treatment is considered appropriate where approved measures are no longer effective or are not considered appropriate or safe in the opinion of the Investigator. If the available standard therapy is not considered appropriate or safe in the opinion of the Investigator, the rationale for ineligibility shall be provided and documented in the CRF. Furthermore, in the opinion of the Investigator, if the individual is intolerant to standard therapy, drug information, toxicity, and grade must be documented in the CRF.

- For Part H, individuals should only be enrolled if, in the Investigator’s judgment, the individual is appropriate to initiate cetuximab at the standard dose in the Package Insert.

5. Individuals must have an Eastern Cooperative Oncology Group (ECOG) performance status of 0 or 1.

6. Individuals must have adequate organ function, as defined in the table below:

System	Laboratory Value
**Hematologic**	
ANC	≥ 1.5 × 10^9/L
Platelets	≥ 100 × 10^9/L
Hemoglobin[a]	≥ 9 g/dL
**Hepatic**	
TBL	≤ 1.5 × ULN,<br>Individuals with Gilbert's syndrome with a TBL ≤ 3.0 × ULN and direct bilirubin within normal limits are permitted.
ALT and AST	≤ 2.5 × ULN **OR**<br>≤ 5 × ULN if the liver has tumor involvement.
**Renal**	
Serum creatinine **OR**<br>CrCl	< 1.5 × ULN **OR**<br>Calculated creatinine clearance of ≥ 50 mL/minute using a validated method such as estimation via Cockcroft/Gault[b], CKD-EPI, or MDRD equations; or direct measurement of creatinine clearance in urine.

Abbreviations: ALT = alanine aminotransferase; AST = aspartate aminotransferase; ANC = absolute neutrophil count; G-CSF = granulocyte-colony stimulating factor; TBL = total bilirubin; ULN = upper limit of normal.

[a] Transfusions to increase an individual's hemoglobin level or initiation of erythropoietin or G-CSF therapy to meet enrollment criteria are not allowed in the 14 days prior to enrollment. If an individual receives transfusions, erythropoietin, or G-CSF therapy ≥ 14 days prior to enrollment, the hematologic criteria listed above must be met following the 14-day window and prior to enrollment. For Cohort B4, Cohort B9, and Part G, individuals can be on a stable dose of erythropoietin (≥ approximately 3 months).

[b] See Section 9.6.

7. Individuals must have discontinued all previous treatments for cancer with improvement of any prior therapy-related AEs, with the exception of alopecia and Grade 2 neuropathy, and of all clinically significant toxic effects of prior locoregional therapy, surgery, radiotherapy, or systemic anticancer therapy to Grade ≤ 1 or prior baseline. Individuals with ongoing endocrinopathies due to prior treatment that have not resolved but are on appropriate replacement therapy (e.g., thyroid, adrenal or pituitary, or pancreatic) are permitted to enroll.

   - For Cohort B9 only: Individuals with therapy-related toxicities that have developed and are related to any or all components of the KEYNOTE-189 treatment regimen (i.e., pembrolizumab, pemetrexed, carboplatin/cisplatin) (with the exception of the immune-related toxicities outlined in Exclusion Criterion #24) may enroll, if the Investigator determines these toxicities are not required to be resolved at the time of study entry and the individual is still an appropriate candidate for study treatment.

8. Individuals must have discontinued from previous treatments, as shown in the table below:

Previous Treatment	Length of Time Prior to Start of Study Drug(s)
Cytotoxic therapies or targeted agents that are small molecule inhibitors	≥ 14 days or ≥ 5 half-lives, whichever is shorter
Biologic agents that are large molecules including immunotherapy	≥ 21 days<br>For Cohort B4, ≥ 28 days
Radiotherapy<br>Limited-field radiotherapy with palliative intent<br>Other radiotherapy	≥ 7 days<br>≥ 21 days

Major surgery	≥ 28 days

Note: The systemic therapy washouts above do not apply to those individuals enrolling into Cohort B9 and Part G who have received prior cycle of therapy, as allowed in Inclusion Criterion 4.

9. Individuals must be able to swallow capsule/tablet.
10. Individuals must agree and adhere to contraceptive use by men or women that is consistent with local regulations regarding the methods of contraception for those participating in clinical studies. See protocol Section 9.3, Appendix 3 for guidance on contraceptive use and collection of pregnancy information.
11. Individuals who are WOCBP must have a negative serum or urine pregnancy test documented within 7 days (72 hours for Cohort B4, Cohort B9, and Part G) prior to enrollment.
12. Individuals must have an estimated life expectancy of ≥ 12 weeks.
13. Individuals must be reliable and willing to make themselves available for the duration of the study and be willing to follow study procedures.
14. Individuals must be capable of giving signed informed consent as described in protocol Section 9.1.2, Appendix 1, which includes compliance with the requirements and restrictions listed in the ICF and in this protocol.

Exclusion Criteria

Individuals are excluded from the study if **any** of the following criteria apply:

15. Individual has disease suitable for local therapy administered with curative intent, except as allowed for specific cohorts in Inclusion Criterion 4.
16. Individual has an active fungal, bacterial, and/or active untreated viral infection, including human immunodeficiency virus (HIV) or viral (A, B, or C) hepatitis (screening is not required unless mandated by local health authority).

    Note: For Cohort B4, Cohort B9, and Part G only:

    a) HIV-infected individuals must be on antiretroviral therapy (ART) and have a well-controlled HIV infection/disease defined as:

    - Individuals on ART must have a CD4+ T-cell count > 350 cells/mm^3 at time of Screening
    - Individuals on ART must have achieved and maintained virologic suppression defined as confirmed HIV RNA level below 50 copies/mL or the lower limit of qualification (below the limit of detection) using the locally available assay at the time of Screening and for at least 12 weeks prior to Screening
    - Individuals on ART must have been on a stable regimen, without changes in drugs or dose modification, for at least 4 weeks prior to study entry (Day 1).
    - HIV-infected Individuals with a history of Kaposi sarcoma and/or Multicentric Castleman Disease are excluded.

    b) Individuals who are Hepatitis B surface antigen (HbsAg) positive are eligible if they have received HBV antiviral therapy for at least 4 weeks and have undetectable hepatitis B virus (HBV) viral load prior to enrollment.

    Note: Individuals should remain on antiviral therapy throughout study intervention and follow local guidelines for HBV antiviral therapy post completion of study intervention.

    Hepatitis B screening tests are not required unless:

- Known history of HBV infection
- As mandated by local health authority

c) Individuals with history of hepatitis C virus (HCV) infection are eligible if HCV viral load is undetectable at Screening.

Note: Individuals must have completed curative antiviral therapy at least 4 weeks prior to enrollment.

Hepatitis C screening tests are not required unless:

- Known history of HCV infection
- As mandated by local health authority

17. Individual has a serious pre-existing medical condition(s) that, in the judgment of the Investigator, would preclude participation in this study, including interstitial lung disease (ILD) or severe dyspnea at rest and uncontrolled disease-related pericardial effusion or pleural effusion.

18. Individual has clinically significant, active cardiovascular disease, unstable angina, or history of myocardial infarction within 6 months prior to planned start of LY3537982, or QT interval corrected for heart rate of ≥ 470 msec on screening ECG as calculated using Fridericia's formula (QTcF). If QTcF > 470 msec on more than 1 ECG is obtained during the screening, repeat 2 additional times and use the average to determine eligibility. Note that individuals with implanted pacemakers may enter study without meeting QTc criteria due to nonevaluable measurement. Correction of suspected drug-induced QTcF prolongation or existing bundle branch block prolonged QTcF can be attempted at the Investigator's discretion and in suspected drug-induced prolongation only if clinically safe to do so with either discontinuation of the offending drug or switching to another drug not known to be associated with QTcF prolongation.

19. Individual has a second active primary malignancy or have been diagnosed and/or treated for an additional malignancy within 3 years prior to enrollment with the exception of curatively-treated basal cell carcinoma of the skin, nonmetastatic prostate cancer treated with observation only, squamous cell carcinoma of the skin, and/or curatively resected in situ cervical and/or breast cancers. Exceptions may be permitted following discussion with study Investigator and Sponsor.

20. For all individuals except those in Cohort B8: Have untreated active central nervous system (CNS) metastases and/or leptomeningeal disease. Individuals with previously treated CNS metastases may participate provided they have

    - completed prior CNS-directed therapy (including radiation and/or surgery) ≥ 28 days prior to the first dose of study intervention, and
    - symptomatically and radiologically stable disease (i.e., without evidence of progression for ≥ 28 days by repeat imaging). For Cohort B4, Cohort B9, and Part G, patients must be clinically stable without requirement of steroid treatment within 14 days prior to C1D1. Prophylactic anticonvulsants are permitted, provided the individual is on a stable dose for ≥ 14 days prior to C1D1, except individuals taking enzyme-inducing anti-epileptic drugs must have discontinued such therapy with a washout period equivalent to 5 half-lives of the drug.

Prior/Concomitant Therapy

21. Individual has received prior treatment with any *KRAS* G12C small molecule inhibitor, except in the following scenarios where such prior therapy is allowed:

    a) Phase 1a dose escalation backfill cohort (NSCLC only)

    b) Cohorts E1 [redacted]

    c) Cohort B4

22. Exclusion criterion #22 has been removed.

23. The following individuals will be excluded from Cohort B4, Cohort B9, and Part G:

    a) Individual has experienced a Grade 3 immune-related toxicity or any immune-related toxicity that led to permanent discontinuation of prior anti-PD-1, anti-PD-L1, or other immunotherapy

    b) Individual has experienced a ≤ Grade 3 immune-related AE (irAE) that has not recovered to Grade 1 after use of corticosteroids that occurred during prior immunotherapy (exceptions: endocrine disorders where individuals with prior endocrine AEs are permitted to enroll if they are considered clinically stable and maintained on appropriate replacement therapy)

    c) Individual has any grade:

        i. Ocular irAE,

        ii. Serious neurologic irAE (e.g., Guillain-Barre syndrome, myasthenia gravis, encephalitis)

        iii. Serious cardiovascular irAE (e.g., myocarditis)

    d) Individual has required immunosuppressive agents other than corticosteroids for the management of irAEs or currently requires maintenance doses of ≥ 10 mg prednisone/prednisolone (or equivalent) per day for irAEs

24. The following individuals will be excluded from Cohort B4, Cohort B9, and Part G:

    a) Individual has an active autoimmune disease that has required systemic anti-autoimmune treatment in the past 2 years (i.e., with use of disease modifying agents, corticosteroids, or immunosuppressive drugs). Replacement therapy (e.g., thyroxine, insulin, or physiologic corticosteroid replacement therapy for adrenal or pituitary insufficiency) is not considered a form of systemic treatment and is allowed.

    b) Exclusion Criterion 24(b) has been removed.

    c) Individual has received a live vaccine within 30 days prior to the first dose of study drug. Examples of live vaccines include, but are not limited to, the following: measles, mumps, rubella, varicella/zoster (chicken pox), yellow fever, rabies, Bacillus Calmette–Guérin, and typhoid vaccine. Seasonal influenza vaccines for injection are generally killed virus vaccines and are allowed; however, intranasal influenza vaccines are live attenuated vaccines and therefore, are not allowed.
    Note: Any licensed COVID-19 vaccine (including for Emergency Use) in a particular country is allowed as long as they are messenger ribonucleic acid (mRNA) vaccines, adenoviral vaccines, or inactivated vaccines.

    d) Individual has had an allogeneic tissue/solid organ transplant.

    e) Individual has severe hypersensitivity (≥ Grade 3) to pembrolizumab and/or any of its excipients.

    f) Individual has received radiation therapy to the lung that is > 30 Gy within 6 months of the first dose of trial treatment

    Note: For Cohort B9 and Part G only:

    g) Individual received prior systemic therapy (chemotherapy, immunotherapy, or biological therapy) for advanced or metastatic disease, except as allowed in Inclusion Criterion #4. Individuals who received adjuvant or neoadjuvant therapy are eligible if the last dose of the systemic treatment was completed at least 6 months prior to enrollment.

        - For individuals who received immunotherapy in either the neoadjuvant or adjuvant settings, relapse/recurrence of metastatic disease should have occurred at least 6 months after last dose.

    Note: For Cohort B9 only:

h) Individual is unable to interrupt nonsteroidal anti-inflammatory drugs (NSAIDs) 2 days before (5 days for long-acting NSAIDs), the day of, and 2 days following administration of pemetrexed.

i) Individual is unable or unwilling to take folic acid, dexamethasone, or vitamin B12 supplementation.

j) Individual has a known hypersensitivity to any of the excipients of carboplatin, cisplatin, or pemetrexed.

k) Individual has creatinine clearance < 45 mL/min (measured or calculated) at C1D1 or within 48 hours prior to C1D1.

l) Individual is known to be intolerant to any component of their planned treatment regimen at the dose levels specified in each combination agent's Package Insert or Summary of Product Characteristics, as evidenced by Grade 4 hematologic toxicity, any toxicity requiring transfusion support or G-CSF, or other prohibitive toxicity.

Other Exclusions

25. Individual is pregnant, breastfeeding, or expecting to conceive or father children within the projected duration of the trial, starting with the Screening visit through 35 days after the last dose of study medication.

26. Individual has a known allergic reaction against any of the components of the study treatments.

27. Exclusion criterion #27 has been removed.

28. For Cohort B4, Cohort B9, and Part G only, individuals with a history of (noninfectious) pneumonitis/radiation pneumonitis/interstitial lung disease that required steroids or has current pneumonitis/interstitial lung disease.

29. Individual has a prior enrollment in another cohort in this study.

Investigational Product

LY3537982 will be provided as an oral solid dosage form and will be provided to the sites for distribution to the participant for outpatient administration at the assigned DL. Dosing will be fixed (i.e., not weight-based or body surface area [BSA]-based). See Protocol Section 5.0 for details for other combination agents.

Study Assessments

Safety observations include physical examination, body weight, ECOG score, clinical AEs, laboratory variables (hematology, serum or plasma chemistries, LFTs, coagulation, and urinalysis), ECGs and vital signs.

Baseline disease assessment with radiographic tumor measurements using computed tomography (CT) or magnetic resonance imaging (MRI) of the chest, abdomen, pelvis, or any other areas with suspected disease involvement must occur within 28 days of Cycle 1 Day 1. During Phase 1a dose escalation and Phase 1b dose expansion, brain imaging is required at baseline for all participants with NSCLC and as clinically indicated for other tumor types (MRI preferred, CT with contrast is acceptable if MRI contraindicated).

Efficacy assessments include tumor evaluation every 6 weeks after treatment initiation through Week 24, and then approximately every 9 weeks thereafter, consistent with disease-defined criteria. Confirmatory response evaluations may be conducted 4 weeks (± 7 days) after the first tumor evaluation that shows a partial response (PR) or better, if consistent with local institution and regulatory authority requirements.

An end of treatment (EOT) visit within 7 days of the last dose of LY3537982 or the decision to terminate treatment is required, where clinically appropriate.

In addition, a SFU visit 28 days after the last dose of study drug (± 7 days) is required to determine the status of any unresolved AEs.

Long-term follow-up (LTFU) visits will occur approximately every 3 months for up to 2 years after last dose of study drug to assess survival and confirm the resolution of any SAEs, progressive disease (PD) not occurring on study, and subsequent anticancer therapy. LTFU may be conducted by phone.

Participants who discontinue study treatment for reasons other than PD, withdrawal of consent, or initiation of a new anticancer therapy should continue undergoing disease assessment (as specified above) until PD, withdrawal of consent, or initiation of a new anticancer therapy.

PK timepoints are specified in the protocol. Additional PK timepoints may also be assessed when considered necessary by the Investigator or the Sponsor to understand the relationship between LY3537982 exposure and safety or efficacy.

Tumor tissue will be obtained for participants. Participants who do not have adequate archival tumor tissue available may undergo a fresh tumor biopsy prior to treatment if it is considered safe to perform. If archival tissue (preferably from the most recent biopsy) is not available and a fresh tumor biopsy cannot be performed, the participant will likely still be eligible to enroll upon review and approval by the Sponsor. The samples may be used for confirmation of *KRAS* G12C mutation and detection of additional genomic variants. Guidance is provided in the Laboratory Manual and Section 7.10 of the protocol. Blood, plasma, and tumor tissue samples for biomarker research will be collected at the times specified in the Schedule of Assessments in the protocol Section 7.1). Samples will be used for research on the drug target, disease process, variable response to study treatment, pathways associated with study treatment, mechanism of action of study treatment, and/or to develop research methods or validate diagnostic tools or assays related to cancer.

Study Endpoints:

**Phase 1a/1b**	**Primary Endpoints Dose Escalation**	○ DLTs<br>○ AEs and SAEs, changes in hematology and blood chemistry values, assessments of physical examinations, vital signs, and ECGs
	**Primary Endpoints Dose Expansion**	○ DLT-equivalent toxicities, AEs, and SAEs, changes in hematology and blood chemistry values, assessments of physical examinations, vital signs, and ECGs
	**Primary Endpoints Dose Optimization**	○ Including, but not limited to, TEAEs, SAEs, deaths, and clinical laboratory abnormalities
	**Secondary Endpoints**	○ ORR, BOR, DOR, TTR, DCR, PFS, OS<br>○ Intracranial ORR and DOR (Cohort B8 only)<br>○ Plasma concentration of LY3537982 as monotherapy and when administered in combination: PK parameters including, but not limited to, AUC, C_{max}, T_{max}, and degree of accumulation

Abbreviations: AE = adverse event; AUC = area under the concentration versus time curve; BOR = best overall response; C_{max} = maximum drug concentration; DCR = disease control rate; DLT = dose-limiting toxicity; DOR = duration of response; ECG = electrocardiogram; ORR = objective response rate; OS = overall survival; PFS = progression-free survival; PK = pharmacokinetics; SAE = serious adverse event; T_{max} = time to maximum plasma concentration; TTR = time to response.

STATISTICAL METHODS:

Safety Analyses:

The Safety Analysis Set will consist of all enrolled participants who receive at least 1 dose of any study drug. A baseline measurement and at least 1 laboratory or other safety-related measurement obtained after the start of study drug may be required for inclusion in the analysis of a specific safety parameter.

An SRC will be established to oversee the safety aspects of the study. The SRC will perform ongoing review of SAEs and other safety-related data throughout the conduct of the study. For Phase 1a dose escalation, the Phase 1a SRC will be convened for each dose escalation decision or as needed. For Phase 1b dose expansion combination cohorts, the Phase 1b SRC will be convened for safety lead-in decisions. For Phase 2 (Part F), a Phase 2 SRC will be convened to oversee safety aspects of the Phase 2 (Part F) portion of the study. For all Phase 1b and Phase 2 cohorts, the SRC will convene at a minimum of every 6 months.

Efficacy Analyses:

For Phase 1b Part G and Part H, efficacy analyses will be based on the intention-to-treat population, defined as all participants randomly assigned to study intervention, regardless of whether they take any doses of study intervention, or if they took the correct treatment.

For Phase 1a, other parts of Phase 1b, and Phase 2, the efficacy analysis will be conducted on the Safety Analysis Set unless otherwise specified. These analyses will be summarized by DL for Phase 1a dose escalation and by Phase 1b dose expansion cohorts and according to the treatment group for Part G and Part H.

Pharmacokinetic Analyses:

Plasma concentrations of LY3537982 will be determined with a validated bioanalytical assay. The following PK parameters will be calculated from plasma concentrations if appropriate: maximum drug concentration (C_{max}), time to maximum plasma concentration (T_{max}), area under the concentration versus time curve from time 0 to t (AUC_{0-t}), area under the concentration versus time curve from time 0 to ∞ ($AUC_{0-\infty}$), apparent oral clearance (CL/F), apparent volume of distribution (Vz/F), and terminal elimination half-life ($T_{1/2}$).

Serum concentrations of pembrolizumab and cetuximab at different time points may be summarized by descriptive statistics.

TABLE OF CONTENTS

1.0 INTRODUCTION....................39
1.1 Study Rationale....................39
1.2 Background....................39
1.3 *KRAS* G12C Inhibitor LY3537982....................42
1.3.1 Chemistry and Description....................42
1.3.2 Nonclinical....................43
1.3.3 Determination of Recommended Starting Dose....................44
1.3.3.1 Preliminary Clinical Data from LOXO-RAS-200001....................44
1.4 Combination of LY3537982 With Other Agents....................45
1.4.1 Phase 1b Cohort B4 and Part G: LY3537982 plus Pembrolizumab....................45
1.4.1.1 Pharmaceutical and Therapeutic Background....................45
1.4.2 Phase 1b Cohort B9: LY3537982 with Pembrolizumab Plus Pemetrexed and Carboplatin or Cisplatin Therapy....................48
1.4.3 Phase 1b Cohort C2 and Part H: LY3537982 plus Cetuximab....................48
1.5 Anticipated Risks....................49
1.5.1 Anticipated Risks of LY3537982....................49
1.5.1.1 Anticipated Risks of LY3537982 from Animal Studies....................49
1.5.2 Safety Experience Reported with Other RAS Inhibitors....................50
1.5.3 Anticipated Risks of Pembrolizumab Combination Therapy....................50
1.5.4 Anticipated Risks of Cetuximab Combination Therapy....................50
1.5.5 Anticipated Risks of Pembrolizumab Plus Pemetrexed and Carboplatin or Cisplatin Chemotherapy....................51
1.6 Benefit/Risk....................51
2.0 STUDY OBJECTIVES....................52
2.1 Primary Objectives....................52
2.2 Secondary Objectives....................53
2.3 Exploratory Objectives....................53
3.0 INVESTIGATIONAL PLAN....................55
3.1 Study Design....................55
3.1.1 Schema for Phase 1a—Dose Escalation....................55
3.1.2 Schema for Phase 1b—Dose Expansion....................55
3.1.3 Schema for Phase 1b—Dose Optimization (Part G and Part H)....................57
3.1.4 Other Aspects of Study Design....................58
3.1.5 Details of Phase 1a Dose Escalation....................59
3.1.5.1 Phase 1a Dose Escalation Method....................60

3.1.5.2 Backfill Criteria 62
3.1.5.3 Dose-Limiting Toxicity Determination for Phase 1a....63
3.1.6 Details of Phase 1b Dose Expansion and Dose Optimization....64
3.1.6.1 Safety Lead-In Cohorts in Phase 1b (Part B and Part C)....67
3.1.7 Details of Phase 2....70
3.2 Dose Scientific Rationale for Study Design 70
3.3 End of Study Definition....70
4.0 SELECTION OF STUDY POPULATION 71
4.1 Inclusion Criteria 71
4.2 Exclusion Criteria 77
4.3 Lifestyle Considerations 81
4.4 Screen Failures....81
5.0 TREATMENT....82
5.1 Study Intervention(s) Administered....82
5.1.1 Study Interventions in Cohort B9 84
5.1.2 Study Interventions in Part G....85
5.2 Preparation/Handling/Storage/Accountability....85
5.3 Study Intervention Compliance 86
5.4 Concomitant Therapy....86
5.4.1 Palliative Medicine and Supportive Care....89
5.4.2 Supportive Management for Diarrhea....90
5.4.3 Supportive Care with Programmed Death-1 Receptor Therapy (Pembrolizumab Cohort B4, Cohort B9, and Part G) 91
5.4.4 Supportive Care with EGFR Therapy (Cetuximab Cohorts) 91
5.4.5 Supportive Care for Pembrolizumab Plus Pemetrexed and Carboplatin or Cisplatin Chemotherapy (Cohort B9) 91
5.5 Dose Modification 92
5.5.1 Dose Modification of LY3537982 for Treatment-Related Adverse Events....93
5.5.1.1 Further Dose Modification Guidance for LY3537982 in Cohort B4, Cohort B9, and Part G....98
5.5.2 Dose Modification of Cetuximab....98
5.5.3 Dose Modification for Pembrolizumab....99
5.5.4 Dose Modification for Pemetrexed and Cisplatin or Carboplatin (Cohort B9) 99
5.6 Continued Access to Study Intervention After the End of the Study 101
5.6.1 Treatment After Study Completion....101

6.0 DISCONTINUATION OF STUDY INTERVENTION AND PARTICIPANT DISCONTINUATION/WITHDRAWAL 102
6.1 Discontinuation of Study Intervention 102
6.2 Participant Discontinuation/Withdrawal from the Study 103
6.2.1 Discontinuation of Inadvertently Enrolled Participants 103
6.3 Lost to Follow-Up 104
7.0 STUDY PROCEDURES AND ASSESSMENTS 105
7.1 Schedule of Assessments 106
7.1.1 Trial Schedules of Assessments 106
7.1.2 Continued Access Schedule of Assessments 139
7.2 Enrollment 140
7.3 Efficacy Assessments 140
[redacted]
7.4 Safety Assessments 146
7.4.1 Electrocardiograms 146
7.4.2 Clinical Safety Laboratory Assessments 147
7.4.2.1 Hepatic Safety Monitoring and Suggested Management 148
7.4.2.2 Guidance for Monitoring Renal Function 149
7.4.2.3 Guidance for ILD/Pneumonitis 149
7.4.2.4 Rash 150
7.4.2.5 Electrolyte Imbalance 150
7.4.2.6 Infusion Reactions 150
7.4.2.7 Immune-Mediated Reactions 151
7.5 Adverse Events and Serious Adverse Events 151
7.5.1 Severity of Adverse Events 152
7.5.2 Relatedness of Adverse Events 153
7.5.3 Serious Adverse Events 153
7.5.3.1 Suspected Unexpected Serious Adverse Reactions 154
7.5.4 Time Period and Frequency for Collecting AEs, SAEs, and Other Potentially Reportable Safety Event Information 155
7.5.5 Follow-Up of AEs, SAEs and ECIs 156
7.5.6 Adverse Event Reporting 156

7.5.7 Serious Adverse Event Reporting—Procedures for Investigators: Initial Report 156
7.5.7.1 SAE Reporting via an Electronic Data Collection Tool 156
7.5.7.2 SAE Reporting via Paper Form 157
7.5.8 Regulatory Reporting Requirements 157
7.5.9 Pregnancy 157
7.5.10 Death Events Due to Disease Progression 157
7.5.11 Complaint Handling 158
7.6 Treatment of Overdose 158
7.7 Pharmacokinetics 158
7.8 Pharmacodynamics 159
7.9 Genetics 159
7.10 Biomarkers 160
7.11 Intraparticipant Dose Escalation 162
7.12 Medical Resource Utilization and Health Economics 162
8.0 STATISTICAL CONSIDERATIONS 163
8.1 Statistical Hypotheses 163
8.2 Sample Size Determination 163
8.2.1 Phase 1a 163
8.2.2 Phase 1b 164
8.2.3 Phase 2 (Cohort F1) 165
8.3 Populations for Analyses 165
8.4 Statistical Analyses 166
8.4.1 General Statistical Considerations 166
8.4.2 Treatment Group Comparability 166
8.4.2.1 Participant Disposition 166
8.4.2.2 Participant Characteristics 167
8.4.2.3 Concomitant Therapy 167
8.4.2.4 Treatment Compliance 167
8.4.3 Efficacy Analyses 167
8.4.4 Safety Analyses 169
8.4.4.1 Safety Review Committees 170
8.4.5 Pharmacokinetics/Pharmacodynamics Analyses 170
8.4.6 Other Analyses 171
8.4.6.1 Subgroup Analyses 171
8.4.6.2 Biomarker Analyses 171

8.5 Interim Analyses 171
8.5.1 Phase 1a....171
8.5.2 Phase 1b and Phase 2 172
9.0 SUPPORTING DOCUMENTATION AND OPERATIONAL CONSIDERATIONS 177
9.1 Appendix 1: Regulatory, Ethical, and Study Oversight Considerations....177
9.1.1 Regulatory and Ethical Considerations....177
9.1.2 Informed Consent Process 177
9.1.3 Data Protection....178
9.1.4 Dissemination of Clinical Study Data....178
9.1.5 Data Quality Assurance....179
9.1.6 Data Capture System....180
9.1.7 Source Documents 180
9.1.8 Study and Site Closure....180
9.1.8.1 Discontinuation of the Study 180
9.1.8.2 Discontinuation of Study Sites....181
9.2 Appendix 2: Clinical Laboratory Tests....181
9.3 Appendix 3: Contraceptive Guidance and Collection of Pregnancy Information 183
9.3.1 Definitions....183
9.3.2 Contraception Guidance....184
9.3.3 Pregnancy Reporting....185
9.4 Appendix 4: Liver Safety: Suggested Actions and Follow-Up Assessments....186
[redacted]
9.6 Appendix 6: Cockcroft-Gault Formula....189
9.7 Appendix 7: Operating Characteristics of Continuous Safety Monitoring....189
9.8 Appendix 8: Dose Escalation Operating Characteristics....191
9.8.1 List of Designs 191
9.8.2 Simulation Scenarios....191
9.8.3 Simulation Results 192
9.9 Appendix 9: Dose Modification for Pembrolizumab....194
9.9.1 Definition of Dose-Limiting Toxicity....194
9.9.2 Dose Modification and Toxicity Management for Immune-Related AEs Associated with Pembrolizumab....195

9.9.3 Dose Modification and Toxicity Management of Infusion Reactions Related to Pembrolizumab 200
9.9.4 Other Allowed Dose Interruption for Pembrolizumab....201
9.9.5 Pembrolizumab Events of Clinical Interest....202
9.10 Appendix 10: Country-Specific Addenda to Clinical Protocol LOXO-RAS-20001....203
9.10.1 France 203
9.10.1.1 Section 3.1 Study Design....203
9.10.1.2 Section 4.1 Inclusion Criteria 203
9.10.1.3 Section 4.2 Exclusion Criteria 205
9.10.1.4 Protocol Section 6.2.1 Discontinuation of Inadvertently Enrolled Participants....206
9.10.1.5 Protocol Section 7.1 Schedule of Assessments....207
9.10.1.6 Protocol Section 7.11 Intraparticipant Dose Escalation 209
9.10.1.7 Protocol Section 9.2 Appendix 2: Clinical Laboratory Tests 210
10.0 REFERENCES....211

LIST OF TABLES

Table 1: Adverse Events Commonly Reported With Other RAS Inhibitors 50
Table 2: DLT Definitions for Phase 1a and Phase 1b Combination Therapy Cohorts Except Cohort B4, Cohort B9, and Part G 63
Table 3: Phase 1b Dose Expansion and Dose Optimization Cohorts 65
Table 4: Safety Lead-in Combination Dosing – Part B and Part C 68
Table 5: Phase 2 Cohort 70
Table 6: Study Interventions Administered 82
Table 7: Dosing for Cohort B9....84
Table 8: Dosing in Part G....85
Table 9: Dose Modifications of LY3537982 for Treatment-Related Adverse Events....94
Table 10: Dose Levels for Study Interventions in Cohort B9....99
Table 11: Recommended Dose Modifications for Chemotherapy: Hematological Toxicity 100
Table 12: Recommended Dose Modifications for Chemotherapy: Nonhematological Toxicity 100
Table 13: Schedule of Assessments for Participants Enrolled in Phase 1a....106
Table 14: Schedule of Additional Assessments for Participants Enrolled in Phase 1a Who Dose Escalate 112

Table 15: Schedule of Assessments for Participants Enrolled in Phase 1b (Except Part G) and Phase 2 113
Table 16: Sampling Schedule for PK and ECGs for Participants in Phase 1a 121
Table 17: Sampling Schedule for Additional PK and ECGs for Participants in Phase 1a who Dose Escalate 122
Table 18: Sampling Schedule for PK and ECGs for Participants in Phase 1b (Except Part G and Cohort B9) and Phase 2 123
Table 19: Sampling Schedule for PK and ECGs for Participants in Cohort B9 125
Table 20: Schedule of Assessments for Participants in Part G – Dose Optimization 126
Table 21: Sampling Schedule for PK for Participants in Part G – Dose Optimization 139
Table 22: Continued Access Schedule of Assessments 139
[redacted]
Table 24: Suggested Management for Hepatic Safety Monitoring 148
Table 25: Adverse Event Severity Classification 152
Table 26: Example Point Estimates of Incidence Rates and Corresponding 2-Sided Clopper-Pearson 95% Confidence Intervals for Sample Size of [redacted] 164
Table 27: Example Point Estimates of Incidence Rates and Corresponding 2-Sided Clopper-Pearson 95% Confidence Intervals for Sample Size of [redacted] 165
Table 28: Example Point Estimates of Incidence Rates and Corresponding 2 Sided Clopper-Pearson 95% Confidence Intervals for Sample Size of [redacted] 165
Table 29: DLT or DLT-Equivalent Boundaries Using Bayesian Toxicity Monitoring 172
[redacted]
Table 33: Dose Modification and Toxicity Management Guidelines for Immune-related AEs Associated with Pembrolizumab 196
Table 34: Pembrolizumab Infusion Reaction Dose Modification and Treatment Guidelines 200

LIST OF FIGURES

Figure 1: Phase 1a Dose Escalation Study Schema 55
Figure 2: Phase 1b Dose Expansion Study Schema 56
Figure 3: Phase 1b Dose Optimization (Part G) Study Schema 57

Figure 4: Phase 1b Dose Optimization (Part H) Study Schema 58

[redacted]

LIST OF ABBREVIATIONS AND DEFINITION OF TERMS

Abbreviation or Term	Definition
AE	adverse event
ALP	alkaline phosphatase
ALT	alanine aminotransferase
ANC	absolute neutrophil count
aPTT	activated partial thromboplastin time
ART	antiretroviral therapy
ASCO	American Society of Clinical Oncology
AST	aspartate aminotransferase
AUC	area under the concentration versus time curve
$AUC_{0-\infty}$	AUC time 0 to infinity
AUC_{0-t}	AUC from time 0 to t
AurA	Aurora A kinase
BCG	Bacillus Calmette–Guérin
BID	twice daily
BOR	best overall response
BSA	body surface area
BUN	blood urea nitrogen
C	Cycle
C1D1	Cycle 1 Day 1
CAP	College of American Pathologists
CD28	cluster of differentiation 28
CD3ζ	cluster of differentiation 3 zeta
CFR	Code of Federal Regulation
CI	confidence interval
CID	combination initial dose
CID_{+1}	one dose level above the CID
CID_{-1}	one dose level below the CID
CID_{-2}	two dose levels below the CID
CL/F	apparent oral clearance of drug
CLIA	Clinical Laboratory Improvement Amendments
C_{max}	maximum drug concentration
CNS	central nervous system
CR	complete response
CRC	colorectal cancer
CRCL	creatinine clearance
CRF	case report form
CSR	clinical study report
CT	computed tomography
CTCAE	Common Terminology Criteria for Adverse Events
CTLA-4	cytotoxic T-lymphocyte-associated protein 4
CYP	cytochrome P450
D	Day
DCR	disease control rate

Abbreviation or Term	Definition
DDI	drug-drug interaction
DILI	drug-induced liver injury
DL	dose level
DLT	dose-limiting toxicity
DNA	deoxyribonucleic acid
DOR	duration of response
DRESS	Drug Rash with Eosinophilia and Systemic Symptom
ECG	Electrocardiogram
ECI	event of clinical interest
ECOG	Eastern Cooperative Oncology Group
eCRF	electronic case report form
EDC	electronic data capture
EGFR	epidermal growth factor receptor
EI	equivalence interval
EOT	End of Treatment
ERB	Ethical Review Board
ERK	extracellular signal regulated kinase
ESMO	European Society for Medical Oncology
FDA	Food and Drug Administration
FT3	free triiodothyronine
FT4	free thyroxine
GCP	Good Clinical Practice
G-CSF	granulocyte-colony stimulating factor
GDP	guanosine diphosphate
GEF	guanine nucleotide exchange factor
GGT	gamma-glutamyl transferase
GI	Gastrointestinal
GnRH	gonadotropin-releasing hormone
GTP	guanosine triphosphate
HbsAg	Hepatitis B surface antigen
HBV	hepatitis B virus
HCV	hepatitis C virus
HCT	Hematocrit
HGB	Hemoglobin
HIV	human immunodeficiency virus
HR	hazard ratio
HRQoL	health-related quality of life
HRT	hormone replacement therapy
IB	Investigator’s Brochure
IC_{50}	50% inhibitory concentration
ICF	informed consent form
ICH	International Council for Harmonization
CCI	[redacted]

Abbreviation or Term	Definition
CCI [redacted]	[redacted]
[redacted]	[redacted]
[redacted]	[redacted]
[redacted]	[redacted]
Ig	Immunoglobulin
IgG4	immunoglobulin G4
IgV	Ig-variable-type
ILD	interstitial lung disease
INR	international normalized ratio
CCI [redacted]	[redacted]
[redacted]	[redacted]
[redacted]	[redacted]
Ir	immune-related
irAE	immune-related AE
IRB	Institutional Review Board
IRC	Independent Review Committee
CCI [redacted]	[redacted]
[redacted]	[redacted]
ISO/IEC	International Organization for Standardization/International Electrotechnical Commission
ITT	Intention-to-treat
CCI [redacted]	[redacted]
IV	Intravenous
IVDR	In Vitro Diagnostic Medical Devices Regulation (EU) 2017/746
IWRS	interactive web response systems
KM	Kaplan Meier
LC-MS/MS	liquid chromatography-tandem mass spectrometry
LFT	liver function test
LHRH	luteinizing hormone-releasing hormone
LLN	lower limit of normal
LTFU	long-term follow-up
LVEF	left ventricular ejection fraction
mAb	monoclonal antibody
MAPK	mitogen-activated protein kinase
MATE1/2K	multidrug and toxin extrusion protein 1 and multidrug and toxic extrusion protein 2
MDRD	Modification of Diet in Renal Disease
Medical Monitor	an individual responsible for the medical conduct of the study; responsibilities of the Medical Monitor may be performed by a physician, clinical research scientist, or other medical officer, such as a nurse practitioner, physician assistant, etc.
MRI	magnetic resonance imaging
mRNA	messenger ribonucleic acid
MTD	maximum tolerated dose
mTPI-2	modified toxicity probability interval 2
MUGA	multigated acquisition scan

Abbreviation or Term	Definition
N	number of participants
NCCN	National Comprehensive Cancer Network
NCI	National Cancer Institute
NL-NT	new lesion-nontarget
NL-T	new lesion-target
NOAEL	no-observed-adverse-effect-level
NSAID	nonsteroidal anti-inflammatory drug
NSCLC	non-small cell lung cancer
NT-Pro-BNP	N-terminal pro b-type natriuretic peptide
OATP	organic anion transporting polypeptide
OCT2	organic cation transporter 2
ORR	objective response rate
OS	overall survival
PABA	para-aminobenzoic acid
PBPK	physiologically based pharmacokinetic
PD	progressive disease
PD-1	programmed cell death 1
PDAC	pancreatic ductal adenocarcinoma
PD-L1	programmed cell death ligand 1
PD-L2	programmed cell death ligand 2
PDX	patient-derived xenograft
PET	positron emission tomography
PFS	progression-free survival
P-gp	p-glycoprotein
PGx	Pharmacogenomics
PI	Principal Investigator
PK	Pharmacokinetic
PKCθ	protein kinase C-theta
PLT	Platelets
PO	Orally
PR	partial response
Prob	Probability
pT	target toxicity rate
PT	prothrombin time
Q2W	every 2 weeks
Q3W	every 3 weeks
QD	once daily
QTc	QT interval corrected for heart rate
QTcF	QT interval corrected for heart rate (Fridericia's formula)
RBC	red blood cell
RECIST v1.1	Response Evaluation Criteria in Solid Tumors Version 1.1
RNA	ribonucleic acid
RP2D	recommended Phase 2 dose
$RP2D_M$	monotherapy recommended Phase 2 dose

Abbreviation or Term	Definition
$RP2D_{M-1}$	1 dose level below the monotherapy recommended Phase 2 dose
$RP2D_{M-X}$	X dose levels below the monotherapy recommended Phase 2 dose
RTK	receptor tyrosine kinase
SAE	serious adverse event
SAP	Statistical Analysis Plan
SAR	serious adverse reaction
SD	stable disease
SFU	safety follow-up
SJS	Stevens-Johnson Syndrome
SoA	Schedule of Assessments
SPF	sun protection factor
SpO2	oxygen saturation
SRC	Safety Review Committee
SRS	stereotactic radiosurgery
SUSAR	suspected unexpected serious adverse reaction
$T_{1/2}$	terminal elimination half-life
T1DM	type 1 diabetes mellitus
TBL	total bilirubin
TEAE	treatment-emergent adverse event
TEN	toxic epidermal necrolysis
T_{max}	time to maximum plasma concentration
TMDD	target-mediated drug disposition
TPS	tumor proportion score
T-reg	regulatory T-cell
TSH	thyroid-stimulating hormone
TTE	transthoracic echocardiography
TTR	time to response
UGT1A3	uridine diphosphate-glucuronosyl-transferase family 1, member A3
ULN	upper limit of normal
V	Visit
VTE	venous thromboembolic event
Vz/F	apparent volume of distribution
WBC	white blood cell
WBRT	whole brain radiation therapy
WOCBP	women of childbearing potential
Wt	Weight
ZAP70	zeta-chain-associated protein kinase

1.0 INTRODUCTION

1.1 Study Rationale

More than 30% of all cancer types possess mutations in RAS. *KRAS* mutations account for approximately 85% of RAS-associated cancers in humans and have typically been associated with worse OS and increased resistance to treatments compared to *KRAS* wild type tumors (Dinu et al. 2014; Ferrer et al. 2018; Windon et al. 2018). *KRAS* mutations occur in approximately 16% to 40% of NSCLC, and G12C mutations represent approximately 40% of total mutations in NSCLC (Fernández-Medarde and Santos 2011). However, due to its lack of deep pockets for binding of small molecule inhibitors, RAS is typically deemed as "undruggable." Various anti-RAS therapeutic strategies have been proven largely ineffective (Ferrer et al. 2018; Román et al. 2018; O'Bryan 2019). Recent studies have been focusing on *KRAS* mutation-specific therapies.

LY3537982 (which has been granted the United States Adopted Name of olomorasib) is a potent and orally bioavailable small molecule inhibitor of KRAS G12C protein. LY3537982 covalently binds the mutated cysteine at position 12 in KRAS G12C and locks KRAS in its GDP-bound inactive form and has demonstrated significant antitumor activity in preclinical models against a wide range of *KRAS* G12C-mutant tumor models.

Study LOXO-RAS-20001 is a Phase 1/2 multicenter, open-label study with dose escalation followed by dose expansions of LY3537982 as monotherapy and in combination with the following agents: pembrolizumab, cetuximab, and pembrolizumab plus pemetrexed and carboplatin or cisplatin therapy. The primary objective of this study is to determine a RP2D of LY3537982 when administered alone and safety and tolerability when administered alone or in combination in participants with *KRAS* G12C-mutant advanced solid tumors.

1.2 Background

The RAS family of genes, including *KRAS*, *HRAS*, and *NRAS*, are the most common oncogenes in cancer (Lindsay et al. 2018). RAS proteins function as a GTPase and have a central coordinating role connecting upstream signals from cell surface receptors to downstream signaling pathways associated with cancer (Lindsay et al. 2018). Typically, RAS proteins stay in a GDP-bound inactive form in quiescent cells. The downstream signaling is activated when GDP is released from RAS via the action of guanine exchange factors and subsequently binds to GTP with high specificity and affinity. The exchange of GDP for GTP leads to characteristic changes in RAS conformation, resulting in recruitment of downstream effector proteins, such as RAF and PI3K, and activation of key signaling pathways.

Among all RAS genes, *KRAS* mutations account for approximately 85% of RAS-associated cancers in humans: about 90% of pancreatic cancers (Zeitouni et al. 2016), 35% to 45% of CRC (Tan and Du 2012; Wilson and Tolias 2016), and 25% NSCLC (Román et al. 2018). *KRAS* mutations are well characterized oncogenic drivers and have typically been associated with worsened OS and increased resistance to treatments than *KRAS* wild type tumors

(Dinu et al. 2014; Ferrer et al. 2018; Windon et al. 2018). Most *KRAS* mutations affect exon 2 (codon 12/13) and exon 3 (codon 61), leading to *KRAS* remaining in the GTP-bound active form (Román et al. 2018). Evidence suggests variability in biological activity across *KRAS* mutant cancers. In patients with NSCLC, the presence of *KRAS* G12C mutation in resected tumors was associated with worse disease-free survival and OS compared to other *KRAS* mutations and to *KRAS* wild type (Nadal et al. 2014). *KRAS* G12C accounts for 12% of all *KRAS* mutations and 40% of *KRAS* mutations in NSCLC (Fernández-Medarde and Santos 2011; Prior et al. 2012). The overall prevalence of *KRAS* G12C mutations is about 4% across all solid tumors, approximately 14% in NSCLC, approximately 3% in CRC, and approximately 1% in pancreatic cancer (Bailey et al. 2016; Campbell et al. 2016; Giannakis et al. 2016; Jordan et al. 2017; Thein et al. 2020; Nassar et al. 2021).

RAS has been a target for therapeutic inhibition for the past 3 decades (Wilson et al. 2016). However, due to its lack of deep pockets for binding of small molecule inhibitors, RAS is typically deemed as “undruggable” (O’Bryan 2019). Various therapeutic strategies, including targeting *KRAS* membrane association, targeting downstream effectors of *KRAS* pathways, and inhibition of synthetic lethal targets, have been investigated in both preclinical and clinical settings and have, with some notable exceptions, proven ineffective (Ferrer et al. 2018; Román et al. 2018; O’Bryan 2019). In the SELECT-1 Phase 3 study, adding the MEK inhibitor, selumetinib, to docetaxel did not show significant improvement in PFS or OS in second-line advanced NSCLC patients with *KRAS* mutations: median PFS was 3.9 months with selumetinib plus docetaxel compared to 2.8 months with docetaxel alone (HR, 0.93) with a median OS of 8.7 months and 7.9 months (HR 1) and an ORR of 20.1% and 13.7%, respectively (Jänne et al. 2017).

Until recently, approaches to treat NSCLC and CRC patients with *KRAS* mutations included standard chemotherapy, antiangiogenics, or immunotherapy, if applicable (Aredo and Padda 2018). Responses are variable across different patient subsets, highlighting the challenges in developing effective therapy for patients with KRA*S* mutant NSCLC, and such therapy is not directed specifically at KRA*S* mutations (Kim et al. 2017; Rittmeyer et al. 2017). For CRC patients with unresectable metastatic disease and KRAS mutations, chemotherapy alone or in combination with antiangiogenic therapy is standard of care in first- and second-line therapy with initially good response to therapy followed universally by progression. Upon progression, therapy with either regorafenib or trifluridine plus tipiracil is preferred and represents current standard of care in third-line therapy, regardless of RAS mutation status (Grothey et al. 2013; Mayer et al. 2015). In the CORRECT study (Grothey et al. 2013), median OS for all previously treated CRC patients (including about 50% to 60% of patients with known *KRAS* mutations) was 6.4 months in the regorafenib group, with a response rate of 1% and median PFS of 1.9 months, indicating the inadequacy of current systemic therapy and high unmet medical need.

Nonetheless, a new wave of development of anti-*KRAS* therapies has been stimulated by recent research findings, particularly focusing on *KRAS* mutation-specific therapies. These therapies

include molecules targeting the inactive form of *KRAS* trapped in GDP-bound form and demonstrate activity in preclinical models (Ostrem et al. 2013; Lito et al. 2016; Patricelli et al. 2016; Janes et al. 2018).

These preclinical observations were further supported by recent disclosures on ongoing studies evaluating the safety and tolerability of KRAS G12C inhibitors, sotorasib (Hong et al. 2020) and adagrasib (Jänne et al. 2020; Johnson et al. 2020). In the Phase 1 trial of sotorasib (CodeBreaK100), a total of 129 patients with various locally advanced or metastatic solid tumors (59 with NSCLC, 42 with CRC, and 28 with other solid tumors) harboring *KRAS* G12C mutations were treated (Hong et al. 2020). The toxicity profile was favorable with no observed DLT at the DLs tested (180 to 960 mg QD) and preliminary results indicated a potential for antitumor activity when administered as a monotherapy based on the response seen in NSCLC patients (confirmed PR, 32.2%; disease control, 88.1%; median PFS, 6.3 months), CRC patients (confirmed PR, 7.1%; disease control, 73.8%; median PFS, 4.0 months), and other solid tumors (confirmed PR, 14.3%; disease control, 75.0%; median PFS, not reported). Similar safety (no new safety findings) and efficacy (ORR, 37.1%; DOR, 10.1 months; median PFS, 6.8 months; DCR, 80.6%) were also reported for sotorasib at RP2D of 960 mg QD (n = 126) from the Phase 2 trial of CodeBreaK100. Favorable toxicity profile and antitumor activity were also observed in patients with NSCLC (objective response, 45%; PR or CR or SD, 96%) (Hong et al. 2020), CRC (objective response, 3 [17%]; disease control, 94%), and other solid tumors (PR, 66.7%) (Johnson et al. 2020) harboring *KRAS* G12C mutations when treated with adagrasib, another KRAS G12C inhibitor. Data from a subsequent Phase 2 NSCLC study of sotorasib (Skoulidis et al. 2021) subsequently led to the accelerated approval of sotorasib (LUMAKRAS®) by the United States FDA for patients with *KRAS* G12C-mutant NSCLC who had received at least 1 prior therapy. Early clinical data in CRC showed ORR of approximately 7.1% for monotherapy sotorasib (Hong et al. 2020) and approximately 17% for adagrasib (Johnson et al. 2020). Recent studies have revealed higher confirmed response rates of approximately 28% for sotorasib (Fakih et al. 2021) and approximately 39% for adagrasib (Weiss et al. 2021) when given in combination with EGFR antibodies. These findings support that KRAS G12C can be effectively targeted with a mutant specific inhibitor alone or in combination to provide potential clinical benefit for certain cancers that carry a *KRAS* G12C mutation.

Additionally, the development and approval of several targeted therapies across multiple tumor types have highlighted several observations, especially in the context of targeting the RAS/RAF/MEK pathway:

1. There is potential for innate and acquired resistance in most patients (Prahallad et al. 2012; Van Emburgh et al. 2016).
2. There is variable activity across different tumor types based on histology, which has been clearly demonstrated in the case of *BRAF* V600E mutations in CRC, melanoma, and NSCLC (Sosman et al. 2012; Bendell et al. 2014; Planchard et al. 2016).

3. Combination therapies may have the potential to overcome innate resistance and delay emerging resistance as evidenced by data obtained in relevant preclinical models and clinical outcomes seen with combination therapy targeting the RAS/RAF/MEK pathway (Bendell et al. 2014; Long et al. 2014; Kopetz et al. 2015; Kopetz et al. 2017, Tabernero et al. 2021).

Mutations in the RAS/RAF pathway have remained a challenge for single–agent-based regimens due to a compensatory mechanism leading to rapid evolution of acquired resistance (Villanueva et al. 2010; Prahallad et al. 2012; Wagle et al. 2014; Ahronian et al. 2015). This has been demonstrated clinically with *BRAF* V600E mutant NSCLC where the combination of BRAF and MEK inhibitors was more effective than the single agent (Planchard et al. 2016). Similarly, a triple combination of EGFR/MEK/BRAF inhibitors was superior to standard of care in *BRAF* V600E mutant CRC (Kopetz et al. 2019). While further and more thorough studies are needed for KRAS G12C inhibitors as monotherapy, in patients with *KRAS* G12C-mutant CRC, at least, the lack of objective response seen on treatment with sotorasib (Hong et al. 2020) and adagrasib (Johnson et al. 2020) suggests that combination therapy may still be required with KRAS G12C-targeted therapy in this setting, and possibly others.

1.3 *KRAS* G12C Inhibitor LY3537982

1.3.1 Chemistry and Description

LY3537982 is a potent, mutant-selective, and orally bioavailable small molecule covalent binder of KRAS G12C with no measured activity against wild type *KRAS* or other *KRAS* mutant proteins. LY3537982 irreversibly binds to the mutated cysteine at position 12 in the KRAS G12C protein in GDP-bound inactive form and inhibits KRAS activity and downstream phospho-extracellular signal regulated kinase in *KRAS* G12C-mutant cell lines. In cell proliferation assays across a panel of tumor cells, LY3537982 was potent in many tumor cells harboring a *KRAS* G12C mutation with a range of sensitivity. In contrast, LY3537982 was inactive in tumor cells with wild type or non-*KRAS* G12C mutations. Synergy or additivity was observed for LY3537982 in combination with several targeted agents including abemaciclib (CDK4 and CDK6 inhibitor), TNO155 (SHP2 inhibitor), erlotinib (EGFR inhibitor), LY3214996 (ERK1/2 inhibitor), and LY3295668 (AurA inhibitor) in cell proliferation assays. In animal models, LY3537982 demonstrated dose- and time-dependent inhibition of KRAS activity and downstream signaling and led to significant tumor growth inhibition/regression in a panel of *KRAS* G12C-mutant NSCLC, CRC, and PDAC xenograft and PDX models. In addition, LY3537982 showed synergistic or additive effects in *KRAS* G12C-mutant in vivo models when combined with various agents including abemaciclib, erlotinib, cetuximab, LY3214996, LY3295668, a SHP2 inhibitor (RMC-4550), and an anti-PD-1 antibody or PD-L1 antibody.

CCI [redacted]

1.3.2 Nonclinical

The pharmacology (absorption, distribution, metabolism, and excretion characteristics) and the toxicity of LY3537982 have been adequately assessed in compliance with worldwide guidelines, as described below:

- A potent selective covalent inhibitor of KRAS G12C
- A highly selective KRAS G12C-mutant inhibitor without appreciable activity against wild type RAS and other KRAS mutant proteins

CCI [redacted]

See Section 4 of the IB for additional details.

1.3.3 Determination of Recommended Starting Dose

The initial human dose of 50 mg BID was selected based on projected target inhibition and safety margin. Treatment with 50 mg BID is anticipated to achieve target inhibition levels associated with efficacy, as predicted by the PK/pharmacodynamics model developed using in vivo target inhibition data from mouse xenograft studies. Based on the nonclinical toxicology and safety pharmacology data discussed in IB Section 4.4, no adverse effects are expected over the entire proposed 50 to 150 mg BID dose range, and higher DLs may be supported by clinical safety determined at lower DLs.

At the time of study initiation, the projected human efficacious dose ranged from 23 mg BID to 94 mg BID. Treatment with LY3537982 50 mg BID was predicted to achieve target inhibition of 85% to 90% at steady-state trough concentrations in humans and was predicted to lead to response in participants. In addition, based on dose and projected human exposure, no adverse effects were expected in the proposed clinical dose range of 50 to 150 mg BID. The starting dose of 50 mg BID was 54-fold below the NOAEL in rats and 24-fold below the NOAEL in dogs treated for 1 month with LY3537982.

1.3.3.1 Preliminary Clinical Data from LOXO-RAS-200001

As of 03 January 2023, 84 patients were treated across Phase 1a dose escalation with LY3537982 monotherapy at 4 dose levels: 50, 100, 150, and 200 mg BID. Roughly dose-proportional increases in steady state exposure were observed consistently from 50 to 150 mg BID, with all doses exceeding the EC90 throughout the majority of the dosing interval (IB, Section 5.1.1.1). LY3537982 monotherapy was well tolerated at all DLs evaluated, with no dose-safety relationship from 50 to 200 mg BID. Preliminary monotherapy efficacy was observed across all monotherapy DLs evaluated and in multiple tumor types (Murciano-Goroff et al. 2023).

Clinical response has also been observed across multiple LY3537982 DLs evaluated in combination with pembrolizumab in participants with NSCLC (Murciano-Goroff et al. 2023), with an approximate 80% ORR in first-line participants (Burns et al. 2024, in press). Due to the continued favorable safety profile and encouraging efficacy of the combination (Burns et al. 2024, in press), LY3537982 at 50 and 100 mg BID in combination with pembrolizumab continues to be investigated in first-line NSCLC participants in dose optimization cohorts (Part G).

In light of the clinical efficacy observed at all doses tested in dose escalation and in dose expansion, evaluation of 50, 100, and 150 mg is justified, depending on the disease/setting-specific expansion cohort, to determine the full benefit/risk profile of LY3537982.

1.4 Combination of LY3537982 With Other Agents

Although LY3537982 is a potent KRAS G12C inhibitor measured by inhibition of KRAS and downstream ERK activation, tumor cell lines harboring the same *KRAS* G12C mutation showed a range of sensitivity to LY3537982, suggesting other mechanisms may be involved in determining response. LY3537982 also demonstrated different degrees of antitumor activity in *KRAS* G12C-mutant animal models. In order to achieve a more durable response and in a broader range of tumors, combining LY3537982 with other therapies could provide superior clinical activity. LY3537982 combinations were evaluated in multiple *KRAS* G12C-mutant in vivo models including 2 lung cancer xenograft models (H358 and H1373), 1 lung cancer PDX model (EL3187), and 1 CRC xenograft model (SW837). In addition, LY3537982 alone at suboptimal doses and monotherapy of abemaciclib, erlotinib, cetuximab, ERK inhibitor LY3214996, or AurA inhibitor were evaluated in these models. LY3295668 showed various antitumor activities in these models. The combination of LY3537982 with each of these targeted therapies led to synergy or improved antitumor activity, based on tumor growth inhibition and tumor regression. Additionally, LY3537982 was evaluated in combination with an anti-PD-1 or anti-PD-L1 antibody in a mouse syngeneic model with *KRAS* G12C. These combinations demonstrated increased efficacy over monotherapy and no adverse effects on body weight. Combination of LY3537982 with a SHP2 inhibitor (RMC-4550) showed robust synergy in multiple NSCLC xenograft and PDX models.

1.4.1 Phase 1b Cohort B4 and Part G: LY3537982 plus Pembrolizumab

Pembrolizumab is a potent humanized IgG4 mAb with high specificity of binding to the PD-1 receptor, thus inhibiting its interaction with PD-L1 and PD-L2. Based on preclinical in vitro data, pembrolizumab has high affinity and potent receptor blocking activity for PD-1. Pembrolizumab has an acceptable preclinical safety profile and is in clinical development as an IV immunotherapy for advanced malignancies. Keytruda® (pembrolizumab) is indicated for the treatment of patients across a number of indications. For more background and details on specific indications refer to the IB.

Refer to the IB/approved labeling for detailed background information on pembrolizumab.

1.4.1.1 Pharmaceutical and Therapeutic Background

The importance of intact immune surveillance function in controlling outgrowth of neoplastic transformations has been known for decades (Disis 2010). Accumulating evidence shows a correlation between tumor-infiltrating lymphocytes in cancer tissue and favorable prognosis in various malignancies. In particular, the presence of CD8+ T cells and the ratio of CD8+ effector T-cells/FoxP3+ T-regs correlates with improved prognosis and long-term survival in solid malignancies, such as ovarian, colorectal, and pancreatic cancer; hepatocellular carcinoma; malignant melanoma; and renal cell carcinoma. Tumor-infiltrating lymphocytes can be expanded ex vivo and reinfused, inducing durable objective tumor responses in cancers such as melanoma (Dudley et al. 2005; Hunder et al. 2008).

The PD-1 receptor-ligand interaction is a major pathway hijacked by tumors to suppress immune control. The normal function of PD-1, expressed on the cell surface of activated T-cells under healthy conditions, is to down-modulate unwanted or excessive immune responses, including autoimmune reactions. PD-1 (encoded by the gene *Pdcd1*) is an Ig superfamily member related to CD28 and CTLA-4 that has been shown to negatively regulate antigen receptor signaling upon engagement of its ligands (PD-L1 and/or PD-L2) (Greenwald et al. 2005; Okazaki et al. 2001).

The structure of murine PD-1 has been resolved (Zhang et al. 2004). PD-1 and its family members are type I transmembrane glycoproteins containing an IgV-type domain responsible for ligand binding and a cytoplasmic tail responsible for the binding of signaling molecules. The cytoplasmic tail of PD-1 contains 2 tyrosine-based signaling motifs, an immunoreceptor tyrosine-based inhibition motif, and an immunoreceptor tyrosine-based switch motif. Following T-cell stimulation, PD-1 recruits the tyrosine phosphatases, SHP-1 and SHP-2, to the immunoreceptor tyrosine-based switch motif within its cytoplasmic tail, leading to the dephosphorylation of effector molecules such as CD3ζ, PKCθ, and ZAP70, which are involved in the CD3 T-cell signaling cascade (Okazaki et al. 2001; Chemnitz et al. 2004; Sheppard et al. 2004; Riley 2009). The mechanism by which PD-1 down-modulates T-cell responses is similar to, but distinct from, that of CTLA-4, because both molecules regulate an overlapping set of signaling proteins (Parry et al. 2005; Francisco et al. 2010). As a consequence, the PD-1/PD-L1 pathway is an attractive target for therapeutic intervention in NSCLC.

1.4.1.1.1 Preclinical and Clinical Trials

Therapeutic studies in mouse models have shown that administration of antibodies blocking PD-1/PD-L1 interaction enhances infiltration of tumor-specific CD8+ T-cells and ultimately leads to tumor rejection, either as a monotherapy or in combination with other treatment modalities (Hirano et al. 2005; Blank et al. 2004; Weber 2010; Strome et al. 2003; Spranger et al. 2014; Curran et al. 2010; Pilon et al. 2010). Anti-mouse PD-1 or anti-mouse PD-L1 antibodies have demonstrated antitumor responses in models of squamous cell carcinoma, pancreatic carcinoma, melanoma, acute myeloid leukemia and colorectal carcinoma (Strome et al. 2003; Curran et al. 2010; Pilon et al. 2010; Nomi et al. 2007; Zhang et al. 2004). In such studies, tumor infiltration by CD8+ T-cells and increased IFN-γ, granzyme B, and perforin expression were observed, indicating that the mechanism underlying the antitumor activity of PD-1 checkpoint inhibition involved local infiltration and activation of effector T-cell function in vivo (Curran et al. 2010). Experiments have confirmed the in vivo efficacy of anti-mouse PD-1 antibody as a monotherapy, as well as in combination with chemotherapy, in syngeneic mouse tumor models (see the IB).

Anti-PD-1 monoclonal antibodies have synergistic effects with RAS inhibitors. Sotorasib increased immune cell infiltration (Canon et al. 2019). Furthermore, the combination of AMG 510 and anti-PD-1 therapy established a memory T cell response against both the CT-26 *KRAS* G12C cells and the parental CT-26 tumor cells. Thus, the potential synergy of pembrolizumab and LY3537982 provides rationale for this combination to be explored.

LY3537982 was evaluated in combination with anti-PD-1 or anti-PD-L1 antibody in a mouse syngeneic model. The results revealed that LY3537982 at 30 mg/kg had significant single-agent activity with tumor growth inhibition of 88.4%; however, the tumors started to regrow when LY3537982 treatment stopped. Monotherapy of anti-PD-L1 or anti-PD-1 showed moderate single-agent activity with average tumor growth inhibition of 36.1% or 70.7% and CR of 0% or 10%, respectively. Combination of LY3537982 with either an anti-PD-L1 antibody or an anti-PD-1 antibody showed durable tumor regression and achieved CR of 40% (4 out of 10 animals) or 60% (6 out of 10 animals), respectively. Both combinations were well tolerated with no significant body weight loss or adverse effects observed.

1.4.1.1.2 Justification for Pembrolizumab Dose

The planned dose of pembrolizumab for this study is 200 mg Q3W. Based on the totality of data generated in the Keytruda development program, 200 mg Q3W is an appropriate dose of pembrolizumab for adults across all indications. As outlined below, this dose is justified by:

- Clinical data from 8 randomized studies in melanoma and NSCLC indications demonstrating flat dose- and exposure-efficacy relationships from 2 mg/kg Q3W to 10 mg/kg Q2W representing an approximate 5 to 7.5-fold exposure range (refer to the pembrolizumab IB),
- Population PK analysis showing that both fixed dosing and weight-based dosing provides similar control of PK variability with considerable overlap in the distributions of exposures, supporting suitability of 200 mg Q3W,
- Clinical data showing meaningful improvement in benefit-risk including overall survival at 200 mg Q3W across multiple indications, and
- Pharmacology data showing full target saturation in both systemic circulation (inferred from PK data) and tumor (inferred from PBPK analysis) at 200 mg Q3W.

Among the 8 randomized dose-comparison studies, a total of 2262 participants were enrolled with melanoma and NSCLC, covering different disease settings (treatment naïve, previously treated, PD-L1 enriched, and all-comers) and different treatment settings (monotherapy and in combination with chemotherapy). Five studies compared 2 mg/kg Q3W versus 10 mg/kg Q2W (KN001 Cohort B2, KN001 Cohort D, KN002, KN010, and KN021), and 3 studies compared 10 mg/kg Q3W versus 10 mg/kg Q2W (KN001 Cohort B3, KN001 Cohort F2 and KN006). All of these studies demonstrated flat dose- and exposure-response relationships across the doses studied representing an approximate 5- to 7.5-fold difference in exposure. The 2 mg/kg (or 200 mg fixed dose) Q3W provided similar responses to the highest doses studied. Subsequently, flat dose-exposure-response relationships were also observed in other tumor types including head and neck cancer, bladder cancer, gastric cancer, and classical Hodgkin Lymphoma, confirming 200 mg Q3W as the appropriate dose independent of the tumor type. These findings are consistent with the mechanism of action of pembrolizumab, which acts by interaction with immune cells, and not via direct binding to cancer cells.

Additionally, pharmacology data clearly show target saturation at 200 mg Q3W. First, PK data in KN001 evaluating TMDD conclusively demonstrated saturation of PD-1 in systemic circulation at doses much lower than 200 mg Q3W. Second, a PBPK analysis was conducted to predict tumor PD-1 saturation over a wide range of tumor penetration and PD-1 expression. This evaluation concluded that pembrolizumab at 200 mg Q3W achieves full PD-1 saturation in both blood and tumor.

Finally, population PK analysis of pembrolizumab, which characterized the influence of body weight and other participant covariates on exposure, has shown that the fixed dosing provides similar control of PK variability as weight-based dosing, with considerable overlap in the distribution of exposures from the 200 mg Q3W fixed dose and 2 mg/kg Q3W dose. Supported by these PK characteristics and given that fixed dose has advantages of reduced dosing complexity and reduced potential of dosing errors, the 200 mg Q3W fixed dose was selected for evaluation across all pembrolizumab protocols.

1.4.2 Phase 1b Cohort B9: LY3537982 with Pembrolizumab Plus Pemetrexed and Carboplatin or Cisplatin Therapy

A standard of care for first-line treatment for patients with locally advanced or metastatic NSCLC without driver alterations is platinum-based chemotherapy regimens with immunotherapy. In the Phase 3 study KEYNOTE-189, patients treated with the combination of pembrolizumab with pemetrexed plus platinum therapy demonstrated better outcomes compared to those treated in the control arm with placebo with pemetrexed plus platinum therapy in PFS (9.0 months vs. 4.9 months; HR = 0.48) and median OS (22.0 months vs. 10.7 months; HR = 0.56) [Gadgeel et al. 2020]). The benefits were observed regardless of PD-L1 status. For patients with high PD-L1 expression (TPS ≥ 50%), monotherapy with a PD-L1 inhibitor may be sufficient: In a randomized Phase 3 trial, participants treated with pembrolizumab monotherapy achieved better outcomes compared to those treated with chemotherapy (median OS of 30.0 months vs. 14.2 months; HR 0.49 when adjusted for crossover [Reck et al. 2019]).

Patients with *KRAS* mutations may respond favorably in the context of modern immunotherapy, although detailed outcome data are limited (Dong et al. 2017; Lee et al. 2018; Sun et al. 2021, Nakajima et al. 2022). The clinical development of targeted agents for patients with *KRAS* G12C-mutant NSCLC has confirmed that these agents can provide benefit in second-line treatment, albeit with modest efficacy and a less than optimal safety profile. The combination of pembrolizumab plus pemetrexed and platinum therapy with a KRAS G12C inhibitor may improve outcomes in current first-line therapies.

1.4.3 Phase 1b Cohort C2 and Part H: LY3537982 plus Cetuximab

Cetuximab is an anti-EGFR monoclonal antibody that is approved for the treatment of patients with head and neck cancer and in patients with metastatic RAS wild type CRC (Price and Cohen 2012; Goldberg et al. 2018). Targeting the RAS/RAF pathway in patients with CRC has proven to be challenging. While *BRAF* V600E is well-established as a prognostic

marker (associated with worse median OS), it is often associated with the lack of response to cetuximab. Additionally, a decade of preclinical and clinical work has demonstrated a lack of efficacy of a single-agent BRAF inhibitor in CRC (Kopetz et al. 2015). An emergent compensatory mechanism via EGFR receptor signaling can overcome BRAF inhibition (Prahallad et al. 2012), and improved responses are seen when cetuximab is added to vemurafenib and irinotecan (Kopetz et al. 2017). More recently, the combination of encorafenib, binimetinib, and cetuximab has demonstrated improved ORR, PFS, and OS over standard of care (Kopetz et al. 2019). The combination of LY3537982 and cetuximab in CRC xenograft models demonstrated improved efficacy compared to either agent dosed as monotherapy and was well tolerated.

1.5 Anticipated Risks

1.5.1 Anticipated Risks of LY3537982

As LY3537982 has not been studied in human participants, the potential risks, contraindications, and warnings are theoretical and are based on observations from animal testing.

Since LY3537982 is in an early stage of drug development, it is possible that unforeseen, unknown, or unanticipated drug reactions and toxicities may occur. However, the study protocol is designed to mitigate risks to participants through a detailed plan for cautious dose escalation, careful safety monitoring, systematic review of AEs, SAEs, PK evaluation, and active pharmacovigilance review to assess for safety signals or trends.

Refer to the LY3537982 IB for additional information on the known and expected benefits, risks, and reasonably expected AEs of LY3537982.

1.5.1.1 Anticipated Risks of LY3537982 from Animal Studies

While there were no adverse effects observed in rats or dogs at planned clinical DLs and projected clinical exposure levels, effects observed in nonclinical studies at significantly higher doses than the maximum planned clinical dose consisted of:

CCI [redacted]

CCI [redacted]

1.5.2 Safety Experience Reported with Other RAS Inhibitors

Adverse events commonly reported with other RAS inhibitors are shown in Table 1.

Table 1: Adverse Events Commonly Reported With Other RAS Inhibitors

Diarrhea	Increased aspartate aminotransferase	Increased blood creatinine
Fatigue	Anemia	QT prolongation
Nausea	Dizziness	
Vomiting	Increased alanine aminotransferase	
Abdominal pain	Constipation	
Dyspnea	Pyrexia	
Cough	Insomnia	
Back pain	Myalgia	
Decreased appetite	Peripheral edema	
Headache	Arthralgia	

Hong et al. 2020 ; Jänne et al. 2020 ; Johnson et al. 2020

1.5.3 Anticipated Risks of Pembrolizumab Combination Therapy

Pembrolizumab is a standard of care for various indications (see Section 1.4.1). The safety profile of pembrolizumab is well documented (FDA 2021). Pembrolizumab as a single agent is associated with immune-mediated adverse reactions and infusion-related reactions. Other pembrolizumab-associated adverse reactions includes fatigue, musculoskeletal pain, rash, diarrhea, pyrexia, cough, decreased appetite, pruritus, dyspnea, constipation, pain, abdominal pain, nausea, and hypothyroidism. Since there were few important effects in nonclinical toxicology studies of LY3537982, there is a low risk of overlapping toxicity when combined with pembrolizumab. Monitoring and dose modifications for pembrolizumab should follow those provided in the protocol.

1.5.4 Anticipated Risks of Cetuximab Combination Therapy

Cetuximab is commonly used for the treatment of extended RAS wild type CRC. Cetuximab has been associated with diarrhea, ILD, rash, hypomagnesemia, and serious infusion reactions. Sudden death has occurred in 2% of patients with head and neck cancer receiving cetuximab with radiation and in 3% of patients receiving cetuximab in combination with platinum-based

therapy. Because there were few important effects in nonclinical toxicology studies of LY3537982, there is a low risk of overlapping toxicity when combined with cetuximab. Monitoring and dose modifications for cetuximab should follow those provided in the product label or institutional guidelines.

1.5.5 Anticipated Risks of Pembrolizumab Plus Pemetrexed and Carboplatin or Cisplatin Chemotherapy

Detailed information about the known and expected benefits and risks of carboplatin, cisplatin, and pemetrexed may be found in the Patient Information Leaflet, Package Insert, or Summary of Product Characteristics. The anticipated risks of pembrolizumab are discussed in Section 1.5.3.

Because there were few important effects in nonclinical toxicology studies of LY3537982, there is a low risk of overlapping toxicity when combined with pembrolizumab plus pemetrexed and carboplatin or cisplatin chemotherapy.

1.6 Benefit/Risk

LY3537982 is a potent, mutant-selective small molecule covalent binder of KRAS G12C and has demonstrated significant activity in preclinical models with *KRAS* G12C mutation. LY3537982 administration to participants with *KRAS* G12C mutation is expected to provide clinical benefit.

Since LY3537982 is in an early stage of drug development, it is possible that unforeseen, unknown, or unanticipated drug reactions and toxicities may occur. However, the study protocol is designed to mitigate risks to participants through a detailed plan for cautious dose escalation, careful safety monitoring, systematic review of AEs and SAEs, PK evaluation, and active pharmacovigilance review to assess safety signals or trends.

Refer to the LY3537982 IB for additional information on the known and expected benefits, risks, and reasonably expected AEs of LY3537982.

Given the potential therapeutic benefit anticipated for LY3537982 administered as monotherapy and in combination with pembrolizumab, cetuximab, or pembrolizumab plus pemetrexed and carboplatin or cisplatin, as well as the known risk profile of these combination therapies and the benefit/risk assessment of LY3537982 monotherapy, the combination in the proposed study is considered acceptable.

More detailed information about the known and expected benefits and risks of cetuximab, pemetrexed, cisplatin, and carboplatin may be found in the Patient Information Leaflet, Package Insert, Summary of Product Characteristics, and IB.

More detailed information about the known and expected benefits and risks of pembrolizumab may be found in the IB, Pharmacy Manual, and Section 9.9, Appendix 9.

2.0 STUDY OBJECTIVES

2.1 Primary Objectives

Phase 1a

Dose Escalation (Part A)

- To determine the RP2D of LY3537982 monotherapy in participants with *KRAS* G12C-mutant advanced solid tumors, as assessed by:
  - DLTs
  - AEs and SAEs, changes in hematology and blood chemistry values, assessments of physical examinations, vital signs, and ECGs

Phase 1b

Dose Expansion (Parts B to E)

- To assess the safety and tolerability of LY3537982 when administered alone or in combination with other investigational agents in participants with advanced solid tumors with *KRAS* G12C mutation in the following subgroups:
  - Part B: NSCLC
  - Part C: CRC
  - Part D: Other solid tumors (except NSCLC, CRC, and pancreatic cancer)
  - Part E: NSCLC who have previously been treated with a KRAS G12C inhibitor

Dose Optimization (Part G)

- To determine the optimal dose of LY3537982 to be administered to treatment-naïve participants with advanced NSCLC in combination with pembrolizumab, as assessed by endpoints including, but not limited to TEAEs, SAEs, deaths, and clinical laboratory abnormalities

Dose Optimization (Part H)

- To determine the optimal dose of LY3537982 in combination with cetuximab to be administered to participants who have received at least one prior oxaliplatin- or irinotecan-containing regimen for advanced or metastatic CRC, as assessed by endpoints including, but not limited to TEAEs, SAEs, deaths, and clinical laboratory abnormalities

Phase 2

Part F

- To assess the antitumor activity of LY3537982 monotherapy in participants with advanced pancreatic cancer with *KRAS* G12C mutation, as assessed by ORR determined by IRC using RECIST v1.1

2.2 Secondary Objectives

- To assess the preliminary antitumor activity of LY3537982 when administered alone or in combination with other investigational agents in participants with advanced solid tumors with *KRAS* G12C mutation using RECIST v1.1 (as assessed by Investigator for Phase 1a and Phase 1b and as assessed by Investigator and IRC for Phase 2 [Part F]) based on evaluation of:
  - ORR
  - BOR
  - DOR
  - TTR
  - DCR
  - PFS
  - OS
  - Intracranial ORR based on modified RECIST v1.1 (Cohort B8 only)
  - Intracranial DOR based on modified RECIST v1.1 (Cohort B8 only)
- To characterize the PK properties of LY3537982 when administered alone or in combination with other investigational agents in participants with advanced solid tumors with *KRAS* G12C mutation
  - Plasma concentrations of LY3537982, pemetrexed, carboplatin, and cisplatin; serum concentrations of pembrolizumab (Cohort B4 only) and cetuximab

2.3 Exploratory Objectives

- To correlate PK/pharmacodynamics changes
- To correlate biomarker characteristics in tumor tissue or blood with clinical benefit (e.g., including, but not limited to, somatic genetic variants in the *STK11*, *KEAP1*, and *TP53* genes)
- To measure changes in biomarkers in response to study treatment and after progression

CCI

[redacted]

3.0 INVESTIGATIONAL PLAN

3.1 Study Design

This is an open-label, multicenter study of oral LY3537982 to evaluate safety and efficacy in participants with *KRAS* G12C-mutant advanced solid tumors who (depending on the cohort) have failed or are intolerant to standard of care or who have not yet received first-line treatment.

3.1.1 Schema for Phase 1a—Dose Escalation

In Phase 1a Part A (Dose Escalation), approximately 120 participants will receive LY3537982 monotherapy (Figure 1). The primary objective of Part A is to determine the RP2D of LY3537982 monotherapy ($RP2D_M$) in participants with *KRAS* G12C-mutant advanced solid tumors.

Figure 1: Phase 1a Dose Escalation Study Schema

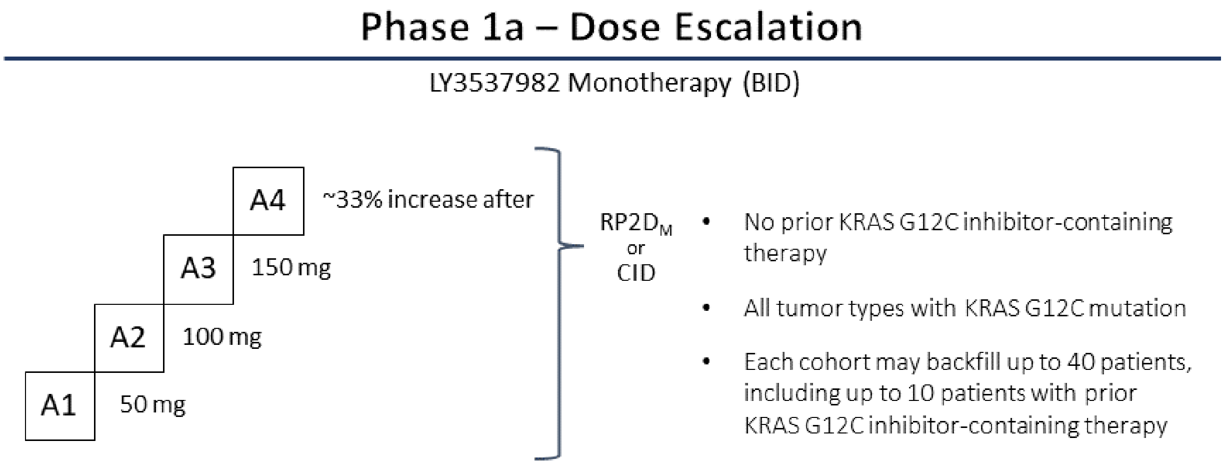

Note: The Combination Initial Dose (CID) is an LY3537982 dose level that may be defined by the Phase 1a SRC prior to $RP2D_M$ determination (see Section 3.1.6.1.1). The CID may be taken forward for safety lead-in cohorts in Part B and Part C.

3.1.2 Schema for Phase 1b—Dose Expansion

In Phase 1b, approximately 290 participants will be enrolled in Part B through Part E (Dose Expansion). During dose expansion, participants will receive either LY3537982 monotherapy or combination therapy, depending on the cohort (Figure 2). Dose expansion cohort sizes in Phase 1b are estimated, may decrease or increase in size, and will not exceed approximately 40 participants.

Figure 2: Phase 1b Dose Expansion Study Schema

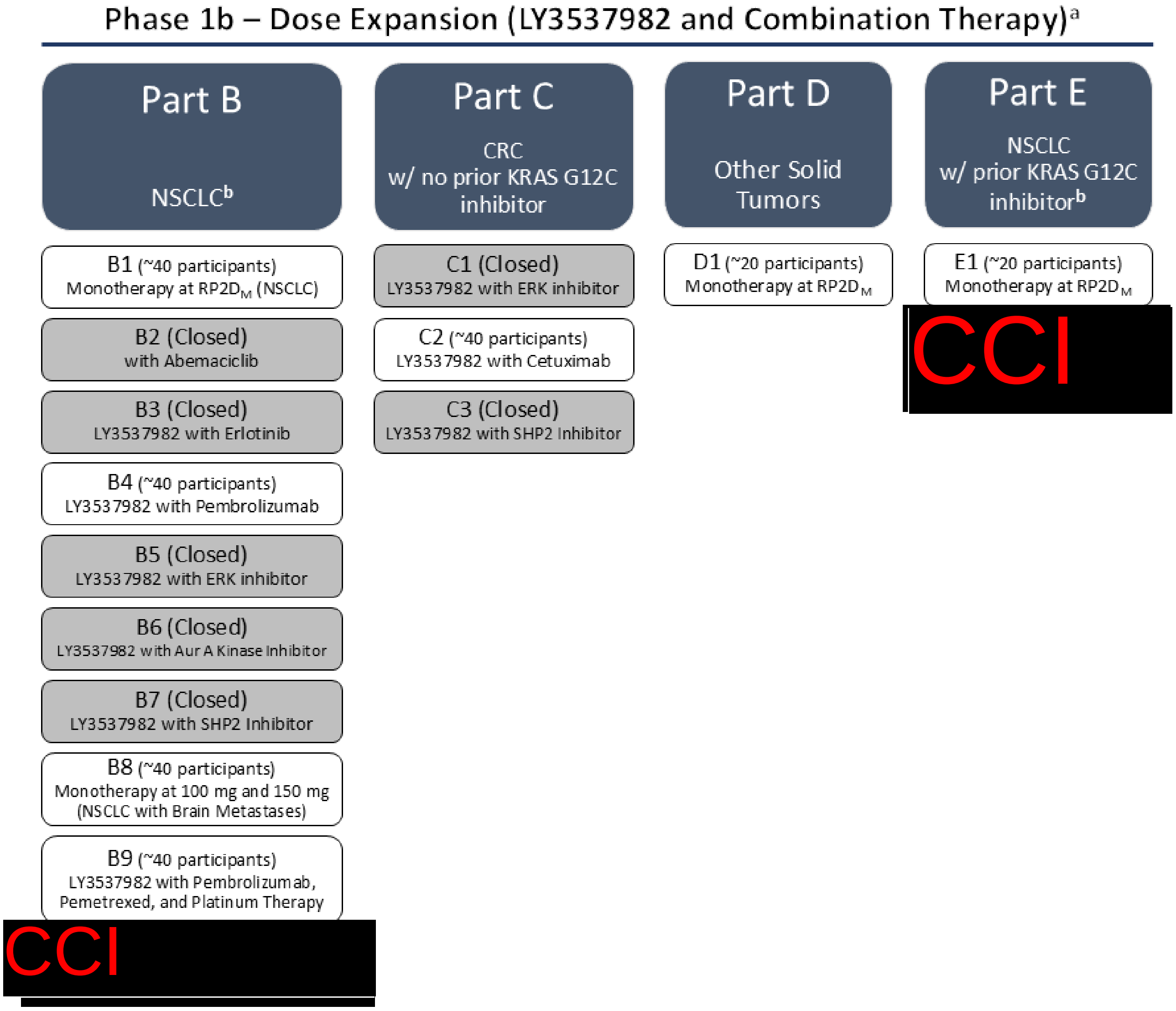

[a] Cohort sizes above (e.g., "~20 participants") indicate estimated enrollment at an established Combination Dose level after the safety lead-in period.

[b] Prior KRAS G12C inhibitor-containing therapy is allowed in Cohort B4 and Cohorts E1 [redacted].

The primary objective of dose expansion is to assess the safety and tolerability of LY3537982 when administered alone or in combination with other agents in participants with advanced solid tumors with a *KRAS* G12C mutation.

- LY3537982 monotherapy will be investigated in Cohort B1, [redacted] Cohort B8, Part D and Part E, and combination therapy will be investigated in all other Phase 1b cohorts.

- Combination therapy agents in dose expansion will include pembrolizumab (Cohort B4 and Cohort B9) and cetuximab (Cohort C2).
- Participants in Cohort B9 will also receive pemetrexed with either carboplatin or cisplatin and will be treatment naïve for advanced or metastatic NSCLC.
- Prior KRAS G12C therapy is only allowed in Phase 1b in Cohort B4 and Cohorts E1 [redacted].

3.1.3 Schema for Phase 1b—Dose Optimization (Part G and Part H)

Part G

In dose optimization Part G, approximately 40 treatment-naïve participants with *KRAS* G12C-mutant NSCLC will be randomized to receive pembrolizumab 200 mg Q3W with 2 different doses of LY3537982 (50 mg BID and 100 mg BID) (Figure 3).

Figure 3: Phase 1b Dose Optimization (Part G) Study Schema

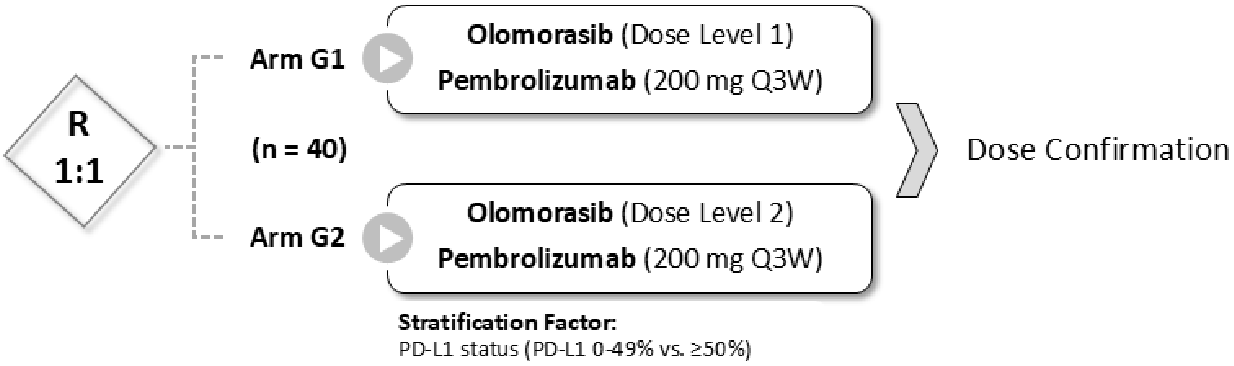

The primary objective of Part G is to optimize the dose of LY3537982 to be administered in combination with pembrolizumab to treatment-naïve participants. Participants will be stratified based on PD-L1 status (0% to 49% versus ≥ 50%). The LY3537982 dose levels in Arm G1 and Arm G2 (50 mg BID and 100 mg BID) were selected based on observed PK, safety, and preliminary efficacy data from this study. The Phase 1b SRC along with the study statistician will select the optimal dose and oversee safety aspects in Part G (with a focus on prespecified AEs). At any time, based on the totality of emerging data from participants receiving LY3537982 and pembrolizumab in either Cohort B4 or Part G, the SRC may declare the optimal dose. If the optimal dose is declared prior to full enrollment of Arm G1 and Arm G2, the remaining participants may be enrolled at the selected dose.

Part H

In dose optimization Part H, approximately 40 participants with *KRAS* G12C-mutant CRC will be randomized to receive either 100 mg BID or 150 mg BID of LY3537982 in combination with cetuximab (Figure 4).

Figure 4: Phase 1b Dose Optimization (Part H) Study Schema

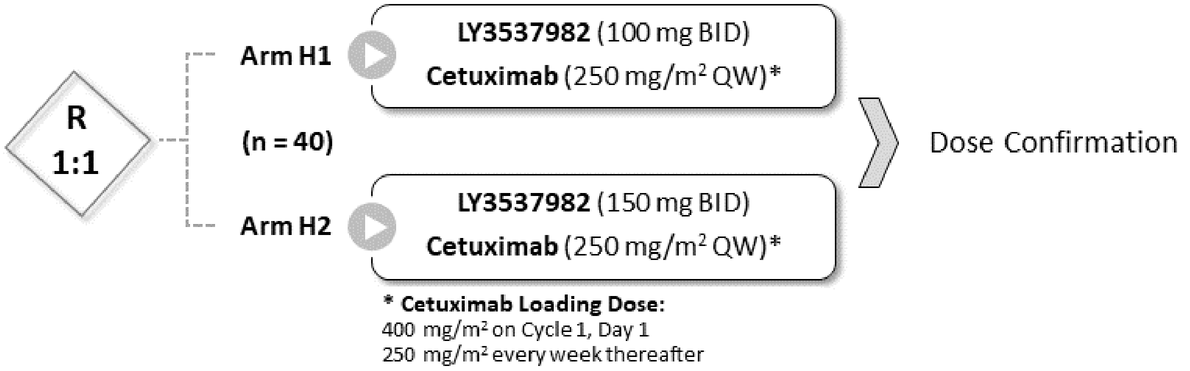

The primary objective of Part H is to optimize the dose of LY3537982 to be administered in combination with cetuximab to participants with advanced or metastatic CRC. The LY3537982 dose levels in Arm H1 and Arm H2 (namely, 100 mg BID and 150 mg BID) were selected based on observed PK, safety, and preliminary efficacy data from this study. The Phase 1b SRC along with the study statistician will select the optimal dose and oversee safety aspects in Part H. At any time, based on the totality of emerging data from participants receiving LY3537982 and cetuximab in either Cohort C2 or Part H, the SRC may declare the optimal dose. If the optimal dose is declared prior to full enrollment of Arm H1 and Arm H2, the remaining participants may be enrolled at the selected dose.

3.1.4 Other Aspects of Study Design

In all phases, treatment cycles will be 21 days. The planned duration of LY3537982 treatment is not fixed; participants will remain on study until disease progression or unacceptable toxicity occurs or the participant withdraws consent. Pembrolizumab treatment will conclude after 35 cycles. If participants are discontinued from a combination agent, they will be allowed to continue study treatment (LY3537982) if receiving ongoing clinical benefit. Additionally, if participants are discontinued from study treatment (LY3537982), they will be allowed to continue therapy with the respective combination drug per the Investigator's discretion, unless otherwise indicated.

KRAS G12C mutations will be identified through standard of care testing as routinely performed at each participating site utilizing material collected prior to participant consent to this protocol. Molecular assays utilized for enrollment are required to be performed in CLIA, ISO/IEC, CAP, or similarly certified laboratory as per local guidelines including, but not limited to, IVDR compliance as applicable. De-identified primary pathology report(s) describing the *KRAS* G12C mutation (and other detected alterations as appropriate based on assay) should be submitted during/prior to eligibility. For Part G, participants without local results for *KRAS* may submit tumor tissue for testing of *KRAS* to determine eligibility.

In Part G, PD-L1 status will be determined through standard of care testing as routinely performed at each participating site utilizing material collected prior to participant consent to this protocol. Results must be from an IHC assay performed in CLIA, ISO/IEC, CAP, or similarly certified laboratory as per local guidelines including, but not limited to, IVDR compliance as applicable. De-identified primary pathology report(s) describing PD-L1 status should be submitted during/prior to randomization. Participants without local results for PD-L1 may submit tumor tissue for testing of PD-L1 to determine PD-L1 status.

LY3537982 will be provided as an oral solid dosage form and will be provided to the sites for distribution to the participant for outpatient administration at the assigned DL. Dosing will be fixed (i.e., not weight-based or BSA-based).

Dose escalation, dose expansion, and dose optimization phases will consist of:

- Screening period
- Treatment period
- EOT visit
- SFU visit
- LTFU

3.1.5 Details of Phase 1a Dose Escalation

Phase 1a dose escalation will assess the safety and tolerability of LY3537982 to identify a $RP2D_M$ in participants with advanced solid tumors with a *KRAS* G12C mutation.

Dose escalation decisions will be based on any DLT events occurring during a 21-day DLT observation period and will be driven by the mTPI-2 method (Section 3.1.5.1). After 3 to 6 participants have completed the DLT period (Section 3.1.5.3), the Sponsor and the Investigators will hold a meeting to review safety and available PK results from evaluable participants to ensure it is safe to proceed with the next cohort and to determine the next DL and schedule. AEs from nonevaluable participants will also be reviewed throughout the dose escalation process in Phase 1a.

Safety data, in particular DLTs, will be the primary criteria for the dose escalation. In addition, if available at the time of the dose escalation decision, PK results (e.g., C_{max}, AUC, and pharmacodynamics results) will be used as secondary/supporting data for dose escalation.

Once a given DL is cleared for initial DLT evaluation, and if the PK supports a wider therapeutic index, more participants may be enrolled (i.e., backfilled; see Section 3.1.5.2) to that DL while a higher DL is being evaluated for DLT. Participants may be enrolled at each DL (inclusive of 3 to 6 dose escalation slots plus backfill slots, provided backfill criteria are met), with a total of approximately 120 participants enrolled in Phase 1a. If supported by the SRC, backfill slots may

be moved between dose levels in a total participant number-neutral manner to allow further exploration of select dose levels.

Any AEs reported by participants backfilled into DLs previously deemed safe by the SRC that would otherwise have met DLT criteria (see Section 3.1.5.3) will be considered DLT-equivalent toxicities. To ensure that the aggressive DLT and DLT-equivalent toxicity rate does not exceed safe levels, a Bayesian toxicity monitoring will be implemented for DLT and DLT-equivalent toxicities added from backfill participants (Section 8.5). In general, the SRC will utilize all available toxicity and PK data, including DLT-equivalents observed from backfill participants, to recommend escalation/de-escalation decisions. DLT evaluation at a higher DL could be halted, and additional participants could be enrolled to a DL that has been cleared for initial DLT evaluation (i.e., stay) or to a lower DL (i.e., de-escalation), depending on the total number of DLTs observed among all DLT-evaluable participants at that DL (initial and backfilled participants).

Recommended Phase 2 Dose Determination

The LY3537982 $RP2D_M$ of CCI [redacted] was determined based on aggregate analysis of the number of observed DLTs, PK, safety, tolerability, and clinical activity. No MTD for LY3537982 monotherapy was determined.

3.1.5.1 Phase 1a Dose Escalation Method

Dose escalation of LY3537982 will be driven by the mTPI-2 method (Yan et al. 2017; Guo et al. 2017) where a precalculated decision table (Figure 5) will guide the dose recommendations until the MTD is determined. Operating characteristics are given in Section 9.8, Appendix 8. The pT of the MTD is set to CCI [redacted] which gives the precalculated decision rules below.

Although mTPI-2 allows a flexible number of participants in each dosing cohort, a minimum of 3 evaluable participants are required for the DLT evaluation at each DL. No more than 6 participants will be enrolled at the current DL, excluding previously enrolled participants if a DL is revisited. The decision table (Figure 5) includes decision rules for cohorts of size N = 1 and N = 2; these columns are included for completeness and to match the standard presentation in academic papers and other protocols.

Though the mTPI-2 method incorporates prespecified escalation rules like the 3 + 3 design, mTPI-2 uses quantitative models to incorporate uncertainty into the decision rules, allowing for a more precise selection of MTD. If 3 or 6 participants are enrolled in a cohort, the escalation rule parallels a traditional 3 + 3 design. For example, with 2 DLTs per 6 participants enrolled, the mTPI-2 would recommend staying at the current dose, analogous to 1 DLT per 3 participants enrolled. If 4 or 5 participants are enrolled, the mTPI-2 algorithm will provide decision rules while 3 + 3 cannot provide decision rules for cohorts of this size.

The mTPI-2 design incorporates safety rules as added ethical constraints. Specifically, if the current dose is considered excessively toxic, then the current dose and all higher doses are excluded and never used again. Furthermore, if the current dose is the lowest dose, and that dose is considered to be excessively toxic, then the escalation may be terminated due to safety. Alternatively, additional DLs and/or schedules below the lowest dose may be introduced. For these added safety constraints, excessive toxicity at a given DL is defined as Prob (DLT rate > [redacted] |data) > [redacted], where the Sponsor assumes DLTs follow a beta-binomial model with a [redacted] prior on the DLT rate.

Figure 5: Decision Rules of Modified Toxicity Probability Interval-2 (mTPI-2) Method

[redacted]

Abbreviations: D = de-escalate to the previous lower dose; DU = de-escalate to the previous lower dose and deem the current dose unacceptably toxic, not to be used again in the study; E = escalate to the next higher dose; S = stay at the current dose, and the current dose has enrolled 6 participants.

The x-axis represents the number of participants enrolled at the dose and the y-axis represents the number of DLTs at the same dose.

Dose escalation decisions are guided by the mTPI-2 design with the targeted DLT rate = [redacted]. Figure 5 lists the decisions for up to 20 participants treated at a given dose.

Dose escalation will proceed until the mTPI-2 decision is to:

- S = stay at the current dose, and the current dose has enrolled 6 participants,
- E = escalate to the next higher dose, and the next higher dose has enrolled 6 participants,
- D = de-escalate to the previous lower dose, and that previous lower dose has enrolled 6 participants.

However, the SRC may decide to exceed 6 participants at an mTPI-2 recommended dose in order to gather additional data pertaining to PK and safety. The final MTD will be the dose for which the estimated isotonically transformed posterior means of DL DLT rates are closest to the pT of CCI [redacted].

Following a discussion between the Sponsor and the Investigators, a more conservative dose selection may be applied to the next cohort (for instance, if PK/pharmacodynamics data suggest that further dose increase would not be expected to yield additional benefit). For example, if the rule indicates "E" to escalate, the dose may stay at the current DL, be de-escalated to a lower level, or escalation may cease.

Decisions to dose escalate will be made on any observed DLTs as well as additional supportive data such as overall safety profile, PK/pharmacodynamics, and observed efficacy. The SRC may elect to pursue intermediate, lower, previously tested, or higher DLs based on overall review of safety, PK/pharmacodynamics, efficacy, pill burden, and other factors. The SRC may also propose that dose escalation may not proceed to the highest specified DLs even in the absence of DLTs or until formal identification of an MTD. Alternative dosing schedules (e.g., QD instead of BID dosing) may also be considered if the PK/pharmacodynamics profile does not support the necessity of BID dosing. After completion of the 21-day DLT period, intraparticipant dose escalation to the highest DL deemed safe by the SRC will be permitted following approval by the Sponsor.

The decision to stop dose escalation will be primarily driven by the occurrence of DLTs and the rules in Figure 5. However, if PK data suggest that increasing the dose further is not expected to yield additional benefit, escalation may cease.

The totality of data including safety, PK exposure, and the incidence of DLTs may help guide evaluating alternative dosing and schedule (e.g., 100 mg QD versus 50 mg BID).

3.1.5.2 Backfill Criteria

During Phase 1a dose escalation, selected DLs may be further expanded (also known as backfilling) to include up to approximately 40 participants treated at a given dose in order to further evaluate tolerability, PK/pharmacodynamics, and biological activity of LY3537982. To ensure that participants are not unnecessarily enrolled to subtherapeutic DLs, these backfills will not be undertaken until a DL already deemed safe by the SRC also demonstrates evidence of therapeutically relevant exposures or direct evidence of clinical activity. The cumulative safety data from the cohorts previously deemed safe (backfilled), and the current dose cohort will be continuously monitored. Bayesian toxicity monitoring will be implemented for DLT and DLT-equivalent toxicities added from backfill participants (Section 8.5).

A total of approximately 10 NSCLC participants with previous KRAS G12C inhibitor-containing therapy treatment may be enrolled into each backfill. This limitation on the

enrollment of participants with previous KRAS G12C inhibitor-containing therapy may be lifted, if supported by SRC and Sponsor based on emerging clinical data.

If slots are available in both backfill cohorts as well as the current dose escalation cohort simultaneously, enrollment to the dose escalation cohort will be prioritized.

Escalation in Phase 1a will proceed through planned LY3537982 DLs (as outlined in Figure 1). Phase 1b dose expansion and dose optimization may occur in parallel with ongoing backfill of Phase 1a.

3.1.5.3 Dose-Limiting Toxicity Determination for Phase 1a

To be eligible for the assessment of a DLT, each participant in each monotherapy dose escalation in Phase 1a or combination safety lead-in cohort in Phase 1b (Part B and Part C only; see Section 3.1.6.1.1) must have completed safety assessments through the first 21 days of study drug. Participants must have received a minimum of 75% of the planned total doses of LY3537982 and any combination agent(s) during that time. Participants who receive less than 75% of planned doses due to reasons other than a DLT will be replaced for the determination of the MTD but will remain part of the safety population if they receive at least 1 dose of study drug. Any toxicity resulting in treatment with less than 75% of planned doses will be reviewed by the SRC and will be considered a DLT if the SRC determines the toxicity is not related to the participant's underlying disease, other medical condition, or concomitant medications.

A DLT is defined as any of the TEAEs listed below (Table 2); a TEAE is defined as an AE that starts or worsens on or after the first dose of study drug, as defined by the NCI CTCAE v5.0, that is deemed clinically significant and occurs during the first 21 days of study drug administration, unless the TEAE can be clearly related to the participant's underlying disease, other medical condition, or concomitant medications. For DLT definitions for Cohort B4, Cohort B9, and Part G, see Section 9.9.1.

Table 2: DLT Definitions for Phase 1a and Phase 1b Combination Therapy Cohorts Except Cohort B4, Cohort B9, and Part G

**Hematologic Toxicity**
• Any Grade 3 thrombocytopenia associated with clinically significant bleeding and/or requiring platelet transfusion or Grade 4 thrombocytopenia of any duration<br>• Grade ≥ 3 febrile neutropenia and/or neutropenia requiring G-CSF<br>• Grade ≥ 3 anemia requiring blood transfusion, except where the hemoglobin decline represents a clinically insignificant decline from baseline (e.g., ≤ 1.5 g/dL)<br>• Grade 4 neutropenia or leukopenia of > 7 days duration<br>• Other Grade ≥ 4 hematologic toxicity not otherwise specified above
**Nonhematologic Toxicity Nonlaboratory**
• Any Grade ≥ 3 toxicities, except the following:<br>○ Grade 3 fatigue, nausea, vomiting, diarrhea, or constipation that lasts for < 72 hours and can be controlled with treatment and does not require hospitalization

**Nonhematologic Toxicities – Laboratory/investigations**
• Grade ≥ 3 laboratory abnormalities lasting > 7 days or requiring medical intervention except the following:<br>○ Grade ≥ 3 electrolyte abnormalities that last up to 72 hours, are not clinically complicated, and resolve spontaneously or respond to conventional medical interventions<br>• Participants with normal ALT or AST at baseline:<br>○ ALT or AST ≥ 5× ULN<br>○ ALT or AST ≥ 3× ULN and TBL ≥ 2× ULN in the absence of significant cholestasis (i.e., ALP > 2× ULN) and in the absence of a clear cause of hepatic injury other than the study drug.<br>• Participants with elevated ALT or AST at baseline:<br>○ ALT or AST ≥ 5× baseline[a] or 8× ULN, whatever is lower<br>○ ALT or AST ≥ 2× baseline[a] and TBL ≥ 2× ULN in the absence of significant cholestasis (i.e., ALP > 2× ULN) and in the absence of a clear cause of hepatic injury other than the study drug.

Any toxicity, regardless of the NCI CTCAE v5.0 grade, resulting in discontinuation, dose reduction, or treatment with < 75% of planned doses of LY3537982 and any combination agent(s), or delay of ≥ 14 days to start the next cycle, will be reviewed by the SRC and will be considered a DLT if the SRC determines the toxicity is not clearly related to the participant's underlying disease, other medical condition, or concomitant medications.

The DLT definitions in the table above apply to combination therapies in Part B and Part C, except those involving pembrolizumab in Cohort B4 and Cohort B9, with the exception of AEs that can clearly be attributed by the Investigator to the combination agent alone.

[a] Multiples of baseline pertain to the aminotransferase (ALT or AST) which is elevated at baseline.

c

Investigators, together with the Sponsor, can declare a DLT if a participant is experiencing increasing toxicity during treatment, and it becomes clear that it is not going to be possible to complete the treatment without exposing the participant to excessive risk.

A DLT-equivalent is defined as an AE that would have met the criteria for a DLT if it had occurred during Cycle 1 for a participant enrolled in the dose escalation phase but that occurs any time after Cycle 1. DLT-equivalent toxicities may be considered in determining the RP2D.

3.1.6 Details of Phase 1b Dose Expansion and Dose Optimization

Details of dose expansion and dose optimization study treatment are provided for each cohort in Phase 1b (Table 3).

In Phase 1b, all monotherapy cohorts except for some participants in Cohort B8, [redacted] will begin at the $RP2D_M$ as shown in Table 3. All combination therapy cohorts will begin as safety lead-in cohorts with LY3537982 doses one level below $RP2D_M$ ($RP2D_{M-1}$), or at the CID, with combination therapy dose(s) at the levels shown in Table 3.

All cohorts may not be opened simultaneously or in all regions. Enrollment to each cohort will be as per treating physician's and participant choice and pending cohort availability.

Table 3: Phase 1b Dose Expansion and Dose Optimization Cohorts

Study Part	Dose Expansion Cohort	Study Drug(s) (Route of Administration)	Proposed Initial Doses[a] (Frequency)
**Part B:** *KRAS* G12C-mutant advanced NSCLC	B1[b]	LY3537982 (PO)	RP2D$_{M}$ (BID)
	B2 (closed)	LY3537982 (PO)	Not applicable
		LY2835219 (abemaciclib) (PO)	Not applicable
	B3 (closed)	LY3537982 (PO)	Not applicable
		Erlotinib (PO)	Not applicable
	B4	LY3537982 (PO)	RP2D$_{M-1}$ or CID (BID)
		Pembrolizumab (IV)	200 mg (Q3W)
	B5 (closed)	LY3537982 (PO)	Not applicable
		LY3214996 (ERK inhibitor) (PO)	Not applicable
	B6 (closed)	LY3537982 (PO)	Not applicable
		LY3295668 (Aurora A kinase inhibitor) (PO)	Not applicable
	B7 (closed)	LY3537982 (PO)	Not applicable
		TNO155 (SHP2 inhibitor) (PO)	Not applicable
	B8[b]	LY3537982 (PO)	100 mg (BID), RP2D$_{M}$ (BID)
	B9	LY3537982 (PO)	RP2D$_{M-1}$ or CID (BID)
		Pembrolizumab (IV)<br>Pemetrexed (IV)<br>and<br>Cisplatin (IV)<br>OR<br>Carboplatin (IV)	200 mg (Q3W)<br>500 mg/m^2 (Q3W)<br>and<br>75 mg/m^2 (Q3W)<br>OR<br>AUC 5 mg/ml/min (Q3W)<br>(max dose = 750 mg)
	[redacted]	[redacted]	CCI [redacted]

**Study Part**	**Dose Expansion Cohort**	**Study Drug(s) (Route of Administration)**	**Proposed Initial Doses[a] (Frequency)**
**Part C:** *KRAS* G12C-mutant advanced CRC	C1 (Closed)	LY3537982 (PO)	Not applicable
		LY3214996 (ERK inhibitor) (PO)	Not applicable
	C2	LY3537982 (PO)	$RP2D_{M-1}$ or CID (BID)
		Cetuximab (IV)	400 mg/m^2 loading dose C1D1; 250 mg/m^2 every week thereafter (QW)
	C3 (Closed)	LY3537982 (PO)	Not applicable
		TNO155 (SHP2 inhibitor) (PO)	Not applicable
**Part D:** *KRAS* G12C-mutant advanced solid tumors (except NSCLC, pancreatic cancer, and CRC)	D1	LY3537982 (PO)	$RP2D_M$ (BID)
**Part E:** *KRAS* G12C-mutant advanced NSCLC who have previously been treated with a KRAS G12C inhibitor	E1[d]	LY3537982 (PO)	$RP2D_M$ (BID)
	[redacted]	[redacted]	[redacted]
**Part G:** Previously untreated *KRAS* G12C-mutant advanced NSCLC	Arm G1	LY3537982 (PO)	50 mg BID
		Pembrolizumab (IV)	200 mg (Q3W)
	Arm G2	LY3537982 (PO)	100 mg BID
		Pembrolizumab (IV)	200 mg (Q3W)
**Part H:** *KRAS* G12C-mutant advanced CRC	Arm H1	LY3537982 (PO)	100 mg BID
		Cetuximab (IV)	400 mg/m^2 loading dose C1D1; 250 mg/m^2 every week thereafter (QW)
	Arm H2	LY3537982 (PO)	150 mg BID
		Cetuximab (IV)	400 mg/m^2 loading dose C1D1; 250 mg/m^2 every week thereafter (QW)

[a] For combination cohorts in Part B and Part C, the LY3537982 doses shown are those allowed in the first safety lead-in cohort. The dose taken forward in each cohort after the safety lead-in period may differ (see Section 3.1.6.1.1). Alternative dosing schedules (i.e., QD) may be considered by the Phase 1b SRC for all agents except pembrolizumab, pemetrexed, carboplatin, and cisplatin, based upon the totality of safety, PK/pharmacodynamics, and efficacy data.

[b] Cohort B1 will enroll participants with *KRAS* G12C-mutant advanced NSCLC, including those with treated, stable brain metastases; Cohort B8 will enroll participants with *KRAS* G12C-mutant advanced NSCLC with untreated, active brain metastases.

[c] Participants in Cohort B8, [redacted] may dose escalate with Sponsor approval under certain conditions; see Section 7.11.

[d] Will only be opened if activity observed in Phase 1a.

3.1.6.1 Safety Lead-In Cohorts in Phase 1b (Part B and Part C)

All combination therapies investigated in the dose expansion cohorts in Part B and Part C will include a safety lead-in cohort of approximately 6 participants, which will be assigned an initial LY3537982 dose and combination agent dose(s) (Section 3.1.6.1.1). Safety lead-in cohorts will be dose-escalated or -reduced consistent with mTPI-2 rules (Section 3.1.6.1.2) based on DLTs observed in the first cycle (Section 3.1.6.1.3). If the combination is deemed intolerable, the LY3537982 dose and/or the combination agent may be reduced by 1 level or more. Pembrolizumab will not be dose reduced.

3.1.6.1.1 Initial Dosing

Safety lead-in cohorts in Phase 1b will generally not open until the RP2D$_M$ is established in Phase 1a. The initial LY3537982 dose in a combination cohort may also be the Combination Initial Dose (CID). The CID is a LY3537982 dose level that may be defined for a particular cohort at any time, if agreed upon by the Sponsor and SRC, if it is at least one DL below the highest level previously deemed safe by the Phase 1b SRC and associated with exposures predicted to be efficacious. The CID may be taken forward as a safety lead-in dose in Part B and Part C before or after a formal declaration of RP2D$_M$, in order to permit preliminary evaluation of the safety and feasibility of the particular combination therapy.

After the safety lead-in period has established 1 or more Combination Doses, combination cohorts in Part B and Part C may enroll additional participants at any Combination Dose of LY3537982 following a discussion between the Sponsor and Investigators. Up to approximately 40 participants may be enrolled at a Combination Dose level (including any safety lead-in participants treated at that dose), except for Cohort CCI, which may enroll up to approximately 30 participants, to further investigate the tolerability, PK, and biological activity of the LY3537982 combination. For instance, the combination cohorts that utilized the CID as the initial safety lead-in dose of LY3537982 may enroll additional participants at that dose level, if it is cleared as a Combination Dose, or other cleared dose levels (e.g., CID$_{+1}$ or CID$_{-1}$) or the RP2D$_M$ once determined, if tolerated according to the DLT rules in Section 3.1.6.1.3.

In the event that any cohort is on hold for safety or other reasons, new participants will be enrolled into remaining available cohorts. Safety will be continually monitored by Bayesian toxicity modeling (Section 8.5).

At any point in Phase 1b, based on emerging safety, intermediate doses of LY3537982 may be explored, and, if necessary, alternative doses and/or schedules of LY3537982 in combination with other investigational agents may be explored. Similarly, doses below the currently approved doses and/or alternative schedules of the other agents may be explored (except that pembrolizumab will not be dose reduced).

In addition, during evaluation of emerging safety data from participants enrolled at any time (including during safety lead-in) in Cohort B9, the Sponsor, with agreement from the SRC, may

explore any of the following changes to Cohort B9 dosing administration, if significant toxicity is detected and the risk-benefit profile of the combination has changed:

- dose reductions or removal of pemetrexed
- dose reductions of either carboplatin or cisplatin
- delayed administration of LY3537982 until after 1 or more cycles of standard of care treatment including administration of any combination of the KEYNOTE-189 treatment regimen (i.e., pembrolizumab, pemetrexed, carboplatin/cisplatin). If LY3537982 is delayed, study treatment should start approximately 21 days after Day 1 of the prior cycle of therapy (or as close as possible after this date). Start of study treatment with LY3537982 may be delayed for a maximum of 42 days from Day 1 of the prior cycle of therapy to allow sufficient time for recovery from treatment-related toxicity.

Pembrolizumab administration will remain unchanged and will not be dose reduced.

3.1.6.1.2 Dose Escalation Method

Dosing decisions in the safety lead-in cohorts are consistent with mTPI-2 dose escalation (Section 3.1.5). Initially, approximately 6 participants will be enrolled into a safety lead-in cohort for each combination therapy in Part B and Part C and treated at the LY3537982 $RP2D_{M-1}$ or CID in combination with other investigational agents (Table 4).

Table 4: Safety Lead-in Combination Dosing – Part B and Part C

	LY3537982 Dose	Combination Agent Dose
**Safety Lead-in Dose**	No greater than $RP2D_{M-1}$ or CID	See the Table 3 dose
**Combination Dose(s)**[a]	$RP2D_M$ or CID_{+1} up to $RP2D_M$	See the Table 3 dose
**Reduction 1**	Dose **MUST** be reduced: $RP2D_{M-2}$ or CID_{-1}	Dose **MAY** be reduced[b]
**Reduction 2**	Dose **MUST** be further reduced: $RP2D_{M-3}$ or CID_{-2}[c]	Dose **MAY** be reduced[b]

[a] If the safety lead-in dose plus combination agent is safe, the Sponsor may open an additional safety lead-in cohort at a lower or higher dose of LY3537982 to see if that dose of LY3537982 plus combination agent is tolerable. The same DLT and dose reduction rules will apply to these cohorts.

[b] Except for pembrolizumab, which will not be dose reduced.

[c] In combination cohorts where a DLT is considered potentially related to the combination agent(s), then either the LY3537982 dose or the combination agent(s) dose may be reduced (except for pembrolizumab, which will not be dose reduced)

These safety lead-in participants will complete a 21-day DLT evaluation period before additional participants can be enrolled into the cohorts. If the number of DLTs is consistent with an mTPI-2 decision to stay at the current dose or escalate CCI [redacted] continued enrollment may be allowed into the cohort at that dose level. Further, if the LY3537982 $RP2D_{M-1}$

(or CID) and dose level of combination agent(s) specified in Table 3 is considered tolerable following a review by the Sponsor, and the number of observed DLTs is consistent with an mTPI-2 recommendation to escalate, the Sponsor may open a safety lead-in cohort of additional participants at a higher dose (e.g., $RP2D_M$ or CID_{+1}) of LY3537982 with the same dose level of combination agent.

If the number of DLTs observed in the first safety lead-in cohort of participants is consistent with an mTPI-2 decision to de-escalate CCI [redacted] the LY3537982 dose will be reduced by 1 level (CID_{-1} or $RP2D_{M-2}$) or more and the combination agent(s) may be reduced by 1 level or more, and 3 more participants will be enrolled in a new safety lead-in cohort and treated at the reduced DLs. Pembrolizumab will not be dose reduced. Further enrollment into the cohort will be paused for all participants to clear the DLT evaluation period. Following a discussion between the Sponsor and the study Investigators, an additional 3 participants may be enrolled to the reduced dose level(s) to further evaluate safety and efficacy.

If the participants in a combination cohort receiving treatment at a de-escalated (reduced) dose level complete the 21-day DLT evaluation period, and if the number of participants with DLTs is consistent with an mTPI-2 decision to re-escalate, the Sponsor may open 1 or more safety lead-in cohorts in a parallel or staggered fashion of additional participants at the next higher or intermediate dose of LY3537982 with the same or reduced dose level(s) of combination agent(s) specified in Table 3. The specific dosing regimen(s) of LY3537982 and combination agent(s) may include delays in administration of LY3537982 or other changes related to toxicity management and must be approved by the SRC after a review of the totality of relevant safety and PK data (if available) in that cohort.

If the first reduced dose is considered intolerable and/or the number of DLTs at this reduced DL is again consistent with an mTPI-2 decision to de-escalate CCI [redacted] enrollment into the reduced-dose safety lead-in cohort will be paused to conduct a review of all available toxicity, safety, and PK data. Following this review, a new safety lead-in cohort of approximately 3 participants may be treated at a further reduced dose of LY3537982 (CID_{-2} or $RP2D_{M-3}$ or lower) and the combination agent(s) (if the toxicity is considered potentially related to the combination agent[s]), if such a dose can be identified by the SRC/Sponsor. Note that pembrolizumab will not be dose reduced. If no suitable reduced dose can be identified, enrollment into this cohort will end.

Following a review of safety, efficacy, and PK data, the Sponsor may apply a more conservative rule during the safety lead-in than specified by mTPI-2. For example, the LY3537982 dose and/or the combination agent dose(s) may also be reduced if CCI [redacted] are observed (see Section 3.1.5.3 for the determination of DLT and other details). Whenever a dose de-escalation occurs during the safety lead-in, all participants that experienced DLTs and all subsequently enrolled participants will have their dose reduced to this new DL.

Based on emerging safety data, it is possible that the $RP2D_M$ of LY3537982 may be altered. If the $RP2D_M$ is changed, participants enrolled to the expansion phase at a different dose may have

the dose changed to the new dose. Finally, participants treated during dose escalation at the dose chosen for further study, who also meet the criteria for one of the monotherapy expansion cohorts, may be considered as part of the evaluable participants for that cohort.

3.1.6.1.3 Dose-Limiting Toxicity Determination for Phase 1b

DLTs are defined for Cohort B4, Cohort B9, and Part G in Section 9.9, Appendix 9. For all other cohorts, DLT definitions for Phase 1a monotherapy (see Section 3.1.5.3) apply to Phase 1b combination therapy, with the exception of TEAEs that can clearly be attributed by the Investigator to the combination agent alone. These cases will be reviewed by the SRC/Sponsor to determine whether LY3537982 may have contributed to the observed toxicity, in which case the TEAE would also be considered a DLT.

3.1.7 Details of Phase 2

In Phase 2, approximately [redacted] participants with pancreatic cancer will be enrolled in Cohort F1 of Part F and will receive LY3537982 monotherapy at the $RP2D_M$ (Table 5). The primary objective of Part F is to assess the antitumor activity of LY3537982 by determining ORR (as assessed by IRC) using RECIST v1.1. DLT definitions for Phase 1a monotherapy (see Section 3.1.5.3) apply to Phase 2.

Table 5: Phase 2 Cohort

Study Part	Cohort	Study Drug(s) (Route of Administration)	Proposed Initial Doses[a] (Frequency)
**Part F:** *KRAS* G12C-mutant pancreatic cancer	F1	LY3537982 (PO)	$RP2D_M$ (BID)

3.2 Dose Scientific Rationale for Study Design

The overall rationale for the study design is described in the introduction section under Study Rationale (Section 1.1) and in the Statistical Considerations (Section 8.0). Dose selection details can be found in Section 1.3.3.

3.3 End of Study Definition

Study completion will occur following the final analysis of primary and secondary objectives, as determined by Sponsor. Study completion does **not** include continued access.

End of Study is defined as the date of the last visit of the last participant in the study globally, inclusive of continued access.

4.0 SELECTION OF STUDY POPULATION

Potential participants must sign an ICF before any study-specific screening tests may be conducted.

4.1 Inclusion Criteria

Individuals are eligible to be included in the study only if **all** of the following criteria apply:

1) Individuals must be able to provide consent consistent with local regulations and be ≥ 18 years of age at the time of signing the informed consent form.
2) Individuals must have measurable disease per RECIST v1.1.
3) Individuals must have disease with evidence of *KRAS* G12C mutation in tumor tissue or circulating tumor DNA as determined by molecular testing performed at a CLIA, ISO/IEC, CAP, or other similar certified laboratory as per local guidelines including, but not limited to, IVDR compliance as applicable. In cohorts where individuals with NSCLC who have progressed on a prior KRAS G12C inhibitor are allowed to enroll, the individuals must have *KRAS* G12C mutation confirmed in a blood or tumor tissue sample collected within approximately 3 months preceding discontinuation of prior KRAS G12C inhibitor due to disease progression or at any time thereafter.
4) Individuals must have a histological or a cytologically proven diagnosis of locally advanced, unresectable, and/or metastatic cancer and meet cohort-specific criteria:

   a) Phase 1a Dose Escalation:

   - Individuals must be appropriate candidates for study treatment. Study treatment is considered appropriate where approved measures are no longer effective or are not considered appropriate or safe in the opinion of the Investigator. If the available standard therapy is not considered appropriate or safe in the opinion of the Investigator, the rationale for ineligibility shall be provided and documented in the CRF. Furthermore, in the opinion of the Investigator, if the individual is intolerant to standard therapy, drug information, toxicity, and grade must be documented in the CRF.

   b) Phase 1b Dose Expansion Part B (NSCLC):

   - Individuals must not have a known targetable oncogenic driver mutation or alteration in genes such as *EGFR*, *ALK*, *BRAF* (V600E), *MET* (exon 14), *ROS1*, *RET,* or *NTRK1/2/3*. Does not apply to individuals in Cohort B4.

     Cohort B9 Only Specific Criteria:

     - Histologically or cytologically confirmed Stage IIIB-IIIC or Stage IV NSCLC that is previously untreated in the advanced/metastatic setting and not suitable for curative intent radical surgery or radiation therapy. Staging will be according to the AJCC Staging System (8th ed [Amin et al. 2017]). The histology of the tumor must be predominantly nonsquamous. Squamous cell and/or mixed small cell/non-small cell histology is not permitted.

- Individuals may have received up to one 21-day cycle of any combination of the KEYNOTE-189 treatment regimen (i.e., pembrolizumab, pemetrexed, carboplatin/cisplatin), at or below the dose levels defined for Cohort B9 in Table 7, Section 5.1 of the protocol; such therapy must have been initiated within 21 days (+14 days) prior to enrollment. Start of study treatment may be delayed for a maximum of 42 days from day 1 of the prior cycle of therapy to allow sufficient time for recovery from treatment-related toxicity.

CCI [redacted]

- Individuals should only be enrolled in Cohort B9 if, in the Investigator's judgment, the individual is an appropriate candidate to initiate their planned treatment regimen at the dose levels specified in each combination agent's Package Insert or Summary of Product Characteristics. Individuals entering Cohort B9 in the maintenance setting need not be appropriate to initiate treatment with carboplatin or cisplatin.

Remaining Part B Cohorts

- Individuals must be appropriate candidates for study treatment, after progressing on, being intolerant to, or ineligible for immunotherapy and platinum-based therapy. Study treatment is considered appropriate where approved measures are no longer effective or are not considered appropriate or safe in the opinion of the Investigator. If the available standard therapy is not considered appropriate or safe in the opinion of the Investigator, the rationale for ineligibility shall be provided and documented in the CRF. Furthermore, in the opinion of the Investigator, if the individual is intolerant to standard therapy, drug information, toxicity, and grade must be documented in the CRF. Progressing on, being intolerant to, or ineligible for immunotherapy is NOT applicable to individuals in Cohort B4.

Additional Cohort B8 Only Specific Criteria:

- Individuals must have at least 1 untreated, active brain metastasis, defined as a new and/or growing lesion at least 5 mm in diameter, without any subsequent local treatment before the start of study treatment, and without associated neurological symptoms requiring urgent intervention with locoregional therapy. Individuals with leptomeningeal disease are not eligible. Prophylactic anticonvulsants are permitted, provided the individual is on a stable dose for ≥ 14 days prior to C1D1, except those taking enzyme-inducing anti-epileptic drugs must have discontinued such therapy with a washout period equivalent to 5 half-lives of the drug.

c) Phase 1b Dose Expansion Part C (CRC):

- Individuals must be appropriate candidates for study treatment and must have received at least one prior oxaliplatin- or irinotecan-containing regimen for advanced or metastatic CRC. Study treatment is considered appropriate where approved measures are no longer effective or are not considered appropriate or safe in the opinion of the Investigator. If the available standard therapy is not considered appropriate or safe in the opinion of the Investigator, the rationale for ineligibility shall be provided and documented in the CRF. Furthermore, in the opinion of the Investigator, if the individual is intolerant to standard therapy, drug information, toxicity, and grade must be documented in the CRF.
- For Cohort C2, individuals should only be enrolled if, in the Investigator's judgment, the individual is appropriate to initiate cetuximab at the standard dose in the Package Insert.

d) Phase 1b Dose Expansion Part D (Solid Tumors Except NSCLC, CRC, and Pancreatic Cancer):

- Individuals must have histological or cytologically proven recurrent/metastatic, unresectable solid tumors except NSCLC, CRC, and pancreatic cancer, and with *KRAS* G12C mutation
- Individuals must be appropriate candidates for study treatment. Study treatment is considered appropriate where approved measures are no longer effective or are not considered appropriate or safe in the opinion of the Investigator. If the available standard therapy is not considered appropriate or safe in the opinion of the Investigator, the rationale for ineligibility shall be provided and documented in the CRF. Furthermore, in the opinion of the Investigator, if the individual is intolerant to standard therapy, drug information, toxicity, and grade must be documented in the CRF.

e) Phase 1b Dose Expansion Part E (NSCLC):

- Individuals must have been previously treated with a KRAS G12C inhibitor.
- Individuals must be appropriate candidates for study treatment. Study treatment is considered appropriate where approved measures are no longer effective or are not considered appropriate or safe in the opinion of the Investigator. If the available standard therapy is not considered appropriate or safe in the opinion of the Investigator, the rationale for ineligibility shall be provided and documented in the CRF. Furthermore, in the opinion of the Investigator, if the individual is intolerant to standard therapy, drug information, toxicity, and grade must be documented in the CRF.

f) Phase 2 (Part F) (Pancreatic Cancer)

- Individuals must have histological or cytologically proven recurrent/metastatic, unresectable pancreatic cancer with *KRAS* G12C mutation.
- Individuals must be appropriate candidates for study treatment. Study treatment is considered appropriate where approved measures are no longer effective or are not considered appropriate or safe in the opinion of the Investigator. If the available standard therapy is not considered appropriate or safe in the opinion of the Investigator, the rationale for ineligibility shall be provided and documented in the CRF. Furthermore, in the opinion of the Investigator, if the individual is intolerant to standard therapy, drug information, toxicity, and grade must be documented in the CRF.

g) Phase 1b Dose Optimization Part G (NSCLC)

- Individuals must not have additional validated oncogenic drivers in NSCLC, if known, e.g., activating alterations in genes such as *EGFR*, *ALK*, *BRAF (V600E)*, *MET* (exon 14), *ROS1*, *RET*, or *NTRK1/2/3*
- Individuals must have histologically or cytologically confirmed Stage IIIB-IIIC or Stage IV NSCLC, previously untreated in the advanced/metastatic setting and not suitable for curative intent radical surgery or radiation therapy. Staging will be according to the AJCC Staging System (8th ed [Amin et al. 2017]).
- Individuals may have received up to one 21-day cycle of pembrolizumab 200 mg Q3W; such therapy must have been initiated within 21 days (+14 days) prior to enrollment.

h) Phase 1b Dose Optimization Part H (CRC):

- Individuals must be appropriate candidates for study treatment and must have received at least 1 prior oxaliplatin- or irinotecan-containing regimen for advanced or metastatic CRC. Study treatment is considered appropriate where approved measures are no longer effective or are not considered appropriate or safe in the opinion of the Investigator. If the available standard therapy is not considered appropriate or safe in the opinion of the Investigator, the rationale for ineligibility shall be provided and documented in the CRF. Furthermore, in the opinion of the Investigator, if the individual is intolerant to standard therapy, drug information, toxicity, and grade must be documented in the CRF.
- For Part H, individuals should only be enrolled if, in the Investigator’s judgment, the individual is appropriate to initiate cetuximab at the standard dose in the Package Insert.

5) Individuals must have an ECOG performance status of 0 or 1 (Oken et al. 1982).

6) Individuals must have adequate organ function, as defined in the table below:

System	Laboratory Value
**Hematologic**	
ANC	≥ 1.5 × 10^9/L
Platelets	≥ 100 × 10^9/L
Hemoglobin[a]	≥ 9 g/dL
**Hepatic**	
TBL	≤ 1.5 × ULN,<br>Individuals with Gilbert's syndrome with a TBL ≤ 3.0 × ULN and direct bilirubin within normal limits are permitted.
ALT and AST	≤ 2.5 × ULN **OR**<br>≤ 5 × ULN if the liver has tumor involvement.
**Renal**	
Serum creatinine **OR**<br>CrCl	< 1.5 × ULN **OR**<br>Calculated creatinine clearance of ≥ 50 mL/min using a validated method such as estimation via Cockcroft/Gault[b], CKD-EPI, or MDRD equations; or direct measurement of creatinine clearance in urine.

a Transfusions to increase an individual's hemoglobin level or initiation of erythropoietin or G-CSF therapy to meet enrollment criteria are not allowed in the 14 days prior to enrollment. If an individual receives transfusions, erythropoietin, or G-CSF therapy ≥ 14 days prior to enrollment, the hematologic criteria listed above must be met following the 14-day window and prior to enrollment. For Cohort B4, Cohort B9, and Part G, individuals can be on a stable dose of erythropoietin (≥ approximately 3 months).

b See Section 9.6.

7) Individuals must have discontinued all previous treatments for cancer with improvement of any prior therapy-related AEs, with the exception of alopecia and Grade 2 neuropathy, and of all clinically significant toxic effects of prior locoregional therapy, surgery, radiotherapy, or systemic anticancer therapy to Grade ≤ 1 or prior baseline. Individuals with ongoing endocrinopathies due to prior treatment that have not resolved but are on appropriate replacement therapy (e.g., thyroid, adrenal or pituitary, or pancreatic) are permitted to enroll.

   - For Cohort B9 only: Individuals with therapy-related toxicities that have developed and are related to any or all components of the KEYNOTE-189 treatment regimen (i.e., pembrolizumab, pemetrexed, carboplatin/cisplatin) (with the exception of the immune-related toxicities outlined in Exclusion Criterion #24) may enroll, if the Investigator determines these toxicities are not required to be resolved at the time of study entry and the individual is still an appropriate candidate for study treatment.

8) Individuals must have discontinued from previous treatments, as shown in the table below:

Previous Treatment	Length of Time Prior to Start of Study Drug(s)
Cytotoxic therapies or targeted agents that are small molecule inhibitors	≥ 14 days or ≥ 5 half-lives, whichever is shorter
Biologic agents that are large molecules including immunotherapy	≥ 21 days<br>For Cohort B4, ≥ 28 days
Radiotherapy<br>Limited-field radiotherapy with palliative intent<br>Other radiotherapy	 ≥ 7 days<br>≥ 21 days
Major surgery	≥ 28 days
**Note:** The systemic therapy washouts above do not apply to those individuals enrolling into Cohort B9 and Part G who have received prior cycle of therapy, as allowed in Inclusion Criterion 4.	

9) Individuals must be able to swallow capsule/tablet.

10) Individuals must agree and adhere to contraceptive use by men or women that is consistent with local regulations regarding the methods of contraception for those participating in clinical studies. See Section 9.3 for guidance on contraceptive use and collection of pregnancy information.

11) Individuals who are WOCBP must have a negative serum or urine pregnancy test documented within 7 days (72 hours for Cohort B4, Cohort B9, and Part G) prior to enrollment (see Section 9.3).

12) Individuals must have an estimated life expectancy of ≥ 12 weeks

13) Individuals must be reliable and willing to make themselves available for the duration of the study and be willing to follow study procedures.

14) Individuals must be capable of giving signed informed consent as described in Section 9.1.2, which includes compliance with the requirements and restrictions listed in the ICF and in this protocol

4.2 Exclusion Criteria

Individuals are excluded from the study if **any** of the following criteria apply:

15) Individual has disease suitable for local therapy administered with curative intent, except as allowed for specific cohorts in Inclusion Criterion 4.
16) Individual has an active fungal, bacterial, and/or active untreated viral infection, including HIV or viral (A, B, or C) hepatitis (screening is not required unless mandated by local health authority).

Note: For Cohort B4, Cohort B9, and Part G only:

- HIV-infected individuals must be on ART and have a well-controlled HIV infection/disease defined as:
    - Individuals on ART must have a CD4+ T-cell count > 350 cells/mm^3 at time of Screening
    - Individuals on ART must have achieved and maintained virologic suppression defined as confirmed HIV RNA level below 50 copies/mL or the lower limit of qualification (below the limit of detection) using the locally available assay at the time of Screening and for at least 12 weeks prior to Screening
    - Individuals on ART must have been on a stable regimen, without changes in drugs or dose modification, for at least 4 weeks prior to study entry (Day 1).
    - HIV-infected individuals with a history of Kaposi sarcoma and/or Multicentric Castleman Disease are excluded.
- Individuals who are HbsAg positive are eligible if they have received HBV antiviral therapy for at least 4 weeks and have undetectable HBV viral load prior to enrollment.

    Note: Individuals should remain on antiviral therapy throughout study intervention and follow local guidelines for HBV antiviral therapy post completion of study intervention.

    Hepatitis B screening tests are not required unless:

    - Known history of HBV infection
    - As mandated by local health authority
- Individuals with history of HCV infection are eligible if HCV viral load is undetectable at Screening.

    Note: Individuals must have completed curative antiviral therapy at least 4 weeks prior to enrollment.

    Hepatitis C screening tests are not required unless:

    - Known history of HCV infection

- As mandated by local health authority

17) Individual has a serious pre-existing medical condition(s) that, in the judgment of the Investigator, would preclude participation in this study, including ILD or severe dyspnea at rest and uncontrolled disease-related pericardial effusion or pleural effusion.

18) Individual has clinically significant, active cardiovascular disease, unstable angina, or history of myocardial infarction within 6 months prior to planned start of LY3537982, or QT interval corrected for heart rate of ≥ 470 msec on screening ECG as calculated using Fridericia's formula (QTcF). If QTcF > 470 msec on more than 1 ECG is obtained during the screening, repeat 2 additional times and use the average to determine eligibility. Note that individuals with implanted pacemakers may enter study without meeting QTc criteria due to nonevaluable measurement. Correction of suspected drug-induced QTcF prolongation or existing bundle branch block prolonged QTcF can be attempted at the Investigator's discretion and in suspected drug-induced prolongation only if clinically safe to do so with either discontinuation of the offending drug or switching to another drug not known to be associated with QTcF prolongation.

19) Individual has a second active primary malignancy or has been diagnosed and/or treated for an additional malignancy within 3 years prior to enrollment with the exception of curatively treated basal cell carcinoma of the skin, nonmetastatic prostate cancer treated with observation only, squamous cell carcinoma of the skin, and/or curatively resected in situ cervical and/or breast cancers. Exceptions may be permitted following discussion with study Investigator and Sponsor.

20) For all individuals except those in Cohort B8: Have untreated active CNS metastases and/or leptomeningeal disease. Individuals with previously treated CNS metastases may participate provided they have:

    - Completed prior CNS-directed therapy (including radiation and/or surgery) ≥ 28 days prior to the first dose of study intervention, and
    - Symptomatically and radiologically stable disease (i.e., without evidence of progression for ≥ 28 days by repeat imaging). For Cohort B4, Cohort B9, and Part G, individuals must be clinically stable without requirement of steroid treatment within 14 days prior to C1D1. Prophylactic anticonvulsants are permitted, provided the individual is on a stable dose for ≥ 14 days prior to C1D1, except individuals taking enzyme-inducing anti-epileptic drugs must have discontinued such therapy with a washout period equivalent to 5 half-lives of the drug.

Prior/Concomitant Therapy

21) Individual has received prior treatment with any KRAS G12C small molecule inhibitor, except in the following scenarios where such prior therapy is allowed:

    a) Phase 1a dose escalation backfill cohort (NSCLC only)

    b) Cohorts E1 [redacted]

    c) Cohort B4

22) Exclusion criterion #22 has been removed.

23) The following individuals will be excluded from Cohort B4, Cohort B9, and Part G:

    a) Individual has experienced a Grade 3 immune-related toxicity or any immune-related toxicity that led to permanent discontinuation of prior anti-PD-1, anti-PD-L1, or other immunotherapy

    b) Individual has experienced a ≤ Grade 3 irAE that has not recovered to Grade 1 after use of corticosteroids that occurred during prior immunotherapy (exceptions: endocrine disorders where individuals with prior endocrine Aes are permitted to enroll if they are considered clinically stable and maintained on appropriate replacement therapy)

    c) Individual has any grade:

        i. Ocular irAE,

        ii. Serious neurologic irAE (e.g., Guillain-Barre syndrome, myasthenia gravis, encephalitis)

        iii. Serious cardiovascular irAE (e.g., myocarditis)

    d) Individual required immunosuppressive agents other than corticosteroids for the management of irAEs or currently requires maintenance doses of ≥ 10 mg prednisone/prednisolone (or equivalent) per day for irAEs

24) The following individuals will be excluded from Cohort B4, Cohort B9, and Part G:

    a) Individual has an active autoimmune disease that has required systemic anti-autoimmune treatment in the past 2 years (i.e., with use of disease modifying agents, corticosteroids, or immunosuppressive drugs). Replacement therapy (e.g., thyroxine, insulin, or physiologic corticosteroid replacement therapy for adrenal or pituitary insufficiency) is not considered a form of systemic treatment and is allowed.

    b) Exclusion Criterion 24(b) has been removed.

c) Individual has received a live vaccine within 30 days prior to the first dose of study drug. Examples of live vaccines include, but are not limited to, the following: measles, mumps, rubella, varicella/zoster (chicken pox), yellow fever, rabies, BCG, and typhoid vaccine. Seasonal influenza vaccines for injection are generally killed virus vaccines and are allowed; however, intranasal influenza vaccines are live attenuated vaccines and therefore are not allowed.
Note: Any licensed COVID-19 vaccine (including for Emergency Use) in a particular country is allowed as long as they are messenger ribonucleic acid (mRNA) vaccines, adenoviral vaccines, or inactivated vaccines.

d) Individual has had an allogeneic tissue/solid organ transplant.

e) Individual has severe hypersensitivity (≥ Grade 3) to pembrolizumab and/or any of its excipients.

f) Individual has received radiation therapy to the lung that is > 30 Gy within 6 months of the first dose of trial treatment.

Note: For Cohort B9 and Part G only:

g) Individual received prior systemic therapy (chemotherapy, immunotherapy, or biological therapy) for advanced or metastatic disease, except as allowed in Inclusion Criterion 4. Individuals who received adjuvant or neoadjuvant therapy are eligible if the last dose of the systemic treatment was completed at least 6 months prior to enrollment.

- For individuals who received immunotherapy in either the neoadjuvant or adjuvant settings, relapse/recurrence of metastatic disease should have occurred at least 6 months after last dose.

Note: For Cohort B9 only:

h) Individual is unable to interrupt NSAIDs 2 days before (5 days for long-acting NSAIDs), the day of, and 2 days following administration of pemetrexed.

i) Individual is unable or unwilling to take folic acid, dexamethasone, or vitamin B12 supplementation.

j) Individual has a known hypersensitivity to any of the excipients of carboplatin, cisplatin, or pemetrexed.

k) Individual has creatinine clearance < 45 mL/min (measured or calculated) at C1D1 or within 48 hours prior to C1D1.

l) Individual is known to be intolerant to any component of their planned treatment regimen at the dose levels specified in each combination agent's Package Insert or Summary of Product Characteristics, as evidenced by Grade 4 hematologic toxicity, any toxicity requiring transfusion support or G-CSF, or other prohibitive toxicity.

Other Exclusions

25) Individual is pregnant, breastfeeding, or expecting to conceive or father children within the projected duration of the trial, starting with the Screening visit through 35 days after the last dose of study medication.
26) Individual has a known allergic reaction against any of the components of the study treatments.
27) Exclusion criterion #27 has been removed.
28) For Cohort B4, Cohort B9, and Part G only, individuals with a history of (noninfectious) pneumonitis/radiation pneumonitis/interstitial lung disease that required steroids or has current pneumonitis/interstitial lung disease.
29) Individual has a prior enrollment in another cohort in this study.

4.3 Lifestyle Considerations

Participants will not be permitted to use herbal supplements in any form while on study due to the unknown risk of potential DDI.

Refer to Section 5.4 for additional guidance on concomitant medications.

4.4 Screen Failures

Screen failures are defined as participants who consent/assent to participate in the clinical study but are not subsequently enrolled. A minimal set of screen failure information is required to ensure transparent reporting of screen failure participants to meet the Consolidated Standards of Reporting Trials publishing requirements and to respond to queries from regulatory authorities. Minimal information includes demography, screen failure details, eligibility criteria, and any SAEs.

Individuals who do not meet the criteria for participation in this study (screen failure) may be rescreened. Individuals may be rescreened up to 2 times if the reason for screen failure is anticipated to have been resolved. The interval between rescreening should be ≥ 2 weeks. Each time rescreening is performed, the participant must sign a new ICF and will be assigned a new identification number. Repeating of laboratory tests during the Screening period or repeating screening tests to comply with the protocol-designated Screening period does not constitute rescreening.

5.0 TREATMENT

Study intervention is defined as any investigational intervention(s) or marketed product(s) intended to be administered to a study participant according to the study protocol.

5.1 Study Intervention(s) Administered

The tables in Section 3.1 show the treatment regimens for Phase 1a dose escalation and Phase 1b dose expansion and dose optimization cohorts. Doses will be administered at approximately the same time on each day. Treatment will be administered in 21-day cycles for all study drugs.

Study interventions are summarized in Table 6.

Table 6: Study Interventions Administered

Intervention	Unit Dose Strength(s)	Route Of Administration
LY3537982[a]	5 mg, 25 mg, and 50 mg capsules	Oral
Cetuximab[b]	2 mg/ml 50 ml vial<br>OR<br>5 mg/ml 20 ml vial	IV infusion
Pembrolizumab[c]	100 mg/4 ml vial	IV infusion
Pemetrexed[c]	Per label dose and schedule	IV infusion
Cisplatin[c]	Per label dose and schedule	IV infusion
Carboplatin[c]	Per label dose and schedule	IV infusion

a Not authorized as defined by the EU Clinical Trial Regulation

b Authorized but not used according to EU authorization

c Authorized as defined by the EU Clinical Trial Regulation and used in accordance with EU authorization

Packaging and Labeling

Study interventions will be supplied by the Sponsor or its designee, or by the site, in accordance with current Good Manufacturing Practice. Study interventions will be labeled as appropriate for country requirements.

For participants enrolled in monotherapy dose escalation, where LY3537982 [redacted] is planned, participants will omit their [redacted] only and should postpone taking [redacted] until site visit. This will allow [redacted] assessment of time-matched ECG and PK to be performed.

The Investigator or his/her designee is responsible for the following:

- Explaining the correct use of the drug(s) and the planned duration of each individual's treatment to the participant and study site personnel
- Verifying that instructions are followed properly

- Maintaining accurate records of study drug dispensing and collection
- Returning all unused medications at the end of the study to the Sponsor or its designee unless Sponsor and sites have agreed all unused medications are to be destroyed by the site, as allowed by local law

Further instructions for each investigational agent are included below.

- **LY3537982** will be administered PO approximately CCI [redacted]
- **Cetuximab** should be administered following institutional-approved standard of care premedication. All concomitant medications, including pre-medication, should be recorded on the CRF. Premedicate with a histamine-1 receptor antagonist intravenously 30 to 60 minutes prior to the first dose or subsequent doses as deemed necessary. Close monitoring is required during the infusion, particularly during the first infusion, and for at least 1 hour after the end of the infusion. Refer to the Package Insert for further details. Participants enrolled into cetuximab combination cohorts will receive an initial loading dose of 400 mg/m^2 cetuximab as an approximately 120-minute infusion on C1D1 followed on C1D8 and C1D15 with a subsequent dose of 250 mg/m^2 as an approximate 60-minute infusion. In Cycle 2 and beyond, participants will receive 250 mg/m^2 cetuximab as an approximately 60-minute infusion on Days 1, 8, and 15 of a 21-day cycle. For participants utilizing a permitted window for a treatment visit, cetuximab must not be administered more frequently than every 5 days or cetuximab should be omitted that visit.
- **Pembrolizumab** will be administered as a dose of 200 mg using a 30-minute IV infusion on Day 1 of each 3-week treatment cycle after all procedures and assessments have been completed. Trial treatment of pembrolizumab may be administered up to 3 days before or after the scheduled Day 1 of each cycle due to administrative reasons. Sites should make every effort to target infusion timing to be as close to 30 minutes as possible. However, given the variability of infusion pumps from site to site, a window between -5 minutes and +10 minutes is permitted (i.e., infusion time is 30 minutes [-5 min/+10 min]). The Pharmacy Manual contains specific instructions for the preparation of the pembrolizumab infusion and administration of infusion solution. Pembrolizumab will be discontinued after approximately 2 years on treatment (35 treatments).

5.1.1 Study Interventions in Cohort B9

The study interventions LY3537982 and pembrolizumab plus pemetrexed with carboplatin or cisplatin for Cohort B9 will be administered as shown in Table 7.

Table 7: Dosing for Cohort B9

Drug	Administration
LY3537982	[redacted]
Pembrolizumab (200 mg Q3W)	• Administered as a dose of 200 mg using a 30-minute IV infusion on Day 1 of each 3-week treatment cycle after all procedures and assessments have been completed and prior to pemetrexed and carboplatin or cisplatin.<br>• It is recommended that any chemotherapy pre-medications not be administered until at least 30 minutes after the completion of the pembrolizumab infusion.
Pemetrexed (500 mg/m^2 Q3W)	• IV infusion over approximately 10 minutes on Day 1. Should occur at a minimum 30 minutes after pembrolizumab.<br>• All participants should receive the appropriate supplementation of vitamin B12, folic acid, and corticosteroid per local practice and/or labels.<br>• The dose will be determined by calculating the participant's BSA at the beginning of each cycle.[a]
Cisplatin (75 mg/m^2 Q3W)	• Administered as an IV infusion approximately 30 minutes after the pemetrexed infusion for the first 4 cycles.<br>• Cisplatin therapy should be immediately preceded and followed by hydration procedures and administered according to local practice and labels. Similarly, antiemetic therapy should follow local guidelines and labels.<br>• The dose will be determined by calculating the participant's BSA at the beginning of each cycle.[a]
Carboplatin (AUC 5 mg/ml/min Q3W)	• Administered as an IV infusion over 15 to 60 minutes (approximately 30 minutes) after pemetrexed for the first 4 cycles as per local practice and labels.<br>• Doses should be calculated using the Calvert formula (Calvert et al. 1989) and should not exceed 750 mg.<br>• Antiemetic therapy should follow local guidelines and labels.

[a] Although it is acceptable to recalculate more frequently, if the participant's weight does not fluctuate by more than ±10% from the weight used to calculate the prior cycle or from baseline, the BSA will not need to be recalculated. A ±10% variance in the calculated total dose will be allowed for ease of dose administration

Pembrolizumab with pemetrexed dosing will begin with Investigator's choice of carboplatin or cisplatin on C1D1. After the completion of 4 cycles of chemotherapy without progressive disease, participants will receive maintenance therapy with pemetrexed and pembrolizumab every 3 weeks. Guidance for administration is provided in Table 7, but it is acceptable to administer pre- or post-medications and study drugs per local practice and/or labels.

During evaluation of emerging safety data from participants enrolled at any time (including during safety lead-in) in Cohort B9, the Sponsor, with agreement from the SRC, may explore the following changes to Cohort B9 dosing administration, if significant toxicity is detected and the risk-benefit profile of the combination has changed:

- On C1D1 only, omit LY3537982 CCI [redacted]
- On the first day of each cycle only (i.e., C1D1, C2D1, etc.), CCI [redacted] while still administering the other combination agents after pembrolizumab in Cohort B9 as specified in Table 7

5.1.2 Study Interventions in Part G

The study interventions LY3537982 and pembrolizumab for Part G will be administered as shown in Table 8.

Table 8: Dosing in Part G

	Arm G1		Arm G2	
**Intervention**[a]	LY3537982	Pembrolizumab	LY3537982	Pembrolizumab
**Starting Dose**	50 mg BID	200 mg	100 mg BID	200 mg
**Schedule**	BID in continuous 21-day cycles	Day 1 of each 21-day cycle	BID in continuous 21-day cycles	Day 1 of each 21-day cycle
**Route**	Oral	30 min IV infusion	Oral	30 min IV infusion
**Authorized as defined by EU Clinical Trial Regulation**	Not authorized	Authorized for participants with PD-L1 TPS ≥50%<br><br>Not used according to authorization for participants with PD-L1 TPS 0% to 49%	Not authorized	Authorized for participants with PD-L1 TPS ≥50%<br><br>Not used according to authorization for participants with PD-L1 TPS 0% to 49%

[a] LY3537982 should be administered at least 30 minutes prior to pembrolizumab.

5.2 Preparation/Handling/Storage/Accountability

1. The Investigator or designee must confirm appropriate temperature conditions have been maintained during transit for all study intervention received and ensure any discrepancies are reported and resolved before use of the study intervention.

2. Only participants enrolled in the study may receive study intervention, and only authorized site staff may supply or administer study intervention. All study intervention must be stored in a secure, environmentally controlled, and monitored (manual or automated) area in accordance with the labeled storage conditions with access limited to the Investigator and authorized site staff.
3. The Investigator, institution, or the head of the medical institution (where applicable) is responsible for study intervention accountability, reconciliation, and record maintenance (i.e., receipt, reconciliation, and final disposition records).
4. Further guidance and information for the final disposition of unused study interventions are provided in the Pharmacy Manual.
5. Investigators should consult the study drug information provided in the Pharmacy Manual or label for the specific administration information (including warnings, precautions, contraindications, adverse reactions, and dose modifications).

5.3 Study Intervention Compliance

Participant compliance with study intervention will be assessed at each visit. A participant diary will be given to the participant in order to help participant compliance with LY3537982, recording food intake time relative to LY3537982 administration, and discussion with Investigators about tolerability. Compliance will be assessed by direct questioning, counting returned capsules/tablets, and reviewing participant diaries. Participants must receive 75% of assigned doses to be considered compliant with study.

Study intervention that is administered by IV will be administered only at the investigational sites by the authorized study site personnel. As a result, treatment compliance is assured. Deviation(s) from the prescribed dosage regimen should be recorded in the eCRF.

5.4 Concomitant Therapy

All treatments that the Investigator considers necessary for a participant’s welfare may be administered at the discretion of the Investigator in keeping with the community standards of medical care.

All concomitant medications received within 28 days prior to the first dose of study treatment and up to 28 days after the last dose of study intervention should be recorded. All concomitant medications administered during SAEs or ECIs are to be recorded. SAEs and ECIs are defined in Section 7.5.

Any medication or vaccine (including over the counter or prescription medicines, vitamins, and/or herbal supplements) must be recorded along with:

- Reason for use

- Dates of administration including start and end dates

Additional guidance on concomitant medications for combination agents is listed below for caution.

[redacted]

CCI [redacted]

Pembrolizumab

Medications or vaccinations specifically prohibited in the exclusion criteria (Section 4.2) are not allowed during the ongoing trial. If there is a clinical indication for any medication or vaccination specifically prohibited during the trial, discontinuation from study intervention or vaccination may be required. The Investigator is to discuss prohibited medication/vaccination with the Sponsor. The final decision on any supportive therapy or vaccination rests with the Investigator and/or the participant's primary physician. However, the decision to continue the participant on study treatment requires the mutual agreement of the Investigator, the Sponsor, and the participant.

The following medications and vaccinations are prohibited during the study for Cohort B4, Cohort B9, and Part G:

- Antineoplastic systemic chemotherapy or biological therapy
- Immunotherapy not specified in this protocol
- Chemotherapy not specified in this protocol
- Investigational agents other than pembrolizumab
- Radiation therapy
  - **Note:** Radiation therapy to a symptomatic solitary lesion or to the brain may be allowed at the Investigator's discretion.
- Live or live attenuated vaccines within 30 days prior to the first dose of study treatment and while participating in the study. **Note:** Killed vaccines are allowed.
  - **Note:** Any licensed COVID-19 vaccine (including for Emergency Use) in a particular country is allowed as long as it is an mRNA vaccine, adenoviral vaccine, or inactivated vaccine. These vaccines will be treated just as any other concomitant therapy.
- Investigational vaccines (i.e., those not licensed or approved for Emergency Use) are not allowed.
- Systemic glucocorticoids except when used for the following purposes:
  - To modulate symptoms of an AE that is suspected to have an immunologic etiology
  - For the prevention of emesis
  - To premedicate for IV contrast allergies
  - To treat chronic obstructive pulmonary disease exacerbations (only short-term oral or IV use in doses > 10mg/day prednisone equivalent)
  - For chronic systemic replacement not to exceed 10 mg/day prednisone equivalent

- Other glucocorticoid use except when used for the following purposes:
  - For topical use or ocular use
  - Intra-articular joint use
  - For inhalation in the management of asthma or chronic obstructive pulmonary disease.
    - **Note:** Inhaled steroids are allowed for management of asthma.

If the Investigator determines that a participant requires any of the aforementioned treatments for any reason, study intervention of pembrolizumab must be discontinued. If an AE is clearly related to the combination agent, dose modification of LY3537982 is not mandated.

Pemetrexed

- Ibuprofen (up to 400 mg daily QD) can be administered with pemetrexed in participants with normal renal function (CRCL > 80 mL/min); caution should be used while administering ibuprofen concurrently with pemetrexed to participants with mild to moderate renal insufficiency (CRCL from 45 to 79 mL/min). Participants with mild to moderate renal insufficiency should avoid taking NSAIDs with short elimination half-lives for a period of 2 days before, the day of, and 2 days following administration of pemetrexed.
- In the absence of data regarding potential interaction between pemetrexed and NSAIDs with longer half-lives, all participants taking these NSAIDs should interrupt dosing for at least 5 days before, the day of, and 2 days following pemetrexed administration. If concomitant administration of an NSAID is necessary, participants should be monitored closely for toxicity, especially myelosuppression, renal, and gastrointestinal toxicity.

5.4.1 Palliative Medicine and Supportive Care

Palliative radiation therapy to small areas of painful metastases, a symptomatic solitary lesion, or to the brain that cannot be managed adequately using systemic or local analgesics is permitted after discussion and agreement with the Sponsor or designee. If the lesion that is to be treated is a target lesion, the lesion will be censored at the time of treatment. However, the participant may be eligible to remain on study drug treatment provided there are other lesions that can be followed up for progression. LY3537982 should not be administered on the day of radiation or surgery and participants should recover from the acute effects of radiation or surgery prior to restarting treatment. Longer delays are acceptable based on Investigator discretion. Appropriate documentation for all forms of premedications, supportive care, and concomitant medications must be captured on the CRF. Replacement hormonal therapy initiated before study entry will be allowed. See Section 6.1 for a discussion of therapies leading to study or study drug discontinuation.

Participants should receive full supportive care. Hematopoietic growth factors (e.g., erythropoietin or G-CSFs) may be administered according to institutional guidelines and the

local Package Insert, except in the DLT observation period. Hematopoietic growth factors will be allowed in the DLT observation period if a participant experiences neutropenia that is declared an AE. Administration of G-CSFs prior to first occurrence of neutropenia is prohibited. Blood product transfusions are permitted throughout the study. If clinically indicated at any time during the study, erythropoietin and packed RBC transfusions may be used according to ASCO guidelines (Rizzo et al. 2008). Prophylactic antibiotic treatment should be consistent with ASCO guidelines (Flowers et al. 2013).

All concomitant medications should be recorded throughout the participant's participation in the study.

5.4.2 Supportive Management for Diarrhea

Participants should receive instructions on the management of diarrhea. In the event of diarrhea, supportive measures should be initiated as early as possible. These include the following:

- At the first sign of loose stools, the participant should initiate antidiarrheal therapy (e.g., loperamide) and notify the Investigator for further instructions and appropriate follow-up.
- Participants should also be encouraged to drink fluids (i.e., 8 to 10 glasses of clear liquids per day).
- Site personnel should assess the response within 24 hours.
- If diarrhea does not resolve with antidiarrheal therapy within 24 hours to at least Grade 1 (per CTCAE Version 5.0), dosing of LY3537982 and, as applicable, combination agents should both be suspended until diarrhea is resolved to at least Grade 1.
- When treatment recommences, dosing should be adjusted as outlined in Section 5.5. For severe cases of diarrhea, the measurement of neutrophil counts and body temperature and proactive management of diarrhea with antidiarrheal agents should be considered. If diarrhea is severe (requiring IV hydration) and/or associated with fever or severe neutropenia, broad-spectrum antibiotics such as fluoroquinolones should be considered, treatment should be suspended, and appropriate dose modification should be instituted per protocol.

Participants with severe diarrhea or any diarrhea associated with severe nausea or vomiting should be carefully monitored and given IV fluids and electrolyte replacement as clinically indicated. Please refer to Section 5.5 for specific dose-related modifications for diarrhea.

5.4.3 Supportive Care with Programmed Death-1 Receptor Therapy (Pembrolizumab Cohort B4, Cohort B9, and Part G)

Participants should receive appropriate supportive care measures as deemed necessary by the treating Investigator. For Cohort B4, Cohort B9, and Part G, participants can be on a stable dose of erythropoietin (≥ approximately 3 months). Suggested supportive care measures for the management of AEs with potential immunologic etiology are outlined along with the dose modification guidelines in Section 9.9.2, Appendix 9.

Note: If after the evaluation of the event, it is determined not to be related to pembrolizumab, the Investigator does not need to follow the treatment guidance. Refer to Table 33 in Section 9.9.2, Appendix 9 for guidelines regarding dose modification and supportive care.

It may be necessary to perform conditional procedures such as bronchoscopy, endoscopy, or skin photography as part of evaluation of the event.

5.4.4 Supportive Care with EGFR Therapy (Cetuximab Cohorts)

Supportive care for cetuximab should follow guidelines provided in the product label or institutional guidelines.

5.4.5 Supportive Care for Pembrolizumab Plus Pemetrexed and Carboplatin or Cisplatin Chemotherapy (Cohort B9)

Standard supportive medications may be used in accordance with institutional guidelines and Investigator discretion. These may include:

- hematopoietic growth factors to treat neutropenia, anemia, or thrombocytopenia in accordance with ASCO or ESMO guidelines, except as noted in Section 5.4.1.
- red blood cells and platelet transfusions
- antiemetic, analgesic, and antidiarrheal medications
- electrolyte repletion (e.g., calcium and magnesium) to correct low electrolyte levels.
- brief, limited use of systemic corticosteroids (≤ 7 days) are permitted where such use is considered SOC (e.g., as premedication for chemotherapeutic agents specified in the protocol, contrast allergy, short courses to treat asthma, chronic obstructive pulmonary disease). Replacement doses of steroids (e.g., prednisone 10 mg daily) are permitted while on study
- thyroid replacement therapy for hypothyroidism
- bisphosphonates, denosumab, and other medications for the treatment of osteoporosis, prevention of skeletal-related events from bone metastases, and/or hypoparathyroidism

Continuation of medications that the participant has been on for the previous 28 days is allowed, provided they are not on the list of prohibited concomitant medications (refer to Exclusion Criteria, Section 4.2). Such therapy may include hormonal therapy for participants with prior breast cancer (e.g., GnRH/LHRH agonists, aromatase inhibitors, selective estrogen receptor modulators, or degraders).

Local treatment while receiving study treatment (e.g., palliative radiation therapy or surgery for bone metastases) is permitted with Sponsor approval as described in Section 5.4.1.

5.5 Dose Modification

The sections below detail dose modification and delays of LY3537982 and combination agents.

If an AE is possibly related to more than 1 study drug, then dose adjustment of LY3537982 should be performed either before or at the same time as dose adjustment of the combination drug(s), as determined appropriate by the Clinical Investigator and in consultation with the Sponsor where required.

If an AE is clearly related to a combination agent, the following guidance for dose modification of LY3537982 is not mandated (see Table 9), except for immune-related AEs in participants receiving pembrolizumab, as detailed in Section 9.9.2, Appendix 9.

If any study drugs are permanently discontinued, dosing could be continued with the remaining compound(s) according to the schedule, if clinically indicated, and per the Investigator's discretion, with the exception of participants enrolled in the cetuximab cohort (C2), where discontinuation will be mandatory since cetuximab is contraindicated in RAS mutant CRC.

Cycles are 21 days in duration regardless of dose interruption unless the dose interruption includes D1 of the next cycle, in which case the next cycle will start with resumption of study drug. In this situation, disease assessments should continue to follow the original schedule.

Treatment cycles may be delayed up to 7 days due to holidays, weekends, bad weather, or other unforeseen circumstances and will not be deemed as protocol violation.

When a study drug is delayed, if possible and appropriate, participants should resume study treatment within 1 treatment cycle and, if not possible, then every effort should be made to start on the originally scheduled first day of the next dosing cycle. In rare circumstances, a delay > 42 days from the previously administered dose may be permitted before permanent treatment discontinuation, as long as the participant has clinical benefit without objective disease progression and is recovering from the toxicity. Such circumstances must be discussed with the Sponsor. All dose modifications should be documented including the approach taken and a clear rationale for the need for modification.

Any participant in the Phase 1a dose escalation who requires a dose reduction will continue to receive the reduced dose for the remainder of the study, unless exceptions are noted.

Participants who require dose modification at the lowest dose level in Phase 1a will be discontinued from study.

For participants in Phase 1b and Phase 2 requiring a dose reduction, any re-escalation of LY3537982 to a prior DL is permitted only after consultation with the Sponsor. After re-escalation, subsequent dose adjustments should be based on the dose that the participant is currently receiving.

5.5.1 Dose Modification of LY3537982 for Treatment-Related Adverse Events

Dose modifications of LY3537982 for TEAEs are shown in Table 9. Participants receiving pembrolizumab (Cohort B4, Cohort B9, and Part G) may also require pembrolizumab dose modifications concurrent with LY3537982 dose modifications; see Section 9.9, Appendix 9.

Table 9: Dose Modifications of LY3537982 for Treatment-Related Adverse Events

Toxicity Type	Toxicity Profile and Severity	LY3537982 Dose Suspension	LY3537982 Dose Reduction
Hematologic toxicity	Grade 3	Dose MUST be suspended until toxicity resolves to at least Grade 2.	Dose MAY be reduced by 1 DL—Investigator's discretion.
	Recurrent Grade 3 or Grade 4	Dose MUST be suspended until toxicity resolves to at least Grade 2.	Dose MUST be reduced by 1 DL.<br>If at lowest DL, then discontinue from study
Hematologic toxicity: Participant requires administration of blood cell growth factors	Regardless of severity<br>(growth factors use according to ASCO guidelines)	Dose MUST be suspended for at least 48 hours after the last dose of blood cell growth factors was administered and until toxicity resolves to at least Grade 2.	Dose MUST be reduced by 1 DL unless already performed for incidence of toxicity that led to the use of growth factor.<br>If at lowest DL, then discontinue from study.
Febrile neutropenia	Any grade	Dose MUST be suspended until toxicity resolves.	Dose MUST be reduced by 1 DL.<br>If already at lowest dose level, then discontinue from study.
Nonhematologic toxicity (except diarrhea, alopecia, and nonhepatic asymptomatic laboratory changes)	Persistent or recurrent Grade 2 that does not resolve with maximal supportive measures within 7 days to baseline or Grade 1	Dose MAY be suspended until toxicity resolves to either baseline or Grade 1.	Dose MAY be reduced by 1 DL—Investigator's discretion.
	Grade 3 or 4 that does not resolve with maximal supportive measures within 72 hours to baseline or Grade 1	Dose MUST be suspended until toxicity resolves to either baseline or Grade 1.	Dose MUST be reduced by 1 DL.<br>If already at lowest dose level, then discontinue from study.

Toxicity Type	Toxicity Profile and Severity	LY3537982 Dose Suspension	LY3537982 Dose Reduction
Hepatotoxicity[a]<br>(see also Section 7.4.2.1)	For Cohort B4, Cohort B9, and Part G only:<br><br>AST or ALT > 3.0 – 5.0 × ULN	Dose MUST be suspended until toxicity resolves to baseline or to < 3.0 × ULN[b]	Dose MAY be reduced by 1 DL—Investigator's discretion.
	Remaining Cohorts:<br><br>AST or ALT > 3.0 – 5.0 × ULN	Dose MAY be suspended until toxicity resolves to baseline or to < 3.0 × ULN	Dose MAY be reduced by 1 DL—Investigator's discretion.
	All cohorts: Persistent or Recurrent:<br><br>AST or ALT > 3.0 – 5.0 × ULN	Dose MUST be suspended until toxicity resolves to baseline or to < 3.0 × ULN	Dose MUST be reduced by 1 DL. If already at lowest DL, then discontinue from study.
	All cohorts:<br><br>AST or ALT > 5.0 – 20 × ULN<br>(*if ALT or AST < 1.5 × ULN at baseline)*<br>**OR**<br>ALT or AST ≥ 3 × baseline<br>(*if ALT or AST ≥ 1.5 × ULN at baseline*)	Dose MUST be suspended until toxicity resolves to baseline or to < 3.0 × ULN	Dose MUST be reduced by 1 DL<br>If already at lowest DL, then discontinue from study.
	All cohorts: AST or ALT > 20.0 × ULN (regardless of baseline)<br>**OR**<br>ALT or AST ≥ 3 × ULN concurrent with TBL ≥ 2 × ULN<br>(*if ALT or AST < 1.5 × ULN at baseline*), in the absence of cholestasis<br>**OR**<br>ALT or AST ≥ 2× baseline concurrent with TBL ≥ 2 × ULN<br>(*if ALT or AST ≥ 1.5 × ULN at baseline*), in the absence of cholestasis	Dose MUST be suspended.	Dose MUST be discontinued.

Toxicity Type	Toxicity Profile and Severity	LY3537982 Dose Suspension	LY3537982 Dose Reduction
Diarrhea	Requires hospitalization or Grade 3 or 4	Dose MUST be suspended until toxicity resolves to at least Grade 1.	Dose MUST be reduced by 1 DL for any Grade 4 and for Grade 3 diarrhea lasting > 3 days despite maximum supportive care<br>If already at lowest DL, then discontinue from study
	Persistent or recurrent Grade 2 that does not resolve with maximal supportive measures within 24 hours to at least Grade 1	Dose MUST be suspended until toxicity resolves to at least Grade 1.	Dose MAY be reduced by 1 DL—Investigator's discretion.
	Persistent or recurrent Grade 1 that does not resolve with maximal supportive measures within 24 hours to baseline	Participant to be followed for 48 hours to ensure grade does not worsen.	Dose MAY be maintained during monitoring—Investigator's discretion.
	Grade ≥ 2 diarrhea recurs despite maximal supportive measures after resuming same DL after initial Grade 2 diarrhea	Dose MUST be suspended until toxicity resolves to at least Grade 1.	Dose MUST be reduced by 1 DL.
Bloody diarrhea	Any grade	Dose MUST be suspended until bloody diarrhea resolves and diarrhea resolves to at least Grade 1.	Dose MUST be reduced by 1 DL.
QTcF Prolongation	Grade ≤ 2 (QTcF average of triplicate readings > 480 ms but ≤ 500 ms)	Continue at current DL.	
	Grade 3 (QTcF average of triplicate readings ≥ 501 ms; > 60 ms change from baseline)	Withhold treatment; if QTcF returns to within 30 ms of baseline or < 450 ms within 14 days, treatment may be resumed at a reduced dose. If the event recurs following dose reduction, treatment will be terminated.	
	Grade 4 (Torsade de pointes; polymorphic ventricular tachycardia; signs/symptoms of serious arrhythmia)	Treatment should be terminated.	

Toxicity Type	Toxicity Profile and Severity	LY3537982 Dose Suspension	LY3537982 Dose Reduction
ILD/Pneumonitis Confirmed	Grade 1	Continue to monitor without dose modification.	
	Grade 2	Hold therapy and treat per institutional guidelines. May resume at a lower DL after pneumonitis is < Grade 2.	
	Grade 3-4	Discontinue and institute appropriate therapy.	

Note: For immune-related AEs, dose modifications for pembrolizumab may also be required, see Section 9.9.

[a] Initiate corticosteroids as per Section 9.9 if immune-mediated hepatitis is suspected.

[b] After a dose suspension for hepatotoxicity, participants in Cohort B4, Cohort B9, and Part G who meet criteria above may resume LY3537982 while still on therapeutic doses of corticosteroids. See Section 9.9.5 to identify laboratory values that must be recorded as ECIs.

5.5.1.1 Further Dose Modification Guidance for LY3537982 in Cohort B4, Cohort B9, and Part G

- LY3537982 should be dose adjusted per the guidelines in Section 5.5.1.
- Adjustment of only 1 study intervention (LY3537982 or pembrolizumab) is acceptable if in the opinion of the Investigator, the toxicity is clearly related to 1 study intervention. For any AEs that develop with potential or known association with pembrolizumab with underlying immune-related etiology as listed in Table 33 (Section 9.9), guidance for pembrolizumab management must be followed and both agents potentially held in accordance with Table 33. If the toxicity is deemed to be possibly related to more than 1 intervention, then each drug should be adjusted.
- Participants who have been dose reduced and who tolerate LY3537982 without toxicity for at least 1 cycle may be re-escalated. Please see Section 5.5.1 and the IB for guidance on specific AEs.
- LY3537982 may be withheld for up to 42 days from the last dose to allow time to recover from drug-related toxicity. Pembrolizumab may be delayed for up to 12 weeks from the last infusion. A longer delay is permitted upon agreement between the Investigator and the Medical Monitor.
- Dose reductions of pembrolizumab are not permitted.

Specific Dose Modification Guidance for LY3537982 in Part G

Participants in Arm G1 and Arm G2 will receive LY3537982 at 50 mg BID and 100 mg BID, respectively. Dose reductions of LY3537982 will be in increments of at least 25 mg and may be greater, based on the nature of the toxicity and the available product formulation. In addition, the dose of LY3537982 can be withheld or adjusted at the Investigator's discretion as clinically indicated (including reducing by more than 1 dose level).

In case a dose adjustment is necessary, the study intervention will be administered as detailed in the following sections. If an AE as outlined in Section 9.9 occurs (suspected immune-mediated adverse events or IRRs), dose adjustments for pembrolizumab and LY357982 should be followed and appropriate intervention initiated.

5.5.2 Dose Modification of Cetuximab

Dose adjustments for cetuximab will be determined by the Investigator in accordance with the approved product label. For participants requiring dose reduction(s), any re-escalation to a prior DL is permitted only after consultation with the Sponsor. After re-escalation, subsequent dose adjustments should be based on the dose that the participant is currently receiving.

5.5.3 Dose Modification for Pembrolizumab

Dose modification guidance for pembrolizumab is detailed in Section 9.9.

5.5.4 Dose Modification for Pemetrexed and Cisplatin or Carboplatin (Cohort B9)

The following rules should guide dosing:

- Toxicity must resolve to a level that, in the opinion of the Investigator (and consistent with applicable clinical guidelines or approved product labels for the respective drugs), allows for retreatment.
- Reduction of only 1 chemotherapy drug is appropriate if in the opinion of the Investigator, the toxicity is clearly related to 1 drug. If the toxicity is deemed to be related to more than 1 drug, then the dose of each drug should be reduced. If a dose reduction of a chemotherapy drug is required, the dose may not be re-escalated. If more than 2 dose reductions are required for any agent, that drug should be discontinued.
- If toxicity is clearly attributed to 1 chemotherapy drug, that drug alone may be discontinued. If participants experience toxicity attributed to cisplatin or carboplatin, the respective agent should be discontinued. Switching between carboplatin or cisplatin is permitted. Participants treated with carboplatin or cisplatin and pemetrexed therapy may have carboplatin/cisplatin, and/or pemetrexed discontinued and continue pembrolizumab alone. Similarly, pembrolizumab may be discontinued and chemotherapy continued, if appropriate.

Dose reductions should be made according to Table 10. In addition, the dose can be delayed or adjusted at the Investigator's discretion as clinically indicated.

Table 10: Dose Levels for Study Interventions in Cohort B9

Intervention	Carboplatin	Cisplatin	Pemetrexed	Pembrolizumab
**Dose Level 0 (Starting Dose)**	AUC 5 mg/ml/min<br>Max dose = 750 mg	75 mg/m^2	500 mg/m^2	200 mg
**Dose Level -1**	AUC 3.75 mg/ml/min<br>Max dose = 562.5 mg	56 mg/m^2	375 mg/m^2	Not permitted
**Dose Level -2**	AUC 2.5 mg/ml/min<br>Max dose = 375 mg	38 mg/m^2	250 mg/m^2	Not permitted
**Dose Level -3**	Discontinue	Discontinue	Discontinue	Not permitted

Please refer to the product label for the current dose modification and toxicity management guidelines for carboplatin, cisplatin, and pemetrexed. If a dose reduction for toxicity occurs with any agent, the dose may not be re-escalated. The guidelines in Table 11 and Table 12 provide

recommendations for dose adjustments. These guidelines do not replace Investigator judgment and applicable local label recommendations if more stringent.

Table 11: Recommended Dose Modifications for Chemotherapy: Hematological Toxicity

Platelets	ANC	Pemetrexed	Cisplatin/Carboplatin
		Dose Level (DL) from Table 10	
≥ 50 × 10^9/L AND	≥ 0.5 × 10^9/L	DL 0	DL 0
≥ 50 × 10^9/L AND	< 0.5 × 10^9/L	DL -1	DL -1
< 50 × 10^9/L without bleeding AND	ANY	DL -1	DL -1
< 50 × 10^9/L with Grade ≥ 2 bleeding AND	ANY	DL -2	DL -2
ANY AND	neutropenic fever	DL -1	DL -1

Table 12: Recommended Dose Modifications for Chemotherapy: Nonhematological Toxicity

Event	CTCAE Grade	Pemetrexed	Cisplatin	Carboplatin
		Dose Level (DL) from Table 10		
Nausea or vomiting[a]	Grade 3 or 4	DL 0	DL 0	DL 0
Diarrhea	Grade 3 or 4	DL -1	DL -1	DL 0
Mucositis	Grade 3 or 4	DL -2	DL 0	DL 0
Neurotoxicity	Grade 2	DL 0	DL -2	DL 0
	Grade 3 or 4	DL -1	Discontinue	DL -1
Transaminase elevation	Grade 3	DL -1	DL -1	DL -1
	Grade 4	Discontinue	Discontinue	Discontinue
Other nonhematological toxicity[b]	Grade 3 or 4	DL -1	DL -1	DL -1

a Despite maximal supportive treatment.

b Of medical consequence that cannot be controlled with treatment.

For participants who develop a clinically significant pleural or peritoneal effusions (on the basis of symptoms or clinical examination) during initiation of pemetrexed therapy, consideration should be given to draining the effusion prior to dosing. However, if, in the Investigator's opinion, the effusion represents progression of disease, the participant should be discontinued from study therapy.

No dosage adjustment is needed in participants with CRCL > 45 mL/min. Insufficient numbers of participants have been studied with CRCL < 45 mL/min to give a dose recommendation. Therefore, pemetrexed/platinum (carboplatin or cisplatin) should not be administered to participants whose CRCL is < 45 mL/min.

5.6 Continued Access to Study Intervention After the End of the Study

End of Study is defined in Section 3.3. Investigators will continue to follow the SoA (Section 7.1) until notified by Sponsor that end of study has occurred.

At the discretion of the Investigator and with prior Sponsor approval, participants who are receiving study intervention at the time of study completion or discontinuation (study termination) may continue to receive study intervention if they are experiencing clinical benefit and continued access would impose no undue risk. The continued access period will apply to this study only if at least 1 participant is still on study intervention when study completion occurs. The Sponsor will notify Investigators when the continued access period begins.

The continued access period will begin after study completion and will conclude at End of Study (Section 3.3). The participant's continued access to trial treatment will end when a criterion for discontinuation is met (Section 6.1) or when LY3537982 is available in the market of the participant's country. Safety follow-up for continued access will begin when the participant and/or the Investigator agree to discontinue study intervention and lasts approximately 28 days. Follow-up procedures will be performed as shown in the Continued Access SOA (Section 7.1.2).

In all cases, no follow-up procedures will be performed for a participant who withdraws informed consent unless he or she has explicitly provided permission and consent.

Participants who are in safety follow-up when the continued access period begins will continue in safety follow-up until the 28-day safety follow-up visit is completed. Long-term follow-up does not apply.

Participants who are in long-term follow-up when the continued access period begins will be discontinued from long-term follow-up.

5.6.1 Treatment After Study Completion

Investigators will continue to follow the SoA (Section 7.1) for all participants until notified by Sponsor that study completion has occurred.

6.0 DISCONTINUATION OF STUDY INTERVENTION AND PARTICIPANT DISCONTINUATION/WITHDRAWAL

The discontinuation of specific sites or of the study as a whole is handled as part of Section 9.1.8.

6.1 Discontinuation of Study Intervention

Possible reasons leading to permanent discontinuation of investigational product:

- Participant decision
- The participant or the participant's designee, e.g., his or her parents or legal guardian, requests to discontinue investigational product.
- If a clinically significant finding is identified (including, but not limited to, changes from baseline in QTcF) after enrollment, the Investigator or qualified designee will determine if the participant can continue in the study, and if any change in participant management is needed. This review of the ECG printed at the time of collection must be documented. Any new clinically relevant finding should be reported as an AE.

In addition, participants will be discontinued from the investigational product in the following circumstances:

- The participant becomes pregnant during the study.
- The participant is significantly noncompliant with study procedures and/or treatment.
- Disease progression. Exceptions for continuing study treatment beyond confirmed radiographic progression may be made on a case-by-case basis for participants who are believed to be clinically benefiting from study treatment, and the Investigator and the Sponsor agree that continuing study treatment is in the participant's best interest. CCI [redacted]
- Unacceptable toxicity
- The participant, for any reason, requires treatment with another therapeutic agent that has been demonstrated to be effective for treatment of the study indication. Discontinuation from study treatment will occur prior to introduction of the new agent.
- The Investigator decides that the participant should be discontinued from study treatment.

- For Cohort B4, Cohort B9, and Part G: Completion of 35 administrations (approximately 2 years) with pembrolizumab.
  Note: The number of administrations is calculated starting with the first dose of pembrolizumab.

Participants discontinuing from the investigational product prematurely for any reason should complete AE and other follow-up procedures per the SoA (Section 7.1), Section 7.4 (Safety Assessments), and Section 7.5 (AEs and SAEs) of the protocol.

6.2 Participant Discontinuation/Withdrawal from the Study

Participants will be discontinued in the following circumstances:

- Enrollment in any other clinical study involving an investigational product or enrollment in any other type of medical research judged not to be scientifically or medically compatible with this study
- Participation in the study needs to be stopped for medical, safety, regulatory, or other reasons consistent with applicable laws, regulations, and GCP.
- The participant or the participant's designee requests to be withdrawn from the study.
- The participant becomes pregnant during the study. See Section 9.3 regarding regulatory reporting requirements on fetal outcome.

Participants discontinuing from the study prematurely for any reason should complete AE and other SFU per the SoA (Section 7.1), Safety Assessments (Section 7.4), and Adverse Events and Serious Adverse Events (Section 7.5) of this protocol.

Participants who withdraw their consent from the study and request to discard their genetic and biomarker samples will have their samples destroyed. Analysis results that are available before the consent withdrawal may be published in articles or other disclosures without identifiable individual participant information.

6.2.1 Discontinuation of Inadvertently Enrolled Participants

If the Sponsor or Investigator identifies a participant who did not meet enrollment criteria and was inadvertently enrolled, then the participant should be discontinued from study treatment, as allowed by local regulations, unless there are extenuating circumstances that make it medically necessary for the participant to continue study treatment. If the Investigator and the Sponsor agree it is medically appropriate to continue, the Investigator must obtain documented approval from the Sponsor to allow the inadvertently enrolled participant to continue in the study, with or without treatment with investigational product. SFU is as outlined in the SoA (Section 7.1), Safety Assessments (Section 7.4), and Adverse Events and Serious Adverse Events (Section 7.5) of the protocol.

6.3 Lost to Follow-Up

A participant will be considered lost to follow-up if he or she repeatedly fails to return for scheduled visits and is unable to be contacted by the study site. Site personnel are expected to make diligent attempts to contact participants who fail to return for a scheduled visit or were otherwise unable to be followed up by the site.

Site personnel, or an independent third party, will attempt to collect the vital status of the participant within legal and ethical boundaries for all participants, including those who did not get investigational product. Public sources may be searched for vital status information. If vital status is determined, this will be documented, and the participant will not be considered lost to follow-up.

Sponsor personnel will not be involved in any attempts to collect vital status information.

7.0 STUDY PROCEDURES AND ASSESSMENTS

Descriptions of assessments are provided in this section. Specific timing requirements for study procedures and assessments by diagnosis at enrollment are provided in SoA Table 13 (Phase 1a), Table 14 (Phase 1a participants who dose escalate), Table 15 (Phase 1b, except Part G, and Phase 2), and Table 20 (Phase 1b, Part G) in Section 7.1.

For PK sampling days and timepoints, see Table 16 (Phase 1a), Table 17 (Phase 1a participants who dose escalate), Table 18 (Phase 1b, except for Part G, and Phase 2), and Table 21 (Phase 1b, Part G).

Note: A time window of ±1 day is permitted for Day 1 visits for Cycles 1 and 2 and ±3 days for Cycle 3 and beyond in all phases.

Additional guidance for study assessments and procedures and collection of samples is provided in Section 9.0 and the Laboratory Manual.

Unless otherwise stated in the subsections below, all samples collected for specified laboratory tests will be destroyed within 60 days of receipt of confirmed test results. Certain samples may be retained for a longer period, if necessary, to comply with applicable laws, regulations, or laboratory certification standards.

Study procedures and their timing are summarized in the SoA (Section 7.1). Protocol waivers or exemptions are not allowed.

Immediate safety concerns should be discussed with the Sponsor immediately upon occurrence or awareness to determine if the participant should continue or discontinue study intervention.

Adherence to the study design requirements, including those specified in the SoA (Section 7.1), is essential and required for study conduct.

All screening evaluations must be completed and reviewed to confirm that potential participants meet all eligibility criteria. The Investigator will maintain a screening log to record details of all participants screened and to confirm eligibility or record reasons for screening failure, as applicable.

Procedures conducted as part of the participant’s routine clinical management (e.g., blood count) and obtained before signing of the ICF may be utilized for screening or baseline purposes, provided the procedures met the protocol-specified criteria and were performed within the time frame defined in the SoA (Section 7.1).

7.1 Schedule of Assessments

7.1.1 Trial Schedules of Assessments

Table 13: Schedule of Assessments for Participants Enrolled in Phase 1a

Phase 1a Screening, On-Study, and Post-Treatment SoA													
	Screening (Day Relative to C1D1)		On Treatment Cycle = 21 days							Post-Treatment			Instructions
			Cycle 1 (±1 days)			Cycle 2 (±1 days)			Cycle 3 and above (±3 days)	EOT[a] (±7 days)	Safety Follow-Up[b] 28 days (±7 days)	Long-Term Follow-Up[c] Every 3 months (±4 weeks)	
**Visit**	≤ 28	≤ 14	D1	D8	D15	D1	D8	D15	D1				
**Procedure**													
Informed consent	X												ICF must be signed before any protocol-specific procedures are performed. The ICF is valid for up to 42 days prior to enrollment.
Medical history	X												Including assessment of pre-existing conditions and historical illnesses, pre-existing toxicities from prior therapies, and habits (such as tobacco and alcohol use).
Cancer treatment history	X												Record prior anticancer therapy.
Concomitant medication	X		See instructions							X	X		At baseline, record prior and concurrent medications. Record all premedication, supportive care, and concomitant medication

Phase 1a Screening, On-Study, and Post-Treatment SoA

	Screening (Day Relative to C1D1)		On Treatment Cycle = 21 days							Post-Treatment			Instructions
			Cycle 1 (±1 days)			Cycle 2 (±1 days)			Cycle 3 and above (±3 days)	EOT[a] (±7 days)	Safety Follow-Up[b] 28 days (±7 days)	Long-Term Follow-Up[c] Every 3 months (±4 weeks)	
**Visit**	**≤ 28**	**≤ 14**	**D1**	**D8**	**D15**	**D1**	**D8**	**D15**	**D1**				
													continuously at every visit and throughout the study.
Physical examination	X		X	X	X	X			X	X	X		
Vital signs	X		X	X	X	X			X	X	X		Measure vital signs (height [at baseline], weight, temperature, blood pressure, pulse rate, and SpO2).
AE collection	X		See instructions.								X		Collect continuously at every visit and throughout the study. CTCAE Version 5.0
ECOG PS	X		X	X	X	X			X	X	X		During study treatment, perform ≤ 3 days prior to treatment.
ECG		X	See Table 16							See Table 16	See Table 16		At baseline (Screening) and SFU: Single ECG<br>On-study: Triplicate ECGs.<br>Participant should be supine for 5 to 10 minutes before collection and remain supine during ECG. Local testing. All digital ECGs will be electronically transmitted to a central ECG laboratory

Phase 1a Screening, On-Study, and Post-Treatment SoA

	Screening (Day Relative to C1D1)		On Treatment Cycle = 21 days							Post-Treatment			Instructions
			Cycle 1			Cycle 2			Cycle 3 and above	EOT[a]	Safety Follow-Up[b]	Long-Term Follow-Up[c]	
			(±1 days)			(±1 days)			(±3 days)	(±7 days)	28 days (±7 days)	Every 3 months (±4 weeks)	
**Visit**	**≤ 28**	**≤ 14**	**D1**	**D8**	**D15**	**D1**	**D8**	**D15**	**D1**				
													designated by the Sponsor for storage.
Hematology		X	X	X	X	X	X	X	X	X	X		May be collected ±1 day from scheduled visit. Unscheduled labs may be included as clinically indicated. See Section 9.2.
Coagulation		X											See Section 9.2. Perform at baseline and as clinically indicated
Clinical chemistry		X	X	X	X	X	X	X	X	X	X		May be collected ±1 day from scheduled visit. See Section 9.2.
Urinalysis		X	X			X			X	X	X		See Section 9.2.
Thyroid function		X											See Section 9.2. Perform at baseline and as clinically indicated.
Pregnancy test		X	X	See instructions		X	See instructions		X	X	X		Applies only to WOCBP. See Section 9.2.<br>Note: During Screening, perform within 7 days.<br>During study treatment, perform as indicated (±3 days) or as required per local regulations and/or institutional guidelines.

Phase 1a Screening, On-Study, and Post-Treatment SoA

	Screening		On Treatment Cycle = 21 days							Post-Treatment			Instructions
	(Day Relative to C1D1)		Cycle 1			Cycle 2			Cycle 3 and above	EOT[a]	Safety Follow-Up[b]	Long-Term Follow-Up[c]	
			(±1 days)			(±1 days)			(±3 days)	(±7 days)	28 days (±7 days)	Every 3 months (±4 weeks)	
**Visit**	**≤ 28**	**≤ 14**	**D1**	**D8**	**D15**	**D1**	**D8**	**D15**	**D1**				
Radiologic imaging and measurement of palpable or visible lesions	X		See instructions.							X			Required for all participants[d]
Brain MRI/CT	X		See instructions.							X			Required at baseline for participants with NSCLC (and as clinically indicated for other tumor types) and serially for participants with treated, stable brain metastases[e]
Survival assessment											X	X	Perform every 3 months (±4 weeks). If an in-person visit is not possible, confirm survival by contacting the participant directly via phone.
Collection of poststudy-treatment anticancer therapy information											X	X	Discontinuation from study must occur prior to introduction of the new agent.
Participant diary			See instructions.										Provide participant diary Day 1. Completed QD by participant. Review at each study visit.

Phase 1a Screening, On-Study, and Post-Treatment SoA

	Screening (Day Relative to C1D1)		On Treatment Cycle = 21 days							Post-Treatment			Instructions
			Cycle 1 (±1 days)			Cycle 2 (±1 days)			Cycle 3 and above (±3 days)	EOT[a] (±7 days)	Safety Follow-Up[b] 28 days (±7 days)	Long-Term Follow-Up[c] Every 3 months (±4 weeks)	
**Visit**	**≤ 28**	**≤ 14**	**D1**	**D8**	**D15**	**D1**	**D8**	**D15**	**D1**				
PK			See Table 16										Refer to Table 16 for PK sampling time points.
Whole blood (PGx)		X											Collect once. Sample can be collected at any time if not collected during Screening.
Biomarker blood		X	X Pre dose			X			See instructions.	X			Collect at Screening, on Day 1 of C1 (predose), C2, C3, C5, C7, and then every third cycle starting with C9D1.<br><br>End of treatment plasma is to be collected when the participant and Investigator agree to discontinue study treatment and prior to the start of new anticancer therapy.
Archival tumor tissue or pretreatment biopsy[f]	X												See Section 7.10 for sample requirements.
Optional tumor biopsy[g]										X			See Section 7.10

[a] An EOT visit within 7 days of the last dose of LY3537982 or the decision to terminate treatment is required, where clinically appropriate.

[b] Safety follow-up begins when the participant and the Investigator agree that the participant will no longer continue study treatment and lasts approximately 28 days (±7 days) from the last dose of study drug.

[c] Long-term follow-up occurs every 3 months (±4 weeks) for the first 2 years after the last dose of study drug and every 6 months thereafter and begins when safety follow-up period is completed and continues until death or study completion. In all cases, no follow-up procedures will be performed for a participant who withdraws informed consent/assent unless he or she has explicitly provided permission and consent/assent.

[d] All participants are required to undergo CT or MRI of chest, abdomen, and pelvis, performed with IV contrast when possible at baseline and then at every 6 weeks (±7 days) for the first 6 months and then every 9 weeks (±7 days) thereafter relative to Cycle 1 Day 1 until radiographic disease progression, death, or study completion, whichever occurs first. All scans at disease assessment timepoints should be performed as scheduled, regardless of any treatment delays or interruptions, and within 14 days of clinical progression according to RECIST v1.1 criteria by the same method used at baseline. A central radiology vendor will be used to collect and store images.

[e] Contrast enhanced brain MRI is preferred; however, if MRI contrast is contraindicated, then MRI without contrast or CT with/without contrast is acceptable. Imaging should be performed at the RECIST response assessment intervals outlined above. A central radiology vendor will be used to collect and store images.

[f] Archival tumor tissue (preferably from the most recent biopsy) should be submitted if available. Formalin-fixed paraffin-embedded tissue blocks or approximately 20 × 5 µm unstained slides, with verification of at least 20% tumor content, should be provided. Fewer than 20 slides will likely be acceptable but should be discussed with the Sponsor. Participants who do not have adequate archival tumor tissue available may undergo an optional fresh tumor biopsy prior to treatment, if it is considered safe to perform. If archival tissue is not available and a fresh tumor biopsy cannot be performed, the participant will likely still be eligible to enroll upon review and approval by the Sponsor.

[g] An optional tissue biopsy may be collected at time of progression if it can be safely performed prior to the start of new anticancer therapy. If disease constituting progression is identified, but the participant is otherwise stable and will continue study treatment beyond progression, the participant may undergo an optional biopsy at the time progression is identified and/or following final discontinuation of study drug. The purpose of these tissue biopsies is to evaluate tumor changes that may have resulted from treatment. For biopsies performed in the setting of disease progression, please contact the Sponsor to inform them of the planned biopsy, and whether therapy will be continued beyond progression or discontinued (EOT).

Table 14: Schedule of Additional Assessments for Participants Enrolled in Phase 1a Who Dose Escalate

	On-Treatment Cycle = 21 days			Instructions for D1 and D8 Additional Assessments
	First Cycle on Escalated Dose		Subsequent Cycles (no additional assessments)	
	(±1 days)			
Visit	D1	D8		
Procedure				
Physical examination	X	X	See Table 13	
Vital signs	X	X	See Table 13	Measure vital signs (height [at baseline], weight, temperature, blood pressure, pulse rate, and SpO2).
ECOG PS	X	X	See Table 13	During study treatment, perform ≤ 3 days prior to treatment.
ECG	See Table 17 for additional ECGs		See Table 16	**At baseline (Screening) and SFU:** Single ECG<br>**On-study:** Triplicate ECGs.<br><br>Participant should be supine for 5 to 10 minutes before collection and remain supine during ECG. Local testing. All digital ECGs will be electronically transmitted to a central ECG laboratory designated by the Sponsor for storage.
Hematology[a]	X	X	See Table 13	May be collected ±1 day from scheduled visit. Unscheduled labs may be included as clinically indicated. See Section 9.2.
Clinical chemistry[a]	X	X	See Table 13	May be collected ±1 day from scheduled visit. See Section 9.2.
PK	See Table 17 for additional PK assessments		See Table 16	

[a] If Day 1 of the new dose falls on the same day (± 3 days) of a previous hematology and blood chemistry assessment, it is not necessary to perform a repeat assessment (physical examination, vital signs, and ECGs must still be performed as indicated).

Table 15: Schedule of Assessments for Participants Enrolled in Phase 1b (Except Part G) and Phase 2

Phase 1b (Except Part G) and Phase 2 Screening, On-Study, and Post-treatment SoA											
	Screening		On-Treatment Cycle = 21 days					Post-treatment			Instructions
	(Day Relative to C1D1)		Cycle 1			Cycle 2	Cycle 3-n	EOT[b]	Safety Follow-Up[c]	Long-Term Follow-Up[d]	
			(±1 days)			(±1 days)	(±3 days)	(±7 days)	28 days (±7 days)	Every 3 months ±4 weeks	
**Visit**	≤ 28	≤ 14	D1	D8[a]	D15[a]	D1	D1				
**Procedure**											
Informed consent	X										ICF must be signed before any protocol-specific procedures are performed. The ICF is valid for up to 42 days prior to enrollment.
Medical history	X										Including assessment of pre-existing conditions and historical illnesses, pre-existing toxicities from prior therapies, and habits (such as tobacco and alcohol use).
Cancer treatment history	X										Record prior anticancer therapy.
Concomitant medication	X		See instructions					X	X		At baseline, record prior and concurrent medications. Record all premedication, supportive care, and concomitant medication continuously at every visit and throughout the study.
Physical examination	X		X	X	X	X	X	X	X		

Phase 1b (Except Part G) and Phase 2 Screening, On-Study, and Post-treatment SoA

	Screening		On-Treatment Cycle = 21 days					Post-treatment			
	(Day Relative to C1D1)		Cycle 1			Cycle 2	Cycle 3-n	EOT[b]	Safety Follow-Up[c]	Long-Term Follow-Up[d]	
			(±1 days)			(±1 days)	(±3 days)	(±7 days)	28 days (±7 days)	Every 3 months ±4 weeks	Instructions
**Visit**	≤ 28	≤ 14	D1	D8[a]	D15[a]	D1	D1				
**Procedure**											
Vital signs	X		X	X	X	X	X	X	X		Measure vital signs (height [at baseline], weight, temperature, blood pressure, pulse rate, and SpO2).
AE collection	X		See instructions						X		Collect continuously at every visit and throughout the study.<br>CTCAE Version 5.0.
ECOG PS	X		X	X	X	X	X	X	X		During study treatment, perform ≤ 3 days prior to treatment.
ECG		X	See Table 18 and Table 19					See Table 18 and Table 19	See Table 18 and Table 19		**At baseline (Screening) and**<br>**On-study:** Single ECG.<br>Participant should be supine for 5 to 10 minutes before collection and remain supine during ECG. Local testing. All digital ECGs will be electronically transmitted to a central ECG laboratory designated by the Sponsor for storage.
Hematology		X	X	X	X	X	X	X	X		May be collected ±1 day from scheduled visit. See Section 9.2, Appendix 2.

Phase 1b (Except Part G) and Phase 2 Screening, On-Study, and Post-treatment SoA

	Screening		On-Treatment Cycle = 21 days					Post-treatment			Instructions
	(Day Relative to C1D1)		Cycle 1			Cycle 2	Cycle 3-n	EOT[b]	Safety Follow-Up[c]	Long-Term Follow-Up[d]	
			(±1 days)			(±1 days)	(±3 days)	(±7 days)	28 days (±7 days)	Every 3 months ±4 weeks	
**Visit**	≤ 28	≤ 14	D1	D8[a]	D15[a]	D1	D1				
**Procedure**											
Coagulation		X									See Section 9.2, Appendix 2. Perform at baseline and as clinically indicated.
Clinical chemistry		X	X	X	X	X	X	X	X		May be collected ±1 day from scheduled visit. See Section 9.2, Appendix 2. For Cohort B9, participants must have CrCl (measured or calculated) of ≥ 45 mL/min at C1D1 (or within 48 hours prior to C1D1).
Urine sample (cohort B1 only)			X								See Table 18
Urinalysis		X	X	See instructions							See Section 9.2, Appendix 2. Perform at baseline and as clinically indicated.

Phase 1b (Except Part G) and Phase 2 Screening, On-Study, and Post-treatment SoA

	Screening		On-Treatment Cycle = 21 days					Post-treatment			Instructions
	(Day Relative to C1D1)		Cycle 1			Cycle 2	Cycle 3-n	EOT[b]	Safety Follow-Up[c]	Long-Term Follow-Up[d]	
			(±1 days)			(±1 days)	(±3 days)	(±7 days)	28 days (±7 days)	Every 3 months ±4 weeks	
**Visit**	≤ 28	≤ 14	D1	D8[a]	D15[a]	D1	D1				
**Procedure**											
Thyroid function		X	X (see instructions)				X (see instructions)		X		May be collected ±1 day from scheduled visit. The screening testing is required for all cohorts. If baseline testing is collected ≤ 3 days prior to C1D1, repeat testing is not required. For participants in Cohort B4 and Cohort B9, additional sampling will be required beginning with C3D1, then D1 every other cycle. For participants who have discontinued pembrolizumab but remain on study, sampling will continue every other cycle for an additional 6 cycles. See Section 9.2, Appendix 2.
Pregnancy test		X	X	See instructions		X	X	X	X		Applies only to WOCBP. See Section 9.2, Appendix 2. **Note:** During Screening, perform within 7 days (72 hours for Cohort B4 and Cohort B9). During study treatment, perform as indicated

Phase 1b (Except Part G) and Phase 2 Screening, On-Study, and Post-treatment SoA

	Screening		On-Treatment Cycle = 21 days					Post-treatment			
	(Day Relative to C1D1)		Cycle 1			Cycle 2	Cycle 3-n	EOT[b]	Safety Follow-Up[c]	Long-Term Follow-Up[d]	
			(±1 days)			(±1 days)	(±3 days)	(±7 days)	28 days (±7 days)	Every 3 months ±4 weeks	Instructions
**Visit**	≤ 28	≤ 14	D1	D8[a]	D15[a]	D1	D1				
**Procedure**											
											(±3 days) or as required per local regulations and/or institutional guidelines.
Radiologic imaging and measure-ment of palpable or visible lesions	X		See instructions[e]					X			Required for all participants[e]
Brain MRI/CT	X		See instructions[f]								Required at baseline for NSCLC participants (and as clinically indicated for other tumor types) and serially for participants with brain metastases[f]
Submit imaging scans to central lab	X		See instructions								For participants in Cohort F1 only. Submit scans in a timely manner; refer to the imaging manual for details.<br>This submission is for central review.

**Phase 1b (Except Part G) and Phase 2 Screening, On-Study, and Post-treatment SoA**											
	Screening		On-Treatment Cycle = 21 days					Post-treatment			Instructions
	(Day Relative to C1D1)		Cycle 1			Cycle 2	Cycle 3-n	EOT[b]	Safety Follow-Up[c]	Long-Term Follow-Up[d]	
			(±1 days)			(±1 days)	(±3 days)	(±7 days)	28 days (±7 days)	Every 3 months ±4 weeks	
**Visit**	≤ 28	≤ 14	D1	D8[a]	D15[a]	D1	D1				
**Procedure**											
Survival assessment									X	X	Perform every 3 months (±4 weeks). If an in-person visit is not possible, confirm survival by contacting the participant directly via phone.
Collection of poststudy-treatment anticancer therapy information									X	X	Discontinuation from study must occur prior to introduction of the new agent.
Participant diary			See instructions								Provide participant diary D1. Completed QD by participant. Review at each study visit for first 2 cycles.
PK			See Table 18 and Table 19								Refer to Table 18 and Table 19 for PK sampling time points.
Whole blood (PGx)		X									Collect once. Sample can be collected at any time if not collected during Screening.

Phase 1b (Except Part G) and Phase 2 Screening, On-Study, and Post-treatment SoA												
	Screening		On-Treatment Cycle = 21 days					Post-treatment			Instructions
	(Day Relative to C1D1)		Cycle 1			Cycle 2	Cycle 3-n	EOT[b]	Safety Follow-Up[c]	Long-Term Follow-Up[d]	
			(±1 days)			(±1 days)	(±3 days)	(±7 days)	28 days (±7 days)	Every 3 months ±4 weeks	
**Visit**	≤ 28	≤ 14	D1	D8[a]	D15[a]	D1	D1				
**Procedure**											
Biomarker blood	X		X (Pre dose)			X	See instructions	X			Collect at Screening, on Day 1 of C1 (predose), C2, C3, C5, C7, and then every third cycle starting with C9D1. End of treatment plasma is to be collected when the participant and Investigator agree to discontinue study treatment prior to the start of new anticancer therapy.
Archival tumor tissue or pretreatment biopsy[g]	X										See Section 7.10 for sample requirement
Optional tumor biopsy[h]								X			See Section 7.10

[a] Cycle 1 Day 8 and Day 15 visits apply only to the safety lead-in participants. For Cohort B9, the C1D15 visit (inclusive of labs) is only necessary as clinically indicated.

[b] An EOT visit within 7 days of the last dose of LY3537982 or the decision to terminate treatment is required, where clinically appropriate.

[c] Safety follow-up begins when the participant and the Investigator agree that the participant will no longer continue study treatment and lasts approximately 28 days ±7 days.

[d] Long-term follow-up occurs every 3 months ±4 weeks for the first 2 years after the last dose of study drug and every 6 months thereafter and begins when safety follow-up period is completed and continues until death or study completion. In all cases, no follow-up procedures will be performed for a participant who withdraws informed consent/assent unless he or she has explicitly provided permission and consent/assent.

[e] All participants are required to undergo CT or MRI of chest, abdomen, and pelvis, performed with IV contrast when possible at baseline and then at every 6 weeks (±7 days) for the first 6 months and then every 9 weeks (±7 days) thereafter relative to C1D1 until radiographic disease progression, death, or study completion, whichever occurs first. All scans at disease assessment timepoints should be performed as scheduled, regardless of any treatment delays or interruptions and within 14 days of clinical progression according to RECIST v1.1 criteria by the same method used at baseline. A central radiology vendor will be used to collect and store images.

[f] Contrast enhanced brain MRI is preferred; however, if MRI contrast is contraindicated, then MRI without contrast or CT with/without contrast is acceptable. Imaging should be performed at the RECIST response assessment intervals outlined in footnote e above). In the absence of known or suspected disease involvement, head and neck CT/MRI scans are not required for malignancies other than those originating in the head and neck region. Other areas of scanning may also differ depending on disease type. A central radiology vendor will be used to collect and store images.

[g] Archival tumor tissue (preferably from the most recent biopsy) should be submitted if available. Formalin-fixed paraffin-embedded tissue blocks or approximately 20 × 5 µm unstained slides, with verification of at least 20% tumor content, should be provided. Fewer than 20 slides will likely be acceptable but should be discussed with the Sponsor. Participants who do not have adequate archival tumor tissue available may undergo an optional fresh tumor biopsy prior to treatment, if it is considered safe to perform. If archival tissue is not available and a fresh tumor biopsy cannot be performed, the participant will likely still be eligible to enroll upon review and approval by the Sponsor.

[h] An optional tissue biopsy may be collected at time of progression if it can be safely performed prior to the start of new anticancer therapy. If disease constituting progression is identified, but the participant is otherwise stable and will continue study treatment beyond progression, the participant may undergo an optional biopsy at the time progression is identified and/or following final discontinuation of study drug. The purpose of these tissue biopsies is to evaluate tumor changes that may have resulted from treatment. For biopsies performed in the setting of disease progression, please contact the Sponsor to inform them of the planned biopsy, and whether therapy will be continued beyond progression or discontinued (EOT).

Table 16: Sampling Schedule for PK and ECGs for Participants in Phase 1a

Phase 1a PK sampling schedule is subject to change based on emerging PK.

Cycle	Day	Time after Dose (hours)[a]	LY3537982	Collect ECG[b]
1	1	Predose[c]	X	X[c]
		[redacted]	X	X
			X	X
			X	
			X	X
			X	
			X	X
			X	X
	2		X	X
	8 (±1 day, to align with LY3537982 dosing day)	Predose[c]	X	X[c]
		[redacted]	X	X
			X	X
			X	
			X	X
			X	
			X	X
			X	X
C2D1, C4D1	1	Predose[c]	X	X[c]
		[redacted]	X	X
EOT				X[f]
Safety Follow-Up				X[g]

In cases where doses are being held, participants do not need to have PK draws completed. If/when a participant resumes treatment, the PK sampling should be completed on the appropriate day as per protocol.

a [redacted]

b All ECGs in Phase 1a (excluding Screening and follow-up ECGs) are to be triplicate ECGs; the 3 replicates of each should be completed within 5 minutes and preferably 1 minute apart.

c Predose ECGs should be taken at either 90 (-90) minutes, 60 (-60) minutes, or 30 (-30) minutes. Predose timepoints are flexible for site convenience. Predose ECG should be taken prior to time match PK sampling.

d This timepoint is flexible based on the emerging PK data.

e [redacted]

f ECG may be collected at any time.

g ECG may be collected at any time for participants who develop on-study clinically significant ECG changes/changes in ECG parameters compared to baseline, to ensure resolution following EOT.

Table 17: Sampling Schedule for Additional PK and ECGs for Participants in Phase 1a who Dose Escalate

Phase 1a PK sampling schedule is subject to change based on emerging PK.

First Cycle on Escalated Dose	Time after Dose (hours)[a, b]	LY3537982	Collect ECG[c]
D1	Predose[d]	X	X[c]
	[redacted]	X	X
		X	X
		X	X
D8 [redacted]	Predose[d]	X	X[c]
	[redacted]	X	X
		X	X
		X	X
Subsequent Cycles	See Table 16		

[a] Time after the participant has ingested the full amount of the [redacted]

[b] Please note windows for the timepoints as follows for D1 and D8: [redacted]

[c] All ECGs in Phase 1a (excluding Screening and follow-up ECGs) are to be triplicate ECGs; the 3 replicates of each should be completed within 5 minutes and preferably 1 minute apart.

[d] Predose timepoints are flexible for site convenience either [redacted]

Table 18: Sampling Schedule for PK and ECGs for Participants in Phase 1b (Except Part G and Cohort B9) and Phase 2

Phase 1b PK sampling schedule is subject to change based on PK data and analysis results from the Phase 1a portion of the study.

Cycle	Day	Time After Dose (hours)[a]	LY3537982 (All Participants)	Combination Agent Additional Sample, per Cohort (B4 and C2 Only)[b]	Collect ECG (All Participants)[c]	Collect urine sample (Cohort B1 Only)[d]
1	1	Predose	X		X[e]	X
		[redacted]	X		X	
			X		X	
			X		X	
			X		X	
2	1	Predose	X	Cetuximab (C2)<br>Pembrolizumab (B4)	X[e]	
		[redacted]	X			
			X		X	
			X			
3 and 5	1	Predose	X	Cetuximab (C2)<br>Pembrolizumab (B4)	X[e]	
EOT					X[g]	
Safety Follow-Up					X[h]	

a Time after dose starts after the participant has ingested the full amount of the LY3537982 capsules. [redacted]

b Combination agent samples are to be collected only if the participant is being treated by that agent. See Table 19 for PK and ECGs for Cohort B9.

c All ECGs in Phase 1b dose expansion cohorts should be single ECGs. In Cohort B4, ECG collections should continue during LY3537982 dose holds or discontinuations.

d Collect urine sample from participants in Cohort B1 only, starting at the time of the morning dose and completed collection at approximately 8 hours postdose. Total urine output will be collected and pooled.

e Predose ECGs should be taken at either 90 (-90) minutes, 60 (-60) minutes, or 30 (-30) minutes. Predose timepoints are flexible for site convenience.

f [redacted]

g ECG may be collected at any time.

[h] ECG may be collected at any time for participants who develop on-study clinically significant ECG changes/changes in ECG parameters compared to baseline, to ensure resolution following EOT.

Table 19: Sampling Schedule for PK and ECGs for Participants in Cohort B9

Cycle	Day	Time (Window) Relative to LY3537982 Dosing	LY3537982[a]	Combination Agent	Collect ECG[b]
1	1	Predose	CCI [redacted]		X[c]
		Pre-pembrolizumab infusion	[redacted]		
		Post-pemetrexed infusion	[redacted]	Pemetrexed	
		Post-cisplatin/carboplatin infusion	[redacted]	Pemetrexed and cisplatin or carboplatin[d]	X[d]
		8 h post dose (PK and ECGs at this timepoint are optional)	[redacted]		X
2	1	Predose	[redacted]		X[c]
		Pre-pembrolizumab infusion	[redacted]		
		Post-pemetrexed infusion	[redacted]	Pemetrexed	
		Post-cisplatin/carboplatin infusion	[redacted]	Pemetrexed and cisplatin or carboplatin[d]	X[d]
		8 h post dose (PK and ECGs at this timepoint are optional)	[redacted]		X
3 and 5	1	Predose	[redacted]		X[c]
SFU					X[e]
EOT					X[f]

[a] CCI [redacted]

[b] All ECGs in this and other Phase 1b dose expansion cohorts should be single ECGs. Collect ECGs 5 minutes before the PK blood draw. ECG collections should proceed as noted in this table during LY3537982 dose holds or discontinuations.

[c] Predose ECGs should be taken at either 90 (-90) minutes, 60 (-60) minutes, or 30 (-30) minutes. Predose timepoints are flexible for site convenience.

[d] **Collections at this timepoint are not required for participants who have completed or discontinued treatment with a platinum agent.**

[e] ECG may be collected at any time.

[f] ECG may be collected at any time for participants who develop on-study clinically significant ECG changes/changes in ECG parameters compared to baseline, to ensure resolution following EOT.

Table 20: Schedule of Assessments for Participants in Part G – Dose Optimization

**Part G**	**Prescreening**	**Screening**		**Treatment Period Cycle = 21 days**		**Posttreatment**			**Comments**
				Cycle 1	Cycle 2-n	EOT[a]	Safety Follow-Up[b]	Long-Term Follow-Up[c]	
Relative Day within Dosing Cycle		≤ 28	≤ 14	1	1	(± 7 days)	28 |(±7 days)	Every 3 months (±4 weeks)	Screening: Procedures may be conducted over more than 1 day as long as all activities are completed within the allowable visit tolerance.
Visit Window (Days)		≤ 28		±1	±1 Cycle 2, ±3 Cycle 3+				
Visit Detail								T	T: Telephone Visit, if no study procedures are scheduled.
Informed consent for prescreening	X								OPTIONAL:<br>For participants without a local result for *KRAS* G12C and/or PD-L1 status a prescreening consent may be used to provide information to the participant regarding testing for determination of *KRAS* and/or PD-L1 status at a Sponsor-designated laboratory.
Informed consent		See comments							The informed consent form must be signed before any protocol-specific tests or procedures are performed. ICF is valid for up to 42 days prior to randomization.
Inclusion and exclusion criteria		X							See Section 4.0.
Pathology and molecular reports describing PD-L1 status and *KRAS* G12C mutation		X							De-identified molecular report(s) and pathology report(s) describing PD-L1 status and *KRAS* G12C mutation, as well as other alterations if known, must be submitted. Sponsor review is required prior to starting study intervention, unless discussed and agreed with the Sponsor.
Demographics		X							Includes year of birth, sex, and race.

**Part G**	**Prescreening**	**Screening**		**Treatment Period Cycle = 21 days**		**Posttreatment**			**Comments**
				Cycle 1	Cycle 2-n	EOT[a]	Safety Follow-Up[b]	Long-Term Follow-Up[c]	
Relative Day within Dosing Cycle		≤ 28	≤ 14	1	1	(± 7 days)	28 |(±7 days)	Every 3 months (±4 weeks)	Screening: Procedures may be conducted over more than 1 day as long as all activities are completed within the allowable visit tolerance.
Visit Window (Days)		≤ 28		±1	±1 Cycle 2, ±3 Cycle 3+				
Visit Detail								T	T: Telephone Visit, if no study procedures are scheduled.
Preexisting conditions and medical history, including relevant surgical history		X							All ongoing conditions and relevant past surgical and medical history should be collected.
Prior treatments for NSCLC		X							Record prior adjuvant or neoadjuvant systemic anticancer therapy, surgeries, and/or radiotherapy.
Substance use (tobacco and nicotine replacement use)		X							
Concomitant medications		X	X	X	X	X	X		Record all medication(s) that are ongoing at time of consent and are administered through 28 days posttreatment follow-up.
Adverse events (AEs)	X	X	X	X	X		X	X	See Section 7.5.4 for timing of AE/SAE collection. During all periods including prescreening, AEs to be recorded per Section 7.5 and SAEs to be reported per Section 7.5.3.

Part G	Prescreening	Screening		Treatment Period Cycle = 21 days		Posttreatment			Comments
				Cycle 1	Cycle 2-n	EOT[a]	Safety Follow-Up[b]	Long-Term Follow-Up[c]	
Relative Day within Dosing Cycle		≤ 28	≤ 14	1	1	(± 7 days)	28 |(±7 days)	Every 3 months (±4 weeks)	Screening: Procedures may be conducted over more than 1 day as long as all activities are completed within the allowable visit tolerance.
Visit Window (Days)		≤ 28		±1	±1 Cycle 2, ±3 Cycle 3+				
Visit Detail								T	T: Telephone Visit, if no study procedures are scheduled.
**Physical Evaluation**									
Height		X							
Weight			X	X	X		X		
Vital Signs			X	X	X	X	X		Include pulse rate, blood pressure, respiratory rate, oxygen saturation, and temperature. Measured after participant has been sitting at least 5 min.
Physical examination			X			X			Excludes pelvic, rectal, and breast exams unless clinically indicated.
Physical assessment				X	X		X		As indicated based on participant status and standard of care.

Part G	Prescreening	Screening		Treatment Period Cycle = 21 days		Posttreatment			Comments
				Cycle 1	Cycle 2-n	EOT[a]	Safety Follow-Up[b]	Long-Term Follow-Up[c]	
Relative Day within Dosing Cycle		≤ 28	≤ 14	1	1	(± 7 days)	28 |(±7 days)	Every 3 months (±4 weeks)	Screening: Procedures may be conducted over more than 1 day as long as all activities are completed within the allowable visit tolerance.
Visit Window (Days)		≤ 28		±1	±1 Cycle 2, ±3 Cycle 3+				
Visit Detail								T	T: Telephone Visit, if no study procedures are scheduled.
12-lead ECG			X	X	X	X	X		Obtain triplicate central ECGs at<br>• Screening<br>• C1D1 approximately 2.5 hr after the LY3537982 dose*<br>• C2-C8D1 within 3 hr after LY3537982 dose*, and<br>• Starting on C10D1, collect on D1 of every other cycle (C10D1, C12D1, etc.) within 3 hr after LY3537982 dose.*<br><br>Obtain additional ECGs as clinically indicated.<br>* ECG For collections at these timepoints should continue during LY3537982 dose holds or discontinuations
ECOG PS Evaluation			X	X	X	X	X		

Part G	Prescreening	Screening		Treatment Period Cycle = 21 days		Posttreatment			Comments
				Cycle 1	Cycle 2-n	EOT[a]	Safety Follow-Up[b]	Long-Term Follow-Up[c]	
Relative Day within Dosing Cycle		≤ 28	≤ 14	1	1	(± 7 days)	28 |(±7 days)	Every 3 months (±4 weeks)	Screening: Procedures may be conducted over more than 1 day as long as all activities are completed within the allowable visit tolerance.
Visit Window (Days)		≤ 28		±1	±1 Cycle 2, ±3 Cycle 3+				
Visit Detail								T	T: Telephone Visit, if no study procedures are scheduled.
Tumor Assessment									
Radiologic imaging and measurement of palpable or visible lesions		X		See comments		X			Timing is based on start of treatment. All participants are required to undergo CT or MRI of chest, abdomen, and pelvis, performed with IV contrast when possible. Perform assessments every 6 wk (±7 days) until Week 24, every 9 wk (±7 days) thereafter, beginning from C1D1 of study therapy, e.g., at Weeks 6, 12, 18, 24, 33, 42, 49, etc., until radiographic disease progression, the start of a new anticancer therapy, death, or study completion. The scanning interval should be maintained even if cycles are delayed and within 14 days of clinical progression according to RECIST v1.1 criteria by the same method used at baseline. As a result, the scans may not always occur at the end of a cycle. Scans may also be obtained as clinically indicated. See Section 7.3. A central radiology vendor will be used to collect and store images.

**Part G**	**Prescreening**	**Screening**		**Treatment Period Cycle = 21 days**		**Posttreatment**			**Comments**
				Cycle 1	Cycle 2-n	EOT[a]	Safety Follow-Up[b]	Long-Term Follow-Up[c]	
Relative Day within Dosing Cycle		≤ 28	≤ 14	1	1	(± 7 days)	28 (±7 days)	Every 3 months (±4 weeks)	Screening: Procedures may be conducted over more than 1 day as long as all activities are completed within the allowable visit tolerance.
Visit Window (Days)		≤ 28		±1	±1 Cycle 2, ±3 Cycle 3+				
Visit Detail								T	T: Telephone Visit, if no study procedures are scheduled.
Nuclear Medicine bone scintigraphy, PET scan, or PET component of PET/CT scan		X		See comments			X	X	For participants with nonmeasurable bone disease, bone scintigraphy (preferred) or PET scan or PET component of PET/CT scan may be obtained every 24 wk (±7 days) or more often if clinically indicated. The scanning interval should be maintained even if cycles are delayed. As a result, the scans may not always occur at the end of a cycle. Scans may also be obtained as clinically indicated. See Section 7.3.

**Part G**	**Prescreening**	**Screening**		**Treatment Period Cycle = 21 days**		**Posttreatment**			**Comments**
				Cycle 1	Cycle 2-n	EOT[a]	Safety Follow-Up[b]	Long-Term Follow-Up[c]	
Relative Day within Dosing Cycle		≤ 28	≤ 14	1	1	(± 7 days)	28 |(±7 days)	Every 3 months (±4 weeks)	Screening: Procedures may be conducted over more than 1 day as long as all activities are completed within the allowable visit tolerance.
Visit Window (Days)		≤ 28		±1	±1 Cycle 2, ±3 Cycle 3+				
Visit Detail								T	T: Telephone Visit, if no study procedures are scheduled.
CT/MRI (Brain)		X		See comments				See comments	Required at baseline for participants with NSCLC (and as clinically indicated for other tumor types) and serially for participants with treated, stable brain metastases. Contrast enhanced brain MRI is preferred; however, if MRI contrast is contraindicated, then MRI without contrast or CT with/without contrast is acceptable. Imaging should be performed at the RECIST response assessment intervals outlined in radiologic imaging comments above. In the absence of known or suspected disease involvement, head and neck CT/MRI scans are not required for malignancies other than those originating in the head and neck region. Other areas of scanning may also differ depending on disease type. A central radiology vendor will be used to collect and store images.
Submit imaging scans to central vendor		X				See comments			Scans to be submitted within timely manner. Refer to the imaging manual for details.

Part G	Prescreening	Screening		Treatment Period Cycle = 21 days		Posttreatment			Comments
				Cycle 1	Cycle 2-n	EOT[a]	Safety Follow-Up[b]	Long-Term Follow-Up[c]	
Relative Day within Dosing Cycle		≤ 28	≤ 14	1	1	(± 7 days)	28 |(±7 days)	Every 3 months (±4 weeks)	Screening: Procedures may be conducted over more than 1 day as long as all activities are completed within the allowable visit tolerance.
Visit Window (Days)		≤ 28		±1	±1 Cycle 2, ±3 Cycle 3+				
Visit Detail								T	T: Telephone Visit, if no study procedures are scheduled.
**Participant Dosing Diary (Paper)**									
Study Administration Log dispensed				X	X				
Study Administration Log review					X				
Study Administration Log return					X				

Part G	Prescreening	Screening		Treatment Period Cycle = 21 days		Posttreatment			Comments
				Cycle 1	Cycle 2-n	EOT[a]	Safety Follow-Up[b]	Long-Term Follow-Up[c]	
Relative Day within Dosing Cycle		≤ 28	≤ 14	1	1	(± 7 days)	28 (±7 days)	Every 3 months (±4 weeks)	Screening: Procedures may be conducted over more than 1 day as long as all activities are completed within the allowable visit tolerance.
Visit Window (Days)		≤ 28		±1	±1 Cycle 2, ±3 Cycle 3+				
Visit Detail								T	T: Telephone Visit, if no study procedures are scheduled.
**Laboratory Tests and Sample Collections**									
Hematology			X	X	X	X	X		If screening testing is performed ≤ 7 days prior to C1D1, repeat testing does not need to occur on C1D1. Should be performed on D1 of every subsequent cycle (e.g., C2D1, C3D1). Additional assessments may be obtained at the discretion of the Investigator. Beginning at Cycle 8 Day 1: Collections should occur every other cycle (C8, C10, C12, etc.)
Clinical Chemistry			X	X	X	X	X		If baseline testing is performed ≤ 7 days prior to C1D1, repeat testing does not need to occur on C1D1. Should be performed on D1 of every subsequent cycle (e.g., C2D1, C3D1). Additional assessments may be obtained at the discretion of the Investigator. Beginning at Cycle 8 Day 1: Collections should occur every other cycle (C8, C10, C12, etc.)

Part G	Prescreening	Screening		Treatment Period Cycle = 21 days		Posttreatment			Comments
				Cycle 1	Cycle 2-n	EOT[a]	Safety Follow-Up[b]	Long-Term Follow-Up[c]	
Relative Day within Dosing Cycle		≤ 28	≤ 14	1	1	(± 7 days)	28 |(±7 days)	Every 3 months (±4 weeks)	Screening: Procedures may be conducted over more than 1 day as long as all activities are completed within the allowable visit tolerance.
Visit Window (Days)		≤ 28		±1	±1 Cycle 2, ±3 Cycle 3+				
Visit Detail								T	T: Telephone Visit, if no study procedures are scheduled.
Thyroid			X	X	See comments		X		Beginning at Cycle 3 Day 1: collect on D1 of every other cycle (C3D1, C5D1, C7D1, etc.). For participants who have discontinued pembrolizumab but remain on study, sampling will continue every other cycle for an additional 6 cycles. See Section 9.2, Appendix 2.
Urinalysis			X		See comments				For Cycles 2-n, collect as clinically indicated.
Serum pregnancy		X							Only for WOCBP and females with a history of tubal ligation. At least 1 pregnancy test before first dose must be serum. See Section 9.3, Appendix 3.
Urine pregnancy				X	See comments	X	X	See comments	Collect for WOCBP only. Done locally and prior to administering study intervention. Additional pregnancy tests should be performed at any time during the trial as required by local regulation, or at the Investigator's discretion. See Section 7.5.9.
Tumor tissue for determination of *KRAS* and/or PD-L1 status	X								Testing at a Sponsor-designated laboratory may be used for determination of *KRAS* and/or PD-L1 status only if qualifying local results are unavailable. See Section 7.10 .

Part G	Prescreening	Screening		Treatment Period Cycle = 21 days		Posttreatment			Comments
				Cycle 1	Cycle 2-n	EOT[a]	Safety Follow-Up[b]	Long-Term Follow-Up[c]	
Relative Day within Dosing Cycle		≤ 28	≤ 14	1	1	(± 7 days)	28 (±7 days)	Every 3 months (±4 weeks)	Screening: Procedures may be conducted over more than 1 day as long as all activities are completed within the allowable visit tolerance.
Visit Window (Days)		≤ 28		±1	±1 Cycle 2, ±3 Cycle 3+				
Visit Detail								T	T: Telephone Visit, if no study procedures are scheduled.
Pharmaco-kinetic (PK) samples				See Table 21					See Table 21
**Stored Samples**									
Whole blood (PGx)				X					Blood sample can be obtained at or after the specified visit. See Section 7.9.
Biomarker blood				X	See comments	X			Blood samples to be collected at C1D1 (predose), C2D1, C3D1, and at progression or EOT. See Section 7.10.
Tumor tissue		X							Submit tissue, if available. If a tissue block with sufficient sample was submitted during prescreening for testing of *KRAS* and/or PD-L1 at a Sponsor-designated laboratory, additional tissue is not required. See Section 7.10.
Tumor biopsy (optional)[d]	X	X		See comments		X			Optional tumor biopsy may be performed prior to treatment for participants who do not have sufficient tumor tissue sample available for testing at a Sponsor-designated laboratory and/or biomarker assessments and upon disease progression or EOT if medically feasible. See Section 7.10.

**Part G**	**Prescreening**	**Screening**		**Treatment Period Cycle = 21 days**		**Posttreatment**			**Comments**
				Cycle 1	Cycle 2-n	EOT[a]	Safety Follow-Up[b]	Long-Term Follow-Up[c]	
Relative Day within Dosing Cycle		≤ 28	≤ 14	1	1	(± 7 days)	28 (±7 day s)	Every 3 months (±4 weeks)	Screening: Procedures may be conducted over more than 1 day as long as all activities are completed within the allowable visit tolerance.
Visit Window (Days)		≤ 28		±1	±1 Cycle 2, ±3 Cycle 3+				
Visit Detail								T	T: Telephone Visit, if no study procedures are scheduled.
**Randomization and Dosing**									
Register visit with IWRS	X	X	X	X	X	X			
Randomization via IWRS				See comments					Upon confirmation of eligibility, site to randomize via IWRS. Participants should be treated within 5 days of randomization.
Dispense pembrolizumab via IWRS				X	X				Upon confirmation of eligibility, site to dispense study intervention via IWRS.
Administer pembrolizumab				X	X				
Dispense LY3537982 via IWRS				X	X				Upon confirmation of eligibility, site to dispense study intervention via IWRS.
Dispense LY3537982 to participant				X	X				
Participant returns LY3537982					X				
Assess LY3537982 compliance					X				

**Part G**	**Prescreening**	**Screening**		**Treatment Period Cycle = 21 days**		**Posttreatment**			**Comments**
				Cycle 1	Cycle 2-n	EOT[a]	Safety Follow-Up[b]	Long-Term Follow-Up[c]	
Relative Day within Dosing Cycle		≤ 28	≤ 14	1	1	(± 7 days)	28 (±7 days)	Every 3 months (±4 weeks)	Screening: Procedures may be conducted over more than 1 day as long as all activities are completed within the allowable visit tolerance.
Visit Window (Days)		≤ 28		±1	±1 Cycle 2, ±3 Cycle 3+				
Visit Detail								T	T: Telephone Visit, if no study procedures are scheduled.
Poststudy treatment and anticancer therapy information							X	X	

[a] An EOT visit within 7 days of the last dose of LY3537982 or the decision to terminate treatment is required, where clinically appropriate.

[b] Safety follow-up begins when the participant and the Investigator agree that the participant will no longer continue study treatment and lasts approximately 28 days (±7 days) after the last dose of study drug.

[c] Long-term follow-up occurs every 3 months (±4 weeks) for the first 2 years after the last dose of study drug and every 6 months thereafter and begins when safety follow-up period is completed and continues until death or study completion. In all cases, no follow-up procedures will be performed for a participant who withdraws informed consent/assent unless he or she has explicitly provided permission and consent/assent.

[d] Participants without qualifying local *KRAS* and/or PD-L1 results who do not have sufficient tumor tissue available for testing at a Sponsor-designated laboratory may undergo a fresh tumor biopsy during prescreening if it is considered safe to perform. Participants with qualifying local *KRAS* and PD-L1 results who do not have sufficient tumor tissue available for biomarker assessments may undergo a fresh tumor biopsy during screening, if it is considered safe to perform; if previously collected tissue is not available and a fresh tumor biopsy cannot be performed, the participant may still be eligible to enroll upon review and approval by the Sponsor. An optional tissue biopsy may be collected at time of progression if it can be safely performed prior to the start of new anticancer therapy. If disease constituting progression is identified, but the participant is otherwise stable and will continue study treatment beyond progression, the participant may undergo an optional biopsy at the time progression is identified and/or following final discontinuation of study drug. The purpose of these tissue biopsies is to evaluate tumor changes that may have resulted from treatment. For biopsies performed in the setting of disease progression, please contact the Sponsor to inform them of the planned biopsy, and whether therapy will be continued beyond progression or discontinued (EOT).

Table 21: Sampling Schedule for PK for Participants in Part G – Dose Optimization

Cycle	Day	Time after LY3537982 dose (hours)[a]	LY3537982
1	1	[redacted]	X
			X
			X
			X
2	1	Predose	X
		[redacted]	X
			X
4	1		X

[a] Time after dose starts after the participant has ingested the [redacted]

7.1.2 Continued Access Schedule of Assessments

The continued access period will begin after trial completion and will end at End of Study (Section 3.3). Continued access will follow the Schedule of Activities in Table 22. See Section 5.6 for additional details.

Table 22: Continued Access Schedule of Assessments

Visit	Continued Access Trial Treatment<br>501-5XX	SFU<br>901	Notes
**Procedure**			
AE Collection	X	X	• Per CTCAE v5.0, for post follow-up, the Investigator should only collect SAEs related to trial regimen or protocol procedures.<br>• Collect throughout continued access.<br>• See Sections 7.5.6 and 7.5.7 for AE recording and SAE reporting, respectively.
Administer trial intervention	X		• See Section 5.0 for trial intervention administration details and guidelines.

Safety follow-up for continued access begins when the participant and the Investigator agree that the participant will no longer continue treatment in the continued access period and lasts approximately 28 days. In all cases, no follow-up procedures will be performed for a participant who withdraws informed consent unless he or she has explicitly provided permission and consent. See also Section 5.6.

7.2 Enrollment

Enrollment procedures will be conducted in accordance with the SoA tables (Section 7.1). Patients who meet eligibility criteria outlined in Section 4.0 will be enrolled in this study. A patient may be enrolled in either dose escalation or dose expansion, but not both. The Investigator may repeat qualifying laboratory tests and vital signs/ECGs prior to enrollment if a nonqualifying finding is considered an error, and/or if an acute finding is likely to meet eligibility criteria on repeat testing.

A representative from the investigational site will contact the Sponsor or designee when a potential study candidate is identified and a participant number (which will consist of a 4-digit site number followed by a sequential accession number) will be assigned that will be used throughout both Screening and study participation. Once screening procedures have been completed for the patient, a completed enrollment form will be submitted to the Sponsor or designee in order to confirm eligibility.

The enrollment form will be returned to the site with the cohort assignment (if applicable) and must be received by the site before treatment may commence. Refer to the Study Manual for enrollment form, contact numbers, and other details of enrollment.

7.3 Efficacy Assessments

Tumor assessments will be performed for each participant at the times shown in the SoA (Section 7.1).

RECIST v1.1 (Eisenhauer et al. 2009) will be applied as the primary criteria for assessment of systemic tumor response and date of disease progression. For assessment of intracranial response, RECIST v1.1 will be modified (mRECIST v1.1) to allow assessment of at least 1 and up to 5 intracranial target lesions at least 5 mm maximum diameter each (or at least twice the slice thickness if the slice is > 2.5 mm). The method of tumor assessment used at baseline must be used consistently throughout the study. Local tumor imaging (Investigator assessment with site radiological reading) will be used.

CT scans, including spiral CT, are the preferred methods of measurement (CT scan thickness recommended to be ≤ 5 mm); however, MRI is also acceptable in certain situations, such as when body scans are indicated or if there is a concern about radiation exposure associated with CT. IV and oral contrast are required unless medically contraindicated or inconsistent with local regulations and/or practice.

The CT portion of a PET-CT scan may be used as a method of response assessment if the site can document that the CT is of identical diagnostic quality to a diagnostic CT (with IV and oral contrast). A PET scan alone or as part of a PET-CT may be performed for additional analyses but cannot be used to assess response according to RECIST v1.1.

Efficacy assessments include tumor evaluation every 6 weeks after treatment initiation through Week 24, and then approximately every 9 weeks thereafter, consistent with disease-defined criteria. Confirmatory response evaluations may be conducted 4 weeks (± 7 days) after the first tumor evaluation that shows a PR or better, if consistent with local institution and regulatory authority requirements. Participants who discontinue treatment without documented progression should continue to be assessed per the above schedule until disease progression is observed or the participant starts subsequent anticancer therapy.

All participants are required to undergo chest/abdomen/pelvis imaging at baseline and subsequent serial scans at disease assessment timepoints. All scans will be collected and stored at a central facility and may be reviewed by a central reviewer for assessment during the study. Response will be assessed per RECIST v1.1 requirements (Eisenhauer et al. 2009).

Finally, all participants will enter LTFU for confirming disease progression if not occurring on treatment, subsequent anticancer therapy(ies), and survival.

Baseline disease assessment with radiographic tumor measurements using CT or MRI of the chest, abdomen, pelvis, or any other areas with suspected disease involvement must occur within 28 days of C1D1. During all phases, brain imaging is required at baseline for all participants with NSCLC and as clinically indicated for other tumor types (MRI preferred, CT with contrast is acceptable if MRI contraindicated). For each modality, IV and oral contrast should be utilized (chest CT does not require IV contrast) unless there is a clear contraindication (e.g., decreased renal function or allergy that cannot be addressed with standard prophylactic treatments). In the absence of known or suspected disease involvement, head and neck CT/MRI scans are not required for malignancies other than those originating in the head and neck region. Other areas of scanning may also differ depending on disease type.

Postbaseline scans should be performed every 6 weeks (±7 days) for 6 months and every 9 weeks (±7 days) thereafter, including imaging of the chest, abdomen, and pelvis, using the same modality(ies) as used for baseline imaging assessment until PD, withdrawal of consent, or initiation of (a) new anticancer therapy(ies). Additionally, any studies performed at baseline that are positive for sites of disease should be repeated at all postbaseline assessments. Additional studies can also be performed as clinically indicated.

In addition, Investigators may conduct an initial tumor evaluation on Cycle 2 Day 1 (±7 days) and a confirmatory tumor evaluation a minimum of 4 weeks (i.e., 28 days) after the first tumor evaluation that shows a CR or PR by RECIST v1.1 (or modified RECIST v1.1, as appropriate to tumor type or cohort), if consistent with local regulatory authority requirements. In addition, an initial postbaseline assessment on Cycle 2 Day 1 (±7 days) is encouraged if consistent with

regulatory guidelines. If a scan is performed on Cycle 2 Day 1, the next scan should continue according to the schedule above (beginning at Cycle 3 Day 1). All scans will be collected and stored at a central facility to permit central reviewer assessment.

A blinded independent data review may be considered in order to confirm response rate across the study cohorts. Please see the Site Imaging Manual for guidelines on how the various imaging studies should be performed.

BOR will be categorized as CR, PR, SD, PD, or not-evaluable according to RECIST v1.1 criteria. To qualify as a response other than PD or unknown, a participant's BOR must be observed at least 6 weeks postbaseline. A PD observed prior to that point will qualify as the participant's BOR. Otherwise, if a participant does not have an adequate disease assessment, per RECIST v1.1 criteria, a BOR of unknown will be assigned.

ORR is the proportion of participants who achieved a CR or PR out of all participants treated. Tumor responses will be measured and recorded using RECIST v1.1 guidelines (Eisenhauer et al. 2009). To confirm objective responses, all lesions should be radiologically assessed, and the same radiologic method used for the initial response determination should be repeated at least 4 weeks following the initial observation of an objective response, using the sample method that was used at baseline.

DCR is the proportion of enrolled participants who have a BOR of confirmed CR, confirmed PR, or SD and will be summarized. Best response is determined from a sequence of responses assessed. Two objective status determinations of CR before progression are required for a best response of CR. Two determinations of PR or better before progression, but not qualifying for a CR, are required for a best response of PR.

DOR is defined as the time from the date measurement criteria for CR or PR (whichever is first recorded) are first met until the first date that disease is recurrent or objective progression is observed, per RECIST v1.1 criteria, or the date of death from any cause in the absence of objectively determined disease progression or recurrence. For each participant who is not known to have died or to have had a progression of disease as of the data-inclusion cutoff date, DOR will be censored at the date of last objective response assessment prior to the date of any subsequent systemic anticancer therapy.

PFS will be defined as the time from the first dose date to the first observation of a PD overall response or death without documented disease progression per RECIST v1.1 criteria. Participants not known to have either of these events will be censored. A full description of censoring rules is provided in Section 8.4.3.

OS will be defined as the time from the first dose date to death from any cause. Participants alive at the end of the study, who have withdrawn from the study, or who are lost to follow-up will be censored on their last known alive date.

Intracranial ORR and Intracranial DOR are defined in Section 8.4.3 along with further definitions of the efficacy endpoints.

[redacted]

CCI

[redacted]

CCI

[redacted]

CCI [redacted]

7.4 Safety Assessments

For each participant, ECGs, vital signs, laboratory tests, and other tests should be collected as shown in the SoA (Section 7.1). Results from any clinical laboratory test analyzed by a local laboratory (refer to Section 9.2, Appendix 2) will be provided to investigative sites by Sponsor or its designee.

Refer to Section 7.5 for details on the recording of AEs.

7.4.1 Electrocardiograms

ECGs will be 12-lead assessments obtained at the time points indicated in the SoA (Section 7.1; single ECG at baseline [Screening], SFU and Phase 1b dose expansion, and triplicate ECG for other assessments, including Phase 1b Part G). Participants must be supine for 5 to 10 minutes in a quiet environment before ECG collection and remain supine but awake during ECG collection. After enrollment, if a clinically significant increase in the QT/QTcF interval from baseline or other clinically significant change from baseline is identified, the participant will be assessed by the Investigator for symptoms (e.g., palpitations, near syncope, or syncope) and to determine whether the participant can continue in the study. The Investigator or qualified designee is responsible for determining if any change in participant management is needed and must

document his/her review of the ECG printed at the time of evaluation from at least 1 of the replicate ECGs from each time point.

All digital ECGs will be electronically transmitted to a central ECG laboratory designated by Sponsor for storage.

7.4.2 Clinical Safety Laboratory Assessments

Local laboratories will be utilized for routine laboratory tests, e.g., blood chemistries from serum or plasma, hematology, and urinalysis. Additional guidance regarding testing and handling and processing of samples for central assessment is provided in the Laboratory Manual.

See Section 9.2 for the list of clinical laboratory tests to be performed and refer to the SoA (Section 7.1) for the timing and frequency.

The Investigator must review the laboratory report, document this review, and record any clinically relevant changes occurring during the study in the AE section of the CRF. The laboratory reports must be filed with the source documents. Clinically significant abnormal laboratory findings are those which are not associated with the underlying disease, unless judged by the Investigator to be more severe than expected for the participant's condition.

All laboratory tests with values considered clinically significantly abnormal during participation in the study or until the completion of the SFU Visit after the last dose of study intervention should be repeated until the values return to normal or baseline or are no longer considered clinically significant by the Investigator or Medical Monitor.

- If such values do not return to normal/baseline within a period of time judged reasonable by the Investigator, the etiology should be identified, and the Sponsor notified.
- All protocol-required laboratory assessments, as defined in Section 9.2, must be conducted in accordance with the Laboratory Manual and the SoA (Section 7.1).

If there is an abnormal laboratory value or abnormal value for any other diagnostic or screening test (e.g., blood pressure increased, neutrophils decreased, etc.) and it is known to be related to a disease diagnosis (e.g., hypertension, neutropenia, etc.), this should be reported in the CRF as an AE. Do not enter the test abnormality, enter the disease diagnosis or categorical term. If laboratory values from non–protocol-specified laboratory assessments performed at the institution's local laboratory require a change in participant management or are considered clinically significant by the Investigator (e.g., SAE or AE, or dose modification), then the event(s) must be reported in the CRF as AE(s).

7.4.2.1 Hepatic Safety Monitoring and Suggested Management

If one or more of the conditions listed in Table 24 occur, liver testing (Section 9.4), including ALT, AST, ALP, TBL, direct bilirubin, GGT, and creatine phosphokinase, should be repeated within 2 to 4 days to confirm the abnormality and to determine if it is increasing or decreasing. See Section 9.9.5 to identify laboratory values that must be recorded as ECIs.

Table 24: Suggested Management for Hepatic Safety Monitoring

If a participant with baseline results of…	develops the following elevations
ALT or AST < 1.5 × ULN	ALT or AST ≥ 5 × ULN or<br>ALT or AST ≥ 3 × ULN concurrent with TBL ≥ 2 × ULN
ALT or AST ≥ 1.5 × ULN	ALT or AST ≥ 3 × baseline or<br>ALT or AST ≥ 2 × baseline concurrent with TBL ≥ 2 × ULN

If the abnormality persists or worsens, clinical and laboratory monitoring and evaluation for possible causes of abnormal liver tests should be initiated by the Investigator in consultation with the Sponsor-designated Medical Monitor. At a minimum, this evaluation should include physical examination and a thorough medical history, including symptoms, recent illnesses (e.g., heart failure, systemic infection, hypotension, or seizures), and history of concomitant medications (including over the counter, herbal and dietary supplements, history of alcohol drinking, and other substance abuse). In addition, the evaluation should include a blood test for PT-INR; serological tests for viral hepatitis A, B, C, E, autoimmune hepatitis; and an abdominal imaging study (e.g., ultrasound or CT scan).

Based on the participant's history and initial evaluation results, further testing should be considered, in consultation with the Sponsor-designated Medical Monitor, including tests for hepatitis D virus, cytomegalovirus, Epstein-Barr virus, acetaminophen levels, acetaminophen protein adducts, urine toxicology screen, Wilson's disease, blood alcohol levels, urinary ethyl glucuronide, and serum phosphatidylethanol. Based on the circumstances and the Investigator's assessment of the participant's clinical condition, the Investigator should consider referring the participant for a hepatologist or gastroenterologist consultation, magnetic resonance cholangiopancreatography, endoscopic retrograde cholangiopancreatography, cardiac echocardiogram, and/or a liver biopsy.

In the event of observed toxicities that may be associated with more than one agent (e.g., hepatic toxicity or unexpected hematologic toxicity), a temporal association should be evaluated to aid in assessment of drug relatedness and dose modification. Other potentially reversible risk factors for the AE should be identified and addressed as appropriate. Refer to Section 5.5 for dose modification guidelines.

7.4.2.2 Guidance for Monitoring Renal Function

7.4.2.2.1 Pembrolizumab Cohorts

Pembrolizumab does not require dose adjustment for participants with mild or moderate renal impairment and has not been studied in participants with severe renal impairment. Nephritis has been reported in participants receiving pembrolizumab and cisplatin (Cohort B9) and can cause severe renal toxicity, including acute renal failure. Participants should be monitored for changes in renal function according to the SoA, and other causes of renal dysfunction excluded. Refer to Table 33 in Section 9.9.2 for management of suspected pembrolizumab-associated renal toxicity and to the cisplatin Patient Information Leaflet, Package Insert, or Summary of Product Characteristics for management of cisplatin-associated renal toxicity.

7.4.2.3 Guidance for ILD/Pneumonitis

7.4.2.3.1 Cetuximab

ILD/pneumonitis was identified as an adverse drug reaction for cetuximab. ILD/pneumonitis, including 1 fatality, occurred in < 0.5% of 1570 participants receiving cetuximab clinical trials. Monitor participants for signs and symptoms of pulmonary toxicity. Interrupt or permanently discontinue cetuximab for acute onset or worsening of pulmonary symptoms. Permanently discontinue cetuximab for confirmed ILD/pneumonitis in accordance with the approved product label.

7.4.2.3.2 Pembrolizumab

ILD/pneumonitis has been identified as an adverse drug reaction for pembrolizumab in clinical trials. Refer to Table 33 in Section 9.9.2 for further details.

Ask participants to report any new or worsening pulmonary symptoms such as dyspnea, cough, and fever and investigate and treat as per local clinical practice (including corticosteroids as appropriate). If ILD/pneumonitis is suspected, investigations may include imaging, such as high-resolution CT, broncheoalveolar lavage, and biopsy as clinically indicated.

Dose adjustments for combination with pembrolizumab will be determined by the Investigator in accordance with Table 33 in Section 9.9.2. Permanently discontinue pembrolizumab in cases of severe (Grade 3 or 4) ILD/pneumonitis and withhold pembrolizumab in case of Grade 2 ILD/pneumonitis.

7.4.2.4 Rash

7.4.2.4.1 Cetuximab Cohorts

Participants receiving cetuximab should be monitored for dermatologic toxicities and infectious sequalae. Participants should be instructed to limit sun exposure during cetuximab therapy. Dose modifications of cetuximab should follow the guidance provided in the approved product label. The severity of acneiform rash is graded according to the CTCAE Version 5.0 definition of rash/desquamation.

7.4.2.4.2 Pembrolizumab Cohorts

Rash has been identified as an adverse drug reaction for pembrolizumab in clinical trials. Refer to Table 33 in Section 9.9.2 for further details. Participants should be instructed to limit sun exposure during pembrolizumab treatment. Dose adjustments for combination with pembrolizumab will be determined by the Investigator in accordance with Table 33 in Section 9.9.2. Discontinue pembrolizumab in cases of severe (Grade 3 or 4) rash.

7.4.2.5 Electrolyte Imbalance

7.4.2.5.1 Cetuximab Cohorts

In 365 participants receiving cetuximab in study CA225-025, the incidence of hypomagnesemia was 55%, both overall and severe (Grades 3 and 4). Hypomagnesemia and accompanying electrolyte abnormalities can occur days to months after initiating cetuximab. Accordingly, participants will be periodically monitored for hypomagnesemia and concomitant hypocalcemia and hypokalemia, during and following the completion of cetuximab therapy. Monitoring should continue for a period of time commensurate with the half-life and persistence of the agent (i.e., 8 weeks). Replace electrolytes as necessary.

7.4.2.6 Infusion Reactions

7.4.2.6.1 Cetuximab Cohorts

Infusion reactions may occur during a cetuximab infusion or may be delayed until any time after the infusion. As a routine precaution, participants enrolled in this study will be observed closely for any potential AEs by the medical staff for the duration of a cetuximab infusion and until at least 1 hour after the end of the initial 400 mg/m^2 infusion in an area with resuscitation equipment and medications necessary for advanced life support and cardiopulmonary resuscitation, such as bronchodilators, vasopressor agents (e.g., epinephrine), oxygen, glucocorticoids, antihistamines, IV fluids, etc. Infusion reactions will be defined according to the CTCAE Version 5.0 definition of allergic reaction/hypersensitivity.

7.4.2.6.2 Pembrolizumab Cohorts

Severe or life-threatening infusion-related reactions may occur during pembrolizumab infusion. As a routine precaution, participants enrolled in this study should be observed for signs and

symptoms of infusion-related reactions including rigors, chills, wheezing, pruritus, flushing, rash, hypotension, hypoxemia, and fever. The infusion may be interrupted or slowed for mild (Grade 1) or moderate (Grade 2) reactions. The infusion should be stopped and permanently discontinued for severe (Grade 3) or life-threatening (Grade 4) infusion-related reactions. Refer to Section 9.9 for further details.

7.4.2.7 Immune-Mediated Reactions

7.4.2.7.1 Pembrolizumab Cohorts

Severe or fatal immune-mediated adverse reactions may occur in any organ system or tissue during pembrolizumab infusion. As a routine precaution, participants enrolled in this study should be monitored for early identification and management of immune-mediated adverse reactions including the following: immune-mediated pneumonitis, immune-mediated colitis, immune-mediated hepatitis, immune-mediated endocrinopathies, immune-mediated nephritis with renal dysfunction, immune-mediated dermatologic adverse reactions, and solid organ transplant rejection. Liver enzymes, creatinine, and thyroid function should be evaluated at baseline and periodically during treatment. Refer to Section 9.9 for further details.

7.5 Adverse Events and Serious Adverse Events

An AE is any unfavorable medical occurrence in a participant administered an investigational product, which does not necessarily have a causal relationship with the treatment. An AE can therefore be any unfavorable and unintended sign, symptom, or disease temporally associated with the use of the investigational product, whether or not considered related to the investigational product.

Medication error, misuse, or abuse of investigational medicinal product(s) are not formally considered AEs but should be recorded in the CRF; associated clinical sequelae are considered AEs and should be recorded as such.

Investigators are responsible for monitoring the safety of participants who have entered this study and for alerting Sponsor or its designee to any event that seems unusual, even if this event may be considered an unanticipated benefit to the participant.

The Investigator is responsible for the appropriate medical care of participants during the study.

Investigators must document their review of each laboratory safety report.

Laboratory test abnormalities considered by the Investigator to be clinically relevant should be reported in the eCRF as an AE.

The Investigator remains responsible for following up on, through an appropriate health care option, AEs that are serious or otherwise medically important, considered related to the investigational product or the study, or that caused the participant to discontinue the investigational product before completing the study. The participant should be followed until the

event resolves, stabilizes with appropriate diagnostic evaluation, or is otherwise explained. The frequency of follow-up evaluations of the AE is left to the discretion of the Investigator.

Lack of drug effect is not an AE in clinical studies because the purpose of the clinical study is to establish treatment effect.

After the ICF is signed, study site personnel will record via CRF the occurrence and nature of each participant's pre-existing conditions, including clinically significant signs and symptoms of the disease under treatment in the study. In addition, site personnel will record any change in the condition(s) and any new conditions. Investigators should record their assessment of the potential relatedness of each event to protocol procedure or investigational product via CRF**.** All events that occur prior to the first dose are considered medical history and will be recorded as such in the eCRF unless the event develops or worsens due to study-related procedures, in which case it will be recorded as an AE.

If a participant's investigational product is discontinued as a result of an AE, study site personnel must report this to Sponsor or its designee via CRF, clarifying, if possible, the circumstances leading to any dosage modifications or discontinuations of treatment.

7.5.1 Severity of Adverse Events

The Investigator will grade the severity of each AE using, when applicable, the NCI CTCAE v5.0. In the event of an AE for which no grading scale exists, the Investigator will classify the AE as mild, moderate, severe, life-threatening/debilitating, or fatal, as defined in Table 25 below.

Table 25: Adverse Event Severity Classification

Severity Classification	Description
Grade 1	Mild; asymptomatic or mild symptoms; clinical or diagnostic observations only; intervention not indicated. Not interfering with normal activities.
Grade 2	Moderate; minimal, local, or noninvasive intervention indicated; limiting age-appropriate instrumental ADL.
Grade 3	Severe or medically significant, but not immediately life-threatening; hospitalization or prolongation of hospitalization indicated; disabling; limiting self-care ADL.
Grade 4	Life-threatening consequences[a]; urgent intervention indicated.
Grade 5	Death related to AE.

[a] A life-threatening serious adverse event is an AE that places the participant at an immediate risk of death. It does not include an adverse reaction that, had it occurred in a more severe form, might have caused death. Laboratory values which meet Grade 4 CTCAE severity based on numeric value may not necessarily meet the life-threatening seriousness criteria.

7.5.2 Relatedness of Adverse Events

The Investigator will categorize each AE as to its potential relationship to underlying disease, other medical conditions or concomitant medications using the categories of Yes (causally related) and No (not related) as defined below. The assessment of the relationship of an AE to the underlying disease, other medical conditions, or concomitant medications is a clinical decision based on all available information at the time.

Yes: The time course between the occurrence or worsening of the AE and the underlying disease, other medical conditions, or concomitant medications is consistent with a causal relationship and another cause is considered to be unlikely.

No: The time course between the occurrence or worsening of the AE and underlying disease, other medical conditions, or concomitant medications rules out a causal relationship and another cause is considered to be more likely.

The following factors should also be considered:

- Temporal sequence from treatment with the study drugs.
- Preclinical and prior clinical data regarding whether a particular AE could be an effect of the study drug (or class of drug).
- Pharmacology and PK of the investigational products.

7.5.3 Serious Adverse Events

An SAE is any AE from this study that at any dose results in one of the following outcomes:

- Death
- A life-threatening experience (that is, immediate risk of dying)
- Initial or prolonged existing inpatient hospitalization
- Persistent or significant disability/incapacity
- Congenital anomaly/birth defect
- Important medical events that may not be immediately life-threatening or result in death or hospitalization but may jeopardize the participant or may require intervention to prevent one of the other outcomes listed in the definition above. Examples of such medical events include allergic bronchospasm requiring intensive treatment in an emergency room or at home, blood dyscrasias or convulsions that do not result in inpatient hospitalization, or the development of drug dependency or drug abuse.

Any hospital admission with at least one overnight stay will be considered an inpatient hospitalization. For a planned hospitalization or procedure for a preexisting condition that was recorded in the patient's medical history at the time of enrollment, the underlying AE requiring intervention should not be reported as an SAE unless it meets other serious criteria. An

emergency room visit without hospital admission will not be recorded as an SAE under this criterion, nor will hospitalization for a procedure scheduled or planned. Unexpected complications and/or prolongation of hospitalization that occur during elective surgery should be recorded as AEs and assessed for seriousness. Admission to the hospital for social or situational reasons (i.e., no place to stay, live too far away to come for hospital visits) will not be considered inpatient hospitalizations. Planned surgeries and nonsurgical interventions should not be reported as AEs unless the underlying medical condition has worsened during the course of the study.

In the event of an accidental or intentional overdose by a participant, the site staff must immediately inform the study team and the study Medical Monitor. The eCRF must be updated to reflect this information. In the event that the overdose is associated with an SAE, the 2 events should be linked.

All AEs occurring after signing the ICF are recorded in the CRF and assessed for serious criteria. Each AE is to be evaluated for duration, severity, and causal relationship with the investigational product or other factors. The SAE reporting to the Sponsor occurs from the time the participant has signed the ICF through 28 days after last dose of study drug (except for Cohort B4, Cohort B9, and Part G, for which the window is 90 days after the last dose of study drug, or 28 days after the last dose of the study drug if the participant initiates new anticancer therapy), regardless of causality. If an SAE occurs after signing the ICF, but prior to receiving study treatment, it needs to be reported **ONLY** if it is considered reasonably possibly related to protocol-mandated procedure. All SAEs that the Investigator considers related to study drug occurring after the 28-day follow-up period must be reported to the Sponsor.

Study site personnel must alert Sponsor or its designee of any SAE within 24 hours of Investigator awareness of the event via a Sponsor-approved method. If alerts are issued via telephone, they are to be immediately followed with official notification on–study-specific SAE forms. This 24-hour notification requirement refers to the initial SAE information and all follow-up SAE information. Participants with a serious hepatic AE should have additional data collected using the CRF.

The Sponsor must be notified of any participant or participant's partner who becomes pregnant during a clinical study. Pregnancy (during maternal or paternal exposure to investigational product) does not meet the definition of an AE. However, to fulfill regulatory requirements, any pregnancy should be reported following the SAE process to collect data on the outcome for both mother and fetus.

7.5.3.1 Suspected Unexpected Serious Adverse Reactions

A SUSAR refers to an AE that occurs in a clinical trial participant, which is assessed by the Sponsor and or Investigator as being unexpected, serious, and as having a reasonable possibility of a causal relationship with the trial intervention.

7.5.4 Time Period and Frequency for Collecting AEs, SAEs, and Other Potentially Reportable Safety Event Information

All AEs and SAEs are recorded by the site in the CRF/electronic data entry. All SAEs will be recorded and reported to the Sponsor or designee immediately, and under no circumstance should this exceed 24 hours. The Investigator will submit any updated SAE data to the Sponsor within 24 hours of it being available. SAEs, including death, caused by disease progression should not be reported unless the Investigator deems them to be possibly related to LY3537982 or any of the combinations. Disease progression of the malignancy in and of itself is captured as an efficacy assessment and should not be captured as an AE (including fatal AEs) unless the disease progression is assessed as related to study treatment. If toxicities due to PD exist and are new or worsened from baseline, these should be reported as AEs. If a new primary malignancy appears, it will also be considered an AE. The event term of 'death' itself should not be reported as an AE; rather, any AEs associated with the occurrence of death or AEs considered to be Grade 5 in severity (fatal) should be reported.

Investigators are not obligated to actively seek AEs or SAEs after conclusion of the study participation. However, if the Investigator learns of any SAE, including a death, at any time after a participant has been discharged from the study, and he/she considers the event to be reasonably related to the study intervention or study participation, the Investigator must promptly notify the Sponsor.

Care will be taken not to introduce bias when detecting AEs and/or SAEs. Open-ended and nonleading verbal questioning of the participant and/or legal guardian is the preferred method to inquire about AE occurrences.

All AEs or ECIs from signing the ICF through 28 days following cessation of study treatment must be reported by the Investigator.

Note: For Cohort B4, Cohort B9, and Part G only:

- All AEs meeting serious criteria, from signing the ICF through 90 days following cessation of study treatment, or 28 days following cessation of study treatment if the participant initiates new anticancer therapy, whichever is earlier, must be reported by the Investigator.
- All pregnancies and exposure during breastfeeding, from the time of treatment/allocation through 120 days following cessation of study treatment, or 28 days following cessation of study treatment if the participant initiates new anticancer therapy, must be reported by the Investigator.
- Please see Section 9.9.5, Appendix 9, for separate reporting information related to pembrolizumab ECIs.

7.5.5 Follow-Up of AEs, SAEs and ECIs

After the initial AE/SAE report, the Investigator is required to proactively follow up with each participant at subsequent visits/contacts. For all SAEs occurring during the study, the Investigator must submit follow-up reports to the Sponsor regarding the status of the SAE and the participant's subsequent course. All SAEs will be followed until resolution, the event is otherwise explained, until the condition stabilizes or is deemed chronic (in the case of persistent impairment), the participant is lost to follow-up (as defined in Section 7.5), or the participant dies.

7.5.6 Adverse Event Reporting

Prompt notification by the Investigator to the Sponsor of an SAE is essential so that legal obligations and ethical responsibilities towards the safety of participants and the safety of a study intervention under clinical investigation are met.

The Sponsor or its representative is required to report certain study events in an expedited manner to the Food and Drug Administration, the European Medicines Agency's EudraVigilance electronic system according to Directive 2001/20/EC, and to all country Regulatory Authorities where the study is being conducted, according to local applicable regulations.

SUSARs will be reported to the IRB/IEC according to their institutional policy by the Investigator or the Sponsor (or Sponsor's designee) and in accordance with country requirements. As applicable, copies of reports and documentation of IRB/IEC notification and acknowledgement of receipt should be kept in the Investigator's study file.

7.5.7 Serious Adverse Event Reporting—Procedures for Investigators: Initial Report

7.5.7.1 SAE Reporting via an Electronic Data Collection Tool

The primary mechanism for reporting an SAE will be the electronic data collection tool.

If the electronic system is unavailable, then the site will use the SAE paper form (see Section 7.5.7.2) in order to report the event within 24 hours. Contacts for SAE reporting can be found in the Global Patient Safety Clinical Trial SAE Transmission Cover Sheet and Form.

The site will enter the SAE data into the electronic system as soon as it becomes available.

After the study is completed at a given site, the electronic data collection tool will be taken offline to prevent the entry of new data or changes to existing data.

If a site receives a report of a new SAE from a study participant or receives updated data on a previously reported SAE after the electronic data collection tool has been taken offline, then the site can report this information on a SAE paper form (see Section 7.5.7.2) or to the Sponsor or designee by telephone.

7.5.7.2 SAE Reporting via Paper Form

Facsimile transmission of the SAE paper form is the preferred method to transmit this information to the Sponsor or designee. Contacts for SAE reporting can be found in the Global Patient Safety Clinical Trial SAE Transmission Cover Sheet and Form.

Initial notification via telephone does not replace the need for the Investigator to complete and sign the SAE CRF pages within the designated reporting time frame.

7.5.8 Regulatory Reporting Requirements

Prompt notification of an SAE by the Investigator to the Sponsor is essential so that legal obligations and ethical responsibilities toward the safety of participants and the safety of the study interventions under clinical investigation are met.

The Sponsor has a legal responsibility to notify both the local regulatory authority and other regulatory agencies about the safety of a study intervention under clinical investigation. The Sponsor will evaluate the reported SAEs, including confirmation of relatedness and assessment of expectedness. The Sponsor has processes for safety reports for identification, recording, and expedited reporting of SUSARs according to local regulatory requirements. The Sponsor will comply with country-specific regulatory requirements relating to safety reporting to the regulatory authority, IRB/IEC, and investigators.

An Investigator who receives an investigator safety report describing an SAE or other specific safety information (for example, summary or listing of SAEs) from the Sponsor will review and then file it along with the IB and will notify the IRB/IEC, if appropriate according to local requirements.

7.5.9 Pregnancy

Additional requirements for pregnancy testing during and after study intervention are in Section 9.3.

The Investigator is responsible for review of medical history, menstrual history, and recent sexual activity to decrease the risk of inclusion of a woman with an early undetected pregnancy.

7.5.10 Death Events Due to Disease Progression

Events leading to the clinical outcome of death due to progression of study disease that are part of the efficacy analyses for this study will not be reported to Sponsor or its designee as SAEs unless the Investigator believes the event may have been caused by the investigational product. Concurrent SAEs that occur at the time of death must be reported as required.

7.5.11 Complaint Handling

The Sponsor collects product complaints on investigational products and drug delivery systems used in clinical studies in order to ensure the safety of study participants, monitor quality, and to facilitate process and product improvements.

Participants will be instructed to contact the Investigator as soon as possible if he or she has a complaint or problem with the investigational product so that the situation can be assessed.

7.6 Treatment of Overdose

An overdose is only considered an AE if it is associated with a clinically significant resultant event (Section 7.5).

For this study, an overdose of pembrolizumab will be defined as any dose of 1000 mg or greater.

No specific information is available on the treatment of overdose of pembrolizumab. In the event of overdose, the participant should be observed closely for signs of toxicity. Appropriate supportive treatment should be provided if clinically indicated.

For all other study treatments, refer to Section 6.8 of the LY3537982 IB and/or Product Label for intervention or comparator for available information on the signs, symptoms, and treatment of overdose.

In the event of an accidental or intentional overdose by a participant, the site staff must immediately inform Clinical Safety and the Medical Monitor. The CRF must be updated to reflect this information. In the event that the overdose is associated with an SAE, the 2 events should be linked. In the event of an SAE associated with an overdose, an SAE report form must be completed describing the AE and the overdose details.

7.7 Pharmacokinetics

At the visits and times specified in the SoA (Section 7.1), venous blood samples will be collected to determine the plasma concentrations of LY3537982, and serum concentration of pembrolizumab and cetuximab. Differences from the time specified in the protocol are not considered protocol deviations as long as samples are collected, and accurate dates and times are recorded in a timely manner on the appropriate forms.

A maximum of 5 samples, in addition to those shown in the SoA (Section 7.1), may be collected at additional time points during the study if warranted and agreed upon between both the Investigator and Sponsor. Instructions for the collection and handling of blood samples will be provided by the Sponsor. The actual date and time (24-hour clock time) of each sampling will be recorded.

Blood samples will be collected on all participants and analyzed as dictated by the Sponsor at an approved laboratory. Plasma concentrations of LY3537982 will be assayed using a validated LC-MS/MS method. Serum concentrations of pembrolizumab and cetuximab will be assayed using a validated enzyme-linked immunosorbent assay method.

Urine samples will be collected from participants in Cohort B1 for determination of LY3537982 concentrations in urine on C1D1. Total urine output from the time of administration of LY3537982 up to the last C1D1 PK collection will be collected, pooled, and refrigerated. Urine collection for determination of LY3537982 should cease after the last C1D1 PK collection. For the collection period, the total urine volume will be recorded, and 2 approximately 10 mL samples will be stored frozen. The remaining urine will be discarded. Instructions for the collection and handling of the urine samples will be provided by the Sponsor.

Urine samples will be analyzed at a laboratory designated by the Sponsor. Urine concentrations of LY3537982 will be quantified using an LC-MS/MS assay. The remaining urine samples may be used for exploratory metabolism work. Results from exploratory metabolism work will not be included in the final integrated study report.

Bioanalytical samples collected to measure investigational product concentration and metabolism and/or protein binding will be retained for a maximum of 1 year following last participant visit for the study. During this time, blood samples remaining after the bioanalyses may be analyzed for exploratory drug metabolism work and other PK/pharmacodynamics work, as deemed appropriate by the Sponsor. The results of such exploratory metabolism work may only be included in the CSR if deemed appropriate by the Sponsor or may be reported in a separate exploratory metabolism report. The assessment of renal concentration of LY3537982 is an exploratory assessment and that failure to collect or analyze a urine sample is not a deviation.

7.8 Pharmacodynamics

Samples collected to measure pharmacodynamics biomarkers will be identified by the participant number (coded) and retained at a facility selected by Sponsor or its designee for a maximum of 15 years following last participant visit for the study at a facility selected by Sponsor or its designee. See Section 7.10 for biomarker information.

7.9 Genetics

A whole blood sample will be collected for pharmacogenetic analysis as specified in the SoA (Section 7.1) where local regulations allow.

Samples will not be used to conduct unspecified disease or population genetic research either now or in the future. Samples will be used to investigate variable response to LY3537982, to investigate genetic variants thought to play a role in cancer, and to determine whether genetic alterations identified in tumor samples are somatic or germline variants. Assessment of variable response may include evaluation of AEs or differences in efficacy. The methods used in this

study to perform genetic research are not clinically validated to detect germline variants, and therefore, no clinical conclusions can be derived from them. As such, subject to local regulations, no incidental findings will be reported to the participants participating in genetic research.

All samples will be coded with the participant number. These samples and any data generated can be linked back to the participant only by the Investigator site personnel.

Samples will be retained at a facility selected by Sponsor or its designee for a maximum of 15 years after the last participant visit for the study, or for a shorter period if local regulations and/or ERBs/IRBs impose shorter time limits. This retention period enables use of new technologies, response to regulatory questions, and investigation of variable response that may not be observed until later in the development of LY3537982 or after LY3537982 becomes commercially available.

Molecular technologies are expected to improve during the 15-year storage period and therefore cannot be specifically named. However, existing approaches include whole genome or exome sequencing, genome wide association studies, and candidate gene studies.

7.10 Biomarkers

Biomarker research is performed to address questions of relevance to drug disposition, target engagement, pharmacodynamics, mechanism of action, variability of participant response (including safety), resistance mechanism, and clinical outcome. Sample collection is incorporated into clinical studies to enable examination of these questions through measurement of biomolecules including DNA, RNA, proteins, lipids, and other cellular elements.

Blood, plasma, and tumor tissue samples for biomarker research will be collected at the times specified in the SoA (Section 7.1) where local regulations allow. Guidance on sample requirements and collection is provided in the Laboratory Manual.

Samples will be used for research on the drug target, disease process, variable response to study treatment, pathways associated with study treatment, mechanism of action of study treatment, and/or to develop research methods or validate diagnostic tools or assays related to cancer.

Archival tumor tissue (preferably from the most recent biopsy) should be submitted if available. Formalin-fixed paraffin-embedded tissue blocks or approximately 20 × 5 μm unstained slides, with verification of at least 20% tumor content, should be provided. Fewer than 20 slides will likely be acceptable but should be discussed with the Sponsor. Participants who do not have adequate archival tumor tissue available may undergo an optional fresh tumor biopsy prior to treatment, if it is considered safe to perform. If archival tissue is not available and a fresh tumor biopsy cannot be performed, the participant will likely still be eligible to enroll upon review and approval by the Sponsor. The samples may be used for confirmation of *KRAS* G12C mutation and detection of additional genomic variants.

For Part G, patients must have known *KRAS* G12C mutation and PD-L1 status prior to randomization. Participants without local test results for a *KRAS* G12C mutation and/or PD-L1 status may submit tumor tissue for testing of *KRAS* and/or PD-L1 at a Sponsor-designated laboratory to determine eligibility. If slides are submitted for PD-L1 testing, the slide cut must be within 5 months of the estimated first day on study intervention, and date of sectioning must be provided in the requisition form. Refer to the Laboratory Manual for additional sample requirements.

An optional tissue biopsy may be collected at time of progression if it can be safely performed prior to the start of new anticancer therapy and participant provides informed consent. If disease constituting progression is identified, but the participant is otherwise stable and will continue study treatment beyond progression, the participant may undergo an optional biopsy at the time progression is identified and/or following final discontinuation of study drug. The purpose of these tissue biopsies is to evaluate tumor changes that may have resulted from treatment. For biopsies performed in the setting of disease progression, please contact the Sponsor to inform them of the planned biopsy, and whether therapy will be continued beyond progression or discontinued (EOT).

The de-identified pathology report accompanying tissue are requested and the most recent report(s) describing *KRAS* G12C (and other) alteration analysis and PD-L1 (Part G only) status should be submitted to the Sponsor or its designee. These reports must be coded with the participant number. Personal identifiers, including the participant's name and initials, must be removed from the institutional pathology report prior to submission. Archival blocks will be sectioned and returned to the study site upon request. Slides and tissue samples collected on-study will not be returned. Sponsor has a right to retain a portion of the submitted tissue.

All samples will be coded with the participant number. These samples and any data generated can be linked back to the participant only by the Investigator site personnel.

Samples will be retained at a facility selected by Sponsor or its designee for a maximum of 15 years after the last participant visit for the study, or for a shorter period if local regulations and ERBs impose shorter time limits. This retention period enables use of new technologies, response to regulatory questions, and investigation of variable response that may not be observed until later in the development of LY3537982 or after LY3537982 becomes commercially available.

Technologies are expected to improve during the 15-year storage period and therefore cannot be specifically named. Existing approaches, including mutation profiling, copy number variability analysis, gene expression assays, and/or immunohistochemistry, may be performed on these tissue samples to assess potential associations between these biomarkers and clinical outcomes. These results will not be disclosed to participants since this research is considered exploratory.

It is possible that biomarker data for participants in the study have already been generated from samples that were collected and analyzed prior to enrolling in this study. This may include data

generated from genetic analyses. If available, these data may be requested from medical records for use in the research described in Sections 7.9 and 7.10.

7.11 Intraparticipant Dose Escalation

Intraparticipant dose escalation may be permitted following completion of the 21-day DLT period in Cycle 1 or after at least 1 scan (Cohort B8, [redacted]). Requests for intraparticipant dose escalation require documented Sponsor approval prior to treating a participant with a new dose.

If a participant in Phase 1a is dose escalated, procedures should continue as detailed in SoA Table 13, and in addition, on Days 1 and 8 of the first cycle on the new dose, additional procedures should be performed as detailed in SoA Table 14.

Additional PK assessments are not required for intraparticipant dose escalation in Phase 1b.

If Day 1 of the new dose falls on the same day (± 3 days) of a previous hematology and blood chemistry assessment, it is not necessary to perform a repeat assessment (physical examination, vital signs, and ECGs must still be performed as indicated).

Treatment with a new dose should start on Day 1 (± 3 days) of a cycle unless discussed with the Sponsor.

7.12 Medical Resource Utilization and Health Economics

Health Economics and Medical Resource Utilization parameters will not be evaluated in this study.

8.0 STATISTICAL CONSIDERATIONS

Handling of missing, unused, and spurious data is addressed prospectively in the overall statistical methods described in the protocol and in the SAP, where appropriate. Adjustments to the planned analyses are described in the final CSR.

8.1 Statistical Hypotheses

No formal statistical hypotheses of efficacy and no formal statistical hypothesis testing will be performed for the dose escalation phase. For Phase 1b, the probability of antitumor activity will be evaluated for each cohort/dose optimization arm. Part F is designed to test the hypothesis that LY3537982 monotherapy will provide clinically meaningful antitumor activity in ORR as assessed by IRC.

8.2 Sample Size Determination

This study will consist of 4 parts:

- Phase 1a dose escalation,
- Phase 1b dose expansion,
- Phase 1b dose optimization, and
- Phase 2

Phase 1a and Phase 2 will evaluate monotherapy of LY3537982 and Phase 1b will evaluate both LY3537982 administered as monotherapy and as combination therapy.

8.2.1 Phase 1a

The dose escalation phase will follow an mTPI-2 design to evaluate doses in sequentially opened cohorts of a minimum of [redacted] participants. Each dose cohort may be expanded (i.e., backfilled) up to approximately [redacted] participants to further investigate the safety, PK, and clinical activity, with a total of approximately [redacted] participants enrolled in Phase 1a. The sample size of up to approximately [redacted] participants per dose level has been selected to allow assessment of safety and antitumor activity. It can provide adequate precision for the estimated incidence rate of the following quantities of interest: (1) participants having a specified AE or (2) participants showing a response (CR/PR) or clinical benefit to treatment. With a sample size of up to [redacted] per dose level, example point estimates of incidence rates and corresponding 2-sided Clopper-Pearson 95% CIs are summarized in Table 26.

Table 26: Example Point Estimates of Incidence Rates and Corresponding 2-Sided Clopper-Pearson 95% Confidence Intervals for Sample Size of [redacted]

Number of Responses	Estimated Rate	95% CI (Clopper-Pearson)	
		Lower Limit	Upper Limit
[redacted]			

If supported by the SRC, backfill slots may be moved between dose levels in a total participant number-neutral manner, to allow further exploration of select dose levels.

The actual number of participants to be enrolled in Phase 1a is dependent upon the observed safety profile, which will determine the number of participants per dose cohort, as well as the number of dose escalations required to achieve the MTD/RP2D for further study. It is possible that based on factors such as safety, PK data, and pill burden, dose escalation may not proceed to the highest planned DLs even in the absence of dose-limiting toxicities or definition of an MTD.

8.2.2 Phase 1b

Once an RP2D is established for LY3537982 in Phase 1a, Phase 1b dose expansion will begin. If agreed upon by the Sponsor and SRC, participants may enroll in dose expansion combination cohorts at the CID prior to or after formal RP2D declaration (see Section 3.1.6.1.1). Each of the Phase 1b dose expansion cohorts/dose optimization arm will enroll approximately [redacted] participants (except for monotherapy Cohorts D1, E1, [redacted] which may each enroll up to approximately [redacted] participants, and combination [redacted], which may enroll up to approximately [redacted] participants), with safety continuously monitored using Bayesian toxicity monitoring (Section 8.5). The sample sizes in the Phase 1b expansions have been selected to allow assessments of safety and antitumor activity. The sample size can provide precision for the estimated incidence rate of the following quantities of interest:

- participants having a specified AE or
- participants showing a response (CR/PR) or clinical benefit to treatment.

With a total sample size of [redacted] example point estimates of incidence rates and corresponding 2-sided Clopper-Pearson 95% CIs are summarized in Table 26 [redacted] Table 27 [redacted] and Table 28 [redacted]. The values are provided as a reference for estimation rather than as a basis of any decision criteria. The RP2D may be revised based on the safety data obtained in Phase 1b (Iasonos and O'Quigley 2013).

Table 27: Example Point Estimates of Incidence Rates and Corresponding 2-Sided Clopper-Pearson 95% Confidence Intervals for Sample Size of [redacted]

Number of Responses	Estimated Rate	95% CI (Clopper-Pearson)	
		Lower Limit	Upper Limit
[redacted]			

Table 28: Example Point Estimates of Incidence Rates and Corresponding 2 Sided Clopper-Pearson 95% Confidence Intervals for Sample Size of [redacted]

Number of Responses	Estimated Rate	95% CI (Clopper-Pearson)	
		Lower Limit	Upper Limit
[redacted]			

8.2.3 Phase 2 (Cohort F1)

The sample size of Cohort F1 was calculated using the exact test of a binomial proportion. The expected ORR of Cohort F1 was set to [redacted] based on results available from participants with pancreatic cancer in Phase 1a, and the threshold ORR was set to 17% based on results of treatment with nal-IRIt5-FU/LV in the [redacted]. Using these 2 parameters, the planned sample size of [redacted] was calculated as the minimum sample size to achieve a power of [redacted] or larger with a 2-sided alpha of [redacted].

8.3 Populations for Analyses

For purposes of analysis, the following populations are defined:

Intention-to-treat (ITT) population (Phase 1b, Part G, and Part H only) will include all participants randomly assigned to study intervention, regardless of whether they take any doses of study intervention, or if they took the correct treatment. Participants will be analyzed according to the treatment group to which they were assigned.

Safety Analysis Set will include all participants who received any quantity of study treatment, regardless of their eligibility for the study. The safety evaluation will be performed based on the first dose of study treatment a participant actually received, regardless of the participant's cohort assignment. The Safety Analysis Set will be used for all dosing/exposure and safety analyses.

DLT-evaluable set will include participants who have either completed the DLT observation period (Cycle 1) and received at least 75% of planned doses of LY3537982 and any combination agent(s) in Cycle 1 or have experienced a DLT during Cycle 1. Safety information, including any AEs from DLT-nonevaluable participants for not completing the DLT period and DLT-equivalent AEs from backfill participants, will also be reviewed for dose escalation decisions.

Pharmacokinetic analysis set will include all participants who received at least 1 full dose of LY3537982 and have at least 1 postbaseline evaluable PK sample.

Biomarker analysis set will include the subset of participants from the Safety Analysis Set from whom a valid assay result has been obtained.

8.4 Statistical Analyses

8.4.1 General Statistical Considerations

Statistical analysis of this study will be the responsibility of Sponsor or its designee.

For Phase 1b Part G and Part H, efficacy analyses will be based on the ITT population. For Phase 1a, other parts of Phase 1b and Phase 2, efficacy analyses will be conducted on the Safety Analysis Set within each phase of the study, unless otherwise specified.

All CIs will be given at a 2-sided 95% level, unless otherwise stated. For Phase 1a, data will be summarized by dose cohort; for Phase 1b, data will be summarized by expansion cohort, unless otherwise stated. Safety and efficacy analyses will also be conducted by disease, across monotherapy cohorts, and prior therapy as deemed appropriate.

Any change to the data analysis methods described in the protocol will require an amendment ONLY if it changes a principal feature of the protocol. Any other change to the data analysis methods described in the protocol, and the justification for making the change, will be described in the CSR. Additional exploratory analyses of the data will be conducted as deemed appropriate.

8.4.2 Treatment Group Comparability

8.4.2.1 Participant Disposition

A detailed description of participant disposition will be provided at the end of the study, including a summary of the number and percentage of participants entered into the study, enrolled in the study, treated in the study, and discontinued from the study (overall and by reason for discontinuation).

8.4.2.2 Participant Characteristics

Demographic data are collected and reported to demonstrate that the study population represents the target patient population. A summary of baseline participant and disease characteristics, historical diagnoses, pre-existing conditions, and prior therapies will be reported using descriptive statistics.

In this study, collection of demographic information includes race and ethnicity. The scientific rationale is based on the need to assess variable response in safety and/or efficacy based on race or ethnicity. This question can be answered only if all the relevant data are collected.

8.4.2.3 Concomitant Therapy

A summary of prior and concomitant medications by treatment cohort will be reported.

8.4.2.4 Treatment Compliance

Study treatment compliance will be assessed as the proportion of treatment that is actually taken, relative to what is expected, after accounting for protocol-defined dose adjustments. Study treatment taken will be derived from the difference between the total number of capsules/tablets dispensed and returned over the course of the participant's treatment. The number of cycles received, dose omissions, dose reductions, dose delays, and dose intensity will be summarized for all treated participants by treatment arm.

8.4.3 Efficacy Analyses

For Phase 1b Part G and Part H, efficacy analyses will be based on the ITT population. For Phase 1a, other parts of Phase 1b, and Phase 2, the efficacy analysis will be conducted on the Safety Analysis Set unless otherwise specified. The primary endpoint in Part F and the secondary endpoints in Parts A through H will assess the preliminary antitumor activity of LY3537982 when administered alone or in combination with other investigational agents in participants with advanced solid tumors with *KRAS* G12C mutation using RECIST v1.1 or modified RECIST v1.1, as appropriate to cohort or tumor type (Section 7.3). Antitumor activity will be assessed by Investigator for Phase 1a and Phase 1b and by Investigator and IRC for Phase 2 (Part F), based on:

- ORR
- BOR
- DOR
- TTR
- DCR
- PFS

- OS
- Intracranial ORR (Cohort B8 only)
- Intracranial DOR (Cohort B8 only)

ORR is defined as the number of participants who achieve a BOR of CR or PR divided by the total number of participants evaluated for response analysis, per disease specific response criteria. The ORR, with 95% CI, will be summarized for each study part by DL or expansion cohort.

BOR corresponds to the best response recorded from the start of the study treatment until the earliest of objective progression or start of new anticancer therapy. Confirmations of CR and PR are required.

DOR is measured only in participants with confirmed CR or PR. The start date should be determined by the initial assessment of CR or PR, not the date of confirmation of CR or PR. For participants who are not known to have died or to have had a progression of disease as of the data-inclusion cutoff date, DOR will be censored at the date of last objective response assessment prior to the date of any subsequent systemic anticancer therapy.

TTR is defined as the time from the first dose date/randomization until the date that measurement criteria for CR or PR (whichever is first recorded) are first met, per disease specific response criteria. TTR is measured only in participants with confirmed CR or PR. The end date should be determined by the initial assessment of CR or PR, not the date of confirmation of CR or PR.

PFS is defined as the time from the first dose date/randomization until the first occurrence of documented disease progression, per disease specific response criteria, or death from any cause in the absence of PD. For participants who are not known to have died or progressed as of the data-inclusion cutoff date, time to progression will be censored at the date of the last objective progression-free disease assessment prior to the date of any subsequent systemic anticancer therapy.

DCR is defined as the proportion of participants who achieved a BOR of CR, PR, or SD out of all participants evaluated for response analysis, per disease specific response criteria, will also be summarized.

OS is defined as the time from first dose date/randomization to the date of death due to any cause. For participants who are alive, lost to follow-up, or withdrawn from the study at the time of analysis, OS will be censored at the last date the participant is known to be alive.

Intracranial ORR (Cohort B8 only) is defined as the number of participants who achieve a BOR of intracranial CR or PR divided by the total number of participants who have measurable

intracranial disease at baseline by modified RECIST v1.1 criteria. The intracranial ORR, with 95% CI, will be summarized.

Intracranial DOR (Cohort B8 only) is defined as the time from the first observation of intracranial response until first observation of intracranial disease progression (not considering extracranial disease progression) or death from any cause by modified RECIST v1.1 criteria.

Time-to-event endpoints (DOR, PFS, TTR and OS) will be summarized graphically for each DL/expansion cohort/dose optimization arm using KM curves. Intracranial DOR will be summarized graphically for Cohort B8 using KM curves. Median event times and event rates at various time points with 95% CIs will be estimated based on the KM curve.

8.4.4 Safety Analyses

All participants who receive at least 1 dose of any study therapy will be evaluated for safety and toxicity. A baseline measurement and at least 1 laboratory or other safety-related measurement obtained after the start of study drug may be required for inclusion in the analysis of a specific safety parameter.

Safety analyses will include summaries of the following:

- DLTs at each DL
- AEs, including severity and possible relationship to study drug
- DLT-equivalent AEs, including severity and possible relationship to study drug
- SAEs, including possible relationship to study drug
- AEs leading to dose adjustments
- Discontinuations from study treatment due to AEs or death
- Treatment-emergent abnormal changes in laboratory values
- Treatment-emergent abnormal changes in vital signs and ECGs

8.4.4.1 Safety Review Committees

A Phase 1a SRC will be established to oversee the safety aspects of the Phase 1a dose escalation portion of the study and to render dose escalation decisions. Specifically, the SRC will perform ongoing review and adjudication of SAEs and other safety-related data throughout the conduct of the study. The SRC membership will consist, at a minimum, of the Sponsor's physician responsible for the study/program, clinical research scientist (if applicable), drug safety physician, and the PI (or clinically qualified designee) from each active clinical site contributing participants to that cohort. Additional ad hoc members may be included as deemed necessary. The SRC will be convened for each cohort dose escalation decision or as needed. The SRC will only be required to convene prior to a cohort dose escalation if there is a DLT reported in a cohort. Decisions will be documented in written minutes.

A Phase 1b SRC will be established to oversee the safety aspects of the Phase 1b dose expansion portion of the study and will consist, at a minimum, of the Sponsor's physician responsible for the study/program, clinical research scientist (if applicable), drug safety physician, and the Global PI. For Phase 1b dose expansion, the SRC will be convened for safety lead-in decisions. The Phase 1b SRC will convene at a minimum of every 6 months (or more frequently depending on enrollment or observed safety profile) and perform ongoing review and adjudication of SAEs and other safety-related data throughout the conduct of the study.

A Phase 2 SRC will be established to oversee the safety aspects of the Phase 2 (Part F) portion of the study and will consist, at a minimum, of the Sponsor's physician responsible for the study/program, clinical research scientist (if applicable), drug safety physician, and the Global PI. The Phase 2 SRC will convene at a minimum of every 6 months (or more frequently depending on enrollment or observed safety profile) and perform ongoing review and adjudication of SAEs and other safety-related data throughout the conduct of the study.

The frequency of SRC meetings may be modified during continued access or once all cohorts are fully enrolled, as determined by the SRC.

8.4.5 Pharmacokinetics/Pharmacodynamics Analyses

PK analyses will be conducted on participants who have received at least 1 dose of the study drug and have sufficient samples collected to allow the estimation of LY3537982 PK parameters.

PK parameter estimates for LY3537982 will be calculated using standard noncompartmental methods of analysis. The primary parameters for analysis will be C_{max}, T_{max}, $AUC_{0\text{-}t}$, and $AUC_{0\text{-}\infty}$ of LY3537982. Other noncompartmental parameters, such as $T_{1/2}$, CL/F, and Vz/F, may be reported.

Additional analyses such as population PK analyses may also be conducted if deemed appropriate. Other validated PK software programs (e.g., NONMEM) may be used if

appropriate. The version of any software used for the analysis will be documented, and the program will meet the Sponsor requirements of software validation.

PK/pharmacodynamics analyses may be conducted to explore exposure-response relationships between LY3537982 concentrations in systemic circulation and various PD measures or clinical outcomes.

Plasma concentrations of LY3537982, pemetrexed, cisplatin, and carboplatin, and serum concentrations of pembrolizumab and cetuximab at different time points may be summarized by descriptive statistics.

8.4.6 Other Analyses

8.4.6.1 Subgroup Analyses

Efficacy and safety will be analyzed by disease, prior therapy, and across monotherapy cohorts. Additional subgroup analyses may be performed as deemed appropriate.

8.4.6.2 Biomarker Analyses

Single-marker and/or multi-marker statistical analysis may be performed to explore the association between biomarkers, dose/exposure, and clinical outcomes.

8.5 Interim Analyses

A Bayesian toxicity monitoring rule (Thall et al. 1995) is implemented in all phases and is explained in detail in the following subsections. Operating characteristics are given in Section 9.7.

8.5.1 Phase 1a

In Phase 1a, data will be reviewed for safety on a cohort-by-cohort basis during the study until the MTD/RP2D$_M$ (or the highest DLs if MTD is not reached) is determined. The purpose of these cohort-by-cohort reviews is to evaluate the safety data at each DL and determine if a DLT has been observed that would suggest MTD has been met or exceeded. The SRC will make the determination regarding dose escalation based upon their review of the safety and tolerability data as described in this protocol. A formal review of the safety, including all Phase 1a data, will be performed to conclude the safety profiles and RP2D$_M$.

Considering escalation participants and additional participants backfilled to select DLs, there may be up to approximately [redacted] participants enrolled in each DL in Phase 1a. A Bayesian toxicity monitoring rule is used to ensure that safety is continuously monitored. DLT and DLT-equivalent events are assumed to follow a beta-binomial model. [redacted] prior is assumed for the true DLT rate parameter at each DL. Enrollment into a particular cohort will be temporarily halted if the following boundary is crossed: posterior

probability (true DLT or DLT-equivalent [redacted] is used to be consistent with the upper EI of the mTPI-2 algorithm. This boundary matches the decision rules for the mTPI-2 algorithm except for the number of enrolled participants of [redacted]. When the number of enrollment participants is [redacted], enrollment is halted when the mTPI-2 algorithm would dictate de-escalating the dose. The mTPI-2 decision rules for up to [redacted] participants are given in Section 3.1.5.1. The boundary is expressed in terms of DLTs and DLT-equivalent events (Table 29). For decision rules with fewer than [redacted] participants, see the mTPI-2 table (Figure 5) in Section 3.1.5.1.

Table 29: DLT or DLT-Equivalent Boundaries Using Bayesian Toxicity Monitoring

Number of Enrolled Participants	Pause if Number of DLT or DLT-Equivalent Events
7–8	[redacted]
9–11	
12–14	
15–17[a]	
18–20[a]	
21–22	
23–25	
26–28	
29–31	
32–33	
34–36	
37–39	
40–42	
43–45	
46–47	
48–50	

a. [redacted]

8.5.2 Phase 1b and Phase 2

The Phase 1b cohorts with safety lead-in and Phase 2 (Part F) will be continually monitored at a cohort level using the same beta-binomial toxicity model as in Phase 1a. It is assumed that DLTs and DLT-equivalents follow a beta-binomial Bayesian model with a [redacted] prior on the DLT rate, and enrollment into a cohort will be paused if the [redacted] Additionally, a trial-level safety review will be conducted if the DLT rate aggregated across all expansion cohorts reaches this same threshold.

For Cohort C2, which may enroll up to approximately [redacted] participants, an interim analysis will be conducted after approximately [redacted] participants have been enrolled. Enrollment may continue while the interim analysis is being conducted. Interim analyses may be potentially delayed if confirmation of response is required. An internal review will be conducted by the Phase 1b SRC as well as the study statistician, and Cohort C2 may be closed early if limited efficacy is observed based upon the interim analysis. A beta-binomial Bayesian model with a prior [redacted] will be employed to evaluate the posterior probability of activity. The stopping rules will be used to guide the decision making for Cohort C2 at interim if the posterior probability [redacted] Cohort C2 will be continuously monitored for safety using DLT or DLT-equivalent boundaries as outlined in Table 29.

For Cohort B1, which may enroll up to approximately [redacted] participants, an interim analysis will be conducted after approximately [redacted] participants have been enrolled. Enrollment may continue while the interim analysis is being conducted. Interim analyses may be potentially delayed if confirmation of response is required. An internal review will be conducted by the Phase 1b SRC as well as the study statistician, and Cohort B1 may be closed early if limited efficacy is observed based upon the interim analysis. A beta-binomial Bayesian model with a prior [redacted] will be employed to evaluate the posterior probability of activity. The stopping rules will be used to guide the decision making for Cohort B1 at interim if the posterior probability [redacted] and a reference ORR of [redacted] is selected to align with the monotherapy treatment effect of the most recently established KRAS G12C inhibitor (de Langen et al. 2023). Cohort B1 will be continuously monitored for safety using DLT or DLT-equivalent boundaries as outlined in Table 29.

For each dose level allowed in Cohort B8 (100 mg BID and 150 mg BID), an interim analysis will be conducted after approximately [redacted] participants have been enrolled at that dose level and had at least 1 post-baseline scan. Enrollment may continue while the interim analysis is being conducted. An internal review will be conducted by the Phase 1b SRC as well as the study statistician, and Cohort B8 may be closed early if limited efficacy is observed based upon the interim analysis. A beta-binomial Bayesian model with a prior [redacted] will be employed to evaluate the posterior probability of activity. The stopping rules will be used to guide the decision making at interim if the posterior probability [redacted].

For Cohort B9, an interim analysis is planned for the purpose of dose confirmation after at least 10 participants have been enrolled at a particular dose and schedule [redacted] and have had the opportunity for [redacted] of treatment. Enrollment may continue while the interim analysis is being conducted. An internal review may be conducted by the Phase 1b SRC as well as the study statistician to [redacted] based on consideration of the [redacted]

Part F will be continually monitored using the same Bayesian toxicity monitoring rule used in Phase 1b. No efficacy interim analysis will be performed.

For Part G, an interim analysis is planned after at least [redacted] participants at each dose level, 50 mg BID and 100 mg BID [redacted] have been followed for a minimum of [redacted] of treatment and the median duration of follow-up is at least [redacted]. A Bayesian toxicity monitoring rule will be used to assess safety. Although all safety data will be assessed, there will be a focus on prespecified AEs, which are defined as the following AEs when they are deemed by the Investigator to be related to study treatment:

[redacted]

An internal review will be conducted by the Phase 1b SRC as well as the study statistician. In the case of unacceptable and/or unmanageable toxicity of the combination, this group may recommend halting 1 or both arms or terminating Part G. Otherwise, at any time enrollment into Arm G1 or Arm G2 will be temporarily halted if the following boundary is crossed: probability [redacted] Refer to the SAP for additional details.

The optimal dose of LY3537982 in combination with pembrolizumab may be determined at the interim analysis. The dose will be selected by the Phase 1b SRC as well as the study statistician based on consideration of the totality of data, including safety (e.g., dose intensity, dosage modifications [time course and prevalence of interruptions, reductions, and discontinuations], DLTs, fatal events, all grade and Grade 3/4 AEs, analyzed by cohort), efficacy (e.g., clinical response, analyzed by dose cohort), and PK (e.g., integrated dose and exposure analyses and PK and PD data analyzed by dose cohort). LY3537982 plasma concentration (PK data) versus time data from Part G participants will be used to enable population PK analysis. For Part G, LY3537982 plasma concentration (PK data) versus time data will be available at an appropriate time to enable population PK analysis to be carried to generate results concomitantly to the safety results. Dosing information (date and time of dose administration and amount of LY3537982 administered) related to PK will be available to interpret the concentration data.

If the optimal dose in Part G is not determined at the interim analysis, a dose optimization analysis will be performed to select the optimal dose after approximately [redacted] participants at each dose level (50 mg BID and 100 mg BID), including participants from the [redacted] have had the opportunity for [redacted] of treatment and a median duration of follow-up of at least [redacted]. Based on emerging data the Sponsor may stop the randomization and continue enrollment at the anticipated dose level before the optimal dose is declared.

Part H

For Part H, an interim analysis is planned after at least [redacted] participants in each arm have been followed for a minimum of [redacted] of treatment. A Bayesian toxicity monitoring rule will be used to assess safety.

An internal review will be conducted by the Phase 1b SRC as well as the study statistician. In the case of unacceptable and/or unmanageable toxicity of the combination, this group may recommend halting 1 or both arms or terminating Part H. Otherwise, at any time enrollment into Arm H1 or Arm H2 will be temporarily halted if the following boundary is crossed: probability [redacted] Refer to the SAP for additional details.

The optimal dose of LY3537982 in combination with cetuximab may be determined at the interim analysis. The dose will be selected by the Phase 1b SRC as well as the study statistician based on consideration of the totality of data, including safety, efficacy, and PK, if available. For Part H, LY3537982 plasma concentration (PK data) versus time data will be assessed at an appropriate time to enable a population PK analysis concurrent with safety results. Dosing information (date and time of dose administration and amount of LY3537982 administered) related to PK will be available to interpret the concentration data.

If the optimal dose in Part H is not determined at the interim analysis, a dose optimization analysis will be performed to select the optimal dose after at least [redacted] participants at each dose level (100 mg BID and 150 mg BID) and have had the opportunity for [redacted] of treatment. Based on emerging data the Sponsor may stop the randomization and continue enrollment at the anticipated dose level before the optimal dose is declared.

In all of Phase 1b and Phase 2, although the decision of whether to close a cohort will be made primarily based on the safety monitored continuously using Bayesian toxicity monitoring (Section 8.5), the totality of data (including safety, efficacy, and PK, if available) will also be reviewed and considered in the final decision made by the Sponsor. The decision will be communicated to sites in writing if the Sponsor determines a cohort is to be closed.

Sponsor has systematic and robust internal processes in place that ensure safety surveillance of development compounds in line with expectations of regulatory agencies. This includes processes with clearly described roles and responsibilities that are owned by Sponsor's Global Patient Safety organization. These processes are designed to monitor the evolving safety profile (i.e., review of cumulative SAEs and other important safety information) by designated cross functional teams in a timely manner at predefined intervals or on an ad-hoc basis. In addition, a dedicated process may be used to perform unblinded comparisons of event rates for SAEs, as necessary.

This system ensures that the accumulating safety data derived from individual and multiple trials across a development program are reviewed on a regular basis and that important new safety information, such as the need for protocol modification or other relevant safety-related material,

is identified and communicated to regulators and Investigators appropriately and in a timely fashion. An internal review of aggregate safety data occurs on at least a quarterly basis or more frequently, as appropriate. Any SARs are reported within the required timeline for expedited reporting.

In addition to annual periodic safety updates and to further inform Investigators, a line listing reports of SUSARs is created and distributed to Investigators on a biannual (twice yearly) basis. Any significant potential risk/safety concerns that are being monitored, as well as any results being reported in other periodic reports for the compound, SRC decisions, and other significant safety data (e.g., nonclinical, clinical findings, and removal of SARs) are included in the report.

9.0 SUPPORTING DOCUMENTATION AND OPERATIONAL CONSIDERATIONS

9.1 Appendix 1: Regulatory, Ethical, and Study Oversight Considerations

9.1.1 Regulatory and Ethical Considerations

- This study will be conducted in accordance with the protocol and with the following:
    - Consensus ethical principles derived from international guidelines including the Declaration of Helsinki and Council for International Organizations of Medical Sciences International Ethical Guidelines
    - Applicable laws and regulations
- The protocol, protocol amendments, ICF, IB, and other relevant documents (e.g., advertisements) must be submitted to an IRB/IEC by the Investigator and reviewed and approved by the IRB/IEC before the study is initiated.
- Any amendments to the protocol will require IRB/IEC approval before implementation of changes made to the study design, except for changes necessary to eliminate an immediate hazard to study participants.
- The Investigator will be responsible for the following:
    - Providing written summaries of the status of the study to the IRB/IEC annually or more frequently in accordance with the requirements, policies, and procedures established by the IRB/IEC
    - Notifying the IRB/IEC of SAEs or other significant safety findings as required by IRB/IEC procedures
    - Providing oversight of the conduct of the study at the site and adherence to requirements of 21 CFR, ICH guidelines, the IRB/IEC, European regulation 536/2014 for clinical studies (if applicable), and all other applicable local regulations, and
    - Reporting significant issues related to participant safety, participant rights, or data integrity.

After reading the protocol, each PI will sign the protocol signature page and send a copy of the signed page to a Sponsor representative.

The CSR Coordinating Investigator will sign the final CSR for this study, indicating agreement that, to the best of his or her knowledge, the report accurately describes the conduct and results of the study.

9.1.2 Informed Consent Process

- The Investigator or his/her representative will explain the nature of the study, including the risks and benefits, to the participant and answer all questions regarding the study.

- Participants must be informed that their participation is voluntary. Participants will be required to sign a statement of informed consent that meets the requirements of 21 CFR 50, local regulations, ICH guidelines, Health Insurance Portability and Accountability Act requirements, where applicable, and the IRB/IEC or study center.
- The medical record must include a statement that written informed consent was obtained before the participant was entered in the study and the date the written consent was obtained. The authorized person obtaining the informed consent must also sign the ICF.
- Participants must be reconsented to the most current version of the ICF(s) during their participation in the study.
- A copy of the ICF(s) must be provided to the participant and kept on file.
- Participants who are rescreened are required to sign a new ICF.

9.1.3 Data Protection

- Participants will be assigned a unique identifier by the Investigator. Any participant records or datasets that are transferred to the Sponsor will contain the identifier only; participant names or any information which would make the participant identifiable will not be transferred.
- The participant and/or legal representative, when applicable, must be informed that his/her personal study-related data will be used by the Sponsor in accordance with local data protection law. The level of disclosure must also be explained to the participant, who will be required to give consent for their data to be used as described in the informed consent. This is done by the site personnel through the informed consent process.
- The participant and/or legal representative, when applicable, must be informed through the ICF by the site personnel that his/her medical records may be examined by Clinical Quality Assurance auditors or other authorized personnel appointed by the Sponsor, by appropriate IRB/IEC members, and by inspectors from regulatory authorities.
- The Sponsor has processes in place to ensure information security, data integrity, and data protection. These processes address management of data transfer, prevention and management of unauthorized access, disclosure, dissemination, and alteration or loss of information or personal data. These processes include contingency plan(s) for appropriate and timely response in the event of a data security breach.
- The transfer of personal data is subject to appropriate safeguards through contractual agreements and processes. The Sponsor’s processes are compliant with local privacy laws and relevant legislations including the GDPR.

9.1.4 Dissemination of Clinical Study Data

Dissemination of study data will be performed according to all applicable Sponsor and international policies.

Reports

The Sponsor will disclose a summary of trial information, including tabular trial results, on publicly available websites where required by local law or regulation.

The summary of results will be posted within the time frame specified by local law or regulation. If the trial remains ongoing in some countries and a statistical analysis of an incomplete dataset would result in analyses lacking scientific rigor (for example, underpowered) or compromise the integrity of the overall analyses (for example, trial not yet unblinded), the summary of results will be submitted within 1 year after the end of the trial globally or as soon as available, whichever is earlier.

Communication of Suspended or Terminated Dosing

If a decision is made to suspend or terminate dosing in the trial due to safety findings, the Sponsor will communicate this decision to all Investigators (for example, by phone and/or email) as soon as possible. It will be a requirement that Investigators respond upon receipt to confirm that they understand the communication and have taken the appropriate action prior to further dosing any participants with study intervention. Any Investigator not responding will be followed up by Sponsor personnel prior to any further planned dosing. If a dose is planned imminently, Sponsor personnel will immediately, and continually, use all efforts to reach Investigators until contact is made and instructions verified.

9.1.5 Data Quality Assurance

To ensure accurate, complete, and reliable data, the Sponsor or its representatives will do the following:

- Provide instructional material to the study sites, as appropriate
- Provide Sponsor start-up training to instruct the Investigators and study coordinators. This training will give instruction on the protocol, the completion of the CRFs, and study procedures.
- Make periodic visits to the study site
- Be available for consultation and stay in contact with the study site personnel by mail, telephone, and/or fax
- Review and verify data reported to detect potential errors

In addition, the Sponsor or its representatives will periodically check a sample of the participant data recorded against source documents at the study site. The study may be audited by the Sponsor or its representatives and/or regulatory agencies at any time. Investigators will be given notice before an audit occurs.

The Investigator will keep records of all original source data. This might include laboratory tests, medical records, and clinical notes. If requested, the Investigator will provide the Sponsor, applicable regulatory agencies, and applicable ERBs with direct access to original source documents.

9.1.6 Data Capture System

The Investigator is responsible for ensuring the accuracy, completeness, legibility, and timeliness of the data reported to the Sponsor.

An electronic data capture system will be used in this study for the collection of CRF data. The Investigator maintains a separate source for the data entered by the Investigator or designee into the Sponsor-provided electronic data capture system. The Investigator is responsible for the identification of any data to be considered source and for the confirmation that data reported are accurate and complete by signing the CRF.

Data collected via the Sponsor-provided data capture system(s) will be stored by a third party. The Investigator will have continuous access to the data during the study and until decommissioning of the data capture system(s). Prior to decommissioning, the Investigator will receive an archival copy of pertinent data for retention.

Data managed by a central vendor, such as laboratory test data, will be stored electronically in the central vendor's database system, and results will be provided to the Investigator for review and retention. Data will subsequently be transferred from the central vendor to the Sponsor data warehouse.

Data from complaint forms submitted to Sponsor will be encoded and stored in the global product complaint management system.

9.1.7 Source Documents

- Source documents provide evidence for the existence of the participant and substantiate the integrity of the data collected. Source documents are filed at the Investigator's site.
- Data reported on the CRF or entered in the eCRF that are transcribed from source documents must be consistent with the source documents or the discrepancies must be explained. The Investigator may need to request previous medical records or transfer records, depending on the study. Also, current medical records must be available.

9.1.8 Study and Site Closure

9.1.8.1 Discontinuation of the Study

The study will be discontinued if Sponsor or its designee judges it necessary for medical, safety, regulatory, or other reasons consistent with applicable laws, regulations, and GCP.

9.1.8.2 Discontinuation of Study Sites

Study site participation may be discontinued if Sponsor or its designee, the Investigator, or the ERB of the study site judges it necessary for medical, safety, regulatory, or other reasons consistent with applicable laws, regulations, and GCP.

9.2 Appendix 2: Clinical Laboratory Tests

- The tests detailed below will be performed as indicated in the table below.
- If a local sample is required, it is important that the sample for central analysis is obtained at the same time (if applicable). If there is an abnormal laboratory value or abnormal value for any other diagnostic or screening test (e.g., blood pressure increased, neutrophils decreased, etc.) and it is known to be related to a diagnosis (e.g., hypertension, neutropenia, etc.), this should be entered into the CRF. Do not enter the test abnormality, enter the diagnosis or categorical term.
- Protocol-specific requirements for inclusion or exclusion of participants are detailed in Section 4.0 of the protocol.
- Additional tests may be performed at any time during the study as determined necessary by the Investigator or required by local regulations, and any clinically significant abnormalities recorded in the AE eCRF.
- Investigators must document their review of each laboratory safety report. Enrollment and treatment decisions may be based upon local laboratory results. Discrepancies between local and central laboratory results will not be considered protocol deviations.

**Clinical Laboratory Tests**	
**Hematology**[a,b]	**Clinical Chemistry**[a,b]
Leukocytes (WBC)	**Serum Concentrations of:**
Neutrophils	ALT
Lymphocytes	Albumin
Monocytes	Alkaline phosphatase
Eosinophils	AST
Basophils	Bilirubin, direct
Erythrocytes (RBC)	Bilirubin, total
HGB	BUN or blood urea
HCT	Calcium
PLT	Creatinine
	Creatine phosphokinase
**Urinalysis**[b]	Glucose (random)
Blood	Magnesium
Glucose	Phosphorous
Ketones	Potassium
pH	Protein

Clinical Laboratory Tests	
**Hematology**[a,b]	**Clinical Chemistry**[a,b]
Protein	Sodium
Specific gravity	
Urine leukocyte esterase[d]	**Pregnancy Test**[b,c]
	Urine or serum pregnancy test
**Thyroid Function**[b,e]	
TSH	**Coagulation**[b]
FT4	PT/INR
T3 or FT3[f]	aPTT

Neutrophils reported by automated differential hematology instruments include both segmented and band forms. When a manual differential is needed to report the neutrophils, the segmented and band forms should be added together and recorded on the CRF, unless the CRF specifically provides an entry field for bands.

a Treatment and enrollment decisions will be based on local laboratory results.

b Local or Investigator-designated laboratory.

c For female participants of childbearing potential.

d Urine microscopy may be used in place of the urine leukocyte esterase assessment to test for the presence of WBC.

e FT4 should only be collected if TSH is not within normal limits.

f For Cohort B4, Cohort B9, and Part G only, thyroid testing should include TSH, FT4, and T3 or FT3.

9.3 Appendix 3: Contraceptive Guidance and Collection of Pregnancy Information

9.3.1 Definitions

Women of Childbearing Potential: A woman is considered fertile following menarche and until becoming postmenopausal unless permanently sterile (see below).

If fertility is unclear (e.g., amenorrhea in adolescents or athletes) and a menstrual cycle cannot be confirmed before first dose of study intervention, additional evaluation should be considered.

Women in the following categories are **not** considered WOCBP:

a. Premenarchal female

b. Premenopausal female with 1 of the following:

- Documented hysterectomy
- Documented bilateral salpingectomy
- Documented bilateral oophorectomy

For individuals with permanent infertility due to an alternate medical cause other than the above (e.g., mullerian agenesis and androgen insensitivity), Investigator discretion should be applied to determining study entry.

Note: Documentation can come from the site personnel's review of the participant's medical records, medical examination, or medical history interview.

c. Postmenopausal female

A postmenopausal state is defined as no menses for 12 months without an alternative medical cause.

A high follicle stimulating hormone level in the postmenopausal range may be used to confirm a postmenopausal state in women not using hormonal contraception or HRT. However, in the absence of 12 months of amenorrhea, confirmation with more than 1 follicle stimulating hormone measurement > 40 mIU/mL is required.

Females on HRT and whose menopausal status is in doubt will be required to use one of the non-estrogen hormonal highly effective contraception methods if they wish to continue their HRT during the study. Otherwise, they must discontinue HRT to allow confirmation of postmenopausal status before study enrollment.

9.3.2 Contraception Guidance

CONTRACEPTIVES[a] ALLOWED DURING THE STUDY INCLUDE:
**Highly Effective Methods[b] That Have Low User Dependency**
• Implantable progestogen-only hormone contraception associated with inhibition of ovulation[c]
• Intrauterine device
• Intrauterine hormone-releasing system[c]
• Bilateral tubal occlusion
• Vasectomized partner<br>*(Vasectomized partner is a highly effective contraceptive method provided that the partner is the sole sexual partner of the woman of childbearing potential and the absence of sperm has been confirmed. If not, an additional highly effective method of contraception should be used. Spermatogenesis cycle is approximately 90 days.)*
**Highly Effective Methods[b] That Are User Dependent**
• Combined (estrogen- and progestogen-containing) hormonal contraception associated with inhibition of ovulation[c]<br>o oral<br>o intravaginal<br>o transdermal<br>o injectable
• Progestogen-only hormone contraception associated with inhibition of ovulation[c]<br>o oral<br>o injectable
• Sexual abstinence<br>*(Sexual abstinence is considered a highly effective method only if defined as refraining from heterosexual intercourse during the entire period of risk associated with the study intervention. The reliability of sexual abstinence needs to be evaluated in relation to the duration of the study and the preferred and usual lifestyle of the participant.)*

Periodic abstinence (calendar, symptothermal, or postovulation methods), withdrawal (coitus interruptus), spermicides only, and lactational amenorrhea method are not acceptable methods of contraception for this study. Male condom and female condom should not be used together (due to risk of failure with friction).

[a] Contraceptive use by men or women should be consistent with local regulations regarding the use of contraceptive methods for those participating in clinical studies.

[b] Failure rate of < 1% per year when used consistently and correctly. Typical use failure rates differ from those when used consistently and correctly.

[c] Male condoms must be used in addition to hormonal contraception. If locally required, in accordance with Clinical Trial Facilitation Group guidelines, acceptable contraceptive methods are limited to those which inhibit ovulation as the primary mode of action.

9.3.3 Pregnancy Reporting

If the participant or partner of a participant participating in the study becomes pregnant during the study or within 28 days of discontinuing study drug, the Investigator should report the pregnancy on the appropriate pregnancy reporting form and submit it to the Sponsor.

Note: For Cohort B4, Cohort B9, and Part G only, all pregnancies and exposure during breastfeeding, from the time of treatment/allocation through 120 days (Cohort B4 and Part G) or 180 days (Cohort B9) following cessation of study treatment, or 28 days following cessation of study treatment if the participant initiates new anticancer therapy, must be reported by the Investigator.

A participant who becomes pregnant while on study drug will immediately be withdrawn from the study, and early termination study procedures will be performed.

The participant or partner should be followed by the Investigator until completion of the pregnancy. If the pregnancy ends for any reason before the anticipated date, the Investigator should notify the Sponsor following the SAE process. At the completion of the pregnancy, the Investigator will document the outcome of the pregnancy. If the outcome of the pregnancy meets the criteria for immediate classification as an SAE (i.e., postpartum complication, spontaneous abortion, stillbirth, neonatal death, or congenital anomaly), the Investigator should follow the procedures for reporting an SAE.

9.4 Appendix 4: Liver Safety: Suggested Actions and Follow-Up Assessments

Selected tests may be obtained in the event of a treatment-emergent hepatic abnormality and may be required in follow-up with participants in consultation with the Sponsor.

**Hepatic Monitoring Tests**	
**Hepatic Hematology**[a]	**Haptoglobin**[a]
HGB	
HCT	**Hepatic Coagulation**[a]
Erythrocytes (RBC)	Prothrombin time
Leukocytes (WBC)	Prothrombin time, INR
Neutrophils[b]	
Lymphocytes	**Hepatic Serologies**[a,c]
Monocytes	Hepatitis A antibody, total
Eosinophils	Hepatitis A antibody, IgM
Basophils	Hepatitis B surface antigen
PLT	Hepatitis B surface antibody
	Hepatitis B Core antibody
**Hepatic Chemistry**[a]	Hepatitis C antibody
TBL	Hepatitis E antibody, IgG
Direct bilirubin	Hepatitis E antibody, IgM
Alkaline phosphatase	
ALT	**Recommended Autoimmune Serology**
AST	Antinuclear antibody[a]
GGT	Antismooth muscle antibody[a]
Creatine phosphokinase	Antiactin antibody[a]

a Assayed by local laboratory.

b Neutrophils reported by automated differential hematology instruments include both segmented and band forms. Whenever a manual differential is needed to report the neutrophils, the segmented and band forms should be added together and recorded on the CRF, unless the CRF specifically provides an entry field for bands.

c Reflex/confirmation dependent on regulatory requirements and/or testing availability.

CCI

[redacted]

CCI

[redacted]

9.6 Appendix 6: Cockcroft-Gault Formula

Note: This formula is to be used for calculating creatinine clearance from **local laboratory results only.**

Cockcroft-Gault prediction of creatinine clearance from serum creatinine:

For serum creatinine concentration in mg/dL:

$$\text{CRCL} = \frac{(\mathbf{140 - age^{a}}) \times (\mathbf{wt}) \times \mathbf{0.85} \text{ (if female), or} \times \mathbf{1.0} \text{ (if male)}}{\mathbf{72} \times \textbf{serum creatinine} \text{ (mg/dL)}}$$
(mL/min)

For serum creatinine concentration in µmol/L:

$$\text{CRCL} = \frac{(\mathbf{140 - age^{a}}) \times (\mathbf{wt}) \times \mathbf{0.85} \text{ (if female), or} \times \mathbf{1.0} \text{ (if male)}}{\mathbf{0.81} \times \textbf{serum creatinine} \text{ (µmol/L)}}$$
(mL/min)

[a] Age in years, wt in kilograms.

Source: Cockcroft and Gault 1976

9.7 Appendix 7: Operating Characteristics of Continuous Safety Monitoring

A Bayesian beta-binomial model is introduced in Section 8.5 to monitor participant safety. It is assumed that DLTs and DLT-equivalents follow a beta-binomial model. [redacted] prior is assumed for the DLT rate. The continuous safety monitoring begins when N = 7 participants have been enrolled into the relevant portion of the study. Enrollment into a particular cohort will be temporarily halted if the following boundary is crossed: posterior probability [redacted]

Operating characteristics for this procedure are given in Table 30, Table 31 and Table 32 for [redacted] respectively. Operating characteristics are calculated using the U-Design software with 10,000 Monte-Carlo samples. Four scenarios for the DLT rate are presented: a true rate of [redacted].

CCI

[redacted]

9.8 Appendix 8: Dose Escalation Operating Characteristics

9.8.1 List of Designs

Simulation results comparing the operating characteristics of the 3+3 and mTPI-2 designs are provided. The 3+3 design was chosen as a comparison because it is a widely used escalation design. The pT for the mTPI is [redacted]. Details about the 2 designs are given in the table below. Simulations are generated by the U-Design software (v1.4 at https://udesign.laiyaconsulting.com).

**Index**	**Design Name**	**Arguments**
Design 1	3+3	Start dose = 1
Design 2	mTPI-2	Start dose = 1,<br>Sample size = 100,<br>Cohort size = 3,<br>$\varepsilon_1 = 0.05$,<br>$\varepsilon_2 = 0.05$,<br>Maximum sample size at a dose = 6

The cohort size is the number of participants enrolled per cohort; ε_1 and ε_2 are 2 small fractions to account for the uncertainty around the true target toxicity, so the equivalence interval is $[p_T-\varepsilon_1, p_T+\varepsilon_2]$ for mTPI-2.

9.8.2 Simulation Scenarios

Six scenarios are listed below, covering a variety of possible dose-toxicity scenarios. The true toxicity probability (pT) at each DL for each scenario is listed below. Each scenario was explored with 10 000 simulations. The DL of the MTD is shown in **bold text** in the table below. In Scenario 6 all doses are below the pT.

[redacted]

9.8.3 Simulation Results

Designs under comparison are represented by different colors. Four metrics are reported for each scenario and compared across designs.

1. **Probability of Selecting MTD**: The proportion of the simulated trials that correctly select the true MTD across all the simulated trials. The higher the value, the better the design is at selecting a dose with the specified pT.
2. **Probability of Toxicity**: The proportion of participants who have experienced DLT across all the simulated trials. The lower the number, the fewer participants having DLTs under the design.
3. **Probability of Selecting Dose-over-MTD**: The proportion of the simulated trials that select a dose higher than the true MTD at the end of the trial.
4. **Probability of Overdosing Allocation**: The average proportion of participants who are assigned to doses higher than the MTD.

The line plot below presents metrics 1 to 4 for both designs across all the scenarios. Note that some designs may have identical values and therefore their colored lines may completely overlay with each other. And the table below summarizes the means and standard deviations of the above 4 statistics for current designs across scenarios.

Summary of Performance

[redacted]

In Scenarios 1 through 4 and 6, the mTPI-2 selects the true MTD more often than 3+3 (top left plot) without observing more DLTs than 3+3 (top right plot) or assigning more participants to doses above the MTD (bottom right panel). For some scenarios there is an increase in the proportion of times a dose above the MTD is selected (bottom left plot).

The difference in performance between mTPI-2 and 3+3 selecting the MTD in Scenario 5 (when all doses above MTD) is due primarily to the decision rule when 2/6 participants at the first dose have experienced DLTs. The 3+3 algorithm would declare no dose is the MTD while mTPI-2 would declare the first dose is the MTD.

CCI

[redacted]

9.9 Appendix 9: Dose Modification for Pembrolizumab

9.9.1 Definition of Dose-Limiting Toxicity

All toxicities will be graded using NCI-CTCAE Version 5.0 based on the Investigator assessment.

The DLT window of observation will be during Cycle 1.

The occurrence of any of the following toxicities during Cycle 1 will be considered a DLT, if assessed by the Investigator to be possibly, probably, or definitely related to study treatment administration.

1. Grade 4 nonhematologic toxicity (not laboratory).
2. Grade 4 hematologic toxicity lasting ≥ 7 days, except thrombocytopenia:
    - Grade 4 thrombocytopenia of any duration
    - Grade 3 thrombocytopenia associated with clinically significant bleeding
3. Any nonhematologic AE ≥ Grade 3 in severity should be considered a DLT, with the following exceptions: Grade 3 fatigue lasting ≤ 3 days; Grade 3 diarrhea, nausea, or vomiting without use of antiemetics or antidiarrheals per standard of care; Grade 3 rash without use of corticosteroids or anti-inflammatory agents per standard of care.
4. Any Grade 3 or Grade 4 non-hematologic laboratory value if:
    - Clinically significant medical intervention is required to treat the participant or
    - The abnormality leads to hospitalization, or
    - The abnormality persists for > 1 week.
    - The abnormality results in a DILI
    - Exceptions: Clinically nonsignificant, treatable, or reversible laboratory abnormalities including LFTs, uric acid, etc.
5. Febrile neutropenia Grade 3 or Grade 4:
    - Grade 3 is defined as ANC < 1000/mm^3 with a single temperature of > 38.3 degrees C (101 degrees F) or a sustained temperature of ≥ 38°C (100.4°F) for more than 1 hour
    - Grade 4 is defined as ANC < 1000/mm^3 with a single temperature of > 38.3°C (101°F) or a sustained temperature of ≥ 38°C (100.4°F) for more than 1 hour, with life-threatening consequences and urgent intervention indicated.
6. Prolonged delay (> 2 weeks) in initiating Cycle 2 due to treatment-related toxicity.
7. Any treatment-related toxicity that causes the participant to discontinue treatment during Cycle 1.

8. Missing > 25% of LY3537982 doses as a result of drug-related AE(s) during the first cycle.
9. Grade 5 toxicity.

9.9.2 Dose Modification and Toxicity Management for Immune-Related AEs Associated with Pembrolizumab

AEs associated with pembrolizumab exposure may represent an immunologic etiology. These irAEs may occur shortly after the first dose or several months after the last dose of pembrolizumab treatment and may affect more than one body system simultaneously. Therefore, early recognition and initiation of treatment is critical to reduce complications. Based on existing clinical study data, most irAEs were reversible and could be managed with interruptions of pembrolizumab, administration of corticosteroids and/or other supportive care. For suspected irAEs, ensure adequate evaluation to confirm etiology or exclude other causes. Additional procedures or tests such as bronchoscopy, endoscopy, skin biopsy may be included as part of the evaluation. Based on the severity of irAEs, withhold or permanently discontinue pembrolizumab and administer corticosteroids. Dose modification and toxicity management guidelines for irAEs associated with pembrolizumab are provided in Table 33.

Attribution of Toxicity

When study interventions are administered in combination, attribution of an adverse event to a single component is likely to be difficult. Therefore, while the Investigator may attribute a toxicity event to the combination, to LY3537982 alone, or to pembrolizumab alone, for adverse events listed in Table 33, both interventions must be held according to the criteria in Table 33.

Holding Study Interventions

When study interventions are administered in combination, if the AE is considered immune-related, both interventions should be held according to recommended dose modifications.

Restarting Study Interventions

Participants may not have any dose modifications (no change in dose or schedule) of pembrolizumab in this study, as described in Table 33.

If the toxicity does not resolve or the criteria for resuming treatment are not met, the participant must be discontinued from all study interventions.

If the toxicities do resolve and conditions are aligned with what is defined in Table 33, the combination of LY3537982 and pembrolizumab may be restarted at the discretion of the Investigator. In these cases where the toxicity is attributed to the combination or to LY3537982 alone, re-initiation of pembrolizumab as a monotherapy may be considered after communication with and agreement by the Sponsor.

Table 33: Dose Modification and Toxicity Management Guidelines for Immune-related AEs Associated with Pembrolizumab

General instructions:

1. Severe and life-threatening irAEs should be treated with IV corticosteroids followed by oral steroids. Other immunosuppressive treatment should begin if the irAEs are not controlled by corticosteroids.
2. Pembrolizumab must be permanently discontinued if the irAE does not resolve or the corticosteroid dose is not ≤ 10 mg/day within 12 weeks of the last pembrolizumab-treatment.
3. The corticosteroid taper should begin when the irAE is ≤ Grade 1 and continue for at least 4 weeks.
4. If pembrolizumab has been withheld, pembrolizumab may resume after the irAE decreased to ≤ Grade 1 after corticosteroid taper.

irAEs	Toxicity grade (CTCAE V5.0)	Action with pembrolizumab	Corticosteroid and/or other therapies	Monitoring and follow-up
Pneumonitis	Grade 2	Withhold	Administer corticosteroids (initial dose of 1 to 2 mg/kg prednisone or equivalent) followed by taper<br>Add prophylactic antibiotics for opportunistic infections	Monitor participants for signs and symptoms of pneumonitis<br>Evaluate participants with suspected pneumonitis with radiographic imaging and initiate corticosteroid treatment
	Recurrent Grade 2,<br>Grade 3 or 4	Permanently discontinue		
Diarrhea / Colitis	Grade 2 or 3	Withhold	Administer corticosteroids (initial dose of 1 to 2 mg/kg prednisone or equivalent) followed by taper	Monitor participants for signs and symptoms of enterocolitis (i.e., diarrhea, abdominal pain, blood, or mucus in stool with or without fever) and of bowel perforation (i.e., peritoneal signs and ileus)<br>Participants with ≥ Grade 2 diarrhea suspecting colitis should consider GI consultation and performing endoscopy to rule out colitis<br>Participants with diarrhea/colitis should be advised to drink liberal quantities of clear fluids. If sufficient oral fluid intake is not feasible, fluid and electrolytes should be substituted via IV infusion
	Recurrent Grade 3 or Grade 4	Permanently discontinue		

General instructions:

1. Severe and life-threatening irAEs should be treated with IV corticosteroids followed by oral steroids. Other immunosuppressive treatment should begin if the irAEs are not controlled by corticosteroids.
2. Pembrolizumab must be permanently discontinued if the irAE does not resolve or the corticosteroid dose is not ≤ 10 mg/day within 12 weeks of the last pembrolizumab-treatment.
3. The corticosteroid taper should begin when the irAE is ≤ Grade 1 and continue for at least 4 weeks.
4. If pembrolizumab has been withheld, pembrolizumab may resume after the irAE decreased to ≤ Grade 1 after corticosteroid taper.

irAEs	Toxicity grade (CTCAE V5.0)	Action with pembrolizumab	Corticosteroid and/or other therapies	Monitoring and follow-up
AST or ALT elevation or increased bilirubin	Grade 2[a]	Withhold	Administer corticosteroids (initial dose of 0.5 to 1 mg/kg prednisone or equivalent) followed by taper	Monitor with LFTs (consider weekly or more frequently until liver enzyme value returned to baseline or is stable)
	Grade 3[b] or 4[c]	Permanently discontinue	Administer corticosteroids (initial dose of 1 to 2 mg/kg prednisone or equivalent) followed by taper	
T1DM or Hyperglycemia	New onset T1DM or Grade 3 or 4 hyperglycemia associated with evidence of β-cell failure	Withhold [d]	Initiate insulin replacement therapy for participants with T1DM<br>Administer antihyperglycemic in participants with hyperglycemia	Monitor participants for hyperglycemia or other signs and symptoms of diabetes
Hypophysitis	Grade 2	Withhold	Administer corticosteroids and initiate hormonal replacements as clinically indicated	Monitor for signs and symptoms of hypophysitis (including hypopituitarism and adrenal insufficiency)
	Grade 3 or 4	Withhold or permanently discontinue [d]		
Hyperthyroidism	Grade 2	Continue	Treat with nonselective beta-blockers (e.g., propranolol) or thionamides as appropriate	Monitor for signs and symptoms of thyroid disorders
	Grade 3 or 4	Withhold or permanently discontinue [d]		

General instructions:<br>1. Severe and life-threatening irAEs should be treated with IV corticosteroids followed by oral steroids. Other immunosuppressive treatment should begin if the irAEs are not controlled by corticosteroids.<br>2. Pembrolizumab must be permanently discontinued if the irAE does not resolve or the corticosteroid dose is not ≤ 10 mg/day within 12 weeks of the last pembrolizumab-treatment.<br>3. The corticosteroid taper should begin when the irAE is ≤ Grade 1 and continue for at least 4 weeks.<br>4. If pembrolizumab has been withheld, pembrolizumab may resume after the irAE decreased to ≤ Grade 1 after corticosteroid taper.				
**irAEs**	**Toxicity grade (CTCAE V5.0)**	**Action with pembrolizumab**	**Corticosteroid and/or other therapies**	**Monitoring and follow-up**
Hypothyroidism	Grade 2, 3 or 4	Continue	Initiate thyroid replacement hormones (eg, levothyroxine or liothyronine) per standard of care	Monitor for signs and symptoms of thyroid disorders
Nephritis:<br>grading according to increased creatinine or acute kidney injury	Grade 2	Withhold	Administer corticosteroids (prednisone 1 to 2 mg/kg or equivalent) followed by taper	Monitor changes of renal function
	Grade 3 or 4	Permanently discontinue		
Neurological Toxicities	Grade 2	Withhold	Based on severity of AE administer corticosteroids	Ensure adequate evaluation to confirm etiology and/or exclude other causes
	Grade 3 or 4	Permanently discontinue		
Myocarditis	Asymptomatic cardiac enzyme elevation with clinical suspicion of myocarditis (previously CTCAE v4.0 Grade 1)	Withhold	Based on severity of AE administer corticosteroids	Ensure adequate evaluation to confirm etiology and/or exclude other causes
	Grade 2, 3 or 4	Permanently discontinue		
Exfoliative Dermatologic Conditions	Suspected SJS, TEN, or DRESS	Withhold	Based on severity of AE administer corticosteroids	Ensure adequate evaluation to confirm etiology or exclude other causes
	Confirmed SJS, TEN, or DRESS	Permanently discontinue		

General instructions:

1. Severe and life-threatening irAEs should be treated with IV corticosteroids followed by oral steroids. Other immunosuppressive treatment should begin if the irAEs are not controlled by corticosteroids.
2. Pembrolizumab must be permanently discontinued if the irAE does not resolve or the corticosteroid dose is not ≤ 10 mg/day within 12 weeks of the last pembrolizumab-treatment.
3. The corticosteroid taper should begin when the irAE is ≤ Grade 1 and continue for at least 4 weeks.
4. If pembrolizumab has been withheld, pembrolizumab may resume after the irAE decreased to ≤ Grade 1 after corticosteroid taper.

**irAEs**	**Toxicity grade (CTCAE V5.0)**	**Action with pembrolizumab**	**Corticosteroid and/or other therapies**	**Monitoring and follow-up**
All Other irAEs	Persistent Grade 2	Withhold	Based on severity of AE administer corticosteroids	Ensure adequate evaluation to confirm etiology or exclude other causes
	Grade 3	Withhold or discontinue based on the event [e]		
	Recurrent Grade 3 or Grade 4	Permanently discontinue		

Non-irAE will be managed as appropriate following clinical practice recommendations.

[a] AST/ALT: > 3.0 to 5.0 × ULN if baseline normal; > 3.0 to 5.0 × baseline, if baseline abnormal; bilirubin: > 1.5 to 3.0 × ULN if baseline normal; > 1.5 to 3.0 × baseline if baseline abnormal

[b] AST/ALT: > 5.0 to 20.0 × ULN, if baseline normal; > 5.0 to 20.0 × baseline, if baseline abnormal; bilirubin: > 3.0 to 10.0 × ULN if baseline normal; > 3.0 to 10.0 × baseline if baseline abnormal

[c] AST/ALT: > 20.0 × ULN, if baseline normal; > 20.0 × baseline, if baseline abnormal; bilirubin: > 10.0 × ULN if baseline normal; > 10.0 × baseline if baseline abnormal

[d] The decision to withhold or permanently discontinue pembrolizumab is at the discretion of the Investigator or treating physician. If control achieved or ≤ Grade 2, pembrolizumab may be resumed.

[e] Events that require discontinuation include, but are not limited to, encephalitis and other clinically important irAEs (e.g., vasculitis and sclerosing cholangitis).

9.9.3 Dose Modification and Toxicity Management of Infusion Reactions Related to Pembrolizumab

Pembrolizumab may cause severe or life-threatening infusion reactions including severe hypersensitivity or anaphylaxis. Signs and symptoms usually develop during or shortly after drug infusion and generally resolve completely within 24 hours of completion of infusion. Dose modification and toxicity management guidelines on pembrolizumab-associated infusion reaction are provided in Table 34.

Table 34: Pembrolizumab Infusion Reaction Dose Modification and Treatment Guidelines

NCI CTCAE Grade	Treatment	Premedication at Subsequent Dosing
**Grade 1**<br><br>Mild reaction; infusion interruption not indicated; intervention not indicated	• Increase monitoring of vital signs as medically indicated until the participant is deemed medically stable in the opinion of the Investigator.	None
**Grade 2**<br><br>Requires therapy or infusion interruption but responds promptly to symptomatic treatment (e.g., antihistamines, NSAIDs, narcotics, IV fluids); prophylactic medications indicated for ≤ 24 hrs.	• **Stop Infusion.**<br>• Additional appropriate medical therapy may include, but is not limited to:<br>• IV fluids<br>• Antihistamines<br>• NSAIDs<br>• Acetaminophen<br>• Narcotics<br>• Increase monitoring of vital signs as medically indicated until the participant is deemed medically stable in the opinion of the Investigator.<br>• If symptoms resolve within 1 hour of stopping drug infusion, the infusion may be restarted at 50% of the original infusion rate (e.g., from 100 mL/hr to 50 mL/hr). Otherwise, dosing will be held until symptoms resolve and the participant should be premedicated for the next scheduled dose.<br>**Participants who develop Grade 2 toxicity despite adequate premedication should be permanently discontinued from further study drug treatment.**	Participant may be premedicated 1.5 hr (± 30 minutes) prior to infusion of study intervention with: diphenhydramine 50 mg PO (or equivalent dose of antihistamine); acetaminophen 500 to 1000 mg PO (or equivalent dose of analgesic)

NCI CTCAE Grade	Treatment	Premedication at Subsequent Dosing
**Grades 3 or 4**<br><br>**Grade 3:**<br>Prolonged (i.e., not rapidly responsive to symptomatic medication and/or brief interruption of infusion); recurrence of symptoms following initial improvement; hospitalization indicated for other clinical sequelae (e.g., renal impairment, pulmonary infiltrates)<br><br>**Grade 4:**<br>Life-threatening; pressor or ventilator support indicated	• **Stop Infusion.**<br>• Additional appropriate medical therapy may include, but is not limited to:<br>• Epinephrine**<br>• IV fluids<br>• Antihistamines<br>• NSAIDs<br>• Acetaminophen<br>• Narcotics<br>• Oxygen<br>• Pressors<br>• Corticosteroids<br>• Increase monitoring of vital signs as medically indicated until the participant is deemed medically stable in the opinion of the Investigator.<br>• Hospitalization may be indicated.<br><br>**In cases of anaphylaxis, epinephrine should be used immediately.<br>Participant is permanently discontinued from further study drug treatment.	No subsequent dosing

Appropriate resuscitation equipment should be available at the bedside and a physician readily available during the period of drug administration. For further information, please refer to the Common Terminology Criteria for Adverse Events v5.0 (CTCAE) at http://ctep.cancer.gov.

9.9.4 Other Allowed Dose Interruption for Pembrolizumab

Pembrolizumab may be interrupted for situations other than treatment-related AEs such as medical or surgical events and/or unforeseen circumstances not related to study intervention. However, study intervention is to be restarted within 21 days of the originally scheduled dose and within 42 days of the previously administered dose, unless otherwise discussed with the Sponsor. The reason for interruption is to be documented in the participant's study record.

9.9.5 Pembrolizumab Events of Clinical Interest

Selected nonserious and serious adverse events are also known as Events of Clinical Interest (ECI) and must be reported to the Sponsor within 24 hours of awareness.

Events of clinical interest for this trial include:

1) An overdose of pembrolizumab, as defined in Section 7.6, that is not associated with clinical symptoms or abnormal laboratory results.
2) An elevated AST or ALT lab value that is greater than or equal to 3× the upper limit of normal and an elevated total bilirubin lab value that is greater than or equal to 2× the upper limit of normal and, at the same time, an alkaline phosphatase lab value that is less than 2× the upper limit of normal, as determined by way of protocol-specified laboratory testing or unscheduled laboratory testing.*

***Note:** These criteria are based upon available regulatory guidance documents. The purpose of the criteria is to specify a threshold of abnormal hepatic tests that may require an additional evaluation for an underlying etiology. The trial site guidance for assessment and follow-up of these criteria can be made available. It may also be appropriate to conduct additional evaluation for an underlying etiology in the setting of abnormalities of liver blood tests including AST, ALT, bilirubin, and alkaline phosphatase that do not meet the criteria noted above. In these cases, the decision to proceed with additional evaluation will be made through consultation between the study Investigators and the Sponsor. However, abnormalities of liver blood tests that do not meet the criteria noted above are not ECIs for this trial.

9.10 Appendix 10: Country-Specific Addenda to Clinical Protocol LOXO-RAS-20001

Changes for each country in the subsections below are shown as strikethrough (i.e., ~~participant~~) for deleted text and as underscore (i.e., participant) for new/amended text compared to the text in the main body of this protocol. All changes made within the protocol body (as outlined below) will be applicable to the protocol synopsis as well.

9.10.1 France

These changes are specific to study sites in France and are based on Agence Nationale de Sécurité du Medicament et des Produits de Santé (ANSM) and Ethics Committee queries.

Changes made within the protocol that are applicable to patients in France are shown in Sections 9.10.1.1 through 9.10.1.7. Specific changes made to protocol assessments are shown in the Schedule of Assessments (SoA) in Section 9.10.1.5.

9.10.1.1 Section 3.1 Study Design

Footnote (a) is to be added to Figure 3, as shown below:

Figure 3: Phase 1b Dose Optimization (Part G) Study Schema[a]

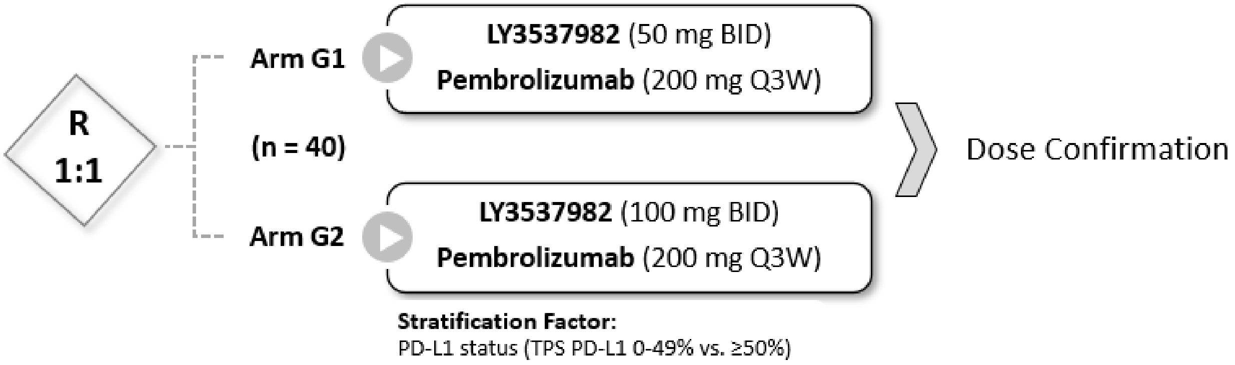

a Participants enrolling in France in Part G must have a PD-L1 expression ≥ 50%.

9.10.1.2 Section 4.1 Inclusion Criteria

For Phase 1b Dose Expansion Part B (NSCLC), Inclusion Criterion 4(b) was modified by adding the following text after the first bullet point:

CCI [redacted]

For Phase 1b Dose Expansion Part C (CRC), Inclusion Criterion 4(c) was modified as follows:

- Individuals must be appropriate candidates for study treatment and must have received at least 2 prior lines of therapy and have failed at least 1 prior oxaliplatin- or irinotecan-containing regimen for advanced or metastatic CRC. Study treatment is considered appropriate where approved measures are no longer effective or are not considered appropriate or safe in the opinion of the Investigator. If the available standard therapy is not considered appropriate or safe in the opinion of the Investigator, the rationale for ineligibility shall be provided and documented in the CRF. Furthermore, if in the opinion of the Investigator, the individual is intolerant to standard therapy, then drug information, toxicity, and grade must be documented in the CRF.

For Phase 1b Dose Expansion Part E (NSCLC), Inclusion Criterion 4(e) was modified as follows:

- Individuals must have been previously treated with a KRAS G12C inhibitor. [redacted]

For Phase 1b Dose Expansion Part G (NSCLC), Inclusion Criterion 4(g) was modified as follows:

- Individuals must not have additional validated oncogenic drivers in NSCLC, if known, e.g., activating alterations in genes such as *EGFR, ALK, BRAF (V600E), MET (exon 14), ROS1, RET, or NTRK1/2/3*.
- Individuals must have histologically or cytologically confirmed Stage IIIB-IIIC or Stage IV NSCLC, previously untreated in the advanced/metastatic setting and not suitable for curative intent radical surgery or radiation therapy. Staging will be according to the AJCC Staging System (8th ed [Amin et al. 2017]).
- Individuals may have received up to one 21-day cycle of pembrolizumab 200 mg Q3W; such therapy must have been initiated within 21 days (+14 days) prior to enrollment.
- Individuals enrolling in France must have a PD-L1-positive (≥ 50% of tumor cells) tumor as determined by IHC at a local or Sponsor-designated laboratory with CLIA, ISO/IEC, CAP, or other similar certification as per local guidelines including, but not limited to, IVDR compliance as applicable.

For Phase 1b Dose Expansion Part H (CRC), Inclusion Criterion 4(h) was modified as follows:

- Individuals must be appropriate candidates for study treatment and must have received at least 2 prior lines of therapy and have failed at least 1 prior oxaliplatin- or irinotecan-containing regimen for advanced or metastatic CRC. Study treatment is considered appropriate where approved measures are no longer effective or are not considered appropriate or safe in the opinion of the Investigator. If the available standard therapy is not considered appropriate or safe in the opinion of the Investigator, the rationale for ineligibility shall be provided and documented in the CRF. Furthermore, if in the opinion of the Investigator, the individual is intolerant to standard therapy, then drug information, toxicity, and grade must be documented in the CRF.

For all cohorts, Inclusion Criterion 8 was modified as follows:

8) Individuals must have discontinued from previous treatments, as shown in the table below:

**Previous Treatment**	**Length of Time Prior to Start of Study Drug(s)**
Cytotoxic therapies or targeted agents that are small molecule inhibitors	≥ ~~14~~ 21 days or ≥ 5 half-lives, whichever is shorter
Biologic agents that are large molecules including immunotherapy	≥ 21 days<br>For Cohort B4, ≥ 28 days
Radiotherapy<br>Limited-field radiotherapy with palliative intent<br>Other radiotherapy	 ≥ 7 days<br>≥ 21 days
Major surgery	≥ 28 days

9.10.1.3 Section 4.2 Exclusion Criteria

The first paragraph of the following exclusion criteria was modified as follows:

16) Individual has an active fungal, bacterial, and/or active untreated viral infection, including HIV or viral (A, B, or C) hepatitis (screening is not required unless mandated by local health authority). In France, screening is required for HIV and viral hepatitis, as indicated in Section 7.1.

9.10.1.4 Protocol Section 6.2.1 Discontinuation of Inadvertently Enrolled Participants

Text was modified as follows:

If the Sponsor or Investigator identifies a participant who did not meet enrollment criteria and was inadvertently enrolled, then the participant should be discontinued from study treatment, as ~~allowed~~ required by local regulations~~, unless there are extenuating circumstances that make it medically necessary for the patient to continue study treatment. If the Investigator and the Sponsor agree it is medically appropriate to continue, the Investigator must obtain documented approval from the Sponsor to allow the inadvertently enrolled participant to continue in the study, with or without treatment with investigational product~~. In France, the participant must be discontinued from study treatment. SFU is as outlined in the SoA (Section 7.1), Section 7.3 (Safety Assessments), and Section 7.4 (AEs and SAEs) of the protocol.

9.10.1.5 Protocol Section 7.1 Schedule of Assessments

Rows were added to the following tables as shown below:

Table 13: Schedule of Assessments for Participants Enrolled in Phase 1a

Screening, On-Study, and Post-treatment SoA													
	**Screening** (Day Relative to C1D1)		**On-Treatment Cycle = 21 days**							**Post-treatment**			**Instructions**
			**Cycle 1**			**Cycle 2**			**Cycle 3 and above**	**EOT[a]**	**Safety Follow-Up[b]**	**Long-Term Follow-Up[c]**	
			(±1 days)			(±1 days)			(±3 days)	(±7 days)	28 days (±7 days)	Every 3 months (±4 weeks)	
**Visit**	**≤ 28**	**≤ 14**	**D1**	**D8**	**D15**	**D1**	**D8**	**D15**	**D1**				
**Procedure**													
HIV and viral (A, B, and C) hepatitis test		X											

Table 14: Schedule for Participants Enrolled in Phase 1b (Except Part G) and Phase 2

Phase 1b (Except Part G) and Phase 2 Screening, On-Study, and Post-treatment SoA											
Part B to Part F	**Screening** (Day Relative to C1D1)		**On-Treatment Cycle = 21 days**					**Post-treatment**			**Instructions**
			**Cycle 1**			**Cycle 2**	**Cycle 3-n**	**EOT[b]**	**Safety Follow-Up[c]**	**Long-Term Follow-Up[d]**	
								(±7 days)	28 days (±7 days)	Every 3 months (±4 weeks)	
**Visit**	**≤ 28**	**≤ 14**	**D1**	**D8 (±1 day)[a]**	**D15 (±1 day)[a]**	**D1 (±3 days)**	**D1 (±3 days)**				
**Procedure**											
HIV and viral (A, B, and C) hepatitis test		X									

Table 19: Schedule of Assessments for Participants in Part G – Dose Optimization

**Part G**	**Prescreening**	**Screening**		**Treatment Period Cycle = 21 days**		**Posttreatment**			**Comments**
				Cycle 1	Cycle 2-n	EOT[a]	Safety Follow-Up[b]	Long-Term Follow-Up[c]	
Relative Day within Dosing Cycle		≤ 28	≤ 14	1	1	(± 7 days)	28 (±7 days)	Every 3 months (±4 weeks)	Screening: Procedures may be conducted over more than 1 day as long as all activities are completed within the allowable visit tolerance.
Visit Window (Days)		≤ 28		±1	±1 Cycle 2, ±3 Cycle 3+				
Visit Detail								T	T: Telephone Visit, if no study procedures are scheduled.
HIV and viral (A, B, and C) hepatitis test			X						

9.10.1.6 Protocol Section 7.11 Intraparticipant Dose Escalation

Paragraph 1 was modified as follows:

Intraparticipant dose escalation may be permitted following completion of the 21-day DLT period in Cycle 1 or after at least 1 scan (Cohort B8, [redacted]). The participant must not have experienced a DLT or DLT-equivalent AE, and any treatment-related AEs must have resolved to Grade 1 or baseline. Requests for intraparticipant dose escalation require documented Sponsor approval prior to treating a participant with a new dose.

If a participant in Phase 1a is dose escalated, procedures should continue as detailed SoA Table 13, and in addition, on Days 1 and 8 of the first cycle on the new dose, additional procedures should be performed as detailed in SoA Table 13. Additional PK assessments are not required for intraparticipant dose escalation in Phase 1b.

9.10.1.7 Protocol Section 9.2 Appendix 2: Clinical Laboratory Tests

Footnote (g) was added to this table as shown below:

**Clinical Laboratory Tests**	
**Hematology**[a,b]	**Clinical Chemistry**[a,b]
Leukocytes (WBC)	**Serum Concentrations of:**
Neutrophils	ALT
Lymphocytes	Albumin
Monocytes	Alkaline phosphatase
Eosinophils	Amylase[g]
Basophils	AST
Erythrocytes (RBC)	Bilirubin, direct
HGB	Bilirubin, total
HCT	BUN or blood urea
PLT	Calcium
	Creatinine
**Urinalysis**[b]	Creatine phosphokinase
Blood	Glucose (random)
Glucose	Lipase[g]
Ketones	Magnesium
pH	Phosphorous
Protein	Potassium
Specific gravity	Protein
Urine leukocyte esterase[d]	Sodium
**Thyroid Function**[b,e]	**Pregnancy Test**[b,c]
TSH	Urine or serum pregnancy test
FT4	
T3 or FT3[f]	**Coagulation**[b]
	PT/INR
	aPTT

Neutrophils reported by automated differential hematology instruments include both segmented and band forms. When a manual differential is needed to report the neutrophils, the segmented and band forms should be added together and recorded on the CRF, unless the CRF specifically provides an entry field for bands.

a Treatment and enrollment decisions will be based on local laboratory results.

b Local or Investigator-designated laboratory.

c For female patients of childbearing potential.

d Urine microscopy may be used in place of the urine leukocyte esterase assessment to test for the presence of WBC.

e FT4 should only be collected if TSH is not within normal limits.

f For Cohort B4, Cohort B9, and Part G only, thyroid testing should include TSH, FT4, and T3 or FT3.

g Radiological imaging is recommended in the case of amylase or lipase > 3 × ULN.

10.0 REFERENCES

Agazie YM, Hayman MJ. Molecular mechanism for a role of SHP2 in epidermal growth factor receptor signaling. *Mol Cell Biol*. 2003;23(21):7875-7886.

Ahronian LG, Sennott EM, Van Allen EM, et al. Clinical acquired resistance to RAF inhibitor combinations in BRAF-mutant colorectal cancer through MAPK pathway alterations. *Cancer Discov.* 2015;5(4):358-367.

Amin MB, Edge SB, Greene FL, et al, editors. *AJCC Cancer Staging Manual*. 8th ed. New York: Springer; 2017.

Aredo JV, Padda SK. Management of *KRAS*-mutant non-small cell lung cancer in the era of precision medicine. *Curr Treat Options Oncol.* 2018;19(8):43.

Bailey P, Chang DK, Nones K, et al. Genomic analyses identify molecular subtypes of pancreatic cancer. *Nature.* 2016;531(7592):47-52.

Bendell JC, Atreya CE, André T, et al. Efficacy and tolerability in an open-label phase I/II study of MEK inhibitor trametinib (T), BRAF inhibitor dabrafenib (D), and anti-EGFR antibody panitumumab (P) in combination in patients (pts) with BRAF V600E mutated colorectal cancer. *J Clin Oncol.* 2014;32(15_suppl):3515.

Blank C, Brown I, Peterson AC, et al. PD-L1/B7H-1 inhibits the effector phase of tumor rejection by T cell receptor (TCR) transgenic CD8+ T cells. *Cancer Res*. 2004;64(3):1140-1145.

Burns TF, Dragnev K, Fujiwara Y, et al. Efficacy and safety of olomorasib (LY3537982), a second-generation KRAS G12C inhibitor (G12Ci), in combination with pembrolizumab in patients with KRAS G12C-mutant advanced NSCLC [abstract]. J Clin Oncol. 2024 (in press)

Calvert AH, Newell DR, Gumbrell LA, O'Reilly S, Burnell M, Boxall FE, Siddik ZH, Judson IR, Gore ME, Wiltshaw E. Carboplatin dosage: prospective evaluation of a simple formula based on renal function. *J Clin Oncol.* 1989;7(11):1748-1756.

Campbell JD, Alexandrov A, Kim J, et al. Cancer Genome Atlas Research Network, Artyomov MN, Schreiber R, Govindan R, Meyerson M. Distinct patterns of somatic genome alterations in lung adenocarcinomas and squamous cell carcinomas. *Nat Genet.* 2016;48(6):607-616.

Canon J, Rex K, Saiki AY, et al: The clinical KRAS(G12C) inhibitor AMG 510 drives anti-tumour immunity. *Nature*. 2019;575(7781):217-223.

Chemnitz JM, Parry RV, Nichols KE, June CH, Riley JL. SHP-1 and SHP-2 associate with immunoreceptor tyrosine-based switch motif of programmed death 1 upon primary human T cell stimulation, but only receptor ligation prevents T cell activation. *J Immunol*. 2004;173(2):945-954.

Cockcroft DW, Gault MD. Prediction of creatinine clearance from serum creatinine. *Nephron*. 1976;16:31-41.

Coleman ML, Marshall CJ, Olson MF. RAS and RHO GTPases in G1-phase cell-cycle regulation. *Nat Rev Mol Cell Biol.* 2004;5(5):355-366.

Curran MA, Montalvo W, Yagita H, Allison JP. PD-1 and CTLA-4 combination blockade expands infiltrating T cells and reduces regulatory T and myeloid cells within B16 melanoma tumors. *Proc Natl Acad Sci USA*. 2010;107(9):4275-4280.

de Langen AJ, Johnson ML, Mazieres J, et al. Sotorasib versus docetaxel for previously treated non-small-cell lung cancer with KRASG12C mutation: a randomised, open-label, phase 3 trial. Lancet. 2023 Mar 4;401(10378):733-746. doi: 10.1016/S0140-6736(23)00221-0. Epub 2023 Feb 7. PMID: 36764316.

Dempsey JA, Chan EM, Burke TF, Beckmann RP. LY2835219, a selective inhibitor of CDK4 and CDK6, inhibits growth in preclinical models of human cancer [abstract]. In: Proceedings of the 104th Annual Meeting of the American Association for Cancer Research; 2013 Apr 6-10; Washington, DC. Philadelphia (PA): AACR; Cancer Res 2013;73(8 Suppl):Abstract nr LB-122. 2013;73(8 Suppl):Abstract nr LB-122.

Dinu D, Dobre M, Panaitescu E, et al. Prognostic significance of *KRAS* gene mutations in colorectal cancer--preliminary study. *J Med Life*. 2014;7(4):581-587.

Disis ML. Immune regulation of cancer. *J Clin Oncol*. 2010;28(29):4531-4538.

Dong ZY, Zhong WZ, Zhang XC, et al. Potential predictive value of TP53 and *KRAS* mutation status for response to PD-1 blockade immunotherapy in lung adenocarcinoma. *Clin Cancer Res.* 2017;23:3012-3024.

Dudley ME, Wunderlich JR, Yang JC, et al. Adoptive cell transfer therapy following non-myeloablative but lymphodepleting chemotherapy for the treatment of patients with refractory metastatic melanoma. *J Clin Oncol*. 2005;23(10):2346-2357.

Eisenhauer EA, Therasse P, Bogaerts J, et al. New response evaluation criteria in solid tumors: revised RECIST guideline (version 1.1). *Eur J Cancer*. 2009;45(2):228-247.

Ellingson BM, Wen PY, Cloughesy TF. Modified Criteria for Radiographic Response Assessment in Glioblastoma Clinical Trials. *Neurotherapeutics*. 2017;14(2):307-320.

Fakih MG, Falchook GS, Hong DS, et al. CodeBreaK101 subprotocol H: Phase 1b study evaluating combination of sotorasib, a KRASG12C inhibitor, and panitumumab (PMab), an EGFR inhibitor, in advanced KRAS p.G12C-mutated colorectal cancer (CRC). *Ann Oncol*. 2021;32 (suppl 5): S530-S582.

Fernández-Medarde A, Santos E. Ras in cancer and developmental diseases. *Genes Cancer*. 2011;2(3):344-358.

Ferrer I, Zugazagoitia J, Herbertz S, et al. *KRAS*-Mutant non-small cell lung cancer: From biology to therapy. *Lung Cancer.* 2018;124:53-64.

Flowers CR, Seidenfeld J, Bow EJ, et al. Antimicrobial prophylaxis and outpatient management of fever and neutropenia in adults treated for malignancy: American Society of Clinical Oncology clinical practice guideline. *J Clin Oncol.* 2013;31(6):794-810.

Food and Drug Administration. Highlights of Prescribing Information for Pembrolizumab. Published March 2021. https://www.accessdata.fda.gov/drugsatfda_docs/label/2021/125514s096lbl.pdf

Francisco LM, Sage PT, Sharpe AH. The PD-1 pathway in tolerance and autoimmunity. *Immunol Rev*. 2010;236:219-242.

Gadgeel S, Rodríguez-Abreu D, Speranza G, et al. Updated Analysis From KEYNOTE-189: Pembrolizumab or Placebo Plus Pemetrexed and Platinum for Previously Untreated Metastatic Nonsquamous Non-Small-Cell Lung Cancer. *J Clin Oncol*. 2020;38(14):1505-1517.

Giacomini KM, Balimane PV, Cho SK, et al: International Transporter Consortium commentary on clinically important transporter polymorphisms. *Clin Pharm Ther*. 2013, 94(1):23-26.

Giannakis M, Mu XJ, Shukla SA, et al. Genomic correlates of immune-cell infiltrates in colorectal carcinoma. *Cell Rep*. 2016;17(4):1206.

Goldberg RM, Montagut C, Wainberg ZA, et al. Optimising the use of cetuximab in the continuum of care for patients with metastatic colorectal cancer. *ESMO Open.* 2018;3(4):e000353.

Greenwald RJ, Freeman GJ, Sharpe AH. The B7 family revisited. *Annu Rev Immunol*. 2005;23:515-548.

Grothey A, Van Cutsem E, Sobrero A, et al. Regorafenib monotherapy for previously treated metastatic colorectal cancer (CORRECT): an international, multicentre, randomised, placebo-controlled, phase 3 trial. *Lancet*. 2013;381(9863):303-312.

Guo W, Wang SJ, Yang S, et al. A Bayesian interval dose-finding design addressing Ockham's razor: mTPI-2. *Contemp Clin Trials.* 2017;58:23-33.

Hirano F, Kaneko K, Tamura H, et al. Blockade of B7-H1 and PD-1 by monoclonal antibodies potentiates cancer therapeutic immunity. *Cancer Res*. 2005;65(3):1089-1096.

Hong DS, Fakih MG, Strickler JH, et al. KRAS(G12C) Inhibition with Sotorasib in Advanced Solid Tumors. *New Engl J Med.* 2020;383(13):1207-1217.

Hunder NN, Wallen H, Cao J, et al. Treatment of metastatic melanoma with autologous CD4+ T cells against NY-ESO-1. *N Engl J Med*. 2008;358(25):2698-2703.

Iasonos A, O'Quigley J: Design considerations for dose-expansion cohorts in phase I trials. *J Clin Oncol*. 2013;31(31):4014-4021.

Janes MR, Zhang J, Li LS, et al. Targeting *KRAS* mutant cancers with a covalent G12C-specific inhibitor. *Cell.* 2018;172(3):578-589.

Jänne PA, van den Heuvel MM, Barlesi F, et al. Selumetinib plus docetaxel compared with docetaxel alone and progression-free survival in patients with *KRAS*-mutant advanced non-small cell lung cancer: the SELECT-1 randomized clinical trial. *JAMA*. 2017;317(18):1844-1853.

Jänne PA, Rybkin II, Spira AI, et al. KRYSTAL-1: Activity and safety of adagrasib (MRTX849) in advanced/metastatic non-small-cell lung cancer (NSCLC) harboring *KRAS* G12C mutation. *Eur J Cancer.* 2020;138:S1-S2.

Johnson ML, Ou SHI, Barve M, et al. KRYSTAL-1: Activity and safety of adagrasib (MRTX849) in patients with colorectal cancer (CRC) and other solid tumors harboring a *KRAS* G12C mutation. *Eur J Cancer.* 2020;138:S2.

Jordan EJ, Kim HR, Arcila ME, et al. Prospective comprehensive molecular characterization of lung adenocarcinomas for efficient patient matching to approved and emerging therapies. *Cancer Discov*. 2017;7(6):596–609.

Kim JH, Kim HS, Kim BJ. Prognostic value of *KRAS* mutation in advanced non-small-cell lung cancer treated with immune checkpoint inhibitors: A meta-analysis and review. *Oncotarget*. 2017;8(29):48248-48252.

Kopetz S, Desai J, Chan E, et al. Phase II pilot study of vemurafenib in patients with metastatic BRAF-mutated colorectal cancer. *J Clin Oncol.* 2015;33(34):4032-4038.

Kopetz S, McDonough SL, Morris VK, et al. Randomized trial of irinotecan and cetuximab with or without vemurafenib in BRAF-mutant metastatic colorectal cancer (SWOG 1406). *J Clin Oncol.* 2017;35(4_suppl):520.

Kopetz S, Grothey A, Van Cutsem E, et al. BEACON CRC: a randomized, 3-arm, phase 3 study of encorafenib and cetuximab with or without binimetinib vs. choice of either irinotecan or FOLFIRI plus cetuximab in BRAF V600E-mutant metastatic colorectal cancer. *Ann Oncol.* 2019;30(4_suppl):mdz183.004.

Lee CK, Man J, Lord S, et al. Clinical and molecular characteristics associated with survival among patients treated with checkpoint inhibitors for advanced non-small cell lung carcinoma: a systematic review and meta-analysis. *JAMA Oncol*. 2018;4:210-216.

Lin NU, Lee EQ, Aoyama H, et al. Response assessment criteria for brain metastases: proposal from the RANO group. *Lancet Oncol*. 2015;16(6):e270-e278.

Lindsay CR, Jamal-Hanjani M, Forster M, et al. *KRAS*: Reasons for optimism in lung cancer. *Eur J Cancer*. 2018;99:20-27.

Lito P, Solomon M, Li LS, Hansen R, et al. Allele-specific inhibitors inactivate mutant *KRAS* G12C by a trapping mechanism. *Science.* 2016;351(6273):604-608.

Liu C, Lu H, Wang H et al. Combinations with allosteric SHP2 inhibitor TNO155 to block receptor tyrosine kinase signaling. *Clin Cancer Res*. 2021; 27(1) 342-354.

Long GV, Stroyakovskiy D, Gogas H, et al. Combined BRAF and MEK inhibition versus BRAF inhibition alone in melanoma. *N Engl J Med*. 2014;371(20):1877-1888.

Lou K, Steri V, Ge AY, et al. $KRAS^{G12C}$ inhibition produces a driver-limited state revealing collateral dependencies. *Sci Signal.* 2019;12(583):pii:eaaw9450.

Mayer RJ, Van Cutsem E, Falcone A, et al. Randomized trial of TAS-102 for refractory metastatic colorectal cancer. *N Engl J Med.* 2015;372(20):1909-1919.Mitchell EP, Lacouture M, Shearer H, et al. Final STEPP results of prophylacatic versus reactive skin toxicity (ST) treatment (tx) for panitumumab (pmab)-related ST in patients (pts) with metastatic colorectal cancer (mCRC). *J Clin Oncol*. 2009;27:18_suppl:CRA4027-CRA4027.

Murciano-Goroff YR, Heist RS, Kuboki Y, et al. Abstract CT028: A first-in-human phase 1 study of LY3537982, a highly selective and potent KRAS G12C inhibitor in patients with KRAS G12C-mutant advanced solid tumors. Cancer Res 15 April 2023; 83 (8_Supplement): CT028. https://doi.org/10.1158/1538-7445.AM2023-CT028

Musgrove EA, Caldon CE, Barraclough J, et al. Cyclin D as a therapeutic target in cancer. *Nat Rev Cancer.* 2011;11(8):558-572.

Nadal E, Chen G, Prensner JR, Shiratsuchi H, et al. *KRAS*-G12C mutation is associated with poor outcome in surgically resected lung adenocarcinoma. *J Thorac Oncol.* 2014;9(10):1513-1522.

Nakajima EC, Vallejo JJ, Akinboro O, et al. Outcomes of first-line immune checkpoint inhibitors with or without chemotherapy according to *KRAS* mutational status and PD-L1 expression in patients with advanced NSCLC: FDA pooled analysis. *J Clin Oncol*. 2022;40(16_suppl):9000-9001.

Nassar AH, Adib E, Kwiatkowski DJ. Distribution of KRASG12C Somatic Mutations across Race, Sex, and Cancer Type. *N Engl J Med.* 2021; 384(2):185-187.

Nishino M, Giobbie-Hurder A, Gargano M, et al. Developing a common language for tumor response to immunotherapy: immune-related response criteria using unidimensional measurements. *Clin Cancer Res.* 2013;19(14):3936-3943.

Nomi T, Sho M, Akahori T, et al. Clinical significance and therapeutic potential of the programmed death-1 ligand/programmed death-1 pathway in human pancreatic cancer. *Clin Cancer Res*. 2007;13(7):2151-2157.

O'Bryan JP. Pharmacological targeting of RAS: Recent success with direct inhibitors. *Pharmacol Res*. 2019;139:503-511.

Okazaki T, Maeda A, Nishimura H, Kurosaki T, Honjo T. PD-1 immunoreceptor inhibits B cell receptor-mediated signaling by recruiting src homology 2-domain-containing tyrosine phosphatase 2 to phosphotyrosine. *Proc Natl Acad Sci USA*. 2001;98(24):13866-13871.

Oken MM, Creech RH, Tormey DC, et al. Toxicity and response criteria of the Eastern Cooperative Oncology Group. *Am J Clin Oncol.* 1982;5(6):649-655.

Ostrem JM, Peters U, Sos ML, et al. K-Ras(G12C) inhibitors allosterically control GTP affinity and effector interactions. *Nature.* 2013;503(7477):548-551.

Parry RV, Chemnitz JM, Frauwirth KA, et al. CTLA-4 and PD-1 receptors inhibit T-cell activation by distinct mechanisms. *Mol Cell Biol*. 2005;25(21):9543-9553.

Patnaik A, Rosen LS, Tolaney SM, et al. Efficacy and safety of abemaciclib, an inhibitor of CDK4 and CDK6, for patients with breast cancer, non-small cell lung cancer, and other solid tumors. *Cancer Discov.* 2016;6(7):740-753.

Patricelli MP, Janes MR, Li LS, et al. Selective inhibition of oncogenic *KRAS* output with small molecules targeting the inactive state. *Cancer Discov*. 2016;6(3):316-329.

Pilon-Thomas S, Mackay A, Vohra N, Mulé JJ. Blockade of programmed death ligand 1 enhances the therapeutic efficacy of combination immunotherapy against melanoma. *J Immunol*. 2010;184(7):3442-3449.

Planchard D, Besse B, Groen HJM, et al. Dabrafenib plus trametinib in patients with previously treated BRAF (V600E)-mutant metastatic non-small cell lung cancer: an open-label, multicentre phase 2 trial. *Lancet Oncol.* 2016;17(7):984-993.

Posada MM, Morse BL, Turner PK, Kulanthaivel P, Hall SD, Dickinson GL. Predicting Clinical Effects of CYP3A4 Modulators on Abemaciclib and Active Metabolites Exposure Using Physiologically Based Pharmacokinetic Modeling. *J Clin Pharmacol.* 2020;60(7):915-930.

Prahallad A, Sun C, Huang S, et al. Unresponsiveness of colon cancer to BRAF(V600E) inhibition through feedback activation of EGFR. *Nature.* 2012;483(7387):100-103.

Price KA, Cohen EE. Current treatment options for metastatic head and neck cancer. *Curr Treat Options Oncol.* 2012;13(1):35-46.

Prior IA, Lewis PD, Mattos C. A comprehensive survey of Ras mutations in cancer. *Cancer Res*. 2012;72(10):2457-2467.

Puyol M, Martín A, Dubus P, et al. A synthetic lethal interaction between K-Ras oncogenes and Cdk4 unveils a therapeutic strategy for non-small cell lung carcinoma. *Cancer Cell.* 2010;18(1):63-73.

Reck M, Rodríguez-Abreu D, Robinson AG, et al. Updated Analysis of KEYNOTE-024: Pembrolizumab Versus Platinum-Based Chemotherapy for Advanced Non-Small-Cell Lung Cancer With PD-L1 Tumor Proportion Score of 50% or Greater. *J Clin Oncol.* 2019;37(7):537-546.

Riley JL. PD-1 signaling in primary T cells. *Immunol Rev*. 2009;229(1):114-125.

Rittmeyer A, Barlesi F, Waterkamp D, et al. Atezolizumab versus docetaxel in patients with previously treated non-small-cell lung cancer (OAK): a phase 3, open-label, multicentre randomised controlled trial. *Lancet.* 2017;389(10066):255–265.

Rizzo JD, Somerfield MR, Hagerty KL, et al. Use of epoetin and darbepoetin in patients with cancer: 2007 American Society of Clinical Oncology/American Society of Hematology clinical practice guideline update. *J Clin Oncol.* 2008;26(1):132-149.

Román M, Baraibar I, López I, et al. *KRAS* oncogene in non-small cell lung cancer: clinical perspectives on the treatment of an old target. *Mol Cancer.* 2018;17(1):33.

CCI [redacted]

Sheppard KA, Fitz LJ, Lee JM, et al. PD-1 inhibits T-cell receptor induced phosphorylation of the ZAP70/CD3zeta signalosome and downstream signaling to PKCtheta. *FEBS Lett*. 2004;574(1-3):37-41.

Siddiqui AD, Piperdi B. *KRAS* mutation in colon cancer: a marker of resistance to EGFR-I therapy. *Ann Surg Oncol*. 2010;17(4):1168-1176.

Skoulidis F, Li BT, Dy GK, et al: Sotorasib for Lung Cancers with *KRAS* p.G12C Mutation. *N Engl J Med*. 2021;384:2371-2381.

Smith TJ, Bohlke K, Lyman GH, et al. Recommendations for the Use of WBC Growth Factors: *J Clin Oncol*. 2015, 33(28):3199-3212.

Sosman JA, Kim KB, Schuchter L, et al. Survival in BRAF V600-mutant advanced melanoma treated with vemurafenib. *N Engl J Med.* 2012;366(8):707-714.

Spranger S, Koblish HK, Horton B, Scherle PA, Newton R, Gajewski TF. Mechanism of tumor rejection with doublets of CTLA-4, PD-1/PD-L1, or IDO blockade involves restored IL-2 production and proliferation of CD8(+) T cells directly within the tumor microenvironment. *J Immunother Cancer*. 2014;2:3.

Sun L, Hsu M, Cohen RB, et al. Association between *KRAS* variant status and outcomes with first-line immune checkpoint inhibitor-based therapy in patients with advanced non-small-cell lung cancer. *JAMA Oncol*. 2021;7:937-939.

Strome SE, Dong H, Tamura H, et al. B7-H1 blockade augments adoptive T-cell immunotherapy for squamous cell carcinoma. *Cancer Res*. 2003;63(19):6501-6505.

Tabernero J, Grothey A, Van Cutsem E, et al. Encorafenib Plus Cetuximab as a New Standard of Care for Previously Treated BRAF V600E-Mutant Metastatic Colorectal Cancer: Updated Survival Results and Subgroup Analyses from the BEACON Study. *J Clin Oncol*. 2021;39(4):273-284.

Tan C, Du X. *KRAS* mutation testing in metastatic colorectal cancer. *World J Gastroenterol.* 2012;18(37):5171-5180.

Thall PF, Simon RM, Estey EH. Bayesian sequential monitoring designs for single-arm clinical trials with multiple outcomes. *Stat Med*. 1995;14:357-379.

Thein K, Banks K, Saam J, et al. The prevalence of KRASG12C mutations utilizing circulating tumor DNA (ctDNA) in 80,911 patients with cancer. *J Clin Oncol.* 2020;38(15 suppl):3547-3547.

Van Emburgh BO, Arena S, Siravegna G, et al. Acquired RAS or EGFR mutations and duration of response to EGFR blockade in colorectal cancer. *Nature Commun.* 2016;7:13665.

Villanueva J, Vultur A, Lee JT, et al. Acquired resistance to BRAF inhibitors mediated by a RAF kinase switch in melanoma can be overcome by co-targeting MEK and IGF-1R/PI3K. *Cancer Cell.* 2010;18(6):683-695.

Wagle N, Van Allen EM, Treacy DJ, et al. MAP kinase pathway alterations in BRAF-mutant melanoma patients with acquired resistance to combined RAF/MEK inhibition. *Cancer Discov.* 2014;4(1):61-68.

CCI [redacted]

Weber J. Immune checkpoint proteins: a new therapeutic paradigm for cancer--preclinical background: CTLA-4 and PD-1 blockade. *Semin Oncol*. 2010;37(5):430-439.

Weiss J, Yaeger R, Johnson ML, et al. KRYSTAL-1: Adagrasib (MRTX849) as monotherapy or in combination with cetuximab in patients with colorectal cancer harboring a KRASG12C mutation. *Annals of Oncology.* 2021;32 (suppl 5): S1283-S1346.

Wilson CY, Tolias P. Recent advances in cancer drug discovery targeting RAS. *Drug Discov Today.* 2016;21(12):1915-1919.

Windon AL, Loaiza-Bonilla A, Jensen CE, et al. A *KRAS* wild type mutational status confers a survival advantage in pancreatic ductal adenocarcinoma. *J Gastrointest Oncol.* 2018;9(1):1-10.

Wolchok JD, Hoos A, O'Day S, et al. Guidelines for the evaluation of immune therapy activity in solid tumors: immune-related response criteria. *Clin Cancer Res.* 2009;15(23):7412-7420.

Xue JY, Zhao Y, Aronowitz J, et al. Rapid non-uniform adaptation to conformation-specific *KRAS*(G12C) inhibition. *Nature*. 2020;577(7790):421-425.

Yan F, Mandrekar SJ, Yuan Y. Keyboard: a novel Bayesian toxicity probability interval design for phase I clinical trials. *Clin Cancer Res.* 2017;23(15):3994-4003.

Zeitouni D, Pylayeva-Gupta Y, et al. *KRAS* mutant pancreatic cancer: no lone path to an effective treatment. *Cancers (Basel)*. 2016;8(4):pii:E45.

Zhang J, Zhang F, Niu R. Functions of Shp2 in cancer. *J Cell Mol Med*. 2015;19(9):2075-2083.

Zhang X, Schwartz JC, Guo X, et al. Structural and functional analysis of the costimulatory receptor programmed death-1 [published correction appears in *Immunity*. 2004;20(5):651]. *Immunity*. 2004;20(3):337-347.

Signature Page for VV-CLIN-173543 v10.0

Approval	PPD Director<br>22-May-2025 14:42:58 GMT+0000

Approval	PPD ian<br>23-May-2025 16:57:36 GMT+0000

Signature Page for VV-CLIN-173543 v10.0
