# Supplementary material for: Pan-tumor activity of olomorasib, a next-generation KRAS G12C inhibitor in KRAS G12C-mutant advanced solid tumors: a first-in-human study
Source: Nat Commun. 2026 Mar 12;17:3834. doi: 10.1038/s41467-026-69943-7 (PMC13121619; doi:10.1038/s41467-026-69943-7)
Supplement: Supplementary file 2 — Reporting Summary [file 41467_2026_69943_MOESM2_ESM.pdf]

nature portfolio

Corresponding author(s): Yonina R. Murciano-Goroff

Last updated by author(s): 12/10/2025

Reporting Summary

Nature Portfolio wishes to improve the reproducibility of the work that we publish. This form provides structure for consistency and transparency in reporting. For further information on Nature Portfolio policies, see our Editorial Policies and the Editorial Policy Checklist.

Statistics

For all statistical analyses, confirm that the following items are present in the figure legend, table legend, main text, or Methods section.

n/a	Confirmed	
☐	☒	The exact sample size (*n*) for each experimental group/condition, given as a discrete number and unit of measurement
☐	☒	A statement on whether measurements were taken from distinct samples or whether the same sample was measured repeatedly
☒	☐	The statistical test(s) used AND whether they are one- or two-sided<br>*Only common tests should be described solely by name; describe more complex techniques in the Methods section.*
☒	☐	A description of all covariates tested
☒	☐	A description of any assumptions or corrections, such as tests of normality and adjustment for multiple comparisons
☐	☒	A full description of the statistical parameters including central tendency (e.g. means) or other basic estimates (e.g. regression coefficient) AND variation (e.g. standard deviation) or associated estimates of uncertainty (e.g. confidence intervals)
☒	☐	For null hypothesis testing, the test statistic (e.g. *F*, *t*, *r*) with confidence intervals, effect sizes, degrees of freedom and *P* value noted<br>*Give P values as exact values whenever suitable.*
☒	☐	For Bayesian analysis, information on the choice of priors and Markov chain Monte Carlo settings
☒	☐	For hierarchical and complex designs, identification of the appropriate level for tests and full reporting of outcomes
☒	☐	Estimates of effect sizes (e.g. Cohen's *d*, Pearson's *r*), indicating how they were calculated

Our web collection on statistics for biologists contains articles on many of the points above.

Software and code

Policy information about availability of computer code

Data collection	No software was used
Data analysis	For stats analysis, no custom code used for analysis. All analyses were performed in SAS.<br>For biomarker analysis, software R (v 4.4.0) was used, unless otherwise specified<br>For PK figure: Software : Phoenix 32 version 8.3 copyright © Certara L.P was used for summary stat analysis of the concentration data per dose and protocol nominal time and the output exported in csv file<br>Software : RStudio Pro 2023.12.1 build 402.prol running R version 4.3.2 copyright © Posit Software PBC was used to create the graph.<br>for supplemental figure 2B box plot of AUC versus dose<br>Software : Phoenix 32 version 8.3 copyright © Certara L.P was used for the NCA analysis and generation of the AUC. The output was exported in xls.<br>Software : SigmaPlot for windows version 11.0 copyright © Systat software inc was used for graphical representation.

For manuscripts utilizing custom algorithms or software that are central to the research but not yet described in published literature, software must be made available to editors and reviewers. We strongly encourage code deposition in a community repository (e.g. GitHub). See the Nature Portfolio guidelines for submitting code & software for further information.

Data

Policy information about availability of data

All manuscripts must include a data availability statement. This statement should provide the following information, where applicable:

- Accession codes, unique identifiers, or web links for publicly available datasets
- A description of any restrictions on data availability
- For clinical datasets or third party data, please ensure that the statement adheres to our policy

Eli Lilly and Company provides access to all individual data collected during the trial, after anonymization, with the exception of pharmacokinetic, genomic, or genetic data. Data are available to request 6 months after the indication studied has been approved in the US and EU and after primary publication acceptance, whichever is later. No expiration date of data requests is currently set once data are made available. Access is provided after a proposal has been approved by an independent review committee identified for this purpose and after receipt of a signed data sharing agreement. Data and documents, including the study protocol, statistical analysis plan, clinical study report, and blank or annotated case report forms, will be provided in a secure data sharing environment. For details on submitting a request, see the instructions provided at www.vivli.org. Source data are provided with this paper.

Research involving human participants, their data, or biological material

Policy information about studies with human participants or human data. See also policy information about sex, gender (identity/presentation), and sexual orientation and race, ethnicity and racism.

Reporting on sex and gender	Provided in manuscript in Supplemental Table 1. Participants were enrolled irrespective of their sex. Any data on sex and gender was self reported and collected at each clinical trial site. The terms of sex and gender were use appropriately throughout the manuscript. There was no efficacy analysis conducted related to sex or gender due to limitations including the heterogenous population, different dose levels, and various lines of therapy, as well as the assumption that there is no sex bias in KRAS G12C mutation frequency, therefore, prior analyses were not investigated during protocol design.
Reporting on race, ethnicity, or other socially relevant groupings	Provided in manuscript in Supplemental Table 1. Participants were screened and enrolled irrespective of their race/ethnicity.
Population characteristics	Detailed baseline characteristics were reported in Supplemental Table 1.
Recruitment	Participants with KRAS G12C-mutant advanced cancers were recruited at participating enrolling centers. There is no identifiable self-selection or other bias, as all participants potentially meeting the eligibility criteria were evaluated for potential enrollment.
Ethics oversight	The study protocol was approved by the institutional review board (IRB) or independent ethics committee (IEC) at each participating site.<br><br>Participants were compensated for completed interviews as outlined in the site-specific informed consent process and in accordance with local regulations.

Note that full information on the approval of the study protocol must also be provided in the manuscript.

Field-specific reporting

Please select the one below that is the best fit for your research. If you are not sure, read the appropriate sections before making your selection.

[x] Life sciences [ ] Behavioural & social sciences [ ] Ecological, evolutionary & environmental sciences

For a reference copy of the document with all sections, see nature.com/documents/nr-reporting-summary-flat.pdf

Life sciences study design

All studies must disclose on these points even when the disclosure is negative.

Sample size	No sample size/power calculations were performed. The same size was determined to allow appropriate assessment of safety and anti-tumor activity. Phase 1a followed an mTPI-2 with cohorts of a minimum of 3 participants. Each dose cohort could be backfilled up<br>to approximately 40 participants to further investigate the safety, PK, and clinical activity, with a total of approximately 120 participants enrolled in Phase 1a.<br>Each of the Phase 1b cohorts enrolled approximately 20 participants (except for Cohort B1, which may enroll up to approximately 40 participants). Sample sizes were selected to allow assessment of safety and antitumor activity. It can provide adequate precision for the estimated incidence rate of the<br>following quantities of interest: (1) participants having a specified AE or (2) participants showing a response (CR/PR) or clinical benefit to treatment. With these sample sizes example point estimates of incidence rates and corresponding 2-sided Clopper-Pearson 95% CIs.
Data exclusions	No data was excluded from this analysis.
Replication	Each statistical analysis was conducted independently by two analysts: a primary analyst and a validation analyst. The validation analysis

independently verified the results, and all analyses yielded matching outcomes between the two analysts.

Randomization	The analysis was based on the Phase 1a/b cohorts and no formal testing was conducted. The implementation of randomization was not needed.
Blinding	The analysis was based on the Phase 1a/b cohorts and no formal testing was conducted. The implementation of blinding was not needed.

Reporting for specific materials, systems and methods

We require information from authors about some types of materials, experimental systems and methods used in many studies. Here, indicate whether each material, system or method listed is relevant to your study. If you are not sure if a list item applies to your research, read the appropriate section before selecting a response.

Materials & experimental systems

n/a	Involved in the study
☒	☐ Antibodies
☒	☐ Eukaryotic cell lines
☒	☐ Palaeontology and archaeology
☒	☐ Animals and other organisms
☐	☒ Clinical data
☒	☐ Dual use research of concern
☒	☐ Plants

Methods

n/a	Involved in the study
☒	☐ ChIP-seq
☒	☐ Flow cytometry
☒	☐ MRI-based neuroimaging

Clinical data

Policy information about clinical studies

All manuscripts should comply with the ICMJE guidelines for publication of clinical research and a completed CONSORT checklist must be included with all submissions.

Clinical trial registration	NCT04956640
Study protocol	Included as a supplemental material, along with the statistical analysis plan
Data collection	Provided in results and methods on pages 7 and 14. Data were collected from 29 July 2021 to 05 July 2024 in a clinical or hospital setting.
Outcomes	Provided in the manuscript on page 16. The primary objective of the phase 1a dose escalation was to determine the recommended phase 2 dose (RP2D) of olomorasib. The phase 1b dose expansion further evaluated the safety and efficacy and tolerability of olomorasib in specific solid tumor types. Secondary objectives were to characterize the PK, determine the preliminary anti-tumor activity of olomorasib, including disease control rate (DCR), overall response rate (ORR), duration of response (DOR), and progression-free survival (PFS) as assessed per RECIST v1.1, and evaluate the intracranial ORR and DOR based on modified RECIST v1.1. Exploratory objectives included correlation of boilermaker characteristics in tumor tissue or blood with clinical benefit and measurement of changes in boilermakers in response to study treatment after progression.

Plants

Seed stocks	*Report on the source of all seed stocks or other plant material used. If applicable, state the seed stock centre and catalogue number. If plant specimens were collected from the field, describe the collection location, date and sampling procedures.*
Novel plant genotypes	*Describe the methods by which all novel plant genotypes were produced. This includes those generated by transgenic approaches, gene editing, chemical/radiation-based mutagenesis and hybridization. For transgenic lines, describe the transformation method, the number of independent lines analyzed and the generation upon which experiments were performed. For gene-edited lines, describe the editor used, the endogenous sequence targeted for editing, the targeting guide RNA sequence (if applicable) and how the editor was applied.*
Authentication	*Describe any authentication procedures for each seed stock used or novel genotype generated. Describe any experiments used to assess the effect of a mutation and, where applicable, how potential secondary effects (e.g. second site T-DNA insertions, mosiacism, off-target gene editing) were examined.*
