# Supplementary material for: Pan-tumor activity of olomorasib, a next-generation KRAS G12C inhibitor in KRAS G12C-mutant advanced solid tumors: a first-in-human study
Source: Nat Commun. 2026 Mar 12;17:3834. doi: 10.1038/s41467-026-69943-7 (PMC13121619; doi:10.1038/s41467-026-69943-7)
Supplement: Supplementary file 3 — Transparent Peer Review file [file 41467_2026_69943_MOESM3_ESM.pdf]

nature portfolio

Peer Review File

Pan-tumor activity of olomorasib, a next-generation KRAS G12C inhibitor in KRAS G12C-mutant advanced solid tumors: a first-in-human study

Corresponding Author: Dr Yonina Murciano-Goroff

This manuscript has been previously reviewed at another journal. This document only contains information relating to versions considered at Nature Communications.

This file contains all reviewer reports in order by version, followed by all author rebuttals in order by version.

Version 0:

Reviewer comments:

Reviewer #2

(Remarks to the Author)
All questions and comments previously raised have been fully resolved by the authors in the revised manuscript. I have no further requests

Reviewer #4

(Remarks to the Author)
This is an interesting manuscript to present new results regarding safety and response to treatment with olomorasib, in particular, examining different doses of treatment.

I have the following comment for clarification:

line 117 - recruitment was open to 5 July 2024 and line 124 a data cutoff date of 5 July 2024 was used. So this means that some patients are included in recruitment but not treatment or adverse event or response analyses? Had all 195 recruited patients been assessed for the first 21-day DLT period?

Should the date of last recruitment of patients in this analysis be earlier than 5 July 2024?

Open Access This Peer Review File is licensed under a Creative Commons Attribution 4.0 International License, which permits use, sharing, adaptation, distribution and reproduction in any medium or format, as long as you give appropriate credit to the original author(s) and the source, provide a link to the Creative Commons license, and indicate if changes were made.
In cases where reviewers are anonymous, credit should be given to 'Anonymous Referee' and the source.
The images or other third party material in this Peer Review File are included in the article's Creative Commons license, unless indicated otherwise in a credit line to the material. If material is not included in the article's Creative Commons license and your intended use is not permitted by statutory regulation or exceeds the permitted use, you will need to obtain permission directly from the copyright holder.
To view a copy of this license, visit https://creativecommons.org/licenses/by/4.0/
